# Supplementary material for: A Network-Centric Framework for the Evaluation of Mutual Exclusivity Tests on Cancer Drivers
Source: Front Genet. 2021 Nov 26;12:746495. doi: 10.3389/fgene.2021.746495 (PMC8664367; doi:10.3389/fgene.2021.746495)
Supplement: Supplementary file 1 [file DataSheet1.PDF]

Supplementary Material for the paper "A Network-centric Framework for the Evaluation of Mutual Exclusivity Tests on Cancer Drivers"

Robustness Analysis of Evaluations Based on Defined Metrics

We also investigate the robustness of our results with respect to *robustness_iterations* value, the p-value significance threshold value, the reference gene set and the employed PPI network. For *robustness_iterations*, we try the values 300 and 500. For p-value significance threshold, we try additional values of 0.01 and 0.1. Regarding the reference gene set, we try using a subset of CGC genes to include only those which have SNV type of mutations in cancer (378 out of 723 genes). To this end, we filter out the genes where the *mutation type* column consists of only A (amplification), D (large deletion) or T (translocation). Additionally, we use an alternative source named *intoGen* [Martínez-Jiménez et al., 2020] to compile reference cancer genes. We download $Unfiltered\_driver\_results\_05.tsv$ file (2020-02-02 release) and include the genes where FILTER column is *PASS*, which results in 503 genes. For the PPI network, we try different confidence threshold values for filtering IntAct. 0.45 threshold value is commonly used in the literature to filter out the interactions with low confidence [Sügis et al., 2019, Porras et al., 2020] . Additionally, to observe the effect of using a lower threshold than the current one, we also tried filtering IntAct with a threshold value of 0.25. Lastly, we utilize two alternative networks in our analyses: $HINT + HI2012$ [Das and Yu, 2012, Yu et al., 2011, Leiserson et al., 2015] and STRING network [Szklarczyk D et al., 2018]. For the latter, we download the file *9606.protein.physical.links.v11.0.txt* and only use physical interactions with scores greater than 700. This results in 9,524 nodes and 146,120 edges.

The results with these different settings are available in Table S2-S9 for $t = 20$ and $c = X_1$. Table S2 and S3 show the results with *robustness_iterations* value 300 and 500, respectively. When *robustness_iterations* value is increased from 100 to 300, the ranking of the ME methods based on F1 score remains the same for all the cancer types except for BLCA and BRCA (Table S2). Similarly, when the *robustness_iterations* value is increased from 100 to 500, the ranking of the ME methods based on F1 score changes only for BLCA and BRCA (Table S3). Table S4 shows the results where p-value significance threshold is decreased from 0.05 to 0.01. We observe that the 0.01 threshold is too strict for most of the methods as evident from low sensitivity values for most cancer types. In fact, Fisher's Exact Test predicts no mutually exclusive cases for six out of eight cancer types. When we use the p-value threshold of 0.1, we observe an overall improvement in F1 scores for all cancer types and for all the methods except for MEGSA which shows decreased F1 scores for four cancer types (Table S5). The observed increase in F1 scores for the majority of the cases is due to the large increase in sensitivity values. Additionally, for some cases precision values also increase with this less stringent p-value threshold. In particular, we observe a dramatic increase in F1/$F1_{strict}$ scores for LUAD cancer type. In terms of ranking of the methods, we observe a difference in BLCA and BRCA types. For BLCA, WeXT's ranking improves from fourth place to first place whereas MEMO's ranking decreases from first place to third place. We observe a similar switch in ranking of the methods for BRCA. When CGC_{SNV} is used as the reference gene set, F1 score-based ranking of the ME methods changes for three cancer types: BLCA, BRCA and LUSC (Table S6). Though, among these three cancer types, the top ranking method remains the same for BRCA and LUSC. When *IntoGen* is used as the reference set, we only observe a change in ranking of the ME methods for BLCA and BRCA types (Table S7). Next, for the experiments where we try different confidence thresholds for IntAct or switch to the $HINT + HI2012$ network, the only

cancer types where the ranking of the ME methods change are BLCA and BRCA (Table S8-S10). When we use the STRING network, we observe a decrease in $F1/F1_{strict}$ scores of all the methods except for WeXT for BLCA and UCEC cancer types as compared to the results obtained with the IntAct network (Table S11). For COADREAD, LUAD and STAD we observe both increases and decreases in F1/ $F1_{strict}$ scores. Lastly, all the methods show decreased F1/ $F1_{strict}$ scores in LUSC and SKCM. The ranking of the methods based on F1 score and $F1_{strict}$ score differs for BRCA and COADREAD datasets. For BRCA, WeXT and DISCOVER Strat switch positions in the ranking based on F1/ $F1_{strict}$ scores. For COADREAD, MEMO's ranking decreases from first position to third position showing that its top performance is not preserved with stricter versions of evaluation metrics. Additionally, the ranking of the methods change in five out of eight cancer types as compared to the results obtained with IntAct network suggesting that the input PPI plays a role in the performance of ME methods.

Results of robustness analyses for $t = 20$ and $c = X_2$ setting is available in Table S13-S20. When *robustness_iterations* is increased to 300 or to 500, the ranking of the ME methods remains the same for all the cancer types. Using a stricter p-value significance threshold of 0.01 results in too few mutual exclusivities predicted for many of the cancer types (Table S16). For instance, for LUSC, the sensitivity values are zero for all the methods. Similarly, for BLCA, SKCM and STAD cancer types the maximum sensitivity value across the ME methods is 0.1. When p-value threshold is switched to 0.1, we observe changes in both directions for different cancer types (Table S17). For BRCA, $F1/F1_{strict}$ scores of all the methods either remain the same or decrease. On the other hand, all the methods have increased $F1/F1_{strict}$ scores for COADREAD. We also observe a change in the ranking of the methods when we change the p-value threshold from 0.05 to 0.1. For LUAD, SKCM and UCEC, we observe significant increase in $F1/F1_{strict}$ scores of all the methods. For LUSC and STAD, we see both increases and decreases in $F1/F1_{strict}$ scores. In particular, WeXT and DISCOVER show dramatic increases in $F1/F1_{strict}$ scores for STAD type. Overall, we can conclude that the p-value threshold of 0.1 leads to a better performance across the employed methods. When the reference gene set is changed to CGC_{SNV} or to *IntoGen*, the F1 score-based ranking of the methods only change for BLCA (Table S18-S19). When we use a version of IntAct filtered with confidence value 0.25, the ranking remains the same for all the cancer types except for BLCA, BRCA and LUSC (Table S20). When the confidence value threshold is increased to 0.45, the number of considered CGC-CGC gene pairs decreases to values that are < 20 for all the cancer types except for SKCM and UCEC (Table S21). For SKCM and UCEC, we observe the same ranking as in our original parameter settings. Switching to $HINT + HI2012$ network also leads to the inclusion of too few gene pairs for many cancer types (Table S22). For the rest, the ranking of the ME methods is in accordance with our original results. When we use the STRING network as the input PPI, we observe very few CGC-CGC pairs for BLCA, BRCA and LUSC. Comparing these results with those obtained with the IntAct network, for COADREAD, MEMo and WeXT are the only methods that show decreased $F1/F1_{strict}$ scores where the magnitude of change is much larger for MEMo. However, these changes do not lead to a difference in the ranking of the methods. For LUAD, we observe large improvements in $F1/F1_{strict}$ scores of all the methods. Lastly, for SKCM, STAD and UCEC we observe smaller $F1/F1_{strict}$ scores in almost all the cases (Table S23). These results reveal that the change of the network leads to distinct changes in different cancer types.

Robustness analyses for $t = 5$ setting is available in Table S25-S35 and in Table S37-S47 for $c = X_1$ and $c = X_2$, respectively. We observe similar patterns compared to the $t = 20$ setting. For instance, changing the p-value threshold to 0.01 decreases the $F1/F1_{strict}$ scores overall whereas increasing the threshold to 0.1 also increases the $F1/F1_{strict}$ scores. Overall, changing the different settings of the analysis do not lead a change in the ranking of the methods.

We also assess whether the F1 scores improve or worsen when different confidence thresholds are used to filter the IntAct network. Increasing the interaction confidence threshold increases the support of both the reference pair and the control pair with respect to X_2, since they are both interacting pairs. In parallel with this, for the $t = 5$ setting where we have adequate number of gene pairs for all the cancer types, we observe an increased F1 score for all the methods for all the cancer types except for BRCA and LUAD. For these two cancer types, we observe both increases and decreases in F1 scores.

On the other hand, it is difficult to propose a similar argument for $c = X_1$ setting, since increasing the interaction confidence threshold increases the support of the reference pair but decreases that of the control pair. This is due to the possibility of considering a random reference cancer gene as a non-neighbor with the 0.45 threshold even though it would considered as a neighbor with a lower confidence threshold e.g. 0.35.

Details of running SELECT

We note that SELECT diverges from the rest of the methods in terms of the output ME scores. The suggested mode of use is based on ASC scores rather than p-values, which is the case with the other ME methods. As such, the SELECT outputs are not directly comparable to those of the rest of the methods. This by itself is not an issue as the framework evaluates each ME method separately and an appropriate threshold similar to the p-value threshold used in other methods can be assigned for the ASC scores of SELECT. However, we find that the ASC threshold determined by SELECT to detect mutually exclusive gene pairs is too stringent. For instance applying this threshold on the COADREAD data results in only 720 gene pairs to be mutually exclusive and among those only one of them appears in the IntAct network as an interacting pair. Therefore, to provide a fair comparison we use a less stringent approach for determining ME pairs from the SELECT outputs. We first find the number of ME pairs identified by the other benchmark ME detection methods and then employ two alternatives. In the first one we calculate the average number of significant ME pairs as detected by the benchmark methods and deem the top that many gene pairs as significantly mutually exclusive among the SELECT-ranked gene pairs. In the second alternative we employ the maximum number of significant ME pairs detected by any benchmark method instead of the average. Yet another issue complicating the comparison of SELECT with the other ME methods is the fact that for some gene pairs SELECT assigns the direction of the relationship as CO signifying "co-occurence" rather than ME. Furthermore, for some gene pairs the resulting ASC scores are assigned as 'nan'. Since our framework requires a significance comparison between an interacting pair of known cancer drivers and a control pair, it is not exactly applicable for such cases.

ME evaluations based on defined metrics on other cancer types

We observe that the methods report a small number of significant p-values for BLCA data (Table S1-a). In line with this, we observe smaller sensitivity values for BLCA data compared to the results on COADREAD data. For BRCA dataset, we observe that the top F1 score is obtained by DISCOVER Strat indicating the benefit of considering subtype information for BRCA (Table S1-b). When we compare the values of the conventional and strict versions of the metrics, we observe that DISCOVER Strat's precision$_{strict}$ is significantly larger than its precision value whereas we observe negligible changes for the other methods. Accordingly, DISCOVER Strat's F1$_{strict}$ score is slightly lower than its F1 score whereas we observe significant drops for the other methods. We also observe that MEGSA and Fisher's Exact Test perform significantly worse than the other methods. Interestingly MEGSA ranks the best in terms of F1 score for LUSC dataset (Table S1-e). However, we should point out that the methods report a small number of significant p-values similar to the BLCA dataset. WeXT shows a dramatically better performance on SKCM dataset (Table S1-f). For UCEC dataset, we observe that Fisher's Exact Test and MEGSA perform poorly due to their conservative calculation of p-values when compared to DISCOVER and WeXT (Table S1-h). Table S13 show the analogous results when the control group is defined as $\mathcal{X}_2$. We observe that the analysis includes less than 50 pairs for BLCA, BRCA and LUSC. Again, we observe that Fisher's Exact Test and MEGSA report zero or very few number of significant p-values across the majority of the the cancer types. For LUAD, SKCM, STAD and UCEC, WeXT gives the largest F1-values. Similar to the results obtained with $\mathcal{X}_1$, MEGSA ranks the top in terms of F1-score on LUSC dataset. MEGSA and MEMo results are not available for some cancer types since we are unable to run these methods due to memory issues.

Table 1: Results of network-centric ME evaluation framework with $\mathcal{G}$ = Intact (w conf. threshold 0.35), $\mathcal{S}$ = CGC, c= X_1, p_t = 0.05, t=20, robustness_iterations = 100

(a) Metrics for BLCA data. (411 samples | 56 CGC-CGC pairs)

Method	Precision	Sensitivity	F1 Score	$\text{Precision}_{\text{strict}}$	$\text{Sensitivity}_{\text{strict}}$	F1 $_{\text{strict}}$ Score
DISCOVER	0.800	0.075	0.137	0.800	0.075	0.137
Fisher's Exact Test	1.000	0.036	0.069	1.000	0.036	0.069
MEGSA	1.000	0.074	0.138	1.000	0.074	0.138
MEMO	0.800	0.079	0.144	0.800	0.079	0.144
WExT	0.571	0.077	0.136	0.571	0.077	0.136

(b) Metrics for BRCA data. (1026 samples | 34 CGC-CGC pairs)

Method	Precision	Sensitivity	F1 Score	$\text{Precision}_{\text{strict}}$	$\text{Sensitivity}_{\text{strict}}$	F1 $_{\text{strict}}$ Score
DISCOVER	0.609	0.452	0.519	0.579	0.355	0.440
DISCOVER Strat	0.744	0.492	0.593	0.811	0.462	0.589
Fisher's Exact Test	1.000	0.061	0.114	1.000	0.061	0.115
MEGSA	1.000	0.059	0.111	1.000	0.059	0.111
MEMO	0.700	0.483	0.571	0.706	0.414	0.522
WExT	0.612	0.526	0.566	0.615	0.421	0.500

(c) Metrics for COADREAD data. (498 samples | 196 CGC-CGC pairs)

Method	Precision	Sensitivity	F1 Score	$\text{Precision}_{\text{strict}}$	$\text{Sensitivity}_{\text{strict}}$	F1 $_{\text{strict}}$ Score
DISCOVER	0.661	0.220	0.331	0.708	0.183	0.291
DISCOVER Strat	0.727	0.041	0.078	0.727	0.041	0.078
Fisher's Exact Test	0.500	0.031	0.058	0.500	0.031	0.058
MEGSA	0.611	0.056	0.103	0.588	0.051	0.094
MEMO	0.658	0.329	0.439	0.647	0.237	0.347
WExT	0.676	0.403	0.505	0.725	0.329	0.453

(d) Metrics for LUAD data. (568 samples | 92 CGC-CGC pairs)

Method	Precision	Sensitivity	F1 Score	$\text{Precision}_{\text{strict}}$	$\text{Sensitivity}_{\text{strict}}$	F1 $_{\text{strict}}$ Score
DISCOVER	0.773	0.099	0.176	0.789	0.088	0.158
Fisher's Exact Test	0.000	0.000	NaN	0.000	0.000	NaN
MEGSA	0.667	0.022	0.043	0.667	0.022	0.043
MEMO	0.722	0.149	0.248	0.733	0.126	0.215
WExT	0.625	0.174	0.273	0.667	0.163	0.262

(e) Metrics for LUSC data. (485 samples | 38 CGC-CGC pairs)

Method	Precision	Sensitivity	F1 Score	$\text{Precision}_{\text{strict}}$	$\text{Sensitivity}_{\text{strict}}$	F1 $_{\text{strict}}$ Score
DISCOVER	1.0	0.054	0.103	1.0	0.054	0.102
Fisher's Exact Test	1.0	0.053	0.100	1.0	0.053	0.101
MEGSA	1.0	0.158	0.273	1.0	0.158	0.273
MEMO	1.0	0.108	0.195	1.0	0.108	0.195
WExT	1.0	0.114	0.205	1.0	0.114	0.205

(f) Metrics for SKCM data. (468 samples | 458 CGC-CGC pairs)

Method	Precision	Sensitivity	F1 Score	$\text{Precision}_{\text{strict}}$	$\text{Sensitivity}_{\text{strict}}$	F1 $_{\text{strict}}$ Score
DISCOVER	0.800	0.045	0.084	0.833	0.045	0.085
Fisher's Exact Test	1.000	0.004	0.009	1.000	0.004	0.008
MEGSA	0.889	0.018	0.034	0.889	0.018	0.035
WExT	0.717	0.121	0.207	0.730	0.116	0.200

(g) Metrics for STAD data. (438 samples | 140 CGC-CGC pairs)

Method	Precision	Sensitivity	F1 Score	$\text{Precision}_{\text{strict}}$	$\text{Sensitivity}_{\text{strict}}$	F1 $_{\text{strict}}$ Score
DISCOVER	0.667	0.122	0.206	0.684	0.099	0.173
Fisher's Exact Test	0.000	0.000	NaN	0.000	0.000	NaN
MEGSA	0.667	0.014	0.028	0.667	0.014	0.027
WExT	0.634	0.190	0.292	0.636	0.153	0.247

(h) Metrics for UCEC data. (531 samples | 1356 CGC-CGC pairs)

Method	Precision	Sensitivity	F1 Score	$\text{Precision}_{\text{strict}}$	$\text{Sensitivity}_{\text{strict}}$	F1 $_{\text{strict}}$ Score
DISCOVER	0.651	0.177	0.279	0.711	0.143	0.238
Fisher's Exact Test	0.833	0.007	0.015	0.833	0.007	0.014
MEGSA	0.786	0.008	0.016	0.786	0.008	0.016
WExT	0.616	0.278	0.383	0.665	0.227	0.338

Table 2: Results of network-centric ME evaluation framework with $\mathcal{G}$ = Intact (w conf. threshold 0.35), $\mathcal{S}$ = CGC, c= X_1, p_t = 0.05, t=20, robustness_iterations = 300

(a) Metrics for BLCA data. (411 samples | 56 CGC-CGC pairs)

Method	Precision	Sensitivity	F1 Score	$\text{Precision}_{\text{strict}}$	$\text{Sensitivity}_{\text{strict}}$	F1 $_{\text{strict}}$ Score
DISCOVER	0.800	0.075	0.138	0.800	0.075	0.137
Fisher's Exact Test	1.000	0.037	0.071	1.000	0.037	0.071
MEGSA	1.000	0.073	0.136	1.000	0.073	0.136
MEMO	0.667	0.078	0.140	0.667	0.078	0.140
WExT	0.571	0.075	0.133	0.571	0.075	0.133

(b) Metrics for BRCA data. (1026 samples | 34 CGC-CGC pairs)

Method	Precision	Sensitivity	F1 Score	$\text{Precision}_{\text{strict}}$	$\text{Sensitivity}_{\text{strict}}$	F1 $_{\text{strict}}$ Score
DISCOVER	0.652	0.484	0.556	0.632	0.387	0.480
DISCOVER Strat	0.762	0.516	0.615	0.833	0.484	0.612
Fisher's Exact Test	1.000	0.062	0.118	1.000	0.062	0.117
MEGSA	1.000	0.059	0.111	1.000	0.059	0.111
MEMO	0.667	0.467	0.549	0.667	0.400	0.500
WExT	0.600	0.536	0.566	0.632	0.429	0.511

(c) Metrics for COADREAD data. (498 samples | 196 CGC-CGC pairs)

Method	Precision	Sensitivity	F1 Score	$\text{Precision}_{\text{strict}}$	$\text{Sensitivity}_{\text{strict}}$	F1 $_{\text{strict}}$ Score
DISCOVER	0.659	0.218	0.327	0.707	0.184	0.292
DISCOVER Strat	0.727	0.042	0.079	0.727	0.042	0.079
Fisher's Exact Test	0.545	0.031	0.059	0.545	0.031	0.059
MEGSA	0.611	0.056	0.103	0.588	0.051	0.094
MEMO	0.660	0.338	0.447	0.662	0.247	0.360
WExT	0.684	0.406	0.510	0.724	0.328	0.451

(d) Metrics for LUAD data. (568 samples | 92 CGC-CGC pairs)

Method	Precision	Sensitivity	F1 Score	$\text{Precision}_{\text{strict}}$	$\text{Sensitivity}_{\text{strict}}$	F1 $_{\text{strict}}$ Score
DISCOVER	0.750	0.103	0.182	0.700	0.080	0.144
Fisher's Exact Test	0.000	0.000	NaN	0.000	0.000	NaN
MEGSA	0.667	0.022	0.043	0.667	0.022	0.043
MEMO	0.722	0.149	0.248	0.733	0.126	0.215
WExT	0.667	0.179	0.282	0.700	0.156	0.255

(e) Metrics for LUSC data. (485 samples | 38 CGC-CGC pairs)

Method	Precision	Sensitivity	F1 Score	$\text{Precision}_{\text{strict}}$	$\text{Sensitivity}_{\text{strict}}$	F1 $_{\text{strict}}$ Score
DISCOVER	1.0	0.054	0.103	1.0	0.054	0.102
Fisher's Exact Test	1.0	0.053	0.100	1.0	0.053	0.101
MEGSA	1.0	0.158	0.273	1.0	0.158	0.273
MEMO	1.0	0.111	0.200	1.0	0.111	0.200
WExT	1.0	0.111	0.200	1.0	0.111	0.200

(f) Metrics for SKCM data. (468 samples | 458 CGC-CGC pairs)

Method	Precision	Sensitivity	F1 Score	$\text{Precision}_{\text{strict}}$	$\text{Sensitivity}_{\text{strict}}$	F1 $_{\text{strict}}$ Score
DISCOVER	0.800	0.045	0.084	0.800	0.045	0.085
Fisher's Exact Test	1.000	0.004	0.009	1.000	0.004	0.008
MEGSA	0.889	0.017	0.034	0.889	0.017	0.033
WExT	0.708	0.117	0.201	0.721	0.113	0.195

(g) Metrics for STAD data. (438 samples | 140 CGC-CGC pairs)

Method	Precision	Sensitivity	F1 Score	$\text{Precision}_{\text{strict}}$	$\text{Sensitivity}_{\text{strict}}$	F1 $_{\text{strict}}$ Score
DISCOVER	0.682	0.112	0.192	0.667	0.090	0.159
Fisher's Exact Test	0.000	0.000	NaN	0.000	0.000	NaN
MEGSA	0.667	0.014	0.028	0.667	0.014	0.027
WExT	0.634	0.191	0.294	0.636	0.154	0.248

(h) Metrics for UCEC data. (531 samples | 1356 CGC-CGC pairs)

Method	Precision	Sensitivity	F1 Score	$\text{Precision}_{\text{strict}}$	$\text{Sensitivity}_{\text{strict}}$	F1 $_{\text{strict}}$ Score
DISCOVER	0.652	0.178	0.279	0.712	0.143	0.238
Fisher's Exact Test	0.833	0.007	0.015	0.833	0.007	0.014
MEGSA	0.786	0.008	0.016	0.786	0.008	0.016
WExT	0.615	0.277	0.382	0.663	0.226	0.337

Table 3: Results of network-centric ME evaluation framework with $\mathcal{G}$ = Intact (w conf. threshold 0.35), $\mathcal{S}$ = CGC, c= X_1, p_t = 0.05, t=20, robustness_iterations = 500

(a) Metrics for BLCA data. (411 samples | 56 CGC-CGC pairs)

Method	Precision	Sensitivity	F1 Score	$\text{Precision}_{\text{strict}}$	$\text{Sensitivity}_{\text{strict}}$	F1 $_{\text{strict}}$ Score
DISCOVER	0.800	0.075	0.138	0.800	0.075	0.137
Fisher's Exact Test	1.000	0.037	0.071	1.000	0.037	0.071
MEGSA	1.000	0.073	0.136	1.000	0.073	0.136
MEMO	0.667	0.078	0.140	0.667	0.078	0.140
WExT	0.571	0.075	0.133	0.571	0.075	0.133

(b) Metrics for BRCA data. (1026 samples | 34 CGC-CGC pairs)

Method	Precision	Sensitivity	F1 Score	$\text{Precision}_{\text{strict}}$	$\text{Sensitivity}_{\text{strict}}$	F1 $_{\text{strict}}$ Score
DISCOVER	0.652	0.469	0.545	0.632	0.375	0.471
DISCOVER Strat	0.711	0.478	0.571	0.769	0.448	0.566
Fisher's Exact Test	1.000	0.061	0.114	1.000	0.061	0.115
MEGSA	1.000	0.059	0.111	1.000	0.059	0.111
MEMO	0.644	0.475	0.547	0.622	0.377	0.469
WExT	0.625	0.536	0.577	0.684	0.464	0.553

(c) Metrics for COADREAD data. (498 samples | 196 CGC-CGC pairs)

Method	Precision	Sensitivity	F1 Score	$\text{Precision}_{\text{strict}}$	$\text{Sensitivity}_{\text{strict}}$	F1 $_{\text{strict}}$ Score
DISCOVER	0.662	0.224	0.335	0.706	0.188	0.297
DISCOVER Strat	0.727	0.042	0.080	0.727	0.042	0.079
Fisher's Exact Test	0.538	0.036	0.067	0.538	0.036	0.067
MEGSA	0.611	0.056	0.103	0.588	0.051	0.094
MEMO	0.660	0.339	0.448	0.657	0.243	0.355
WExT	0.691	0.412	0.516	0.744	0.342	0.469

(d) Metrics for LUAD data. (568 samples | 92 CGC-CGC pairs)

Method	Precision	Sensitivity	F1 Score	$\text{Precision}_{\text{strict}}$	$\text{Sensitivity}_{\text{strict}}$	F1 $_{\text{strict}}$ Score
DISCOVER	0.714	0.114	0.197	0.727	0.091	0.162
Fisher's Exact Test	0.000	0.000	NaN	0.000	0.000	NaN
MEGSA	0.667	0.022	0.043	0.667	0.022	0.043
MEMO	0.722	0.149	0.248	0.733	0.126	0.215
WExT	0.640	0.179	0.279	0.667	0.156	0.253

(e) Metrics for LUSC data. (485 samples | 38 CGC-CGC pairs)

Method	Precision	Sensitivity	F1 Score	$\text{Precision}_{\text{strict}}$	$\text{Sensitivity}_{\text{strict}}$	F1 $_{\text{strict}}$ Score
DISCOVER	1.0	0.054	0.103	1.0	0.054	0.102
Fisher's Exact Test	1.0	0.053	0.100	1.0	0.053	0.101
MEGSA	1.0	0.158	0.273	1.0	0.158	0.273
MEMO	1.0	0.111	0.200	1.0	0.111	0.200
WExT	1.0	0.111	0.200	1.0	0.111	0.200

(f) Metrics for SKCM data. (468 samples | 458 CGC-CGC pairs)

Method	Precision	Sensitivity	F1 Score	$\text{Precision}_{\text{strict}}$	$\text{Sensitivity}_{\text{strict}}$	F1 $_{\text{strict}}$ Score
DISCOVER	0.833	0.044	0.084	0.833	0.044	0.084
Fisher's Exact Test	1.000	0.004	0.009	1.000	0.004	0.008
MEGSA	0.889	0.018	0.034	0.889	0.018	0.035
WExT	0.697	0.121	0.206	0.708	0.116	0.199

(g) Metrics for STAD data. (438 samples | 140 CGC-CGC pairs)

Method	Precision	Sensitivity	F1 Score	$\text{Precision}_{\text{strict}}$	$\text{Sensitivity}_{\text{strict}}$	F1 $_{\text{strict}}$ Score
DISCOVER	0.667	0.119	0.201	0.684	0.096	0.168
Fisher's Exact Test	0.000	0.000	NaN	0.000	0.000	NaN
MEGSA	0.667	0.014	0.028	0.667	0.014	0.027
WExT	0.634	0.191	0.294	0.636	0.154	0.248

(h) Metrics for UCEC data. (531 samples | 1356 CGC-CGC pairs)

Method	Precision	Sensitivity	F1 Score	$\text{Precision}_{\text{strict}}$	$\text{Sensitivity}_{\text{strict}}$	F1 $_{\text{strict}}$ Score
DISCOVER	0.655	0.177	0.278	0.716	0.143	0.238
Fisher's Exact Test	0.833	0.007	0.015	0.833	0.007	0.014
MEGSA	0.786	0.008	0.016	0.786	0.008	0.016
WExT	0.617	0.277	0.383	0.669	0.227	0.339

Table 4: Results of network-centric ME evaluation framework with $\mathcal{G}$ = Intact (w conf. threshold 0.35), $\mathcal{S}$ = CGC, c= X_1, p_t = 0.01, t=20, robustness_iterations = 100

(a) Metrics for BLCA data. (411 samples | 56 CGC-CGC pairs)

Method	Precision	Sensitivity	F1 Score	$\text{Precision}_{\text{strict}}$	$\text{Sensitivity}_{\text{strict}}$	F1 $_{\text{strict}}$ Score
DISCOVER	1.000	0.036	0.07	1.000	0.036	0.069
Fisher's Exact Test	NaN	0.000	NaN	NaN	0.000	NaN
MEGSA	NaN	0.000	NaN	NaN	0.000	NaN
MEMO	0.667	0.037	0.07	0.667	0.037	0.070
WExT	0.667	0.037	0.07	0.667	0.037	0.070

(b) Metrics for BRCA data. (1026 samples | 34 CGC-CGC pairs)

Method	Precision	Sensitivity	F1 Score	$\text{Precision}_{\text{strict}}$	$\text{Sensitivity}_{\text{strict}}$	F1 $_{\text{strict}}$ Score
DISCOVER	0.714	0.156	0.256	0.714	0.156	0.256
DISCOVER Strat	0.857	0.176	0.293	0.857	0.176	0.292
Fisher's Exact Test	NaN	0.000	NaN	NaN	0.000	NaN
MEGSA	NaN	0.000	NaN	NaN	0.000	NaN
MEMO	0.625	0.147	0.238	0.625	0.147	0.238
WExT	0.556	0.152	0.238	0.556	0.152	0.239

(c) Metrics for COADREAD data. (498 samples | 196 CGC-CGC pairs)

Method	Precision	Sensitivity	F1 Score	$\text{Precision}_{\text{strict}}$	$\text{Sensitivity}_{\text{strict}}$	F1 $_{\text{strict}}$ Score
DISCOVER	0.686	0.125	0.211	0.690	0.104	0.181
DISCOVER Strat	0.000	0.000	NaN	0.000	0.000	NaN
Fisher's Exact Test	0.000	0.000	NaN	0.000	0.000	NaN
MEGSA	0.400	0.010	0.020	0.400	0.010	0.020
MEMO	0.689	0.265	0.383	0.655	0.187	0.291
WExT	0.679	0.280	0.396	0.724	0.222	0.340

(d) Metrics for LUAD data. (568 samples | 92 CGC-CGC pairs)

Method	Precision	Sensitivity	F1 Score	$\text{Precision}_{\text{strict}}$	$\text{Sensitivity}_{\text{strict}}$	F1 $_{\text{strict}}$ Score
DISCOVER	0.667	0.044	0.082	0.667	0.044	0.083
Fisher's Exact Test	NaN	0.000	NaN	NaN	0.000	NaN
MEGSA	0.667	0.022	0.042	0.667	0.022	0.043
MEMO	0.700	0.078	0.141	0.667	0.067	0.122
WExT	0.750	0.105	0.184	0.700	0.081	0.145

(e) Metrics for LUSC data. (485 samples | 38 CGC-CGC pairs)

Method	Precision	Sensitivity	F1 Score	$\text{Precision}_{\text{strict}}$	$\text{Sensitivity}_{\text{strict}}$	F1 $_{\text{strict}}$ Score
DISCOVER	1.0	0.053	0.100	1.0	0.053	0.101
Fisher's Exact Test	NaN	0.000	NaN	NaN	0.000	NaN
MEGSA	NaN	0.000	NaN	NaN	0.000	NaN
MEMO	1.0	0.056	0.105	1.0	0.056	0.106
WExT	1.0	0.053	0.100	1.0	0.053	0.101

(f) Metrics for SKCM data. (468 samples | 458 CGC-CGC pairs)

Method	Precision	Sensitivity	F1 Score	$\text{Precision}_{\text{strict}}$	$\text{Sensitivity}_{\text{strict}}$	F1 $_{\text{strict}}$ Score
DISCOVER	1.000	0.013	0.026	1.00	0.013	0.026
Fisher's Exact Test	1.000	0.004	0.009	1.00	0.004	0.008
MEGSA	1.000	0.004	0.009	1.00	0.004	0.008
WExT	0.731	0.042	0.080	0.75	0.040	0.076

(g) Metrics for STAD data. (438 samples | 140 CGC-CGC pairs)

Method	Precision	Sensitivity	F1 Score	$\text{Precision}_{\text{strict}}$	$\text{Sensitivity}_{\text{strict}}$	F1 $_{\text{strict}}$ Score
DISCOVER	0.667	0.059	0.108	0.70	0.051	0.095
Fisher's Exact Test	NaN	0.000	NaN	NaN	0.000	NaN
MEGSA	NaN	0.000	NaN	NaN	0.000	NaN
WExT	0.622	0.084	0.148	0.63	0.062	0.113

(h) Metrics for UCEC data. (531 samples | 1356 CGC-CGC pairs)

Method	Precision	Sensitivity	F1 Score	$\text{Precision}_{\text{strict}}$	$\text{Sensitivity}_{\text{strict}}$	F1 $_{\text{strict}}$ Score
DISCOVER	0.654	0.120	0.203	0.718	0.100	0.176
Fisher's Exact Test	0.857	0.004	0.009	0.857	0.004	0.008
MEGSA	0.857	0.004	0.009	0.857	0.004	0.008
WExT	0.636	0.199	0.303	0.699	0.158	0.258

Table 5: Results of network-centric ME evaluation framework with $\mathcal{G}$ = Intact (w conf. threshold 0.35), $\mathcal{S}$ = CGC, c= X_1, p_t = 0.1, t=20, robustness_iterations = 100

(a) Metrics for BLCA data. (411 samples | 56 CGC-CGC pairs)

Method	Precision	Sensitivity	F1 Score	$\text{Precision}_{\text{strict}}$	$\text{Sensitivity}_{\text{strict}}$	F1 $_{\text{strict}}$ Score
DISCOVER	0.75	0.111	0.194	0.750	0.111	0.193
Fisher's Exact Test	1.00	0.036	0.070	1.000	0.036	0.069
MEGSA	0.80	0.074	0.136	0.800	0.074	0.135
MEMO	0.60	0.113	0.190	0.600	0.113	0.190
WExT	0.60	0.117	0.195	0.667	0.117	0.199

(b) Metrics for BRCA data. (1026 samples | 34 CGC-CGC pairs)

Method	Precision	Sensitivity	F1 Score	$\text{Precision}_{\text{strict}}$	$\text{Sensitivity}_{\text{strict}}$	F1 $_{\text{strict}}$ Score
DISCOVER	0.660	0.600	0.629	0.692	0.491	0.574
DISCOVER Strat	0.667	0.600	0.632	0.696	0.533	0.604
Fisher's Exact Test	1.000	0.062	0.118	1.000	0.062	0.117
MEGSA	0.667	0.059	0.108	0.667	0.059	0.108
MEMO	0.635	0.600	0.617	0.667	0.473	0.553
WExT	0.638	0.698	0.667	0.707	0.547	0.617

(c) Metrics for COADREAD data. (498 samples | 196 CGC-CGC pairs)

Method	Precision	Sensitivity	F1 Score	$\text{Precision}_{\text{strict}}$	$\text{Sensitivity}_{\text{strict}}$	F1 $_{\text{strict}}$ Score
DISCOVER	0.649	0.291	0.401	0.702	0.245	0.363
DISCOVER Strat	0.738	0.127	0.217	0.737	0.111	0.193
Fisher's Exact Test	0.708	0.087	0.156	0.667	0.072	0.130
MEGSA	0.714	0.103	0.179	0.688	0.085	0.151
MEMO	0.670	0.423	0.519	0.678	0.324	0.438
WExT	0.640	0.421	0.508	0.703	0.347	0.465

(d) Metrics for LUAD data. (568 samples | 92 CGC-CGC pairs)

Method	Precision	Sensitivity	F1 Score	$\text{Precision}_{\text{strict}}$	$\text{Sensitivity}_{\text{strict}}$	F1 $_{\text{strict}}$ Score
DISCOVER	0.698	0.173	0.278	0.703	0.150	0.247
Fisher's Exact Test	0.667	0.022	0.043	0.667	0.022	0.043
MEGSA	0.667	0.066	0.120	0.667	0.066	0.120
MEMO	0.645	0.220	0.328	0.654	0.187	0.291
WExT	0.659	0.326	0.436	0.667	0.270	0.384

(e) Metrics for LUSC data. (485 samples | 38 CGC-CGC pairs)

Method	Precision	Sensitivity	F1 Score	$\text{Precision}_{\text{strict}}$	$\text{Sensitivity}_{\text{strict}}$	F1 $_{\text{strict}}$ Score
DISCOVER	1.000	0.111	0.200	1.000	0.111	0.200
Fisher's Exact Test	1.000	0.056	0.105	1.000	0.056	0.106
MEGSA	0.857	0.154	0.261	0.857	0.154	0.261
MEMO	0.800	0.118	0.205	0.800	0.118	0.206
WExT	0.750	0.176	0.286	0.750	0.176	0.285

(f) Metrics for SKCM data. (468 samples | 458 CGC-CGC pairs)

Method	Precision	Sensitivity	F1 Score	$\text{Precision}_{\text{strict}}$	$\text{Sensitivity}_{\text{strict}}$	F1 $_{\text{strict}}$ Score
DISCOVER	0.740	0.105	0.184	0.756	0.101	0.178
Fisher's Exact Test	1.000	0.004	0.009	1.000	0.004	0.008
MEGSA	0.800	0.026	0.051	0.800	0.026	0.050
WExT	0.622	0.170	0.267	0.634	0.163	0.259

(g) Metrics for STAD data. (438 samples | 140 CGC-CGC pairs)

Method	Precision	Sensitivity	F1 Score	$\text{Precision}_{\text{strict}}$	$\text{Sensitivity}_{\text{strict}}$	F1 $_{\text{strict}}$ Score
DISCOVER	0.641	0.183	0.285	0.625	0.147	0.238
Fisher's Exact Test	0.667	0.014	0.028	0.667	0.014	0.027
MEGSA	0.500	0.014	0.028	0.500	0.014	0.027
WExT	0.632	0.224	0.331	0.623	0.179	0.278

(h) Metrics for UCEC data. (531 samples | 1356 CGC-CGC pairs)

Method	Precision	Sensitivity	F1 Score	$\text{Precision}_{\text{strict}}$	$\text{Sensitivity}_{\text{strict}}$	F1 $_{\text{strict}}$ Score
DISCOVER	0.621	0.219	0.324	0.672	0.176	0.279
Fisher's Exact Test	0.750	0.013	0.026	0.773	0.013	0.026
MEGSA	0.806	0.018	0.036	0.821	0.017	0.033
WExT	0.594	0.344	0.436	0.640	0.279	0.389

Table 6: Results of network-centric ME evaluation framework with $\mathcal{G}$ = Intact (w conf. threshold 0.35), $\mathcal{S} = CGC_{SNV}$, c= X_1, p_t = 0.05, t=20, robustness_iterations = 100

(a) Metrics for BLCA data. (411 samples | 36 CGC_{SNV}-CGC_{SNV} pairs)

Method	Precision	Sensitivity	F1 Score	$\text{Precision}_{\text{strict}}$	$\text{Sensitivity}_{\text{strict}}$	F1 $_{\text{strict}}$ Score
DISCOVER	0.8	0.118	0.205	0.8	0.118	0.206
Fisher's Exact Test	1.0	0.056	0.105	1.0	0.056	0.106
MEGSA	1.0	0.113	0.203	1.0	0.113	0.203
MEMO	0.8	0.121	0.211	0.8	0.121	0.210
WExT	0.8	0.129	0.222	0.8	0.129	0.222

(b) Metrics for BRCA data. (1026 samples | 28 CGC_{SNV}-CGC_{SNV} pairs)

Method	Precision	Sensitivity	F1 Score	$\text{Precision}_{\text{strict}}$	$\text{Sensitivity}_{\text{strict}}$	F1 $_{\text{strict}}$ Score
DISCOVER	0.636	0.519	0.571	0.647	0.407	0.500
DISCOVER Strat	0.737	0.560	0.636	0.812	0.520	0.634
Fisher's Exact Test	1.000	0.073	0.136	1.000	0.073	0.136
MEGSA	1.000	0.071	0.133	1.000	0.071	0.133
MEMO	0.667	0.480	0.558	0.667	0.400	0.500
WExT	0.625	0.556	0.588	0.645	0.444	0.526

(c) Metrics for COADREAD data. (498 samples | 146 CGC_{SNV}-CGC_{SNV} pairs)

Method	Precision	Sensitivity	F1 Score	$\text{Precision}_{\text{strict}}$	$\text{Sensitivity}_{\text{strict}}$	F1 $_{\text{strict}}$ Score
DISCOVER	0.635	0.237	0.345	0.675	0.194	0.301
DISCOVER Strat	0.750	0.042	0.079	0.750	0.042	0.080
Fisher's Exact Test	0.600	0.041	0.077	0.600	0.041	0.077
MEGSA	0.667	0.068	0.123	0.643	0.061	0.111
MEMO	0.634	0.330	0.434	0.627	0.234	0.341
WExT	0.656	0.380	0.482	0.706	0.304	0.425

(d) Metrics for LUAD data. (568 samples | 70 CGC_{SNV}-CGC_{SNV} pairs)

Method	Precision	Sensitivity	F1 Score	$\text{Precision}_{\text{strict}}$	$\text{Sensitivity}_{\text{strict}}$	F1 $_{\text{strict}}$ Score
DISCOVER	0.727	0.121	0.208	0.667	0.091	0.160
Fisher's Exact Test	0.000	0.000	NaN	0.000	0.000	NaN
MEGSA	0.500	0.029	0.055	0.500	0.029	0.055
MEMO	0.686	0.185	0.291	0.690	0.154	0.252
WExT	0.609	0.211	0.313	0.600	0.180	0.277

(e) Metrics for LUSC data. (485 samples | 30 CGC_{SNV}-CGC_{SNV} pairs)

Method	Precision	Sensitivity	F1 Score	$\text{Precision}_{\text{strict}}$	$\text{Sensitivity}_{\text{strict}}$	F1 $_{\text{strict}}$ Score
DISCOVER	1.0	0.067	0.125	1.0	0.067	0.126
Fisher's Exact Test	1.0	0.067	0.125	1.0	0.067	0.126
MEGSA	1.0	0.200	0.333	1.0	0.200	0.333
MEMO	1.0	0.138	0.242	1.0	0.138	0.243
WExT	1.0	0.140	0.246	1.0	0.140	0.246

(f) Metrics for SKCM data. (468 samples | 288 CGC_{SNV}-CGC_{SNV} pairs)

Method	Precision	Sensitivity	F1 Score	$\text{Precision}_{\text{strict}}$	$\text{Sensitivity}_{\text{strict}}$	F1 $_{\text{strict}}$ Score
DISCOVER	0.714	0.036	0.068	0.714	0.036	0.069
Fisher's Exact Test	1.000	0.007	0.014	1.000	0.007	0.014
MEGSA	0.800	0.014	0.027	0.800	0.014	0.028
WExT	0.651	0.131	0.218	0.660	0.122	0.206

(g) Metrics for STAD data. (438 samples | 112 CGC_{SNV}-CGC_{SNV} pairs)

Method	Precision	Sensitivity	F1 Score	$\text{Precision}_{\text{strict}}$	$\text{Sensitivity}_{\text{strict}}$	F1 $_{\text{strict}}$ Score
DISCOVER	0.600	0.111	0.187	0.562	0.083	0.145
Fisher's Exact Test	0.000	0.000	NaN	0.000	0.000	NaN
MEGSA	0.667	0.018	0.034	0.667	0.018	0.035
WExT	0.609	0.182	0.281	0.588	0.140	0.226

(h) Metrics for UCEC data. (531 samples | 776 CGC_{SNV}-CGC_{SNV} pairs)

Method	Precision	Sensitivity	F1 Score	$\text{Precision}_{\text{strict}}$	$\text{Sensitivity}_{\text{strict}}$	F1 $_{\text{strict}}$ Score
DISCOVER	0.637	0.210	0.316	0.681	0.166	0.267
Fisher's Exact Test	0.833	0.013	0.025	0.833	0.013	0.026
MEGSA	0.846	0.014	0.028	0.846	0.014	0.028
WExT	0.599	0.318	0.415	0.630	0.250	0.358

Table 7: Results of network-centric ME evaluation framework with $\mathcal{G}$ = Intact (w conf. threshold 0.35), $\mathcal{S}$ = Intogen , c= X_1, p_t = 0.05, t=20, robustness_iterations = 100

(a) Metrics for BLCA data. (411 samples | 56 Intogen-Intogen pairs)

Method	Precision	Sensitivity	F1 Score	$\text{Precision}_{\text{strict}}$	$\text{Sensitivity}_{\text{strict}}$	F1 $_{\text{strict}}$ Score
DISCOVER	0.889	0.145	0.250	0.889	0.145	0.249
Fisher's Exact Test	1.000	0.036	0.070	1.000	0.036	0.069
MEGSA	1.000	0.109	0.197	1.000	0.109	0.197
MEMO	0.800	0.150	0.252	0.800	0.150	0.253
WExT	0.727	0.154	0.254	0.727	0.154	0.254

(b) Metrics for BRCA data. (1026 samples | 36 Intogen-Intogen pairs)

Method	Precision	Sensitivity	F1 Score	$\text{Precision}_{\text{strict}}$	$\text{Sensitivity}_{\text{strict}}$	F1 $_{\text{strict}}$ Score
DISCOVER	0.654	0.500	0.567	0.619	0.382	0.472
DISCOVER Strat	0.700	0.444	0.544	0.722	0.413	0.525
Fisher's Exact Test	1.000	0.057	0.108	1.000	0.057	0.108
MEGSA	1.000	0.056	0.105	1.000	0.056	0.106
MEMO	0.667	0.500	0.571	0.650	0.406	0.500
WExT	0.654	0.567	0.607	0.667	0.467	0.549

(c) Metrics for COADREAD data. (498 samples | 206 Intogen-Intogen pairs)

Method	Precision	Sensitivity	F1 Score	$\text{Precision}_{\text{strict}}$	$\text{Sensitivity}_{\text{strict}}$	F1 $_{\text{strict}}$ Score
DISCOVER	0.657	0.224	0.335	0.685	0.189	0.296
DISCOVER Strat	0.679	0.048	0.090	0.679	0.048	0.090
Fisher's Exact Test	0.538	0.034	0.065	0.538	0.034	0.064
MEGSA	0.611	0.054	0.099	0.588	0.049	0.090
MEMO	0.652	0.339	0.446	0.638	0.245	0.354
WExT	0.678	0.416	0.515	0.722	0.338	0.460

(d) Metrics for LUAD data. (568 samples | 96 Intogen-Intogen pairs)

Method	Precision	Sensitivity	F1 Score	$\text{Precision}_{\text{strict}}$	$\text{Sensitivity}_{\text{strict}}$	F1 $_{\text{strict}}$ Score
DISCOVER	0.800	0.091	0.163	0.778	0.080	0.145
Fisher's Exact Test	0.000	0.000	NaN	0.000	0.000	NaN
MEGSA	0.667	0.021	0.041	0.667	0.021	0.041
MEMO	0.765	0.152	0.254	0.786	0.129	0.222
WExT	0.667	0.184	0.288	0.700	0.161	0.262

(e) Metrics for LUSC data. (485 samples | 50 Intogen-Intogen pairs)

Method	Precision	Sensitivity	F1 Score	$\text{Precision}_{\text{strict}}$	$\text{Sensitivity}_{\text{strict}}$	F1 $_{\text{strict}}$ Score
DISCOVER	1.000	0.081	0.150	1.000	0.081	0.150
Fisher's Exact Test	1.000	0.040	0.077	1.000	0.040	0.077
MEGSA	1.000	0.167	0.286	1.000	0.167	0.286
MEMO	1.000	0.128	0.226	1.000	0.128	0.227
WExT	0.857	0.129	0.224	0.857	0.129	0.224

(f) Metrics for SKCM data. (468 samples | 464 Intogen-Intogen pairs)

Method	Precision	Sensitivity	F1 Score	$\text{Precision}_{\text{strict}}$	$\text{Sensitivity}_{\text{strict}}$	F1 $_{\text{strict}}$ Score
DISCOVER	0.750	0.040	0.075	0.783	0.040	0.076
Fisher's Exact Test	1.000	0.004	0.009	1.000	0.004	0.008
MEGSA	0.857	0.013	0.026	0.857	0.013	0.026
WExT	0.671	0.115	0.197	0.681	0.111	0.191

(g) Metrics for STAD data. (438 samples | 162 Intogen-Intogen pairs)

Method	Precision	Sensitivity	F1 Score	$\text{Precision}_{\text{strict}}$	$\text{Sensitivity}_{\text{strict}}$	F1 $_{\text{strict}}$ Score
DISCOVER	0.625	0.095	0.164	0.600	0.076	0.135
Fisher's Exact Test	0.000	0.000	NaN	0.000	0.000	NaN
MEGSA	0.667	0.012	0.024	0.667	0.012	0.024
WExT	0.673	0.214	0.325	0.650	0.169	0.268

(h) Metrics for UCEC data. (531 samples | 1240 Intogen-Intogen pairs)

Method	Precision	Sensitivity	F1 Score	$\text{Precision}_{\text{strict}}$	$\text{Sensitivity}_{\text{strict}}$	F1 $_{\text{strict}}$ Score
DISCOVER	0.636	0.210	0.315	0.687	0.167	0.269
Fisher's Exact Test	0.778	0.011	0.022	0.765	0.011	0.022
MEGSA	0.727	0.013	0.025	0.750	0.012	0.024
WExT	0.586	0.309	0.405	0.621	0.245	0.351

Table 8: Results of network-centric ME evaluation framework with $\mathcal{G}$ = Intact (w conf. threshold 0.25), $\mathcal{S}$ = CGC, c= X_1, p_t = 0.05, t=20, robustness_iterations = 100

(a) Metrics for BLCA data. (411 samples | 92 CGC-CGC pairs)

Method	Precision	Sensitivity	F1 Score	$\text{Precision}_{\text{strict}}$	$\text{Sensitivity}_{\text{strict}}$	F1 $_{\text{strict}}$ Score
DISCOVER	0.833	0.114	0.200	0.833	0.114	0.201
Fisher's Exact Test	1.000	0.066	0.124	1.000	0.066	0.124
MEGSA	1.000	0.109	0.197	1.000	0.109	0.197
MEMO	0.750	0.103	0.182	0.750	0.103	0.181
WExT	0.737	0.163	0.267	0.778	0.163	0.270

(b) Metrics for BRCA data. (1026 samples | 54 CGC-CGC pairs)

Method	Precision	Sensitivity	F1 Score	$\text{Precision}_{\text{strict}}$	$\text{Sensitivity}_{\text{strict}}$	F1 $_{\text{strict}}$ Score
DISCOVER	0.712	0.485	0.577	0.702	0.412	0.519
DISCOVER Strat	0.782	0.473	0.589	0.796	0.429	0.558
Fisher's Exact Test	0.667	0.038	0.071	0.667	0.038	0.072
MEGSA	0.667	0.037	0.070	0.667	0.037	0.070
MEMO	0.706	0.485	0.575	0.700	0.424	0.528
WExT	0.630	0.580	0.604	0.639	0.460	0.535

(c) Metrics for COADREAD data. (498 samples | 264 CGC-CGC pairs)

Method	Precision	Sensitivity	F1 Score	$\text{Precision}_{\text{strict}}$	$\text{Sensitivity}_{\text{strict}}$	F1 $_{\text{strict}}$ Score
DISCOVER	0.659	0.216	0.325	0.713	0.181	0.289
DISCOVER Strat	0.667	0.054	0.100	0.684	0.050	0.093
Fisher's Exact Test	0.583	0.053	0.097	0.591	0.049	0.090
MEGSA	0.667	0.072	0.131	0.667	0.065	0.118
MEMO	0.648	0.318	0.427	0.615	0.220	0.324
WExT	0.681	0.372	0.481	0.731	0.301	0.426

(d) Metrics for LUAD data. (568 samples | 138 CGC-CGC pairs)

Method	Precision	Sensitivity	F1 Score	$\text{Precision}_{\text{strict}}$	$\text{Sensitivity}_{\text{strict}}$	F1 $_{\text{strict}}$ Score
DISCOVER	0.755	0.153	0.254	0.800	0.137	0.234
Fisher's Exact Test	0.800	0.029	0.056	0.800	0.029	0.056
MEGSA	0.727	0.058	0.107	0.727	0.058	0.107
MEMO	0.735	0.191	0.303	0.767	0.176	0.286
WExT	0.686	0.225	0.339	0.753	0.210	0.328

(e) Metrics for LUSC data. (485 samples | 70 CGC-CGC pairs)

Method	Precision	Sensitivity	F1 Score	$\text{Precision}_{\text{strict}}$	$\text{Sensitivity}_{\text{strict}}$	F1 $_{\text{strict}}$ Score
DISCOVER	1.000	0.029	0.056	1.000	0.029	0.056
Fisher's Exact Test	1.000	0.029	0.056	1.000	0.029	0.056
MEGSA	0.857	0.087	0.158	0.857	0.087	0.158
MEMO	0.800	0.059	0.110	0.800	0.059	0.110
WExT	0.571	0.058	0.106	0.571	0.058	0.105

(f) Metrics for SKCM data. (468 samples | 640 CGC-CGC pairs)

Method	Precision	Sensitivity	F1 Score	$\text{Precision}_{\text{strict}}$	$\text{Sensitivity}_{\text{strict}}$	F1 $_{\text{strict}}$ Score
DISCOVER	0.845	0.048	0.091	0.870	0.048	0.091
Fisher's Exact Test	1.000	0.003	0.006	1.000	0.003	0.006
MEGSA	0.889	0.013	0.025	0.889	0.013	0.026
WExT	0.714	0.110	0.190	0.721	0.105	0.183

(g) Metrics for STAD data. (438 samples | 194 CGC-CGC pairs)

Method	Precision	Sensitivity	F1 Score	$\text{Precision}_{\text{strict}}$	$\text{Sensitivity}_{\text{strict}}$	F1 $_{\text{strict}}$ Score
DISCOVER	0.675	0.142	0.235	0.710	0.116	0.199
Fisher's Exact Test	0.667	0.010	0.020	0.667	0.010	0.020
MEGSA	0.667	0.021	0.040	0.667	0.021	0.041
WExT	0.661	0.201	0.308	0.698	0.163	0.264

(h) Metrics for UCEC data. (531 samples | 1916 CGC-CGC pairs)

Method	Precision	Sensitivity	F1 Score	$\text{Precision}_{\text{strict}}$	$\text{Sensitivity}_{\text{strict}}$	F1 $_{\text{strict}}$ Score
DISCOVER	0.652	0.163	0.261	0.708	0.132	0.223
Fisher's Exact Test	0.771	0.007	0.014	0.771	0.007	0.014
MEGSA	0.773	0.009	0.018	0.773	0.009	0.018
WExT	0.610	0.267	0.371	0.656	0.220	0.329

Table 9: Results of network-centric ME evaluation framework with $\mathcal{G}$ = Intact (w conf. threshold 0.45), $\mathcal{S}$ = CGC, c= X_1, p_t = 0.05, t=20, robustness_iterations = 100

(a) Metrics for BLCA data. (411 samples | 34 CGC-CGC pairs)

Method	Precision	Sensitivity	F1 Score	$\text{Precision}_{\text{strict}}$	$\text{Sensitivity}_{\text{strict}}$	F1 $_{\text{strict}}$ Score
DISCOVER	1.0	0.127	0.225	1.0	0.127	0.225
Fisher's Exact Test	1.0	0.060	0.113	1.0	0.060	0.113
MEGSA	1.0	0.125	0.222	1.0	0.125	0.222
MEMO	0.8	0.129	0.222	0.8	0.129	0.222
WExT	0.8	0.127	0.219	0.8	0.127	0.219

(b) Metrics for BRCA data. (1026 samples | 18 CGC-CGC pairs)

Method	Precision	Sensitivity	F1 Score	$\text{Precision}_{\text{strict}}$	$\text{Sensitivity}_{\text{strict}}$	F1 $_{\text{strict}}$ Score
DISCOVER	0.667	0.457	0.542	0.700	0.400	0.509
DISCOVER Strat	0.778	0.438	0.560	0.778	0.438	0.560
Fisher's Exact Test	1.000	0.125	0.222	1.000	0.125	0.222
MEGSA	1.000	0.114	0.205	1.000	0.114	0.205
MEMO	0.636	0.467	0.538	0.667	0.400	0.500
WExT	0.583	0.467	0.519	0.667	0.400	0.500

(c) Metrics for COADREAD data. (498 samples | 100 CGC-CGC pairs)

Method	Precision	Sensitivity	F1 Score	$\text{Precision}_{\text{strict}}$	$\text{Sensitivity}_{\text{strict}}$	F1 $_{\text{strict}}$ Score
DISCOVER	0.731	0.261	0.384	0.837	0.218	0.346
DISCOVER Strat	0.750	0.062	0.114	0.750	0.062	0.115
Fisher's Exact Test	0.600	0.030	0.058	0.600	0.030	0.057
MEGSA	0.727	0.080	0.144	0.700	0.070	0.127
MEMO	0.700	0.311	0.431	0.724	0.233	0.353
WExT	0.660	0.368	0.473	0.732	0.316	0.441

(d) Metrics for LUAD data. (568 samples | 46 CGC-CGC pairs)

Method	Precision	Sensitivity	F1 Score	$\text{Precision}_{\text{strict}}$	$\text{Sensitivity}_{\text{strict}}$	F1 $_{\text{strict}}$ Score
DISCOVER	0.800	0.089	0.160	0.750	0.067	0.123
Fisher's Exact Test	NaN	0.000	NaN	NaN	0.000	NaN
MEGSA	1.000	0.044	0.085	1.000	0.044	0.084
MEMO	0.800	0.143	0.242	0.769	0.119	0.206
WExT	0.727	0.184	0.294	0.778	0.161	0.267

(e) Metrics for LUSC data. (485 samples | 22 CGC-CGC pairs)

Method	Precision	Sensitivity	F1 Score	$\text{Precision}_{\text{strict}}$	$\text{Sensitivity}_{\text{strict}}$	F1 $_{\text{strict}}$ Score
DISCOVER	1.0	0.095	0.174	1.0	0.095	0.174
Fisher's Exact Test	1.0	0.091	0.167	1.0	0.091	0.167
MEGSA	1.0	0.273	0.429	1.0	0.273	0.429
MEMO	1.0	0.195	0.327	1.0	0.195	0.326
WExT	1.0	0.182	0.308	1.0	0.182	0.308

(f) Metrics for SKCM data. (468 samples | 194 CGC-CGC pairs)

Method	Precision	Sensitivity	F1 Score	$\text{Precision}_{\text{strict}}$	$\text{Sensitivity}_{\text{strict}}$	F1 $_{\text{strict}}$ Score
DISCOVER	0.769	0.052	0.098	0.769	0.052	0.097
Fisher's Exact Test	1.000	0.010	0.020	1.000	0.010	0.020
MEGSA	1.000	0.021	0.041	1.000	0.021	0.041
WExT	0.757	0.151	0.252	0.794	0.146	0.247

(g) Metrics for STAD data. (438 samples | 72 CGC-CGC pairs)

Method	Precision	Sensitivity	F1 Score	$\text{Precision}_{\text{strict}}$	$\text{Sensitivity}_{\text{strict}}$	F1 $_{\text{strict}}$ Score
DISCOVER	0.556	0.072	0.127	0.571	0.058	0.105
Fisher's Exact Test	NaN	0.000	NaN	NaN	0.000	NaN
MEGSA	1.000	0.028	0.055	1.000	0.028	0.054
WExT	0.533	0.113	0.186	0.583	0.099	0.169

(h) Metrics for UCEC data. (531 samples | 606 CGC-CGC pairs)

Method	Precision	Sensitivity	F1 Score	$\text{Precision}_{\text{strict}}$	$\text{Sensitivity}_{\text{strict}}$	F1 $_{\text{strict}}$ Score
DISCOVER	0.666	0.218	0.329	0.729	0.171	0.277
Fisher's Exact Test	0.800	0.013	0.026	0.800	0.013	0.026
MEGSA	0.818	0.015	0.029	0.818	0.015	0.029
WExT	0.624	0.306	0.411	0.693	0.249	0.366

Table 10: Results of network-centric ME evaluation framework with $\mathcal{G}$ = HINT, $\mathcal{S}$ = CGC, c= X_1, p_t = 0.05, t=20, robustness_iterations = 100

(a) Metrics for BLCA data. (411 samples | 76 CGC-CGC pairs)

Method	Precision	Sensitivity	F1 Score	$\text{Precision}_{\text{strict}}$	$\text{Sensitivity}_{\text{strict}}$	F1 $_{\text{strict}}$ Score
DISCOVER	0.750	0.080	0.145	0.750	0.080	0.145
Fisher's Exact Test	1.000	0.054	0.103	1.000	0.054	0.102
MEGSA	1.000	0.081	0.149	1.000	0.081	0.150
MEMO	0.667	0.082	0.146	0.667	0.082	0.146
WExT	0.615	0.110	0.186	0.667	0.110	0.189

(b) Metrics for BRCA data. (1026 samples | 42 CGC-CGC pairs)

Method	Precision	Sensitivity	F1 Score	$\text{Precision}_{\text{strict}}$	$\text{Sensitivity}_{\text{strict}}$	F1 $_{\text{strict}}$ Score
DISCOVER	0.643	0.450	0.529	0.652	0.375	0.476
DISCOVER Strat	0.750	0.405	0.526	0.789	0.405	0.535
Fisher's Exact Test	0.667	0.049	0.091	0.667	0.049	0.091
MEGSA	0.800	0.095	0.170	0.800	0.095	0.170
MEMO	0.655	0.480	0.554	0.682	0.400	0.504
WExT	0.606	0.556	0.580	0.654	0.472	0.548

(c) Metrics for COADREAD data. (498 samples | 198 CGC-CGC pairs)

Method	Precision	Sensitivity	F1 Score	$\text{Precision}_{\text{strict}}$	$\text{Sensitivity}_{\text{strict}}$	F1 $_{\text{strict}}$ Score
DISCOVER	0.623	0.204	0.308	0.702	0.177	0.283
DISCOVER Strat	0.800	0.064	0.118	0.800	0.064	0.119
Fisher's Exact Test	0.757	0.071	0.130	0.788	0.066	0.122
MEGSA	0.714	0.076	0.138	0.722	0.066	0.121
MEMO	0.620	0.302	0.406	0.597	0.212	0.313
WExT	0.636	0.367	0.466	0.716	0.304	0.427

(d) Metrics for LUAD data. (568 samples | 78 CGC-CGC pairs)

Method	Precision	Sensitivity	F1 Score	$\text{Precision}_{\text{strict}}$	$\text{Sensitivity}_{\text{strict}}$	F1 $_{\text{strict}}$ Score
DISCOVER	0.625	0.067	0.121	0.625	0.067	0.121
Fisher's Exact Test	0.667	0.026	0.050	0.667	0.026	0.050
MEGSA	0.667	0.026	0.050	0.667	0.026	0.050
MEMO	0.708	0.116	0.199	0.739	0.116	0.201
WExT	0.645	0.140	0.230	0.645	0.140	0.230

(e) Metrics for LUSC data. (485 samples | 60 CGC-CGC pairs)

Method	Precision	Sensitivity	F1 Score	$\text{Precision}_{\text{strict}}$	$\text{Sensitivity}_{\text{strict}}$	F1 $_{\text{strict}}$ Score
DISCOVER	1.000	0.033	0.065	1.000	0.033	0.064
Fisher's Exact Test	1.000	0.033	0.065	1.000	0.033	0.064
MEGSA	1.000	0.067	0.126	1.000	0.067	0.126
MEMO	0.667	0.069	0.125	0.667	0.069	0.125
WExT	0.667	0.068	0.123	0.667	0.068	0.123

(f) Metrics for SKCM data. (468 samples | 438 CGC-CGC pairs)

Method	Precision	Sensitivity	F1 Score	$\text{Precision}_{\text{strict}}$	$\text{Sensitivity}_{\text{strict}}$	F1 $_{\text{strict}}$ Score
DISCOVER	0.875	0.032	0.062	0.875	0.032	0.062
Fisher's Exact Test	NaN	0.000	NaN	NaN	0.000	NaN
MEGSA	1.000	0.014	0.027	1.000	0.014	0.028
WExT	0.644	0.077	0.138	0.640	0.076	0.136

(g) Metrics for STAD data. (438 samples | 150 CGC-CGC pairs)

Method	Precision	Sensitivity	F1 Score	$\text{Precision}_{\text{strict}}$	$\text{Sensitivity}_{\text{strict}}$	F1 $_{\text{strict}}$ Score
DISCOVER	0.581	0.124	0.204	0.625	0.103	0.177
Fisher's Exact Test	0.800	0.026	0.051	0.800	0.026	0.050
MEGSA	0.800	0.027	0.052	0.800	0.027	0.052
WExT	0.600	0.185	0.283	0.629	0.151	0.244

(h) Metrics for UCEC data. (531 samples | 1024 CGC-CGC pairs)

Method	Precision	Sensitivity	F1 Score	$\text{Precision}_{\text{strict}}$	$\text{Sensitivity}_{\text{strict}}$	F1 $_{\text{strict}}$ Score
DISCOVER	0.658	0.209	0.317	0.733	0.170	0.276
Fisher's Exact Test	0.846	0.011	0.021	0.846	0.011	0.022
MEGSA	0.778	0.014	0.027	0.824	0.014	0.028
WExT	0.628	0.304	0.410	0.704	0.255	0.374

Table 11: Results of network-centric ME evaluation framework with $\mathcal{G}$ = STRING, $\mathcal{S}$ = CGC, c= X_1, p_t = 0.05, t=20, robustness_iterations = 100

(a) Metrics for BLCA data. (411 samples | 122 CGC-CGC pairs)

Method	Precision	Sensitivity	F1 Score	$\text{Precision}_{\text{strict}}$	$\text{Sensitivity}_{\text{strict}}$	F1 $_{\text{strict}}$ Score
DISCOVER	0.800	0.067	0.124	0.800	0.067	0.124
Fisher's Exact Test	1.000	0.034	0.065	1.000	0.034	0.066
MEGSA	1.000	0.033	0.065	1.000	0.033	0.064
MEMO	0.667	0.067	0.122	0.667	0.067	0.122
WExT	0.714	0.085	0.152	0.714	0.085	0.152

(b) Metrics for BRCA data. (1026 samples | 94 CGC-CGC pairs)

Method	Precision	Sensitivity	F1 Score	$\text{Precision}_{\text{strict}}$	$\text{Sensitivity}_{\text{strict}}$	F1 $_{\text{strict}}$ Score
DISCOVER	0.667	0.322	0.434	0.714	0.287	0.409
DISCOVER Strat	0.730	0.314	0.439	0.812	0.302	0.440
Fisher's Exact Test	0.667	0.022	0.042	0.667	0.022	0.043
MEGSA	0.800	0.043	0.081	0.800	0.043	0.082
MEMO	0.628	0.309	0.414	0.657	0.263	0.376
WExT	0.618	0.380	0.470	0.667	0.325	0.437

(c) Metrics for COADREAD data. (498 samples | 292 CGC-CGC pairs)

Method	Precision	Sensitivity	F1 Score	$\text{Precision}_{\text{strict}}$	$\text{Sensitivity}_{\text{strict}}$	F1 $_{\text{strict}}$ Score
DISCOVER	0.652	0.215	0.323	0.750	0.194	0.308
DISCOVER Strat	0.754	0.081	0.146	0.807	0.081	0.147
Fisher's Exact Test	0.882	0.052	0.097	0.933	0.048	0.091
MEGSA	0.842	0.055	0.103	0.882	0.052	0.098
MEMO	0.613	0.245	0.350	0.639	0.192	0.295
WExT	0.577	0.246	0.345	0.670	0.214	0.324

(d) Metrics for LUAD data. (568 samples | 110 CGC-CGC pairs)

Method	Precision	Sensitivity	F1 Score	$\text{Precision}_{\text{strict}}$	$\text{Sensitivity}_{\text{strict}}$	F1 $_{\text{strict}}$ Score
DISCOVER	0.750	0.113	0.196	0.923	0.113	0.201
Fisher's Exact Test	1.000	0.073	0.137	1.000	0.073	0.136
MEGSA	1.000	0.073	0.137	1.000	0.073	0.136
MEMO	0.600	0.115	0.193	0.706	0.115	0.198
WExT	0.621	0.176	0.274	0.750	0.176	0.285

(e) Metrics for LUSC data. (485 samples | 90 CGC-CGC pairs)

Method	Precision	Sensitivity	F1 Score	$\text{Precision}_{\text{strict}}$	$\text{Sensitivity}_{\text{strict}}$	F1 $_{\text{strict}}$ Score
DISCOVER	1.0	0.045	0.087	1.0	0.045	0.086
Fisher's Exact Test	1.0	0.022	0.044	1.0	0.022	0.043
MEGSA	1.0	0.047	0.089	1.0	0.047	0.090
MEMO	1.0	0.047	0.090	1.0	0.047	0.090
WExT	1.0	0.071	0.133	1.0	0.071	0.133

(f) Metrics for SKCM data. (468 samples | 560 CGC-CGC pairs)

Method	Precision	Sensitivity	F1 Score	$\text{Precision}_{\text{strict}}$	$\text{Sensitivity}_{\text{strict}}$	F1 $_{\text{strict}}$ Score
DISCOVER	0.806	0.046	0.087	0.828	0.044	0.084
Fisher's Exact Test	1.000	0.004	0.007	1.000	0.004	0.008
MEGSA	0.889	0.015	0.029	0.889	0.015	0.030
WExT	0.686	0.112	0.192	0.717	0.105	0.183

(g) Metrics for STAD data. (438 samples | 194 CGC-CGC pairs)

Method	Precision	Sensitivity	F1 Score	$\text{Precision}_{\text{strict}}$	$\text{Sensitivity}_{\text{strict}}$	F1 $_{\text{strict}}$ Score
DISCOVER	0.636	0.113	0.192	0.667	0.097	0.169
Fisher's Exact Test	1.000	0.010	0.021	1.000	0.010	0.020
MEGSA	0.667	0.010	0.020	0.667	0.010	0.020
WExT	0.605	0.139	0.227	0.639	0.123	0.206

(h) Metrics for UCEC data. (531 samples | 1566 CGC-CGC pairs)

Method	Precision	Sensitivity	F1 Score	$\text{Precision}_{\text{strict}}$	$\text{Sensitivity}_{\text{strict}}$	F1 $_{\text{strict}}$ Score
DISCOVER	0.672	0.170	0.271	0.745	0.140	0.236
Fisher's Exact Test	1.000	0.008	0.015	1.000	0.008	0.016
MEGSA	1.000	0.008	0.016	1.000	0.008	0.016
WExT	0.619	0.276	0.382	0.680	0.228	0.341

Table 12: Degree-normalized network-centric evaluations X_1 and $t = 20$

(a) Metrics for BLCA data.

Method	Precision	Sensitivity	F1 Score	$\text{Precision}_{\text{strict}}$	$\text{Sensitivity}_{\text{strict}}$	F1 $_{\text{strict}}$ Score
DISCOVER	0.875	0.432	0.531	0.875	0.432	0.531
Fisher's Exact Test	1.0	0.25	0.393	1.0	0.25	0.393
MEGSA	1.0	0.432	0.538	1.0	0.432	0.538
MEMO	0.7	0.35	0.43	0.7	0.35	0.43
WExT	0.667	0.379	0.453	0.667	0.379	0.453

(b) Metrics for BRCA data.

Method	Precision	Sensitivity	F1 Score	$\text{Precision}_{\text{strict}}$	$\text{Sensitivity}_{\text{strict}}$	F1 $_{\text{strict}}$ Score
DISCOVER	0.758	0.8	0.747	0.767	0.683	0.711
DISCOVER Strat	0.882	0.821	0.837	0.88	0.803	0.829
Fisher's Exact Test	1.0	0.6	0.667	1.0	0.6	0.667
MEGSA	1.0	0.6	0.667	1.0	0.6	0.667
MEMO	0.739	0.697	0.698	0.747	0.657	0.687
WExT	0.718	0.692	0.669	0.769	0.654	0.69

(c) Metrics for COADREAD data.

Method	Precision	Sensitivity	F1 Score	$\text{Precision}_{\text{strict}}$	$\text{Sensitivity}_{\text{strict}}$	F1 $_{\text{strict}}$ Score
DISCOVER	0.867	0.657	0.671	0.899	0.612	0.675
DISCOVER Strat	0.906	0.433	0.524	0.906	0.433	0.524
Fisher's Exact Test	0.806	0.307	0.381	0.778	0.287	0.357
MEGSA	0.865	0.485	0.549	0.852	0.471	0.536
MEMO	0.838	0.738	0.728	0.83	0.64	0.689
WExT	0.846	0.714	0.721	0.851	0.645	0.7

(d) Metrics for LUAD data.

Method	Precision	Sensitivity	F1 Score	$\text{Precision}_{\text{strict}}$	$\text{Sensitivity}_{\text{strict}}$	F1 $_{\text{strict}}$ Score
DISCOVER	0.939	0.757	0.781	0.914	0.7	0.743
Fisher's Exact Test	0.0	0.0	0.0	0.0	0.0	0.0
MEGSA	0.75	0.225	0.343	0.75	0.225	0.343
MEMO	0.8	0.606	0.646	0.794	0.572	0.629
WExT	0.687	0.575	0.586	0.714	0.552	0.595

(e) Metrics for LUSC data.

Method	Precision	Sensitivity	F1 Score	$\text{Precision}_{\text{strict}}$	$\text{Sensitivity}_{\text{strict}}$	F1 $_{\text{strict}}$ Score
DISCOVER	1.0	0.75	0.833	1.0	0.75	0.833
Fisher's Exact Test	1.0	0.75	0.833	1.0	0.75	0.833
MEGSA	1.0	0.75	0.833	1.0	0.75	0.833
MEMO	1.0	0.75	0.833	1.0	0.75	0.833
WExT	1.0	0.75	0.833	1.0	0.75	0.833

(f) Metrics for SKCM data.

Method	Precision	Sensitivity	F1 Score	$\text{Precision}_{\text{strict}}$	$\text{Sensitivity}_{\text{strict}}$	F1 $_{\text{strict}}$ Score
DISCOVER	0.857	0.353	0.445	0.857	0.353	0.445
Fisher's Exact Test	1.0	0.417	0.583	1.0	0.417	0.583
MEGSA	0.889	0.318	0.424	0.889	0.318	0.424
WExT	0.81	0.383	0.458	0.809	0.372	0.453

(g) Metrics for STAD data.

Method	Precision	Sensitivity	F1 Score	$\text{Precision}_{\text{strict}}$	$\text{Sensitivity}_{\text{strict}}$	F1 $_{\text{strict}}$ Score
DISCOVER	0.833	0.428	0.487	0.808	0.364	0.446
Fisher's Exact Test	0.0	0.0	0.0	0.0	0.0	0.0
MEGSA	0.75	0.25	0.375	0.75	0.25	0.375
WExT	0.829	0.528	0.585	0.809	0.475	0.553

(h) Metrics for UCEC data.

Method	Precision	Sensitivity	F1 Score	$\text{Precision}_{\text{strict}}$	$\text{Sensitivity}_{\text{strict}}$	F1 $_{\text{strict}}$ Score
DISCOVER	0.878	0.377	0.447	0.885	0.343	0.439
Fisher's Exact Test	0.939	0.127	0.21	0.939	0.127	0.21
MEGSA	0.946	0.174	0.268	0.946	0.174	0.268
WExT	0.794	0.471	0.526	0.797	0.422	0.51

Table 13: Results of network-centric ME evaluation framework with $\mathcal{G}$ = Intact (w conf. threshold 0.35), $\mathcal{S}$ = CGC, c= X_2, p_t = 0.05, t=20, robustness_iterations = 100

(a) Metrics for BLCA data. (411 samples | 24 CGC-CGC pairs)

Method	Precision	Sensitivity	F1 Score	$\text{Precision}_{\text{strict}}$	$\text{Sensitivity}_{\text{strict}}$	F1 $_{\text{strict}}$ Score
DISCOVER	1.000	0.083	0.154	1.000	0.083	0.153
Fisher's Exact Test	1.000	0.042	0.080	1.000	0.042	0.081
MEGSA	1.000	0.083	0.154	1.000	0.083	0.153
MEMO	0.667	0.083	0.148	0.667	0.083	0.148
WExT	0.500	0.083	0.143	0.500	0.083	0.142

(b) Metrics for BRCA data. (1026 samples | 9 CGC-CGC pairs)

Method	Precision	Sensitivity	F1 Score	$\text{Precision}_{\text{strict}}$	$\text{Sensitivity}_{\text{strict}}$	F1 $_{\text{strict}}$ Score
DISCOVER	0.625	0.556	0.588	0.714	0.556	0.625
DISCOVER Strat	0.714	0.556	0.625	0.714	0.556	0.625
Fisher's Exact Test	NaN	0.000	NaN	NaN	0.000	NaN
MEGSA	NaN	0.000	NaN	NaN	0.000	NaN
MEMO	0.625	0.556	0.588	0.714	0.556	0.625
WExT	0.625	0.556	0.588	0.714	0.556	0.625

(c) Metrics for COADREAD data. (498 samples | 107 CGC-CGC pairs)

Method	Precision	Sensitivity	F1 Score	$\text{Precision}_{\text{strict}}$	$\text{Sensitivity}_{\text{strict}}$	F1 $_{\text{strict}}$ Score
DISCOVER	0.537	0.276	0.365	0.579	0.210	0.308
DISCOVER Strat	0.455	0.048	0.086	0.400	0.038	0.069
Fisher's Exact Test	0.444	0.038	0.069	0.375	0.028	0.052
MEGSA	0.571	0.075	0.133	0.538	0.066	0.118
MEMO	0.566	0.388	0.460	0.495	0.215	0.300
WExT	0.575	0.438	0.497	0.596	0.295	0.395

(d) Metrics for LUAD data. (568 samples | 54 CGC-CGC pairs)

Method	Precision	Sensitivity	F1 Score	$\text{Precision}_{\text{strict}}$	$\text{Sensitivity}_{\text{strict}}$	F1 $_{\text{strict}}$ Score
DISCOVER	0.583	0.123	0.203	0.600	0.105	0.179
Fisher's Exact Test	NaN	0.000	NaN	NaN	0.000	NaN
MEGSA	0.400	0.034	0.062	0.400	0.034	0.063
MEMO	0.688	0.193	0.301	0.714	0.175	0.281
WExT	0.684	0.228	0.342	0.688	0.193	0.301

(e) Metrics for LUSC data. (485 samples | 22 CGC-CGC pairs)

Method	Precision	Sensitivity	F1 Score	$\text{Precision}_{\text{strict}}$	$\text{Sensitivity}_{\text{strict}}$	F1 $_{\text{strict}}$ Score
DISCOVER	NaN	0.000	NaN	NaN	0.000	NaN
Fisher's Exact Test	NaN	0.000	NaN	NaN	0.000	NaN
MEGSA	1.000	0.136	0.240	1.000	0.136	0.239
MEMO	0.667	0.087	0.154	0.667	0.087	0.154
WExT	0.400	0.087	0.143	0.500	0.087	0.148

(f) Metrics for SKCM data. (468 samples | 313 CGC-CGC pairs)

Method	Precision	Sensitivity	F1 Score	$\text{Precision}_{\text{strict}}$	$\text{Sensitivity}_{\text{strict}}$	F1 $_{\text{strict}}$ Score
DISCOVER	0.757	0.045	0.085	0.757	0.045	0.085
Fisher's Exact Test	1.000	0.006	0.013	1.000	0.006	0.012
MEGSA	0.923	0.019	0.038	0.923	0.019	0.037
WExT	0.679	0.118	0.201	0.714	0.115	0.198

(g) Metrics for STAD data. (438 samples | 70 CGC-CGC pairs)

Method	Precision	Sensitivity	F1 Score	$\text{Precision}_{\text{strict}}$	$\text{Sensitivity}_{\text{strict}}$	F1 $_{\text{strict}}$ Score
DISCOVER	0.600	0.129	0.212	0.727	0.114	0.197
Fisher's Exact Test	NaN	0.000	NaN	NaN	0.000	NaN
MEGSA	1.000	0.014	0.028	1.000	0.014	0.028
WExT	0.696	0.229	0.344	0.824	0.200	0.322

(h) Metrics for UCEC data. (531 samples | 1179 CGC-CGC pairs)

Method	Precision	Sensitivity	F1 Score	$\text{Precision}_{\text{strict}}$	$\text{Sensitivity}_{\text{strict}}$	F1 $_{\text{strict}}$ Score
DISCOVER	0.649	0.184	0.287	0.744	0.154	0.255
Fisher's Exact Test	0.833	0.008	0.017	0.833	0.008	0.016
MEGSA	0.793	0.010	0.019	0.793	0.010	0.020
WExT	0.607	0.286	0.389	0.686	0.241	0.357

Table 14: Results of network-centric ME evaluation framework with $\mathcal{G}$ = Intact (w conf. threshold 0.35), $\mathcal{S}$ = CGC, c= X_2, p_t = 0.05, t=20, robustness_iterations = 300

(a) Metrics for BLCA data. (411 samples | 24 CGC-CGC pairs)

Method	Precision	Sensitivity	F1 Score	$\text{Precision}_{\text{strict}}$	$\text{Sensitivity}_{\text{strict}}$	F1 $_{\text{strict}}$ Score
DISCOVER	1.000	0.083	0.154	1.000	0.083	0.153
Fisher's Exact Test	1.000	0.042	0.080	1.000	0.042	0.081
MEGSA	1.000	0.083	0.154	1.000	0.083	0.153
MEMO	0.667	0.083	0.148	0.667	0.083	0.148
WExT	0.500	0.083	0.143	0.500	0.083	0.142

(b) Metrics for BRCA data. (1026 samples | 9 CGC-CGC pairs)

Method	Precision	Sensitivity	F1 Score	$\text{Precision}_{\text{strict}}$	$\text{Sensitivity}_{\text{strict}}$	F1 $_{\text{strict}}$ Score
DISCOVER	0.625	0.556	0.588	0.714	0.556	0.625
DISCOVER Strat	0.714	0.556	0.625	0.714	0.556	0.625
Fisher's Exact Test	NaN	0.000	NaN	NaN	0.000	NaN
MEGSA	NaN	0.000	NaN	NaN	0.000	NaN
MEMO	0.625	0.556	0.588	0.714	0.556	0.625
WExT	0.625	0.556	0.588	0.714	0.556	0.625

(c) Metrics for COADREAD data. (498 samples | 107 CGC-CGC pairs)

Method	Precision	Sensitivity	F1 Score	$\text{Precision}_{\text{strict}}$	$\text{Sensitivity}_{\text{strict}}$	F1 $_{\text{strict}}$ Score
DISCOVER	0.536	0.278	0.366	0.575	0.213	0.311
DISCOVER Strat	0.500	0.056	0.101	0.500	0.056	0.101
Fisher's Exact Test	0.429	0.029	0.054	0.429	0.029	0.054
MEGSA	0.571	0.075	0.132	0.538	0.065	0.116
MEMO	0.554	0.383	0.453	0.500	0.224	0.309
WExT	0.578	0.441	0.500	0.619	0.308	0.411

(d) Metrics for LUAD data. (568 samples | 54 CGC-CGC pairs)

Method	Precision	Sensitivity	F1 Score	$\text{Precision}_{\text{strict}}$	$\text{Sensitivity}_{\text{strict}}$	F1 $_{\text{strict}}$ Score
DISCOVER	0.571	0.138	0.222	0.545	0.103	0.173
Fisher's Exact Test	NaN	0.000	NaN	NaN	0.000	NaN
MEGSA	0.400	0.034	0.062	0.400	0.034	0.063
MEMO	0.688	0.193	0.301	0.714	0.175	0.281
WExT	0.706	0.214	0.329	0.733	0.196	0.309

(e) Metrics for LUSC data. (485 samples | 22 CGC-CGC pairs)

Method	Precision	Sensitivity	F1 Score	$\text{Precision}_{\text{strict}}$	$\text{Sensitivity}_{\text{strict}}$	F1 $_{\text{strict}}$ Score
DISCOVER	NaN	0.000	NaN	NaN	0.000	NaN
Fisher's Exact Test	NaN	0.000	NaN	NaN	0.000	NaN
MEGSA	1.0	0.136	0.240	1.0	0.136	0.239
MEMO	1.0	0.095	0.174	1.0	0.095	0.174
WExT	0.4	0.095	0.154	0.5	0.095	0.160

(f) Metrics for SKCM data. (468 samples | 313 CGC-CGC pairs)

Method	Precision	Sensitivity	F1 Score	$\text{Precision}_{\text{strict}}$	$\text{Sensitivity}_{\text{strict}}$	F1 $_{\text{strict}}$ Score
DISCOVER	0.778	0.045	0.085	0.778	0.045	0.085
Fisher’s Exact Test	1.000	0.006	0.013	1.000	0.006	0.012
MEGSA	0.857	0.019	0.037	0.857	0.019	0.037
WExT	0.667	0.119	0.202	0.700	0.116	0.199

(g) Metrics for STAD data. (438 samples | 70 CGC-CGC pairs)

Method	Precision	Sensitivity	F1 Score	$\text{Precision}_{\text{strict}}$	$\text{Sensitivity}_{\text{strict}}$	F1 $_{\text{strict}}$ Score
DISCOVER	0.600	0.129	0.212	0.636	0.100	0.173
Fisher’s Exact Test	NaN	0.000	NaN	NaN	0.000	NaN
MEGSA	1.000	0.014	0.028	1.000	0.014	0.028
WExT	0.696	0.229	0.344	0.824	0.200	0.322

(h) Metrics for UCEC data. (531 samples | 1179 CGC-CGC pairs)

Method	Precision	Sensitivity	F1 Score	$\text{Precision}_{\text{strict}}$	$\text{Sensitivity}_{\text{strict}}$	F1 $_{\text{strict}}$ Score
DISCOVER	0.651	0.184	0.286	0.756	0.155	0.257
Fisher’s Exact Test	0.833	0.008	0.017	0.833	0.008	0.016
MEGSA	0.846	0.009	0.018	0.846	0.009	0.018
WExT	0.601	0.286	0.388	0.668	0.238	0.351

Table 15: Results of network-centric ME evaluation framework with $\mathcal{G}$ = Intact (w conf. threshold 0.35), $\mathcal{S}$ = CGC, c= X_2, p_t = 0.05, t=20, robustness_iterations = 500

(a) Metrics for BLCA data. (411 samples | 24 CGC-CGC pairs)

Method	Precision	Sensitivity	F1 Score	$\text{Precision}_{\text{strict}}$	$\text{Sensitivity}_{\text{strict}}$	F1 $_{\text{strict}}$ Score
DISCOVER	1.000	0.083	0.154	1.000	0.083	0.153
Fisher's Exact Test	1.000	0.042	0.080	1.000	0.042	0.081
MEGSA	1.000	0.083	0.154	1.000	0.083	0.153
MEMO	0.667	0.083	0.148	0.667	0.083	0.148
WExT	0.500	0.083	0.143	0.500	0.083	0.142

(b) Metrics for BRCA data. (1026 samples | 9 CGC-CGC pairs)

Method	Precision	Sensitivity	F1 Score	$\text{Precision}_{\text{strict}}$	$\text{Sensitivity}_{\text{strict}}$	F1 $_{\text{strict}}$ Score
DISCOVER	0.625	0.556	0.588	0.714	0.556	0.625
DISCOVER Strat	0.714	0.556	0.625	0.714	0.556	0.625
Fisher's Exact Test	NaN	0.000	NaN	NaN	0.000	NaN
MEGSA	NaN	0.000	NaN	NaN	0.000	NaN
MEMO	0.625	0.556	0.588	0.714	0.556	0.625
WExT	0.625	0.556	0.588	0.714	0.556	0.625

(c) Metrics for COADREAD data. (498 samples | 107 CGC-CGC pairs)

Method	Precision	Sensitivity	F1 Score	$\text{Precision}_{\text{strict}}$	$\text{Sensitivity}_{\text{strict}}$	F1 $_{\text{strict}}$ Score
DISCOVER	0.536	0.280	0.368	0.575	0.215	0.313
DISCOVER Strat	0.500	0.052	0.094	0.500	0.052	0.094
Fisher's Exact Test	0.500	0.038	0.070	0.500	0.038	0.071
MEGSA	0.615	0.075	0.134	0.583	0.066	0.119
MEMO	0.568	0.396	0.467	0.489	0.217	0.301
WExT	0.575	0.438	0.497	0.596	0.295	0.395

(d) Metrics for LUAD data. (568 samples | 54 CGC-CGC pairs)

Method	Precision	Sensitivity	F1 Score	$\text{Precision}_{\text{strict}}$	$\text{Sensitivity}_{\text{strict}}$	F1 $_{\text{strict}}$ Score
DISCOVER	0.571	0.138	0.222	0.545	0.103	0.173
Fisher's Exact Test	NaN	0.000	NaN	NaN	0.000	NaN
MEGSA	0.400	0.034	0.062	0.400	0.034	0.063
MEMO	0.688	0.193	0.301	0.714	0.175	0.281
WExT	0.684	0.228	0.342	0.688	0.193	0.301

(e) Metrics for LUSC data. (485 samples | 22 CGC-CGC pairs)

Method	Precision	Sensitivity	F1 Score	$\text{Precision}_{\text{strict}}$	$\text{Sensitivity}_{\text{strict}}$	F1 $_{\text{strict}}$ Score
DISCOVER	NaN	0.000	NaN	NaN	0.000	NaN
Fisher's Exact Test	NaN	0.000	NaN	NaN	0.000	NaN
MEGSA	1.0	0.136	0.240	1.000	0.136	0.239
MEMO	1.0	0.095	0.174	1.000	0.095	0.174
WExT	0.5	0.091	0.154	0.667	0.091	0.160

(f) Metrics for SKCM data. (468 samples | 313 CGC-CGC pairs)

Method	Precision	Sensitivity	F1 Score	$\text{Precision}_{\text{strict}}$	$\text{Sensitivity}_{\text{strict}}$	F1 $_{\text{strict}}$ Score
DISCOVER	0.737	0.045	0.084	0.737	0.045	0.085
Fisher's Exact Test	1.000	0.006	0.013	1.000	0.006	0.012
MEGSA	0.857	0.019	0.037	0.857	0.019	0.037
WExT	0.679	0.118	0.201	0.714	0.115	0.198

(g) Metrics for STAD data. (438 samples | 70 CGC-CGC pairs)

Method	Precision	Sensitivity	F1 Score	$\text{Precision}_{\text{strict}}$	$\text{Sensitivity}_{\text{strict}}$	F1 $_{\text{strict}}$ Score
DISCOVER	0.615	0.116	0.195	0.700	0.101	0.177
Fisher's Exact Test	NaN	0.000	NaN	NaN	0.000	NaN
MEGSA	1.000	0.014	0.028	1.000	0.014	0.028
WExT	0.696	0.229	0.344	0.824	0.200	0.322

(h) Metrics for UCEC data. (531 samples | 1179 CGC-CGC pairs)

Method	Precision	Sensitivity	F1 Score	$\text{Precision}_{\text{strict}}$	$\text{Sensitivity}_{\text{strict}}$	F1 $_{\text{strict}}$ Score
DISCOVER	0.653	0.184	0.287	0.755	0.155	0.257
Fisher's Exact Test	0.833	0.008	0.017	0.833	0.008	0.016
MEGSA	0.786	0.009	0.018	0.786	0.009	0.018
WExT	0.603	0.286	0.388	0.683	0.242	0.357

Table 16: Results of network-centric ME evaluation framework with $\mathcal{G}$ = Intact (w conf. threshold 0.35), $\mathcal{S}$ = CGC, c= X_2, p_t = 0.01, t=20, robustness_iterations = 100

(a) Metrics for BLCA data. (411 samples | 24 CGC-CGC pairs)

Method	Precision	Sensitivity	F1 Score	$\text{Precision}_{\text{strict}}$	$\text{Sensitivity}_{\text{strict}}$	F1 $_{\text{strict}}$ Score
DISCOVER	1.0	0.042	0.08	1.0	0.042	0.081
Fisher's Exact Test	NaN	0.000	NaN	NaN	0.000	NaN
MEGSA	NaN	0.000	NaN	NaN	0.000	NaN
MEMO	1.0	0.042	0.08	1.0	0.042	0.081
WExT	1.0	0.042	0.08	1.0	0.042	0.081

(b) Metrics for BRCA data. (1026 samples | 9 CGC-CGC pairs)

Method	Precision	Sensitivity	F1 Score	$\text{Precision}_{\text{strict}}$	$\text{Sensitivity}_{\text{strict}}$	F1 $_{\text{strict}}$ Score
DISCOVER	0.75	0.333	0.462	0.75	0.333	0.461
DISCOVER Strat	0.50	0.111	0.182	0.50	0.111	0.182
Fisher's Exact Test	NaN	0.000	NaN	NaN	0.000	NaN
MEGSA	NaN	0.000	NaN	NaN	0.000	NaN
MEMO	0.60	0.333	0.429	0.60	0.333	0.428
WExT	0.60	0.333	0.429	0.60	0.333	0.428

(c) Metrics for COADREAD data. (498 samples | 107 CGC-CGC pairs)

Method	Precision	Sensitivity	F1 Score	$\text{Precision}_{\text{strict}}$	$\text{Sensitivity}_{\text{strict}}$	F1 $_{\text{strict}}$ Score
DISCOVER	0.613	0.178	0.275	0.625	0.140	0.229
DISCOVER Strat	0.000	0.000	NaN	0.000	0.000	NaN
Fisher's Exact Test	0.000	0.000	NaN	0.000	0.000	NaN
MEGSA	0.500	0.009	0.018	0.500	0.009	0.018
MEMO	0.596	0.321	0.417	0.474	0.170	0.250
WExT	0.603	0.330	0.427	0.590	0.217	0.317

(d) Metrics for LUAD data. (568 samples | 54 CGC-CGC pairs)

Method	Precision	Sensitivity	F1 Score	$\text{Precision}_{\text{strict}}$	$\text{Sensitivity}_{\text{strict}}$	F1 $_{\text{strict}}$ Score
DISCOVER	0.750	0.051	0.095	0.750	0.051	0.096
Fisher's Exact Test	NaN	0.000	NaN	NaN	0.000	NaN
MEGSA	1.000	0.034	0.066	1.000	0.034	0.066
MEMO	0.778	0.119	0.206	0.750	0.102	0.180
WExT	0.640	0.137	0.225	0.667	0.120	0.203

(e) Metrics for LUSC data. (485 samples | 22 CGC-CGC pairs)

Method	Precision	Sensitivity	F1 Score	$\text{Precision}_{\text{strict}}$	$\text{Sensitivity}_{\text{strict}}$	F1 $_{\text{strict}}$ Score
DISCOVER	NaN	0.0	NaN	NaN	0.0	NaN
Fisher's Exact Test	NaN	0.0	NaN	NaN	0.0	NaN
MEGSA	NaN	0.0	NaN	NaN	0.0	NaN
MEMO	NaN	0.0	NaN	NaN	0.0	NaN
WExT	NaN	0.0	NaN	NaN	0.0	NaN

(f) Metrics for SKCM data. (468 samples | 313 CGC-CGC pairs)

Method	Precision	Sensitivity	F1 Score	$\text{Precision}_{\text{strict}}$	$\text{Sensitivity}_{\text{strict}}$	F1 $_{\text{strict}}$ Score
DISCOVER	0.833	0.016	0.031	0.833	0.016	0.031
Fisher's Exact Test	1.000	0.006	0.013	1.000	0.006	0.012
MEGSA	1.000	0.006	0.013	1.000	0.006	0.012
WExT	0.710	0.035	0.067	0.710	0.035	0.067

(g) Metrics for STAD data. (438 samples | 70 CGC-CGC pairs)

Method	Precision	Sensitivity	F1 Score	$\text{Precision}_{\text{strict}}$	$\text{Sensitivity}_{\text{strict}}$	F1 $_{\text{strict}}$ Score
DISCOVER	0.625	0.071	0.128	0.667	0.057	0.105
Fisher's Exact Test	NaN	0.000	NaN	NaN	0.000	NaN
MEGSA	NaN	0.000	NaN	NaN	0.000	NaN
WExT	0.583	0.101	0.173	0.714	0.072	0.131

(h) Metrics for UCEC data. (531 samples | 1179 CGC-CGC pairs)

Method	Precision	Sensitivity	F1 Score	$\text{Precision}_{\text{strict}}$	$\text{Sensitivity}_{\text{strict}}$	F1 $_{\text{strict}}$ Score
DISCOVER	0.673	0.129	0.217	0.782	0.113	0.197
Fisher's Exact Test	1.000	0.005	0.010	1.000	0.005	0.010
MEGSA	1.000	0.005	0.010	1.000	0.005	0.010
WExT	0.640	0.205	0.311	0.744	0.170	0.277

Table 17: Results of network-centric ME evaluation framework with $\mathcal{G}$ = Intact (w conf. threshold 0.35), $\mathcal{S}$ = CGC, c= X_2, p_t = 0.1, t=20, robustness_iterations = 100

(a) Metrics for BLCA data. (411 samples | 24 CGC-CGC pairs)

Method	Precision	Sensitivity	F1 Score	$\text{Precision}_{\text{strict}}$	$\text{Sensitivity}_{\text{strict}}$	F1 $_{\text{strict}}$ Score
DISCOVER	0.667	0.083	0.148	0.667	0.083	0.148
Fisher's Exact Test	1.000	0.042	0.080	1.000	0.042	0.081
MEGSA	1.000	0.083	0.154	1.000	0.083	0.153
MEMO	0.500	0.083	0.143	0.500	0.083	0.142
WExT	0.400	0.083	0.138	0.400	0.083	0.137

(b) Metrics for BRCA data. (1026 samples | 9 CGC-CGC pairs)

Method	Precision	Sensitivity	F1 Score	$\text{Precision}_{\text{strict}}$	$\text{Sensitivity}_{\text{strict}}$	F1 $_{\text{strict}}$ Score
DISCOVER	0.625	0.556	0.588	0.714	0.556	0.625
DISCOVER Strat	0.667	0.667	0.667	0.714	0.556	0.625
Fisher's Exact Test	NaN	0.000	NaN	NaN	0.000	NaN
MEGSA	NaN	0.000	NaN	NaN	0.000	NaN
MEMO	0.625	0.556	0.588	0.714	0.556	0.625
WExT	0.667	0.667	0.667	0.714	0.556	0.625

(c) Metrics for COADREAD data. (498 samples | 107 CGC-CGC pairs)

Method	Precision	Sensitivity	F1 Score	$\text{Precision}_{\text{strict}}$	$\text{Sensitivity}_{\text{strict}}$	F1 $_{\text{strict}}$ Score
DISCOVER	0.529	0.333	0.409	0.553	0.241	0.336
DISCOVER Strat	0.547	0.136	0.218	0.535	0.108	0.180
Fisher's Exact Test	0.650	0.124	0.208	0.588	0.095	0.164
MEGSA	0.577	0.142	0.227	0.550	0.104	0.175
MEMO	0.578	0.449	0.505	0.509	0.262	0.346
WExT	0.558	0.457	0.503	0.600	0.314	0.412

(d) Metrics for LUAD data. (568 samples | 54 CGC-CGC pairs)

Method	Precision	Sensitivity	F1 Score	$\text{Precision}_{\text{strict}}$	$\text{Sensitivity}_{\text{strict}}$	F1 $_{\text{strict}}$ Score
DISCOVER	0.686	0.212	0.324	0.710	0.195	0.306
Fisher's Exact Test	1.000	0.034	0.067	1.000	0.034	0.066
MEGSA	0.500	0.086	0.147	0.556	0.086	0.149
MEMO	0.636	0.237	0.346	0.684	0.220	0.333
WExT	0.667	0.339	0.449	0.692	0.305	0.423

(e) Metrics for LUSC data. (485 samples | 22 CGC-CGC pairs)

Method	Precision	Sensitivity	F1 Score	$\text{Precision}_{\text{strict}}$	$\text{Sensitivity}_{\text{strict}}$	F1 $_{\text{strict}}$ Score
DISCOVER	0.500	0.100	0.167	0.667	0.100	0.174
Fisher's Exact Test	NaN	0.000	NaN	NaN	0.000	NaN
MEGSA	0.500	0.130	0.207	0.600	0.130	0.214
MEMO	0.364	0.089	0.143	0.444	0.089	0.148
WExT	0.500	0.136	0.214	0.600	0.136	0.222

(f) Metrics for SKCM data. (468 samples | 313 CGC-CGC pairs)

Method	Precision	Sensitivity	F1 Score	$\text{Precision}_{\text{strict}}$	$\text{Sensitivity}_{\text{strict}}$	F1 $_{\text{strict}}$ Score
DISCOVER	0.697	0.101	0.176	0.713	0.101	0.177
Fisher's Exact Test	1.000	0.006	0.013	1.000	0.006	0.012
MEGSA	0.818	0.029	0.056	0.818	0.029	0.056
WExT	0.616	0.176	0.274	0.650	0.173	0.273

(g) Metrics for STAD data. (438 samples | 70 CGC-CGC pairs)

Method	Precision	Sensitivity	F1 Score	$\text{Precision}_{\text{strict}}$	$\text{Sensitivity}_{\text{strict}}$	F1 $_{\text{strict}}$ Score
DISCOVER	0.696	0.229	0.344	0.765	0.186	0.299
Fisher's Exact Test	0.500	0.014	0.028	0.500	0.014	0.027
MEGSA	0.500	0.014	0.027	0.500	0.014	0.027
WExT	0.704	0.271	0.392	0.800	0.229	0.356

(h) Metrics for UCEC data. (531 samples | 1179 CGC-CGC pairs)

Method	Precision	Sensitivity	F1 Score	$\text{Precision}_{\text{strict}}$	$\text{Sensitivity}_{\text{strict}}$	F1 $_{\text{strict}}$ Score
DISCOVER	0.622	0.226	0.331	0.708	0.186	0.295
Fisher's Exact Test	0.857	0.015	0.030	0.850	0.014	0.028
MEGSA	0.862	0.021	0.041	0.889	0.020	0.039
WExT	0.584	0.353	0.440	0.657	0.294	0.406

Table 18: Results of network-centric ME evaluation framework with $\mathcal{G}$ = Intact (w conf. threshold 0.35), $\mathcal{S} = CGC_{SNV}$, c= X_2, p_t = 0.05, t=20, robustness_iterations = 100

(a) Metrics for BLCA data. (411 samples | 21 CGC_{SNV}-CGC_{SNV} pairs)

Method	Precision	Sensitivity	F1 Score	$\text{Precision}_{\text{strict}}$	$\text{Sensitivity}_{\text{strict}}$	F1 $_{\text{strict}}$ Score
DISCOVER	1.00	0.143	0.250	1.00	0.143	0.250
Fisher's Exact Test	1.00	0.095	0.174	1.00	0.095	0.174
MEGSA	1.00	0.143	0.250	1.00	0.143	0.250
MEMO	1.00	0.146	0.255	1.00	0.146	0.255
WExT	0.75	0.143	0.240	0.75	0.143	0.240

(b) Metrics for BRCA data. (1026 samples | 10 CGC_{SNV}-CGC_{SNV} pairs)

Method	Precision	Sensitivity	F1 Score	$\text{Precision}_{\text{strict}}$	$\text{Sensitivity}_{\text{strict}}$	F1 $_{\text{strict}}$ Score
DISCOVER	0.571	0.4	0.471	0.667	0.4	0.5
DISCOVER Strat	0.667	0.4	0.500	0.667	0.4	0.5
Fisher's Exact Test	NaN	0.0	NaN	NaN	0.0	NaN
MEGSA	NaN	0.0	NaN	NaN	0.0	NaN
MEMO	0.571	0.4	0.471	0.667	0.4	0.5
WExT	0.571	0.4	0.471	0.667	0.4	0.5

(c) Metrics for COADREAD data. (498 samples | 101 CGC_{SNV}-CGC_{SNV} pairs)

Method	Precision	Sensitivity	F1 Score	$\text{Precision}_{\text{strict}}$	$\text{Sensitivity}_{\text{strict}}$	F1 $_{\text{strict}}$ Score
DISCOVER	0.581	0.248	0.347	0.645	0.198	0.303
DISCOVER Strat	0.600	0.060	0.109	0.600	0.060	0.109
Fisher's Exact Test	0.500	0.040	0.073	0.500	0.040	0.074
MEGSA	0.667	0.080	0.143	0.636	0.070	0.126
MEMO	0.593	0.327	0.421	0.583	0.214	0.313
WExT	0.612	0.408	0.490	0.696	0.318	0.437

(d) Metrics for LUAD data. (568 samples | 46 CGC_{SNV}-CGC_{SNV} pairs)

Method	Precision	Sensitivity	F1 Score	$\text{Precision}_{\text{strict}}$	$\text{Sensitivity}_{\text{strict}}$	F1 $_{\text{strict}}$ Score
DISCOVER	0.545	0.136	0.218	0.556	0.114	0.189
Fisher's Exact Test	NaN	0.000	NaN	NaN	0.000	NaN
MEGSA	0.500	0.043	0.080	0.500	0.043	0.079
MEMO	0.690	0.227	0.342	0.720	0.205	0.319
WExT	0.719	0.258	0.380	0.741	0.225	0.345

(e) Metrics for LUSC data. (485 samples | 22 CGC_{SNV}-CGC_{SNV} pairs)

Method	Precision	Sensitivity	F1 Score	$\text{Precision}_{\text{strict}}$	$\text{Sensitivity}_{\text{strict}}$	F1 $_{\text{strict}}$ Score
DISCOVER	NaN	0.000	NaN	NaN	0.000	NaN
Fisher's Exact Test	NaN	0.000	NaN	NaN	0.000	NaN
MEGSA	1.000	0.136	0.240	1.000	0.136	0.239
MEMO	0.667	0.091	0.160	0.667	0.091	0.160
WExT	0.400	0.087	0.143	0.500	0.087	0.148

(f) Metrics for SKCM data. (468 samples | 228 CGC_{SNV}-CGC_{SNV} pairs)

Method	Precision	Sensitivity	F1 Score	$\text{Precision}_{\text{strict}}$	$\text{Sensitivity}_{\text{strict}}$	F1 $_{\text{strict}}$ Score
DISCOVER	0.842	0.035	0.068	0.842	0.035	0.067
Fisher's Exact Test	1.000	0.009	0.017	1.000	0.009	0.018
MEGSA	0.800	0.018	0.034	0.800	0.018	0.035
WExT	0.699	0.129	0.218	0.747	0.125	0.214

(g) Metrics for STAD data. (438 samples | 53 CGC_{SNV}-CGC_{SNV} pairs)

Method	Precision	Sensitivity	F1 Score	$\text{Precision}_{\text{strict}}$	$\text{Sensitivity}_{\text{strict}}$	F1 $_{\text{strict}}$ Score
DISCOVER	0.579	0.107	0.180	0.692	0.087	0.155
Fisher's Exact Test	NaN	0.000	NaN	NaN	0.000	NaN
MEGSA	1.000	0.019	0.037	1.000	0.019	0.037
WExT	0.667	0.226	0.338	0.769	0.189	0.303

(h) Metrics for UCEC data. (531 samples | 739 CGC_{SNV}-CGC_{SNV} pairs)

Method	Precision	Sensitivity	F1 Score	$\text{Precision}_{\text{strict}}$	$\text{Sensitivity}_{\text{strict}}$	F1 $_{\text{strict}}$ Score
DISCOVER	0.658	0.212	0.321	0.771	0.182	0.294
Fisher's Exact Test	0.833	0.014	0.027	0.833	0.014	0.028
MEGSA	0.846	0.015	0.029	0.846	0.015	0.029
WExT	0.637	0.329	0.434	0.731	0.282	0.407

Table 19: Results of network-centric ME evaluation framework with $\mathcal{G}$ = Intact (w conf. threshold 0.35), $\mathcal{S}$ = Intogen , c= X_2, p_t = 0.05, t=20, robustness_iterations = 100

(a) Metrics for BLCA data. (411 samples | 23 Intogen-Intogen pairs)

Method	Precision	Sensitivity	F1 Score	$\text{Precision}_{\text{strict}}$	$\text{Sensitivity}_{\text{strict}}$	F1 $_{\text{strict}}$ Score
DISCOVER	1.00	0.130	0.231	1.00	0.130	0.230
Fisher's Exact Test	1.00	0.043	0.083	1.00	0.043	0.082
MEGSA	1.00	0.087	0.160	1.00	0.087	0.160
MEMO	0.75	0.130	0.222	0.75	0.130	0.222
WExT	0.60	0.130	0.214	0.60	0.130	0.214

(b) Metrics for BRCA data. (1026 samples | 7 Intogen-Intogen pairs)

Method	Precision	Sensitivity	F1 Score	$\text{Precision}_{\text{strict}}$	$\text{Sensitivity}_{\text{strict}}$	F1 $_{\text{strict}}$ Score
DISCOVER	0.6	0.429	0.5	0.6	0.429	0.5
DISCOVER Strat	0.6	0.429	0.5	0.6	0.429	0.5
Fisher's Exact Test	NaN	0.000	NaN	NaN	0.000	NaN
MEGSA	NaN	0.000	NaN	NaN	0.000	NaN
MEMO	0.6	0.429	0.5	0.6	0.429	0.5
WExT	0.6	0.429	0.5	0.6	0.429	0.5

(c) Metrics for COADREAD data. (498 samples | 105 Intogen-Intogen pairs)

Method	Precision	Sensitivity	F1 Score	$\text{Precision}_{\text{strict}}$	$\text{Sensitivity}_{\text{strict}}$	F1 $_{\text{strict}}$ Score
DISCOVER	0.564	0.301	0.392	0.590	0.223	0.324
DISCOVER Strat	0.545	0.058	0.104	0.545	0.058	0.105
Fisher's Exact Test	0.400	0.038	0.070	0.333	0.029	0.053
MEGSA	0.571	0.076	0.134	0.538	0.067	0.119
MEMO	0.581	0.417	0.486	0.562	0.262	0.357
WExT	0.590	0.467	0.521	0.630	0.324	0.428

(d) Metrics for LUAD data. (568 samples | 53 Intogen-Intogen pairs)

Method	Precision	Sensitivity	F1 Score	$\text{Precision}_{\text{strict}}$	$\text{Sensitivity}_{\text{strict}}$	F1 $_{\text{strict}}$ Score
DISCOVER	0.538	0.135	0.215	0.500	0.096	0.161
Fisher's Exact Test	NaN	0.000	NaN	NaN	0.000	NaN
MEGSA	0.250	0.019	0.035	0.250	0.019	0.035
MEMO	0.667	0.196	0.303	0.692	0.176	0.281
WExT	0.667	0.231	0.343	0.667	0.192	0.298

(e) Metrics for LUSC data. (485 samples | 24 Intogen-Intogen pairs)

Method	Precision	Sensitivity	F1 Score	$\text{Precision}_{\text{strict}}$	$\text{Sensitivity}_{\text{strict}}$	F1 $_{\text{strict}}$ Score
DISCOVER	1.0	0.042	0.080	1.0	0.042	0.081
Fisher's Exact Test	NaN	0.000	NaN	NaN	0.000	NaN
MEGSA	1.0	0.125	0.222	1.0	0.125	0.222
MEMO	1.0	0.125	0.222	1.0	0.125	0.222
WExT	0.5	0.120	0.194	0.6	0.120	0.200

(f) Metrics for SKCM data. (468 samples | 335 Intogen-Intogen pairs)

Method	Precision	Sensitivity	F1 Score	$\text{Precision}_{\text{strict}}$	$\text{Sensitivity}_{\text{strict}}$	F1 $_{\text{strict}}$ Score
DISCOVER	0.600	0.036	0.067	0.600	0.036	0.068
Fisher's Exact Test	1.000	0.006	0.012	1.000	0.006	0.012
MEGSA	0.750	0.018	0.035	0.750	0.018	0.035
WExT	0.615	0.110	0.186	0.642	0.107	0.183

(g) Metrics for STAD data. (438 samples | 65 Intogen-Intogen pairs)

Method	Precision	Sensitivity	F1 Score	$\text{Precision}_{\text{strict}}$	$\text{Sensitivity}_{\text{strict}}$	F1 $_{\text{strict}}$ Score
DISCOVER	0.600	0.138	0.225	0.636	0.108	0.185
Fisher's Exact Test	NaN	0.000	NaN	NaN	0.000	NaN
MEGSA	1.000	0.015	0.030	1.000	0.015	0.030
WExT	0.696	0.250	0.368	0.781	0.195	0.312

(h) Metrics for UCEC data. (531 samples | 1111 Intogen-Intogen pairs)

Method	Precision	Sensitivity	F1 Score	$\text{Precision}_{\text{strict}}$	$\text{Sensitivity}_{\text{strict}}$	F1 $_{\text{strict}}$ Score
DISCOVER	0.657	0.214	0.323	0.771	0.183	0.296
Fisher's Exact Test	0.875	0.013	0.025	0.875	0.013	0.026
MEGSA	0.882	0.014	0.027	0.882	0.014	0.028
WExT	0.616	0.323	0.424	0.707	0.271	0.392

Table 20: Results of network-centric ME evaluation framework with $\mathcal{G}$ = Intact (w conf. threshold 0.25), $\mathcal{S}$ = CGC, c= X_2, p_t = 0.05, t=20, robustness_iterations = 100

(a) Metrics for BLCA data. (411 samples | 30 CGC-CGC pairs)

Method	Precision	Sensitivity	F1 Score	$\text{Precision}_{\text{strict}}$	$\text{Sensitivity}_{\text{strict}}$	F1 $_{\text{strict}}$ Score
DISCOVER	1.00	0.100	0.182	1.00	0.100	0.182
Fisher's Exact Test	1.00	0.067	0.125	1.00	0.067	0.126
MEGSA	1.00	0.133	0.235	1.00	0.133	0.235
MEMO	0.75	0.100	0.176	0.75	0.100	0.176
WExT	0.80	0.133	0.229	0.80	0.133	0.228

(b) Metrics for BRCA data. (1026 samples | 15 CGC-CGC pairs)

Method	Precision	Sensitivity	F1 Score	$\text{Precision}_{\text{strict}}$	$\text{Sensitivity}_{\text{strict}}$	F1 $_{\text{strict}}$ Score
DISCOVER	0.900	0.600	0.720	1.0	0.600	0.75
DISCOVER Strat	1.000	0.600	0.750	1.0	0.600	0.75
Fisher's Exact Test	NaN	0.000	NaN	NaN	0.000	NaN
MEGSA	NaN	0.000	NaN	NaN	0.000	NaN
MEMO	0.900	0.600	0.720	1.0	0.600	0.75
WExT	0.909	0.667	0.769	1.0	0.667	0.80

(c) Metrics for COADREAD data. (498 samples | 177 CGC-CGC pairs)

Method	Precision	Sensitivity	F1 Score	$\text{Precision}_{\text{strict}}$	$\text{Sensitivity}_{\text{strict}}$	F1 $_{\text{strict}}$ Score
DISCOVER	0.560	0.237	0.333	0.611	0.186	0.285
DISCOVER Strat	0.545	0.068	0.121	0.550	0.062	0.111
Fisher's Exact Test	0.600	0.068	0.122	0.611	0.062	0.113
MEGSA	0.655	0.102	0.177	0.667	0.091	0.160
MEMO	0.585	0.335	0.426	0.519	0.198	0.287
WExT	0.602	0.371	0.459	0.635	0.269	0.378

(d) Metrics for LUAD data. (568 samples | 80 CGC-CGC pairs)

Method	Precision	Sensitivity	F1 Score	$\text{Precision}_{\text{strict}}$	$\text{Sensitivity}_{\text{strict}}$	F1 $_{\text{strict}}$ Score
DISCOVER	0.632	0.150	0.242	0.688	0.138	0.230
Fisher's Exact Test	NaN	0.000	NaN	NaN	0.000	NaN
MEGSA	0.571	0.051	0.093	0.571	0.051	0.094
MEMO	0.680	0.215	0.327	0.714	0.190	0.300
WExT	0.679	0.232	0.345	0.708	0.207	0.320

(e) Metrics for LUSC data. (485 samples | 31 CGC-CGC pairs)

Method	Precision	Sensitivity	F1 Score	$\text{Precision}_{\text{strict}}$	$\text{Sensitivity}_{\text{strict}}$	F1 $_{\text{strict}}$ Score
DISCOVER	1.00	0.033	0.065	1.00	0.033	0.064
Fisher's Exact Test	1.00	0.032	0.062	1.00	0.032	0.062
MEGSA	1.00	0.097	0.176	1.00	0.097	0.177
MEMO	1.00	0.103	0.188	1.00	0.103	0.187
WExT	0.75	0.100	0.176	0.75	0.100	0.176

(f) Metrics for SKCM data. (468 samples | 522 CGC-CGC pairs)

Method	Precision	Sensitivity	F1 Score	$\text{Precision}_{\text{strict}}$	$\text{Sensitivity}_{\text{strict}}$	F1 $_{\text{strict}}$ Score
DISCOVER	0.814	0.046	0.088	0.814	0.046	0.087
Fisher's Exact Test	1.000	0.004	0.008	1.000	0.004	0.008
MEGSA	0.778	0.013	0.026	0.778	0.013	0.026
WExT	0.691	0.101	0.176	0.716	0.099	0.174

(g) Metrics for STAD data. (438 samples | 107 CGC-CGC pairs)

Method	Precision	Sensitivity	F1 Score	$\text{Precision}_{\text{strict}}$	$\text{Sensitivity}_{\text{strict}}$	F1 $_{\text{strict}}$ Score
DISCOVER	0.621	0.171	0.269	0.750	0.143	0.240
Fisher's Exact Test	1.000	0.019	0.037	1.000	0.019	0.037
MEGSA	0.800	0.037	0.071	0.800	0.037	0.071
WExT	0.619	0.243	0.349	0.724	0.196	0.308

(h) Metrics for UCEC data. (531 samples | 1794 CGC-CGC pairs)

Method	Precision	Sensitivity	F1 Score	$\text{Precision}_{\text{strict}}$	$\text{Sensitivity}_{\text{strict}}$	F1 $_{\text{strict}}$ Score
DISCOVER	0.664	0.164	0.264	0.768	0.140	0.237
Fisher's Exact Test	0.824	0.008	0.015	0.824	0.008	0.016
MEGSA	0.783	0.010	0.020	0.818	0.010	0.020
WExT	0.619	0.267	0.373	0.695	0.229	0.344

Table 21: Results of network-centric ME evaluation framework with $\mathcal{G}$ = Intact (w conf. threshold 0.45), $\mathcal{S}$ = CGC, c= X_2, p_t = 0.05, t=20, robustness_iterations = 100

(a) Metrics for BLCA data. (411 samples | 12 CGC-CGC pairs)

Method	Precision	Sensitivity	F1 Score	$\text{Precision}_{\text{strict}}$	$\text{Sensitivity}_{\text{strict}}$	F1 $_{\text{strict}}$ Score
DISCOVER	1.0	0.083	0.154	1.0	0.083	0.153
Fisher's Exact Test	1.0	0.083	0.154	1.0	0.083	0.153
MEGSA	1.0	0.083	0.154	1.0	0.083	0.153
MEMO	0.5	0.077	0.133	0.5	0.077	0.133
WExT	0.5	0.083	0.143	0.5	0.083	0.142

(b) Metrics for BRCA data. (1026 samples | 1 CGC-CGC pairs)

Method	Precision	Sensitivity	F1 Score	$\text{Precision}_{\text{strict}}$	$\text{Sensitivity}_{\text{strict}}$	F1 $_{\text{strict}}$ Score
DISCOVER	1.0	1.0	1.0	1.0	1.0	1.0
DISCOVER Strat	1.0	1.0	1.0	1.0	1.0	1.0
Fisher's Exact Test	NaN	0.0	NaN	NaN	0.0	NaN
MEGSA	NaN	0.0	NaN	NaN	0.0	NaN
MEMO	1.0	1.0	1.0	1.0	1.0	1.0
WExT	1.0	1.0	1.0	1.0	1.0	1.0

(c) Metrics for COADREAD data. (498 samples | 15 CGC-CGC pairs)

Method	Precision	Sensitivity	F1 Score	$\text{Precision}_{\text{strict}}$	$\text{Sensitivity}_{\text{strict}}$	F1 $_{\text{strict}}$ Score
DISCOVER	0.467	0.467	0.467	0.556	0.333	0.417
DISCOVER Strat	0.333	0.133	0.190	0.200	0.067	0.100
Fisher's Exact Test	0.333	0.062	0.105	0.333	0.062	0.105
MEGSA	0.400	0.133	0.200	0.250	0.067	0.106
MEMO	0.485	0.516	0.500	0.286	0.194	0.231
WExT	0.529	0.643	0.581	0.600	0.429	0.500

(d) Metrics for LUAD data. (568 samples | 12 CGC-CGC pairs)

Method	Precision	Sensitivity	F1 Score	$\text{Precision}_{\text{strict}}$	$\text{Sensitivity}_{\text{strict}}$	F1 $_{\text{strict}}$ Score
DISCOVER	0.571	0.308	0.400	0.600	0.231	0.334
Fisher's Exact Test	NaN	0.000	NaN	NaN	0.000	NaN
MEGSA	0.667	0.167	0.267	0.667	0.167	0.267
MEMO	0.625	0.385	0.476	0.667	0.308	0.421
WExT	0.667	0.333	0.444	0.600	0.250	0.353

(e) Metrics for LUSC data. (485 samples | 5 CGC-CGC pairs)

Method	Precision	Sensitivity	F1 Score	$\text{Precision}_{\text{strict}}$	$\text{Sensitivity}_{\text{strict}}$	F1 $_{\text{strict}}$ Score
DISCOVER	NaN	0.0	NaN	NaN	0.0	NaN
Fisher's Exact Test	NaN	0.0	NaN	NaN	0.0	NaN
MEGSA	0.667	0.4	0.500	0.5	0.2	0.286
MEMO	1.000	0.2	0.333	1.0	0.2	0.333
WExT	0.333	0.2	0.250	0.5	0.2	0.286

(f) Metrics for SKCM data. (468 samples | 104 CGC-CGC pairs)

Method	Precision	Sensitivity	F1 Score	$\text{Precision}_{\text{strict}}$	$\text{Sensitivity}_{\text{strict}}$	F1 $_{\text{strict}}$ Score
DISCOVER	0.800	0.039	0.074	0.8	0.039	0.074
Fisher's Exact Test	NaN	0.000	NaN	NaN	0.000	NaN
MEGSA	NaN	0.000	NaN	NaN	0.000	NaN
WExT	0.714	0.144	0.240	0.7	0.135	0.226

(g) Metrics for STAD data. (438 samples | 16 CGC-CGC pairs)

Method	Precision	Sensitivity	F1 Score	$\text{Precision}_{\text{strict}}$	$\text{Sensitivity}_{\text{strict}}$	F1 $_{\text{strict}}$ Score
DISCOVER	0.667	0.250	0.364	1.0	0.250	0.400
Fisher's Exact Test	NaN	0.000	NaN	NaN	0.000	NaN
MEGSA	1.000	0.062	0.118	1.0	0.062	0.117
WExT	0.571	0.250	0.348	1.0	0.250	0.400

(h) Metrics for UCEC data. (531 samples | 425 CGC-CGC pairs)

Method	Precision	Sensitivity	F1 Score	$\text{Precision}_{\text{strict}}$	$\text{Sensitivity}_{\text{strict}}$	F1 $_{\text{strict}}$ Score
DISCOVER	0.614	0.230	0.334	0.713	0.182	0.290
Fisher's Exact Test	0.778	0.016	0.032	0.778	0.016	0.031
MEGSA	0.818	0.021	0.041	0.818	0.021	0.041
WExT	0.565	0.319	0.408	0.633	0.250	0.358

Table 22: Results of network-centric ME evaluation framework with $\mathcal{G}$ = HINT, $\mathcal{S}$ = CGC, c= X_2, p_t = 0.05, t=20, robustness_iterations = 100

(a) Metrics for BLCA data. (411 samples | 6 CGC-CGC pairs)

Method	Precision	Sensitivity	F1 Score	$\text{Precision}_{\text{strict}}$	$\text{Sensitivity}_{\text{strict}}$	F1 $_{\text{strict}}$ Score
DISCOVER	NaN	0.0	NaN	NaN	0.0	NaN
Fisher's Exact Test	NaN	0.0	NaN	NaN	0.0	NaN
MEGSA	NaN	0.0	NaN	NaN	0.0	NaN
MEMO	NaN	0.0	NaN	NaN	0.0	NaN
WExT	NaN	0.0	NaN	NaN	0.0	NaN

(b) Metrics for BRCA data. (1026 samples | 3 CGC-CGC pairs)

Method	Precision	Sensitivity	F1 Score	$\text{Precision}_{\text{strict}}$	$\text{Sensitivity}_{\text{strict}}$	F1 $_{\text{strict}}$ Score
DISCOVER	NaN	0.0	NaN	NaN	0.0	NaN
DISCOVER Strat	NaN	0.0	NaN	NaN	0.0	NaN
Fisher's Exact Test	NaN	0.0	NaN	NaN	0.0	NaN
MEGSA	NaN	0.0	NaN	NaN	0.0	NaN
MEMO	NaN	0.0	NaN	NaN	0.0	NaN
WExT	NaN	0.0	NaN	NaN	0.0	NaN

(c) Metrics for COADREAD data. (498 samples | 58 CGC-CGC pairs)

Method	Precision	Sensitivity	F1 Score	$\text{Precision}_{\text{strict}}$	$\text{Sensitivity}_{\text{strict}}$	F1 $_{\text{strict}}$ Score
DISCOVER	0.455	0.273	0.341	0.478	0.200	0.282
DISCOVER Strat	0.364	0.069	0.116	0.333	0.052	0.090
Fisher's Exact Test	0.600	0.158	0.250	0.583	0.123	0.203
MEGSA	0.600	0.155	0.247	0.583	0.121	0.200
MEMO	0.529	0.474	0.500	0.344	0.193	0.247
WExT	0.537	0.518	0.527	0.559	0.339	0.422

(d) Metrics for LUAD data. (568 samples | 24 CGC-CGC pairs)

Method	Precision	Sensitivity	F1 Score	$\text{Precision}_{\text{strict}}$	$\text{Sensitivity}_{\text{strict}}$	F1 $_{\text{strict}}$ Score
DISCOVER	0.667	0.083	0.148	0.667	0.083	0.148
Fisher's Exact Test	NaN	0.000	NaN	NaN	0.000	NaN
MEGSA	0.000	0.000	NaN	0.000	0.000	NaN
MEMO	0.800	0.167	0.276	0.800	0.167	0.276
WExT	0.800	0.167	0.276	0.800	0.167	0.276

(e) Metrics for LUSC data. (485 samples | 9 CGC-CGC pairs)

Method	Precision	Sensitivity	F1 Score	$\text{Precision}_{\text{strict}}$	$\text{Sensitivity}_{\text{strict}}$	F1 $_{\text{strict}}$ Score
DISCOVER	NaN	0.000	NaN	NaN	0.000	NaN
Fisher's Exact Test	NaN	0.000	NaN	NaN	0.000	NaN
MEGSA	1.000	0.222	0.364	1.0	0.222	0.363
MEMO	1.000	0.250	0.400	1.0	0.250	0.400
WExT	0.667	0.250	0.364	1.0	0.250	0.400

(f) Metrics for SKCM data. (468 samples | 237 CGC-CGC pairs)

Method	Precision	Sensitivity	F1 Score	$\text{Precision}_{\text{strict}}$	$\text{Sensitivity}_{\text{strict}}$	F1 $_{\text{strict}}$ Score
DISCOVER	0.900	0.038	0.073	0.900	0.038	0.073
Fisher's Exact Test	NaN	0.000	NaN	NaN	0.000	NaN
MEGSA	0.800	0.017	0.033	0.800	0.017	0.033
WExT	0.667	0.085	0.152	0.655	0.081	0.144

(g) Metrics for STAD data. (438 samples | 33 CGC-CGC pairs)

Method	Precision	Sensitivity	F1 Score	$\text{Precision}_{\text{strict}}$	$\text{Sensitivity}_{\text{strict}}$	F1 $_{\text{strict}}$ Score
DISCOVER	0.588	0.303	0.400	0.818	0.273	0.409
Fisher's Exact Test	0.750	0.091	0.162	0.750	0.091	0.162
MEGSA	0.750	0.091	0.162	0.750	0.091	0.162
WExT	0.609	0.424	0.500	0.786	0.333	0.468

(h) Metrics for UCEC data. (531 samples | 721 CGC-CGC pairs)

Method	Precision	Sensitivity	F1 Score	$\text{Precision}_{\text{strict}}$	$\text{Sensitivity}_{\text{strict}}$	F1 $_{\text{strict}}$ Score
DISCOVER	0.629	0.233	0.340	0.748	0.193	0.307
Fisher's Exact Test	0.714	0.014	0.027	0.769	0.014	0.027
MEGSA	0.733	0.015	0.030	0.786	0.015	0.029
WExT	0.605	0.343	0.438	0.713	0.285	0.407

Table 23: Results of network-centric ME evaluation framework with $\mathcal{G}$ = STRING, $\mathcal{S}$ = CGC, c= X_2, p_t = 0.05, t=20, robustness_iterations = 100

(a) Metrics for BLCA data. (411 samples | 13 CGC-CGC pairs)

Method	Precision	Sensitivity	F1 Score	$\text{Precision}_{\text{strict}}$	$\text{Sensitivity}_{\text{strict}}$	F1 $_{\text{strict}}$ Score
DISCOVER	0.500	0.071	0.125	0.500	0.071	0.124
Fisher's Exact Test	NaN	0.000	NaN	NaN	0.000	NaN
MEGSA	NaN	0.000	NaN	NaN	0.000	NaN
MEMO	0.500	0.077	0.133	0.500	0.077	0.133
WExT	0.667	0.080	0.143	0.667	0.080	0.143

(b) Metrics for BRCA data. (1026 samples | 7 CGC-CGC pairs)

Method	Precision	Sensitivity	F1 Score	$\text{Precision}_{\text{strict}}$	$\text{Sensitivity}_{\text{strict}}$	F1 $_{\text{strict}}$ Score
DISCOVER	0.333	0.143	0.200	0.5	0.143	0.222
DISCOVER Strat	0.500	0.143	0.222	1.0	0.143	0.250
Fisher's Exact Test	NaN	0.000	NaN	NaN	0.000	NaN
MEGSA	NaN	0.000	NaN	NaN	0.000	NaN
MEMO	0.333	0.143	0.200	0.5	0.143	0.222
WExT	0.333	0.143	0.200	0.5	0.143	0.222

(c) Metrics for COADREAD data. (498 samples | 116 CGC-CGC pairs)

Method	Precision	Sensitivity	F1 Score	$\text{Precision}_{\text{strict}}$	$\text{Sensitivity}_{\text{strict}}$	F1 $_{\text{strict}}$ Score
DISCOVER	0.581	0.218	0.317	0.767	0.201	0.319
DISCOVER Strat	0.650	0.113	0.192	0.722	0.113	0.195
Fisher's Exact Test	1.000	0.096	0.175	1.000	0.096	0.175
MEGSA	1.000	0.113	0.203	1.000	0.113	0.203
MEMO	0.551	0.237	0.331	0.469	0.132	0.206
WExT	0.547	0.252	0.345	0.714	0.217	0.333

(d) Metrics for LUAD data. (568 samples | 49 CGC-CGC pairs)

Method	Precision	Sensitivity	F1 Score	$\text{Precision}_{\text{strict}}$	$\text{Sensitivity}_{\text{strict}}$	F1 $_{\text{strict}}$ Score
DISCOVER	0.750	0.184	0.295	0.818	0.184	0.300
Fisher's Exact Test	1.000	0.102	0.185	1.000	0.102	0.185
MEGSA	0.833	0.102	0.182	0.833	0.102	0.182
MEMO	0.588	0.208	0.308	0.769	0.208	0.327
WExT	0.650	0.265	0.377	0.812	0.265	0.400

(e) Metrics for LUSC data. (485 samples | 16 CGC-CGC pairs)

Method	Precision	Sensitivity	F1 Score	$\text{Precision}_{\text{strict}}$	$\text{Sensitivity}_{\text{strict}}$	F1 $_{\text{strict}}$ Score
DISCOVER	NaN	0.000	NaN	NaN	0.000	NaN
Fisher's Exact Test	NaN	0.000	NaN	NaN	0.000	NaN
MEGSA	NaN	0.000	NaN	NaN	0.000	NaN
MEMO	NaN	0.000	NaN	NaN	0.000	NaN
WExT	1.0	0.062	0.118	1.0	0.062	0.117

(f) Metrics for SKCM data. (468 samples | 373 CGC-CGC pairs)

Method	Precision	Sensitivity	F1 Score	$\text{Precision}_{\text{strict}}$	$\text{Sensitivity}_{\text{strict}}$	F1 $_{\text{strict}}$ Score
DISCOVER	0.717	0.052	0.096	0.735	0.049	0.092
Fisher's Exact Test	1.000	0.005	0.011	1.000	0.005	0.010
MEGSA	0.727	0.021	0.042	0.727	0.021	0.041
WExT	0.708	0.125	0.213	0.741	0.117	0.202

(g) Metrics for STAD data. (438 samples | 48 CGC-CGC pairs)

Method	Precision	Sensitivity	F1 Score	$\text{Precision}_{\text{strict}}$	$\text{Sensitivity}_{\text{strict}}$	F1 $_{\text{strict}}$ Score
DISCOVER	1.0	0.062	0.118	1.0	0.062	0.117
Fisher's Exact Test	NaN	0.000	NaN	NaN	0.000	NaN
MEGSA	NaN	0.000	NaN	NaN	0.000	NaN
WExT	0.8	0.083	0.151	0.8	0.083	0.150

(h) Metrics for UCEC data. (531 samples | 1440 CGC-CGC pairs)

Method	Precision	Sensitivity	F1 Score	$\text{Precision}_{\text{strict}}$	$\text{Sensitivity}_{\text{strict}}$	F1 $_{\text{strict}}$ Score
DISCOVER	0.677	0.172	0.274	0.776	0.148	0.249
Fisher's Exact Test	0.909	0.007	0.014	1.000	0.007	0.014
MEGSA	0.917	0.008	0.015	1.000	0.008	0.016
WExT	0.629	0.282	0.389	0.715	0.235	0.354

Table 24: Degree-normalized network-centric evaluations X_2 and $t = 20$

(a) Metrics for BLCA data.

Method	Precision	Sensitivity	F1 Score	$\text{Precision}_{\text{strict}}$	$\text{Sensitivity}_{\text{strict}}$	F1 $_{\text{strict}}$ Score
DISCOVER	1.0	1.0	0.625	1.0	0.571	0.625
Fisher's Exact Test	1.0	1.0	0.25	1.0	0.143	0.25
MEGSA	1.0	1.0	0.625	1.0	0.571	0.625
MEMO	0.75	0.929	0.611	0.75	0.571	0.611
WExT	0.667	0.857	0.6	0.667	0.571	0.6

(b) Metrics for BRCA data.

Method	Precision	Sensitivity	F1 Score	$\text{Precision}_{\text{strict}}$	$\text{Sensitivity}_{\text{strict}}$	F1 $_{\text{strict}}$ Score
DISCOVER	0.733	0.7	0.693	0.8	0.7	0.733
DISCOVER Strat	0.8	0.8	0.733	0.8	0.7	0.733
Fisher's Exact Test	NaN	NaN	NaN	NaN	NaN	NaN
MEGSA	NaN	NaN	NaN	NaN	NaN	NaN
MEMO	0.733	0.7	0.693	0.8	0.7	0.733
WExT	0.733	0.7	0.693	0.8	0.7	0.733

(c) Metrics for COADREAD data.

Method	Precision	Sensitivity	F1 Score	$\text{Precision}_{\text{strict}}$	$\text{Sensitivity}_{\text{strict}}$	F1 $_{\text{strict}}$ Score
DISCOVER	0.64	0.528	0.364	0.667	0.481	0.519
DISCOVER Strat	0.525	0.562	0.3	0.5	0.231	0.281
Fisher's Exact Test	0.475	0.728	0.286	0.475	0.206	0.286
MEGSA	0.777	0.853	0.658	0.753	0.577	0.635
MEMO	0.683	0.581	0.473	0.677	0.539	0.57
WExT	0.728	0.603	0.466	0.722	0.57	0.603

(d) Metrics for LUAD data.

Method	Precision	Sensitivity	F1 Score	$\text{Precision}_{\text{strict}}$	$\text{Sensitivity}_{\text{strict}}$	F1 $_{\text{strict}}$ Score
DISCOVER	0.643	0.702	0.486	0.643	0.429	0.471
Fisher's Exact Test	NaN	NaN	NaN	NaN	NaN	NaN
MEGSA	0.375	0.625	0.162	0.375	0.104	0.162
MEMO	0.75	0.792	0.539	0.75	0.471	0.529
WExT	0.682	0.796	0.507	0.673	0.43	0.488

(e) Metrics for LUSC data.

Method	Precision	Sensitivity	F1 Score	$\text{Precision}_{\text{strict}}$	$\text{Sensitivity}_{\text{strict}}$	F1 $_{\text{strict}}$ Score
DISCOVER	NaN	NaN	NaN	NaN	NaN	NaN
Fisher's Exact Test	NaN	NaN	NaN	NaN	NaN	NaN
MEGSA	1.0	1.0	0.778	1.0	0.667	0.778
MEMO	1.0	1.0	0.833	1.0	0.75	0.833
WExT	0.667	0.5	0.5	0.75	0.75	0.75

(f) Metrics for SKCM data.

Method	Precision	Sensitivity	F1 Score	$\text{Precision}_{\text{strict}}$	$\text{Sensitivity}_{\text{strict}}$	F1 $_{\text{strict}}$ Score
DISCOVER	0.812	0.971	0.467	0.812	0.375	0.467
Fisher's Exact Test	1.0	1.0	0.583	1.0	0.417	0.583
MEGSA	0.857	0.99	0.495	0.857	0.381	0.495
WExT	0.763	0.883	0.447	0.775	0.414	0.482

(g) Metrics for STAD data.

Method	Precision	Sensitivity	F1 Score	$\text{Precision}_{\text{strict}}$	$\text{Sensitivity}_{\text{strict}}$	F1 $_{\text{strict}}$ Score
DISCOVER	0.75	0.847	0.442	0.773	0.39	0.445
Fisher's Exact Test	NaN	NaN	NaN	NaN	NaN	NaN
MEGSA	1.0	1.0	0.286	1.0	0.167	0.286
WExT	0.9	0.864	0.528	0.907	0.529	0.588

(h) Metrics for UCEC data.

Method	Precision	Sensitivity	F1 Score	$\text{Precision}_{\text{strict}}$	$\text{Sensitivity}_{\text{strict}}$	F1 $_{\text{strict}}$ Score
DISCOVER	0.896	0.894	0.457	0.913	0.355	0.454
Fisher's Exact Test	0.952	0.988	0.211	0.952	0.127	0.211
MEGSA	0.946	0.983	0.268	0.946	0.174	0.268
WExT	0.74	0.79	0.536	0.753	0.401	0.481

Table 25: Results of network-centric ME evaluation framework with $\mathcal{G}$ = Intact (w conf. threshold 0.35), $\mathcal{S}$ = CGC, c= X_1, p_t = 0.05, t=5, robustness_iterations = 100

(a) Metrics for BLCA data. (411 samples | 1048 CGC-CGC pairs)

Method	Precision	Sensitivity	F1 Score	$\text{Precision}_{\text{strict}}$	$\text{Sensitivity}_{\text{strict}}$	F1 $_{\text{strict}}$ Score
DISCOVER	0.923	0.012	0.023	0.923	0.012	0.024
Fisher's Exact Test	1.000	0.002	0.004	1.000	0.002	0.004
WExT	0.690	0.019	0.037	0.690	0.019	0.037

(b) Metrics for BRCA data. (1026 samples | 958 CGC-CGC pairs)

Method	Precision	Sensitivity	F1 Score	$\text{Precision}_{\text{strict}}$	$\text{Sensitivity}_{\text{strict}}$	F1 $_{\text{strict}}$ Score
DISCOVER	0.727	0.025	0.049	0.727	0.025	0.048
DISCOVER Strat	0.789	0.031	0.060	0.789	0.031	0.060
Fisher's Exact Test	0.667	0.002	0.004	0.667	0.002	0.004
WExT	0.691	0.059	0.109	0.707	0.056	0.104

(c) Metrics for COADREAD data. (498 samples | 1748 CGC-CGC pairs)

Method	Precision	Sensitivity	F1 Score	$\text{Precision}_{\text{strict}}$	$\text{Sensitivity}_{\text{strict}}$	F1 $_{\text{strict}}$ Score
DISCOVER	0.647	0.052	0.096	0.658	0.046	0.086
DISCOVER Strat	0.618	0.012	0.024	0.618	0.012	0.024
Fisher's Exact Test	0.583	0.008	0.016	0.565	0.007	0.014
WExT	0.645	0.121	0.203	0.668	0.102	0.177

(d) Metrics for LUAD data. (568 samples | 1344 CGC-CGC pairs)

Method	Precision	Sensitivity	F1 Score	$\text{Precision}_{\text{strict}}$	$\text{Sensitivity}_{\text{strict}}$	F1 $_{\text{strict}}$ Score
DISCOVER	0.769	0.015	0.029	0.760	0.014	0.027
Fisher's Exact Test	0.000	0.000	NaN	0.000	0.000	NaN
WExT	0.788	0.031	0.060	0.812	0.029	0.056

(e) Metrics for LUSC data. (485 samples | 1080 CGC-CGC pairs)

Method	Precision	Sensitivity	F1 Score	$\text{Precision}_{\text{strict}}$	$\text{Sensitivity}_{\text{strict}}$	F1 $_{\text{strict}}$ Score
DISCOVER	1.000	0.002	0.004	1.000	0.002	0.004
Fisher's Exact Test	1.000	0.002	0.004	1.000	0.002	0.004
WExT	0.857	0.006	0.011	0.857	0.006	0.012

(f) Metrics for SKCM data. (468 samples | 2254 CGC-CGC pairs)

Method	Precision	Sensitivity	F1 Score	$\text{Precision}_{\text{strict}}$	$\text{Sensitivity}_{\text{strict}}$	F1 $_{\text{strict}}$ Score
DISCOVER	0.838	0.014	0.027	0.838	0.014	0.028
Fisher's Exact Test	1.000	0.001	0.002	1.000	0.001	0.002
WExT	0.725	0.042	0.080	0.732	0.041	0.078

(g) Metrics for STAD data. (438 samples | 1460 CGC-CGC pairs)

Method	Precision	Sensitivity	F1 Score	$\text{Precision}_{\text{strict}}$	$\text{Sensitivity}_{\text{strict}}$	F1 $_{\text{strict}}$ Score
DISCOVER	0.667	0.028	0.053	0.673	0.025	0.048
Fisher's Exact Test	0.667	0.003	0.005	0.667	0.003	0.006
WExT	0.688	0.069	0.125	0.705	0.060	0.111

(h) Metrics for UCEC data. (531 samples | 2274 CGC-CGC pairs)

Method	Precision	Sensitivity	F1 Score	$\text{Precision}_{\text{strict}}$	$\text{Sensitivity}_{\text{strict}}$	F1 $_{\text{strict}}$ Score
DISCOVER	0.666	0.129	0.216	0.711	0.111	0.192
Fisher's Exact Test	0.833	0.004	0.007	0.833	0.004	0.008
WExT	0.626	0.210	0.314	0.674	0.178	0.282

Table 26: Results of network-centric ME evaluation framework with $\mathcal{G}$ = Intact (w conf. threshold 0.35), $\mathcal{S}$ = CGC, c= X_1, p_t = 0.05, t=5, robustness_iterations = 300

(a) Metrics for BLCA data. (411 samples | 1048 CGC-CGC pairs)

Method	Precision	Sensitivity	F1 Score	$\text{Precision}_{\text{strict}}$	$\text{Sensitivity}_{\text{strict}}$	F1 $_{\text{strict}}$ Score
DISCOVER	0.923	0.011	0.023	0.923	0.011	0.022
Fisher's Exact Test	1.000	0.002	0.004	1.000	0.002	0.004
WExT	0.690	0.019	0.037	0.690	0.019	0.037

(b) Metrics for BRCA data. (1026 samples | 958 CGC-CGC pairs)

Method	Precision	Sensitivity	F1 Score	$\text{Precision}_{\text{strict}}$	$\text{Sensitivity}_{\text{strict}}$	F1 $_{\text{strict}}$ Score
DISCOVER	0.742	0.024	0.047	0.742	0.024	0.046
DISCOVER Strat	0.789	0.032	0.061	0.789	0.032	0.062
Fisher's Exact Test	0.667	0.002	0.004	0.667	0.002	0.004
WExT	0.705	0.058	0.107	0.726	0.056	0.104

(c) Metrics for COADREAD data. (498 samples | 1748 CGC-CGC pairs)

Method	Precision	Sensitivity	F1 Score	$\text{Precision}_{\text{strict}}$	$\text{Sensitivity}_{\text{strict}}$	F1 $_{\text{strict}}$ Score
DISCOVER	0.645	0.052	0.096	0.658	0.046	0.086
DISCOVER Strat	0.636	0.012	0.024	0.636	0.012	0.024
Fisher's Exact Test	0.583	0.008	0.016	0.565	0.007	0.014
WExT	0.644	0.120	0.202	0.669	0.102	0.177

(d) Metrics for LUAD data. (568 samples | 1344 CGC-CGC pairs)

Method	Precision	Sensitivity	F1 Score	$\text{Precision}_{\text{strict}}$	$\text{Sensitivity}_{\text{strict}}$	F1 $_{\text{strict}}$ Score
DISCOVER	0.800	0.015	0.029	0.792	0.014	0.028
Fisher's Exact Test	0.000	0.000	NaN	0.000	0.000	NaN
WExT	0.769	0.030	0.058	0.776	0.029	0.056

(e) Metrics for LUSC data. (485 samples | 1080 CGC-CGC pairs)

Method	Precision	Sensitivity	F1 Score	$\text{Precision}_{\text{strict}}$	$\text{Sensitivity}_{\text{strict}}$	F1 $_{\text{strict}}$ Score
DISCOVER	1.000	0.002	0.004	1.000	0.002	0.004
Fisher's Exact Test	1.000	0.002	0.004	1.000	0.002	0.004
WExT	0.857	0.006	0.011	0.857	0.006	0.012

(f) Metrics for SKCM data. (468 samples | 2254 CGC-CGC pairs)

Method	Precision	Sensitivity	F1 Score	$\text{Precision}_{\text{strict}}$	$\text{Sensitivity}_{\text{strict}}$	F1 $_{\text{strict}}$ Score
DISCOVER	0.800	0.014	0.028	0.800	0.014	0.028
Fisher's Exact Test	1.000	0.001	0.002	1.000	0.001	0.002
WExT	0.728	0.042	0.080	0.735	0.041	0.078

(g) Metrics for STAD data. (438 samples | 1460 CGC-CGC pairs)

Method	Precision	Sensitivity	F1 Score	$\text{Precision}_{\text{strict}}$	$\text{Sensitivity}_{\text{strict}}$	F1 $_{\text{strict}}$ Score
DISCOVER	0.667	0.028	0.053	0.673	0.026	0.050
Fisher's Exact Test	0.667	0.003	0.005	0.667	0.003	0.006
WExT	0.688	0.069	0.125	0.705	0.060	0.111

(h) Metrics for UCEC data. (531 samples | 2274 CGC-CGC pairs)

Method	Precision	Sensitivity	F1 Score	$\text{Precision}_{\text{strict}}$	$\text{Sensitivity}_{\text{strict}}$	F1 $_{\text{strict}}$ Score
DISCOVER	0.662	0.129	0.216	0.706	0.111	0.192
Fisher's Exact Test	0.769	0.004	0.007	0.769	0.004	0.008
WExT	0.623	0.211	0.315	0.670	0.178	0.281

Table 27: Results of network-centric ME evaluation framework with $\mathcal{G}$ = Intact (w conf. threshold 0.35), $\mathcal{S}$ = CGC, c= X_1, p_t = 0.05, t=5, robustness_iterations = 500

(a) Metrics for BLCA data. (411 samples | 1048 CGC-CGC pairs)

Method	Precision	Sensitivity	F1 Score	$\text{Precision}_{\text{strict}}$	$\text{Sensitivity}_{\text{strict}}$	F1 $_{\text{strict}}$ Score
DISCOVER	0.923	0.012	0.023	0.923	0.012	0.024
Fisher's Exact Test	1.000	0.002	0.004	1.000	0.002	0.004
WExT	0.690	0.019	0.037	0.690	0.019	0.037

(b) Metrics for BRCA data. (1026 samples | 958 CGC-CGC pairs)

Method	Precision	Sensitivity	F1 Score	$\text{Precision}_{\text{strict}}$	$\text{Sensitivity}_{\text{strict}}$	F1 $_{\text{strict}}$ Score
DISCOVER	0.719	0.024	0.047	0.719	0.024	0.046
DISCOVER Strat	0.789	0.031	0.060	0.789	0.031	0.060
Fisher's Exact Test	0.667	0.002	0.004	0.667	0.002	0.004
WExT	0.705	0.058	0.107	0.726	0.056	0.104

(c) Metrics for COADREAD data. (498 samples | 1748 CGC-CGC pairs)

Method	Precision	Sensitivity	F1 Score	$\text{Precision}_{\text{strict}}$	$\text{Sensitivity}_{\text{strict}}$	F1 $_{\text{strict}}$ Score
DISCOVER	0.643	0.052	0.097	0.653	0.046	0.086
DISCOVER Strat	0.600	0.012	0.024	0.600	0.012	0.024
Fisher's Exact Test	0.591	0.007	0.015	0.591	0.007	0.014
WExT	0.652	0.120	0.202	0.677	0.102	0.177

(d) Metrics for LUAD data. (568 samples | 1344 CGC-CGC pairs)

Method	Precision	Sensitivity	F1 Score	$\text{Precision}_{\text{strict}}$	$\text{Sensitivity}_{\text{strict}}$	F1 $_{\text{strict}}$ Score
DISCOVER	0.800	0.015	0.029	0.792	0.014	0.028
Fisher's Exact Test	0.000	0.000	NaN	0.000	0.000	NaN
WExT	0.784	0.030	0.058	0.792	0.029	0.056

(e) Metrics for LUSC data. (485 samples | 1080 CGC-CGC pairs)

Method	Precision	Sensitivity	F1 Score	$\text{Precision}_{\text{strict}}$	$\text{Sensitivity}_{\text{strict}}$	F1 $_{\text{strict}}$ Score
DISCOVER	1.000	0.002	0.004	1.000	0.002	0.004
Fisher's Exact Test	1.000	0.002	0.004	1.000	0.002	0.004
WExT	0.857	0.006	0.011	0.857	0.006	0.012

(f) Metrics for SKCM data. (468 samples | 2254 CGC-CGC pairs)

Method	Precision	Sensitivity	F1 Score	$\text{Precision}_{\text{strict}}$	$\text{Sensitivity}_{\text{strict}}$	F1 $_{\text{strict}}$ Score
DISCOVER	0.821	0.014	0.028	0.821	0.014	0.028
Fisher's Exact Test	1.000	0.001	0.002	1.000	0.001	0.002
WExT	0.723	0.043	0.080	0.730	0.042	0.079

(g) Metrics for STAD data. (438 samples | 1460 CGC-CGC pairs)

Method	Precision	Sensitivity	F1 Score	$\text{Precision}_{\text{strict}}$	$\text{Sensitivity}_{\text{strict}}$	F1 $_{\text{strict}}$ Score
DISCOVER	0.667	0.028	0.053	0.673	0.026	0.050
Fisher's Exact Test	0.667	0.003	0.005	0.667	0.003	0.006
WExT	0.692	0.069	0.125	0.711	0.060	0.111

(h) Metrics for UCEC data. (531 samples | 2274 CGC-CGC pairs)

Method	Precision	Sensitivity	F1 Score	$\text{Precision}_{\text{strict}}$	$\text{Sensitivity}_{\text{strict}}$	F1 $_{\text{strict}}$ Score
DISCOVER	0.661	0.128	0.215	0.704	0.110	0.190
Fisher's Exact Test	0.769	0.004	0.007	0.769	0.004	0.008
WExT	0.628	0.213	0.318	0.675	0.180	0.284

Table 28: Results of network-centric ME evaluation framework with $\mathcal{G}$ = Intact (w conf. threshold 0.35), $\mathcal{S}$ = CGC, c= X_1, p_t = 0.01, t=5, robustness_iterations = 100

(a) Metrics for BLCA data. (411 samples | 1048 CGC-CGC pairs)

Method	Precision	Sensitivity	F1 Score	$\text{Precision}_{\text{strict}}$	$\text{Sensitivity}_{\text{strict}}$	F1 $_{\text{strict}}$ Score
DISCOVER	1.0	0.002	0.004	1.0	0.002	0.004
Fisher's Exact Test	NaN	0.000	NaN	NaN	0.000	NaN
WExT	0.8	0.004	0.008	0.8	0.004	0.008

(b) Metrics for BRCA data. (1026 samples | 958 CGC-CGC pairs)

Method	Precision	Sensitivity	F1 Score	$\text{Precision}_{\text{strict}}$	$\text{Sensitivity}_{\text{strict}}$	F1 $_{\text{strict}}$ Score
DISCOVER	0.667	0.006	0.012	0.667	0.006	0.012
DISCOVER Strat	0.750	0.006	0.012	0.750	0.006	0.012
Fisher's Exact Test	NaN	0.000	NaN	NaN	0.000	NaN
WExT	0.556	0.011	0.021	0.556	0.011	0.022

(c) Metrics for COADREAD data. (498 samples | 1748 CGC-CGC pairs)

Method	Precision	Sensitivity	F1 Score	$\text{Precision}_{\text{strict}}$	$\text{Sensitivity}_{\text{strict}}$	F1 $_{\text{strict}}$ Score
DISCOVER	0.673	0.020	0.038	0.674	0.018	0.035
DISCOVER Strat	0.000	0.000	NaN	0.000	0.000	NaN
Fisher's Exact Test	0.400	0.001	0.002	0.400	0.001	0.002
WExT	0.678	0.069	0.125	0.703	0.055	0.102

(d) Metrics for LUAD data. (568 samples | 1344 CGC-CGC pairs)

Method	Precision	Sensitivity	F1 Score	$\text{Precision}_{\text{strict}}$	$\text{Sensitivity}_{\text{strict}}$	F1 $_{\text{strict}}$ Score
DISCOVER	0.667	0.003	0.006	0.667	0.003	0.006
Fisher's Exact Test	NaN	0.000	NaN	NaN	0.000	NaN
WExT	0.829	0.013	0.025	0.829	0.013	0.026

(e) Metrics for LUSC data. (485 samples | 1080 CGC-CGC pairs)

Method	Precision	Sensitivity	F1 Score	$\text{Precision}_{\text{strict}}$	$\text{Sensitivity}_{\text{strict}}$	F1 $_{\text{strict}}$ Score
DISCOVER	1.0	0.002	0.004	1.0	0.002	0.004
Fisher's Exact Test	NaN	0.000	NaN	NaN	0.000	NaN
WExT	1.0	0.002	0.004	1.0	0.002	0.004

(f) Metrics for SKCM data. (468 samples | 2254 CGC-CGC pairs)

Method	Precision	Sensitivity	F1 Score	$\text{Precision}_{\text{strict}}$	$\text{Sensitivity}_{\text{strict}}$	F1 $_{\text{strict}}$ Score
DISCOVER	0.750	0.003	0.005	0.750	0.003	0.006
Fisher's Exact Test	1.000	0.001	0.002	1.000	0.001	0.002
WExT	0.792	0.008	0.017	0.792	0.008	0.016

(g) Metrics for STAD data. (438 samples | 1460 CGC-CGC pairs)

Method	Precision	Sensitivity	F1 Score	$\text{Precision}_{\text{strict}}$	$\text{Sensitivity}_{\text{strict}}$	F1 $_{\text{strict}}$ Score
DISCOVER	0.615	0.008	0.016	0.595	0.008	0.016
Fisher's Exact Test	0.800	0.001	0.003	0.800	0.001	0.002
WExT	0.631	0.028	0.054	0.654	0.023	0.044

(h) Metrics for UCEC data. (531 samples | 2274 CGC-CGC pairs)

Method	Precision	Sensitivity	F1 Score	$\text{Precision}_{\text{strict}}$	$\text{Sensitivity}_{\text{strict}}$	F1 $_{\text{strict}}$ Score
DISCOVER	0.676	0.072	0.129	0.721	0.063	0.116
Fisher's Exact Test	0.857	0.002	0.004	0.857	0.002	0.004
WExT	0.650	0.136	0.224	0.710	0.116	0.199

Table 29: Results of network-centric ME evaluation framework with $\mathcal{G}$ = Intact (w conf. threshold 0.35), $\mathcal{S}$ = CGC, c= X_1, p_t = 0.1, t=5, robustness_iterations = 100

(a) Metrics for BLCA data. (411 samples | 1048 CGC-CGC pairs)

Method	Precision	Sensitivity	F1 Score	$\text{Precision}_{\text{strict}}$	$\text{Sensitivity}_{\text{strict}}$	F1 $_{\text{strict}}$ Score
DISCOVER	0.769	0.019	0.038	0.769	0.019	0.037
Fisher's Exact Test	1.000	0.002	0.004	1.000	0.002	0.004
WExT	0.746	0.047	0.089	0.742	0.046	0.087

(b) Metrics for BRCA data. (1026 samples | 958 CGC-CGC pairs)

Method	Precision	Sensitivity	F1 Score	$\text{Precision}_{\text{strict}}$	$\text{Sensitivity}_{\text{strict}}$	F1 $_{\text{strict}}$ Score
DISCOVER	0.696	0.051	0.094	0.697	0.049	0.092
DISCOVER Strat	0.728	0.056	0.105	0.741	0.054	0.101
Fisher's Exact Test	0.667	0.002	0.004	0.667	0.002	0.004
WExT	0.670	0.109	0.187	0.695	0.099	0.173

(c) Metrics for COADREAD data. (498 samples | 1748 CGC-CGC pairs)

Method	Precision	Sensitivity	F1 Score	$\text{Precision}_{\text{strict}}$	$\text{Sensitivity}_{\text{strict}}$	F1 $_{\text{strict}}$ Score
DISCOVER	0.647	0.089	0.156	0.662	0.079	0.141
DISCOVER Strat	0.663	0.039	0.074	0.660	0.038	0.072
Fisher's Exact Test	0.656	0.017	0.034	0.646	0.015	0.029
WExT	0.648	0.191	0.295	0.672	0.166	0.266

(d) Metrics for LUAD data. (568 samples | 1344 CGC-CGC pairs)

Method	Precision	Sensitivity	F1 Score	$\text{Precision}_{\text{strict}}$	$\text{Sensitivity}_{\text{strict}}$	F1 $_{\text{strict}}$ Score
DISCOVER	0.764	0.031	0.060	0.784	0.030	0.058
Fisher's Exact Test	0.750	0.004	0.009	0.750	0.004	0.008
WExT	0.737	0.057	0.106	0.757	0.052	0.097

(e) Metrics for LUSC data. (485 samples | 1080 CGC-CGC pairs)

Method	Precision	Sensitivity	F1 Score	$\text{Precision}_{\text{strict}}$	$\text{Sensitivity}_{\text{strict}}$	F1 $_{\text{strict}}$ Score
DISCOVER	1.0	0.004	0.007	1.0	0.004	0.008
Fisher's Exact Test	1.0	0.002	0.004	1.0	0.002	0.004
WExT	0.7	0.013	0.026	0.7	0.013	0.026

(f) Metrics for SKCM data. (468 samples | 2254 CGC-CGC pairs)

Method	Precision	Sensitivity	F1 Score	$\text{Precision}_{\text{strict}}$	$\text{Sensitivity}_{\text{strict}}$	F1 $_{\text{strict}}$ Score
DISCOVER	0.763	0.039	0.075	0.768	0.039	0.074
Fisher's Exact Test	1.000	0.002	0.004	1.000	0.002	0.004
WExT	0.662	0.080	0.144	0.666	0.078	0.140

(g) Metrics for STAD data. (438 samples | 1460 CGC-CGC pairs)

Method	Precision	Sensitivity	F1 Score	$\text{Precision}_{\text{strict}}$	$\text{Sensitivity}_{\text{strict}}$	F1 $_{\text{strict}}$ Score
DISCOVER	0.698	0.052	0.097	0.715	0.048	0.090
Fisher's Exact Test	0.667	0.005	0.011	0.667	0.005	0.010
WExT	0.639	0.093	0.163	0.652	0.081	0.144

(h) Metrics for UCEC data. (531 samples | 2274 CGC-CGC pairs)

Method	Precision	Sensitivity	F1 Score	$\text{Precision}_{\text{strict}}$	$\text{Sensitivity}_{\text{strict}}$	F1 $_{\text{strict}}$ Score
DISCOVER	0.640	0.174	0.274	0.684	0.148	0.243
Fisher's Exact Test	0.704	0.007	0.014	0.720	0.006	0.012
WExT	0.599	0.273	0.375	0.640	0.230	0.338

Table 30: Results of network-centric ME evaluation framework with $\mathcal{G}$ = Intact (w conf. threshold 0.35), $\mathcal{S}$ = CGC_{SNV} , c= X_1, p_t = 0.05, t=5, robustness_iterations = 100

(a) Metrics for BLCA data. (411 samples | 363 CGC_{SNV}-CGC_{SNV} pairs)

Method	Precision	Sensitivity	F1 Score	$\text{Precision}_{\text{strict}}$	$\text{Sensitivity}_{\text{strict}}$	F1 $_{\text{strict}}$ Score
DISCOVER	0.889	0.013	0.025	0.889	0.013	0.026
Fisher's Exact Test	1.000	0.003	0.006	1.000	0.003	0.006
WExT	0.667	0.022	0.043	0.667	0.022	0.043

(b) Metrics for BRCA data. (1026 samples | 568 CGC_{SNV}-CGC_{SNV} pairs)

Method	Precision	Sensitivity	F1 Score	$\text{Precision}_{\text{strict}}$	$\text{Sensitivity}_{\text{strict}}$	F1 $_{\text{strict}}$ Score
DISCOVER	0.653	0.029	0.055	0.653	0.029	0.056
DISCOVER Strat	0.759	0.039	0.074	0.759	0.039	0.074
Fisher's Exact Test	0.667	0.004	0.007	0.667	0.004	0.008
WExT	0.672	0.080	0.144	0.694	0.077	0.139

(c) Metrics for COADREAD data. (498 samples | 636 CGC_{SNV}-CGC_{SNV} pairs)

Method	Precision	Sensitivity	F1 Score	$\text{Precision}_{\text{strict}}$	$\text{Sensitivity}_{\text{strict}}$	F1 $_{\text{strict}}$ Score
DISCOVER	0.621	0.065	0.117	0.637	0.057	0.105
DISCOVER Strat	0.704	0.020	0.039	0.704	0.020	0.039
Fisher's Exact Test	0.632	0.012	0.025	0.611	0.011	0.022
WExT	0.608	0.147	0.237	0.622	0.123	0.205

(d) Metrics for LUAD data. (568 samples | 694 CGC_{SNV}-CGC_{SNV} pairs)

Method	Precision	Sensitivity	F1 Score	$\text{Precision}_{\text{strict}}$	$\text{Sensitivity}_{\text{strict}}$	F1 $_{\text{strict}}$ Score
DISCOVER	0.737	0.020	0.040	0.722	0.019	0.037
Fisher's Exact Test	0.000	0.000	NaN	0.000	0.000	NaN
WExT	0.780	0.047	0.089	0.789	0.044	0.083

(e) Metrics for LUSC data. (485 samples | 646 CGC_{SNV}-CGC_{SNV} pairs)

Method	Precision	Sensitivity	F1 Score	$\text{Precision}_{\text{strict}}$	$\text{Sensitivity}_{\text{strict}}$	F1 $_{\text{strict}}$ Score
DISCOVER	1.000	0.003	0.006	1.000	0.003	0.006
Fisher's Exact Test	1.000	0.003	0.006	1.000	0.003	0.006
WExT	0.857	0.009	0.019	0.857	0.009	0.018

(f) Metrics for SKCM data. (468 samples | 1144 CGC_{SNV}-CGC_{SNV} pairs)

Method	Precision	Sensitivity	F1 Score	$\text{Precision}_{\text{strict}}$	$\text{Sensitivity}_{\text{strict}}$	F1 $_{\text{strict}}$ Score
DISCOVER	0.640	0.014	0.028	0.640	0.014	0.027
Fisher's Exact Test	1.000	0.002	0.003	1.000	0.002	0.004
WExT	0.677	0.057	0.105	0.682	0.054	0.100

(g) Metrics for STAD data. (438 samples | 800 CGC_{SNV}-CGC_{SNV} pairs)

Method	Precision	Sensitivity	F1 Score	$\text{Precision}_{\text{strict}}$	$\text{Sensitivity}_{\text{strict}}$	F1 $_{\text{strict}}$ Score
DISCOVER	0.615	0.030	0.058	0.629	0.028	0.054
Fisher's Exact Test	0.667	0.003	0.005	0.667	0.003	0.006
WExT	0.667	0.080	0.143	0.692	0.069	0.125

(h) Metrics for UCEC data. (531 samples | 1294 CGC_{SNV}-CGC_{SNV} pairs)

Method	Precision	Sensitivity	F1 Score	$\text{Precision}_{\text{strict}}$	$\text{Sensitivity}_{\text{strict}}$	F1 $_{\text{strict}}$ Score
DISCOVER	0.657	0.180	0.283	0.696	0.152	0.250
Fisher's Exact Test	0.833	0.008	0.015	0.833	0.008	0.016
WExT	0.627	0.291	0.397	0.675	0.242	0.356

Table 31: Results of network-centric ME evaluation framework with $\mathcal{G}$ = Intact (w conf. threshold 0.35), $\mathcal{S}$ = Intogen , c= X_1, p_t = 0.05, t=5, robustness_iterations = 100

(a) Metrics for BLCA data. (411 samples | 968 Intogen-Intogen pairs)

Method	Precision	Sensitivity	F1 Score	$\text{Precision}_{\text{strict}}$	$\text{Sensitivity}_{\text{strict}}$	F1 $_{\text{strict}}$ Score
DISCOVER	0.941	0.017	0.033	0.941	0.017	0.033
Fisher's Exact Test	1.000	0.002	0.004	1.000	0.002	0.004
WExT	0.733	0.023	0.045	0.733	0.023	0.045

(b) Metrics for BRCA data. (1026 samples | 908 Intogen-Intogen pairs)

Method	Precision	Sensitivity	F1 Score	$\text{Precision}_{\text{strict}}$	$\text{Sensitivity}_{\text{strict}}$	F1 $_{\text{strict}}$ Score
DISCOVER	0.711	0.030	0.057	0.711	0.030	0.058
DISCOVER Strat	0.780	0.035	0.068	0.780	0.035	0.067
Fisher's Exact Test	0.667	0.002	0.004	0.667	0.002	0.004
WExT	0.693	0.068	0.124	0.704	0.064	0.117

(c) Metrics for COADREAD data. (498 samples | 1618 Intogen-Intogen pairs)

Method	Precision	Sensitivity	F1 Score	$\text{Precision}_{\text{strict}}$	$\text{Sensitivity}_{\text{strict}}$	F1 $_{\text{strict}}$ Score
DISCOVER	0.655	0.068	0.124	0.669	0.060	0.110
DISCOVER Strat	0.680	0.021	0.041	0.673	0.021	0.041
Fisher's Exact Test	0.558	0.009	0.018	0.551	0.008	0.016
WExT	0.644	0.145	0.237	0.661	0.122	0.206

(d) Metrics for LUAD data. (568 samples | 1164 Intogen-Intogen pairs)

Method	Precision	Sensitivity	F1 Score	$\text{Precision}_{\text{strict}}$	$\text{Sensitivity}_{\text{strict}}$	F1 $_{\text{strict}}$ Score
DISCOVER	0.720	0.016	0.030	0.708	0.015	0.029
Fisher's Exact Test	0.000	0.000	NaN	0.000	0.000	NaN
WExT	0.754	0.038	0.071	0.755	0.035	0.067

(e) Metrics for LUSC data. (485 samples | 996 Intogen-Intogen pairs)

Method	Precision	Sensitivity	F1 Score	$\text{Precision}_{\text{strict}}$	$\text{Sensitivity}_{\text{strict}}$	F1 $_{\text{strict}}$ Score
DISCOVER	1.000	0.004	0.008	1.000	0.004	0.008
Fisher's Exact Test	1.000	0.002	0.004	1.000	0.002	0.004
WExT	0.909	0.010	0.020	0.909	0.010	0.020

(f) Metrics for SKCM data. (468 samples | 1844 Intogen-Intogen pairs)

Method	Precision	Sensitivity	F1 Score	$\text{Precision}_{\text{strict}}$	$\text{Sensitivity}_{\text{strict}}$	F1 $_{\text{strict}}$ Score
DISCOVER	0.691	0.015	0.030	0.691	0.015	0.029
Fisher's Exact Test	1.000	0.001	0.002	1.000	0.001	0.002
WExT	0.702	0.048	0.090	0.708	0.047	0.088

(g) Metrics for STAD data. (438 samples | 1398 Intogen-Intogen pairs)

Method	Precision	Sensitivity	F1 Score	$\text{Precision}_{\text{strict}}$	$\text{Sensitivity}_{\text{strict}}$	F1 $_{\text{strict}}$ Score
DISCOVER	0.648	0.033	0.063	0.656	0.030	0.057
Fisher's Exact Test	0.667	0.004	0.009	0.667	0.004	0.008
WExT	0.693	0.084	0.150	0.712	0.073	0.132

(h) Metrics for UCEC data. (531 samples | 2082 Intogen-Intogen pairs)

Method	Precision	Sensitivity	F1 Score	$\text{Precision}_{\text{strict}}$	$\text{Sensitivity}_{\text{strict}}$	F1 $_{\text{strict}}$ Score
DISCOVER	0.643	0.170	0.268	0.692	0.142	0.236
Fisher's Exact Test	0.765	0.006	0.012	0.765	0.006	0.012
WExT	0.602	0.258	0.361	0.645	0.212	0.319

Table 32: Results of network-centric ME evaluation framework with $\mathcal{G}$ = Intact (w conf. threshold 0.25), $\mathcal{S}$ = CGC, c= X_1, p_t = 0.05, t=5, robustness_iterations = 100

(a) Metrics for BLCA data. (411 samples | 1530 CGC-CGC pairs)

Method	Precision	Sensitivity	F1 Score	$\text{Precision}_{\text{strict}}$	$\text{Sensitivity}_{\text{strict}}$	F1 $_{\text{strict}}$ Score
DISCOVER	0.947	0.012	0.023	0.947	0.012	0.024
Fisher's Exact Test	1.000	0.004	0.008	1.000	0.004	0.008
WExT	0.732	0.020	0.039	0.732	0.020	0.039

(b) Metrics for BRCA data. (1026 samples | 1376 CGC-CGC pairs)

Method	Precision	Sensitivity	F1 Score	$\text{Precision}_{\text{strict}}$	$\text{Sensitivity}_{\text{strict}}$	F1 $_{\text{strict}}$ Score
DISCOVER	0.720	0.026	0.051	0.720	0.026	0.050
DISCOVER Strat	0.820	0.030	0.058	0.820	0.030	0.058
Fisher's Exact Test	0.800	0.003	0.006	0.800	0.003	0.006
WExT	0.707	0.058	0.106	0.727	0.055	0.102

(c) Metrics for COADREAD data. (498 samples | 2588 CGC-CGC pairs)

Method	Precision	Sensitivity	F1 Score	$\text{Precision}_{\text{strict}}$	$\text{Sensitivity}_{\text{strict}}$	F1 $_{\text{strict}}$ Score
DISCOVER	0.646	0.044	0.083	0.669	0.039	0.074
DISCOVER Strat	0.645	0.012	0.023	0.659	0.012	0.024
Fisher's Exact Test	0.611	0.009	0.017	0.618	0.008	0.016
WExT	0.652	0.107	0.183	0.676	0.092	0.162

(d) Metrics for LUAD data. (568 samples | 1948 CGC-CGC pairs)

Method	Precision	Sensitivity	F1 Score	$\text{Precision}_{\text{strict}}$	$\text{Sensitivity}_{\text{strict}}$	F1 $_{\text{strict}}$ Score
DISCOVER	0.775	0.016	0.031	0.789	0.015	0.029
Fisher's Exact Test	0.667	0.002	0.004	0.667	0.002	0.004
WExT	0.767	0.029	0.056	0.783	0.028	0.054

(e) Metrics for LUSC data. (485 samples | 1536 CGC-CGC pairs)

Method	Precision	Sensitivity	F1 Score	$\text{Precision}_{\text{strict}}$	$\text{Sensitivity}_{\text{strict}}$	F1 $_{\text{strict}}$ Score
DISCOVER	1.000	0.001	0.003	1.000	0.001	0.002
Fisher's Exact Test	1.000	0.001	0.003	1.000	0.001	0.002
WExT	0.727	0.005	0.010	0.727	0.005	0.010

(f) Metrics for SKCM data. (468 samples | 3208 CGC-CGC pairs)

Method	Precision	Sensitivity	F1 Score	$\text{Precision}_{\text{strict}}$	$\text{Sensitivity}_{\text{strict}}$	F1 $_{\text{strict}}$ Score
DISCOVER	0.774	0.013	0.025	0.774	0.013	0.026
Fisher's Exact Test	1.000	0.001	0.001	1.000	0.001	0.002
WExT	0.704	0.036	0.068	0.709	0.035	0.067

(g) Metrics for STAD data. (438 samples | 2286 CGC-CGC pairs)

Method	Precision	Sensitivity	F1 Score	$\text{Precision}_{\text{strict}}$	$\text{Sensitivity}_{\text{strict}}$	F1 $_{\text{strict}}$ Score
DISCOVER	0.685	0.028	0.053	0.707	0.026	0.050
Fisher's Exact Test	0.727	0.004	0.007	0.727	0.004	0.008
WExT	0.687	0.061	0.111	0.722	0.054	0.100

(h) Metrics for UCEC data. (531 samples | 4120 CGC-CGC pairs)

Method	Precision	Sensitivity	F1 Score	$\text{Precision}_{\text{strict}}$	$\text{Sensitivity}_{\text{strict}}$	F1 $_{\text{strict}}$ Score
DISCOVER	0.652	0.116	0.196	0.695	0.100	0.175
Fisher's Exact Test	0.737	0.003	0.007	0.737	0.003	0.006
WExT	0.618	0.202	0.305	0.654	0.172	0.272

Table 33: Results of network-centric ME evaluation framework with $\mathcal{G}$ = Intact (w conf. threshold 0.45), $\mathcal{S}$ = CGC, c= X_1, p_t = 0.05, t=5, robustness_iterations = 100

(a) Metrics for BLCA data. (411 samples | 468 CGC-CGC pairs)

Method	Precision	Sensitivity	F1 Score	Precision$_{strict}$	Sensitivity$_{strict}$	F1 $_{strict}$ Score
DISCOVER	1.000	0.013	0.026	1.000	0.013	0.026
Fisher's Exact Test	1.000	0.004	0.009	1.000	0.004	0.008
WExT	0.727	0.017	0.034	0.727	0.017	0.033

(b) Metrics for BRCA data. (1026 samples | 420 CGC-CGC pairs)

Method	Precision	Sensitivity	F1 Score	Precision$_{strict}$	Sensitivity$_{strict}$	F1 $_{strict}$ Score
DISCOVER	0.667	0.024	0.046	0.667	0.024	0.046
DISCOVER Strat	0.824	0.034	0.065	0.824	0.034	0.065
Fisher's Exact Test	1.000	0.005	0.010	1.000	0.005	0.010
WExT	0.750	0.066	0.121	0.743	0.063	0.116

(c) Metrics for COADREAD data. (498 samples | 784 CGC-CGC pairs)

Method	Precision	Sensitivity	F1 Score	Precision$_{strict}$	Sensitivity$_{strict}$	F1 $_{strict}$ Score
DISCOVER	0.687	0.059	0.109	0.702	0.052	0.097
DISCOVER Strat	0.706	0.015	0.030	0.706	0.015	0.029
Fisher's Exact Test	0.583	0.009	0.018	0.583	0.009	0.018
WExT	0.668	0.133	0.221	0.705	0.110	0.190

(d) Metrics for LUAD data. (568 samples | 636 CGC-CGC pairs)

Method	Precision	Sensitivity	F1 Score	Precision$_{strict}$	Sensitivity$_{strict}$	F1 $_{strict}$ Score
DISCOVER	0.800	0.013	0.025	0.80	0.013	0.026
Fisher's Exact Test	NaN	0.000	NaN	NaN	0.000	NaN
WExT	0.769	0.032	0.061	0.76	0.030	0.058

(e) Metrics for LUSC data. (485 samples | 478 CGC-CGC pairs)

Method	Precision	Sensitivity	F1 Score	Precision$_{strict}$	Sensitivity$_{strict}$	F1 $_{strict}$ Score
DISCOVER	1.000	0.004	0.008	1.000	0.004	0.008
Fisher's Exact Test	1.000	0.004	0.008	1.000	0.004	0.008
WExT	0.857	0.013	0.025	0.857	0.013	0.026

(f) Metrics for SKCM data. (468 samples | 1036 CGC-CGC pairs)

Method	Precision	Sensitivity	F1 Score	Precision$_{strict}$	Sensitivity$_{strict}$	F1 $_{strict}$ Score
DISCOVER	0.842	0.016	0.031	0.842	0.016	0.031
Fisher's Exact Test	1.000	0.002	0.004	1.000	0.002	0.004
WExT	0.807	0.048	0.090	0.832	0.047	0.089

(g) Metrics for STAD data. (438 samples | 714 CGC-CGC pairs)

Method	Precision	Sensitivity	F1 Score	Precision$_{strict}$	Sensitivity$_{strict}$	F1 $_{strict}$ Score
DISCOVER	0.684	0.037	0.069	0.706	0.034	0.065
Fisher's Exact Test	0.800	0.006	0.011	0.800	0.006	0.012
WExT	0.667	0.077	0.138	0.692	0.064	0.117

(h) Metrics for UCEC data. (531 samples | 1256 CGC-CGC pairs)

Method	Precision	Sensitivity	F1 Score	Precision$_{strict}$	Sensitivity$_{strict}$	F1 $_{strict}$ Score
DISCOVER	0.673	0.154	0.250	0.721	0.130	0.220
Fisher's Exact Test	0.800	0.006	0.013	0.800	0.006	0.012
WExT	0.637	0.231	0.339	0.683	0.190	0.297

Table 34: Results of network-centric ME evaluation framework with $\mathcal{G}$ = HINT, $\mathcal{S}$ = CGC, c= X_1, p_t = 0.05, t=5, robustness_iterations = 100

(a) Metrics for BLCA data. (411 samples | 800 CGC-CGC pairs)

Method	Precision	Sensitivity	F1 Score	Precision$_{strict}$	Sensitivity$_{strict}$	F1 $_{strict}$ Score
DISCOVER	0.903	0.018	0.035	0.903	0.018	0.035
Fisher's Exact Test	1.000	0.005	0.010	1.000	0.005	0.010
WExT	0.750	0.030	0.058	0.750	0.030	0.058

(b) Metrics for BRCA data. (1026 samples | 736 CGC-CGC pairs)

Method	Precision	Sensitivity	F1 Score	Precision$_{strict}$	Sensitivity$_{strict}$	F1 $_{strict}$ Score
DISCOVER	0.741	0.027	0.053	0.741	0.027	0.052
DISCOVER Strat	0.800	0.033	0.063	0.800	0.033	0.063
Fisher's Exact Test	1.000	0.003	0.005	1.000	0.003	0.006
WExT	0.770	0.075	0.136	0.785	0.071	0.130

(c) Metrics for COADREAD data. (498 samples | 1278 CGC-CGC pairs)

Method	Precision	Sensitivity	F1 Score	Precision$_{strict}$	Sensitivity$_{strict}$	F1 $_{strict}$ Score
DISCOVER	0.695	0.071	0.128	0.736	0.064	0.118
DISCOVER Strat	0.771	0.029	0.056	0.783	0.028	0.054
Fisher's Exact Test	0.738	0.018	0.034	0.754	0.017	0.033
WExT	0.657	0.137	0.226	0.707	0.118	0.202

(d) Metrics for LUAD data. (568 samples | 1018 CGC-CGC pairs)

Method	Precision	Sensitivity	F1 Score	Precision$_{strict}$	Sensitivity$_{strict}$	F1 $_{strict}$ Score
DISCOVER	0.769	0.010	0.019	0.769	0.010	0.020
Fisher's Exact Test	0.667	0.002	0.004	0.667	0.002	0.004
WExT	0.722	0.026	0.050	0.714	0.025	0.048

(e) Metrics for LUSC data. (485 samples | 794 CGC-CGC pairs)

Method	Precision	Sensitivity	F1 Score	Precision$_{strict}$	Sensitivity$_{strict}$	F1 $_{strict}$ Score
DISCOVER	1.000	0.003	0.005	1.000	0.003	0.006
Fisher's Exact Test	1.000	0.003	0.005	1.000	0.003	0.006
WExT	0.667	0.005	0.010	0.667	0.005	0.010

(f) Metrics for SKCM data. (468 samples | 1560 CGC-CGC pairs)

Method	Precision	Sensitivity	F1 Score	Precision$_{strict}$	Sensitivity$_{strict}$	F1 $_{strict}$ Score
DISCOVER	0.800	0.010	0.020	0.800	0.010	0.020
Fisher's Exact Test	NaN	0.000	NaN	NaN	0.000	NaN
WExT	0.721	0.039	0.074	0.718	0.038	0.072

(g) Metrics for STAD data. (438 samples | 1100 CGC-CGC pairs)

Method	Precision	Sensitivity	F1 Score	Precision$_{strict}$	Sensitivity$_{strict}$	F1 $_{strict}$ Score
DISCOVER	0.630	0.029	0.055	0.656	0.027	0.052
Fisher's Exact Test	0.500	0.002	0.004	0.500	0.002	0.004
WExT	0.713	0.081	0.145	0.750	0.070	0.128

(h) Metrics for UCEC data. (531 samples | 1884 CGC-CGC pairs)

Method	Precision	Sensitivity	F1 Score	Precision$_{strict}$	Sensitivity$_{strict}$	F1 $_{strict}$ Score
DISCOVER	0.681	0.153	0.249	0.741	0.132	0.224
Fisher's Exact Test	0.800	0.006	0.013	0.800	0.006	0.012
WExT	0.657	0.252	0.365	0.717	0.217	0.333

Table 35: Results of network-centric ME evaluation framework with $\mathcal{G}$ = STRING, $\mathcal{S}$ = CGC, c= X_1, p_t = 0.05, t=5, robustness_iterations = 100

(a) Metrics for BLCA data. (411 samples | 1368 CGC-CGC pairs)

Method	Precision	Sensitivity	F1 Score	$\text{Precision}_{\text{strict}}$	$\text{Sensitivity}_{\text{strict}}$	F1 $_{\text{strict}}$ Score
DISCOVER	0.933	0.010	0.020	0.933	0.010	0.020
Fisher's Exact Test	1.000	0.003	0.006	1.000	0.003	0.006
WExT	0.706	0.018	0.035	0.706	0.018	0.035

(b) Metrics for BRCA data. (1026 samples | 1110 CGC-CGC pairs)

Method	Precision	Sensitivity	F1 Score	$\text{Precision}_{\text{strict}}$	$\text{Sensitivity}_{\text{strict}}$	F1 $_{\text{strict}}$ Score
DISCOVER	0.824	0.038	0.073	0.824	0.038	0.073
DISCOVER Strat	0.900	0.041	0.078	0.900	0.041	0.078
Fisher's Exact Test	1.000	0.002	0.004	1.000	0.002	0.004
WExT	0.775	0.077	0.141	0.788	0.073	0.134

(c) Metrics for COADREAD data. (498 samples | 1994 CGC-CGC pairs)

Method	Precision	Sensitivity	F1 Score	$\text{Precision}_{\text{strict}}$	$\text{Sensitivity}_{\text{strict}}$	F1 $_{\text{strict}}$ Score
DISCOVER	0.701	0.048	0.090	0.746	0.045	0.085
DISCOVER Strat	0.862	0.017	0.034	0.873	0.017	0.033
Fisher's Exact Test	0.850	0.009	0.017	0.850	0.009	0.018
WExT	0.664	0.096	0.168	0.711	0.085	0.152

(d) Metrics for LUAD data. (568 samples | 1464 CGC-CGC pairs)

Method	Precision	Sensitivity	F1 Score	$\text{Precision}_{\text{strict}}$	$\text{Sensitivity}_{\text{strict}}$	F1 $_{\text{strict}}$ Score
DISCOVER	0.800	0.014	0.027	0.833	0.014	0.028
Fisher's Exact Test	0.909	0.007	0.014	0.909	0.007	0.014
WExT	0.745	0.024	0.047	0.786	0.023	0.045

(e) Metrics for LUSC data. (485 samples | 1188 CGC-CGC pairs)

Method	Precision	Sensitivity	F1 Score	$\text{Precision}_{\text{strict}}$	$\text{Sensitivity}_{\text{strict}}$	F1 $_{\text{strict}}$ Score
DISCOVER	0.800	0.003	0.007	0.800	0.003	0.006
Fisher's Exact Test	1.000	0.002	0.003	1.000	0.002	0.004
WExT	0.857	0.005	0.010	0.857	0.005	0.010

(f) Metrics for SKCM data. (468 samples | 2400 CGC-CGC pairs)

Method	Precision	Sensitivity	F1 Score	$\text{Precision}_{\text{strict}}$	$\text{Sensitivity}_{\text{strict}}$	F1 $_{\text{strict}}$ Score
DISCOVER	0.830	0.016	0.032	0.844	0.016	0.031
Fisher's Exact Test	1.000	0.002	0.003	1.000	0.002	0.004
WExT	0.714	0.037	0.070	0.740	0.036	0.069

(g) Metrics for STAD data. (438 samples | 1628 CGC-CGC pairs)

Method	Precision	Sensitivity	F1 Score	$\text{Precision}_{\text{strict}}$	$\text{Sensitivity}_{\text{strict}}$	F1 $_{\text{strict}}$ Score
DISCOVER	0.718	0.017	0.034	0.750	0.017	0.033
Fisher's Exact Test	0.667	0.001	0.002	0.667	0.001	0.002
WExT	0.714	0.039	0.074	0.765	0.036	0.069

(h) Metrics for UCEC data. (531 samples | 3044 CGC-CGC pairs)

Method	Precision	Sensitivity	F1 Score	$\text{Precision}_{\text{strict}}$	$\text{Sensitivity}_{\text{strict}}$	F1 $_{\text{strict}}$ Score
DISCOVER	0.679	0.110	0.190	0.734	0.096	0.170
Fisher's Exact Test	0.923	0.004	0.008	0.923	0.004	0.008
WExT	0.620	0.194	0.295	0.666	0.164	0.263

Table 36: Degree-normalized network-centric evaluations X_1 and $t = 5$

(a) Metrics for BLCA data.

Method	Precision	Sensitivity	F1 Score	$\text{Precision}_{\text{strict}}$	$\text{Sensitivity}_{\text{strict}}$	F1 $_{\text{strict}}$ Score
DISCOVER	0.958	0.268	0.367	0.958	0.268	0.367
Fisher's Exact Test	1.0	0.072	0.133	1.0	0.072	0.133
WExT	0.798	0.279	0.369	0.798	0.279	0.369

(b) Metrics for BRCA data.

Method	Precision	Sensitivity	F1 Score	$\text{Precision}_{\text{strict}}$	$\text{Sensitivity}_{\text{strict}}$	F1 $_{\text{strict}}$ Score
DISCOVER	0.873	0.408	0.496	0.873	0.408	0.496
DISCOVER Strat	0.927	0.393	0.481	0.927	0.393	0.481
Fisher's Exact Test	0.667	0.125	0.193	0.667	0.125	0.193
WExT	0.874	0.352	0.448	0.876	0.348	0.446

(c) Metrics for COADREAD data.

Method	Precision	Sensitivity	F1 Score	$\text{Precision}_{\text{strict}}$	$\text{Sensitivity}_{\text{strict}}$	F1 $_{\text{strict}}$ Score
DISCOVER	0.844	0.306	0.389	0.843	0.293	0.381
DISCOVER Strat	0.812	0.117	0.195	0.812	0.117	0.195
Fisher's Exact Test	0.881	0.198	0.293	0.875	0.194	0.288
WExT	0.831	0.35	0.429	0.827	0.323	0.417

(d) Metrics for LUAD data.

Method	Precision	Sensitivity	F1 Score	$\text{Precision}_{\text{strict}}$	$\text{Sensitivity}_{\text{strict}}$	F1 $_{\text{strict}}$ Score
DISCOVER	0.935	0.346	0.449	0.932	0.343	0.446
Fisher's Exact Test	0.0	0.0	0.0	0.0	0.0	0.0
WExT	0.935	0.299	0.394	0.936	0.296	0.393

(e) Metrics for LUSC data.

Method	Precision	Sensitivity	F1 Score	$\text{Precision}_{\text{strict}}$	$\text{Sensitivity}_{\text{strict}}$	F1 $_{\text{strict}}$ Score
DISCOVER	1.0	0.625	0.7	1.0	0.625	0.7
Fisher's Exact Test	1.0	0.6	0.667	1.0	0.6	0.667
WExT	0.917	0.334	0.436	0.917	0.334	0.436

(f) Metrics for SKCM data.

Method	Precision	Sensitivity	F1 Score	$\text{Precision}_{\text{strict}}$	$\text{Sensitivity}_{\text{strict}}$	F1 $_{\text{strict}}$ Score
DISCOVER	0.849	0.18	0.266	0.849	0.18	0.266
Fisher's Exact Test	1.0	0.156	0.267	1.0	0.156	0.267
WExT	0.82	0.205	0.293	0.819	0.202	0.291

(g) Metrics for STAD data.

Method	Precision	Sensitivity	F1 Score	$\text{Precision}_{\text{strict}}$	$\text{Sensitivity}_{\text{strict}}$	F1 $_{\text{strict}}$ Score
DISCOVER	0.897	0.214	0.317	0.896	0.21	0.314
Fisher's Exact Test	0.833	0.227	0.33	0.833	0.227	0.33
WExT	0.892	0.293	0.389	0.887	0.282	0.382

(h) Metrics for UCEC data.

Method	Precision	Sensitivity	F1 Score	$\text{Precision}_{\text{strict}}$	$\text{Sensitivity}_{\text{strict}}$	F1 $_{\text{strict}}$ Score
DISCOVER	0.874	0.297	0.381	0.876	0.278	0.374
Fisher's Exact Test	0.939	0.086	0.151	0.939	0.086	0.151
WExT	0.804	0.378	0.451	0.805	0.348	0.439

Table 37: Results of network-centric ME evaluation framework with $\mathcal{G}$ = Intact (w conf. threshold 0.35), $\mathcal{S}$ = CGC, c= X_2, p_t = 0.05, t=5, robustness_iterations = 100

(a) Metrics for BLCA data. (411 samples | 895 CGC-CGC pairs)

Method	Precision	Sensitivity	F1 Score	$\text{Precision}_{\text{strict}}$	$\text{Sensitivity}_{\text{strict}}$	F1 $_{\text{strict}}$ Score
DISCOVER	1.0	0.013	0.026	1.0	0.013	0.026
Fisher's Exact Test	1.0	0.002	0.004	1.0	0.002	0.004
WExT	0.8	0.022	0.043	0.8	0.022	0.043

(b) Metrics for BRCA data. (1026 samples | 737 CGC-CGC pairs)

Method	Precision	Sensitivity	F1 Score	$\text{Precision}_{\text{strict}}$	$\text{Sensitivity}_{\text{strict}}$	F1 $_{\text{strict}}$ Score
DISCOVER	0.773	0.023	0.045	0.773	0.023	0.045
DISCOVER Strat	0.852	0.031	0.060	0.852	0.031	0.060
Fisher's Exact Test	1.000	0.003	0.005	1.000	0.003	0.006
WExT	0.698	0.060	0.110	0.724	0.057	0.106

(c) Metrics for COADREAD data. (498 samples | 1625 CGC-CGC pairs)

Method	Precision	Sensitivity	F1 Score	$\text{Precision}_{\text{strict}}$	$\text{Sensitivity}_{\text{strict}}$	F1 $_{\text{strict}}$ Score
DISCOVER	0.721	0.052	0.097	0.746	0.048	0.090
DISCOVER Strat	0.641	0.013	0.025	0.641	0.013	0.025
Fisher's Exact Test	0.619	0.008	0.016	0.619	0.008	0.016
WExT	0.670	0.118	0.200	0.712	0.103	0.180

(d) Metrics for LUAD data. (568 samples | 1197 CGC-CGC pairs)

Method	Precision	Sensitivity	F1 Score	$\text{Precision}_{\text{strict}}$	$\text{Sensitivity}_{\text{strict}}$	F1 $_{\text{strict}}$ Score
DISCOVER	0.889	0.013	0.026	0.889	0.013	0.026
Fisher's Exact Test	NaN	0.000	NaN	NaN	0.000	NaN
WExT	0.804	0.031	0.060	0.818	0.030	0.058

(e) Metrics for LUSC data. (485 samples | 906 CGC-CGC pairs)

Method	Precision	Sensitivity	F1 Score	$\text{Precision}_{\text{strict}}$	$\text{Sensitivity}_{\text{strict}}$	F1 $_{\text{strict}}$ Score
DISCOVER	1.000	0.002	0.004	1.000	0.002	0.004
Fisher's Exact Test	0.667	0.002	0.004	0.667	0.002	0.004
WExT	0.600	0.007	0.013	0.600	0.007	0.014

(f) Metrics for SKCM data. (468 samples | 2142 CGC-CGC pairs)

Method	Precision	Sensitivity	F1 Score	$\text{Precision}_{\text{strict}}$	$\text{Sensitivity}_{\text{strict}}$	F1 $_{\text{strict}}$ Score
DISCOVER	0.784	0.014	0.027	0.806	0.014	0.028
Fisher's Exact Test	1.000	0.001	0.002	1.000	0.001	0.002
WExT	0.743	0.042	0.080	0.753	0.042	0.080

(g) Metrics for STAD data. (438 samples | 1308 CGC-CGC pairs)

Method	Precision	Sensitivity	F1 Score	$\text{Precision}_{\text{strict}}$	$\text{Sensitivity}_{\text{strict}}$	F1 $_{\text{strict}}$ Score
DISCOVER	0.743	0.029	0.055	0.763	0.027	0.052
Fisher's Exact Test	0.800	0.003	0.006	0.800	0.003	0.006
WExT	0.718	0.072	0.132	0.757	0.065	0.120

(h) Metrics for UCEC data. (531 samples | 2668 CGC-CGC pairs)

Method	Precision	Sensitivity	F1 Score	$\text{Precision}_{\text{strict}}$	$\text{Sensitivity}_{\text{strict}}$	F1 $_{\text{strict}}$ Score
DISCOVER	0.703	0.130	0.219	0.777	0.117	0.203
Fisher's Exact Test	0.833	0.004	0.007	0.833	0.004	0.008
WExT	0.651	0.213	0.321	0.728	0.189	0.300

Table 38: Results of network-centric ME evaluation framework with $\mathcal{G}$ = Intact (w conf. threshold 0.35), $\mathcal{S}$ = CGC, c= X_2, p_t = 0.05, t=5, robustness_iterations = 300

(a) Metrics for BLCA data. (411 samples | 895 CGC-CGC pairs)

Method	Precision	Sensitivity	F1 Score	$\text{Precision}_{\text{strict}}$	$\text{Sensitivity}_{\text{strict}}$	F1 $_{\text{strict}}$ Score
DISCOVER	1.0	0.013	0.026	1.0	0.013	0.026
Fisher's Exact Test	1.0	0.002	0.004	1.0	0.002	0.004
WExT	0.8	0.022	0.043	0.8	0.022	0.043

(b) Metrics for BRCA data. (1026 samples | 737 CGC-CGC pairs)

Method	Precision	Sensitivity	F1 Score	$\text{Precision}_{\text{strict}}$	$\text{Sensitivity}_{\text{strict}}$	F1 $_{\text{strict}}$ Score
DISCOVER	0.773	0.023	0.045	0.773	0.023	0.045
DISCOVER Strat	0.852	0.031	0.060	0.852	0.031	0.060
Fisher's Exact Test	1.000	0.003	0.005	1.000	0.003	0.006
WExT	0.698	0.060	0.110	0.724	0.057	0.106

(c) Metrics for COADREAD data. (498 samples | 1625 CGC-CGC pairs)

Method	Precision	Sensitivity	F1 Score	$\text{Precision}_{\text{strict}}$	$\text{Sensitivity}_{\text{strict}}$	F1 $_{\text{strict}}$ Score
DISCOVER	0.714	0.053	0.098	0.736	0.048	0.090
DISCOVER Strat	0.645	0.012	0.024	0.645	0.012	0.024
Fisher's Exact Test	0.619	0.008	0.016	0.619	0.008	0.016
WExT	0.676	0.116	0.198	0.720	0.101	0.177

(d) Metrics for LUAD data. (568 samples | 1197 CGC-CGC pairs)

Method	Precision	Sensitivity	F1 Score	$\text{Precision}_{\text{strict}}$	$\text{Sensitivity}_{\text{strict}}$	F1 $_{\text{strict}}$ Score
DISCOVER	0.842	0.013	0.026	0.842	0.013	0.026
Fisher's Exact Test	NaN	0.000	NaN	NaN	0.000	NaN
WExT	0.800	0.030	0.058	0.814	0.029	0.056

(e) Metrics for LUSC data. (485 samples | 906 CGC-CGC pairs)

Method	Precision	Sensitivity	F1 Score	$\text{Precision}_{\text{strict}}$	$\text{Sensitivity}_{\text{strict}}$	F1 $_{\text{strict}}$ Score
DISCOVER	1.0	0.002	0.004	1.0	0.002	0.004
Fisher's Exact Test	1.0	0.002	0.004	1.0	0.002	0.004
WExT	0.6	0.007	0.013	0.6	0.007	0.014

(f) Metrics for SKCM data. (468 samples | 2142 CGC-CGC pairs)

Method	Precision	Sensitivity	F1 Score	$\text{Precision}_{\text{strict}}$	$\text{Sensitivity}_{\text{strict}}$	F1 $_{\text{strict}}$ Score
DISCOVER	0.800	0.013	0.026	0.800	0.013	0.026
Fisher's Exact Test	1.000	0.001	0.002	1.000	0.001	0.002
WExT	0.754	0.042	0.079	0.765	0.041	0.078

(g) Metrics for STAD data. (438 samples | 1308 CGC-CGC pairs)

Method	Precision	Sensitivity	F1 Score	$\text{Precision}_{\text{strict}}$	$\text{Sensitivity}_{\text{strict}}$	F1 $_{\text{strict}}$ Score
DISCOVER	0.738	0.029	0.056	0.758	0.028	0.054
Fisher's Exact Test	0.800	0.003	0.006	0.800	0.003	0.006
WExT	0.725	0.074	0.134	0.771	0.066	0.122

(h) Metrics for UCEC data. (531 samples | 2668 CGC-CGC pairs)

Method	Precision	Sensitivity	F1 Score	$\text{Precision}_{\text{strict}}$	$\text{Sensitivity}_{\text{strict}}$	F1 $_{\text{strict}}$ Score
DISCOVER	0.700	0.131	0.220	0.772	0.117	0.203
Fisher's Exact Test	0.833	0.004	0.007	0.833	0.004	0.008
WExT	0.649	0.214	0.322	0.725	0.188	0.299

Table 39: Results of network-centric ME evaluation framework with $\mathcal{G}$ = Intact (w conf. threshold 0.35), $\mathcal{S}$ = CGC, c= X_2, p_t = 0.05, t=5, robustness_iterations = 500

(a) Metrics for BLCA data. (411 samples | 895 CGC-CGC pairs)

Method	Precision	Sensitivity	F1 Score	$\text{Precision}_{\text{strict}}$	$\text{Sensitivity}_{\text{strict}}$	F1 $_{\text{strict}}$ Score
DISCOVER	0.923	0.012	0.023	0.923	0.012	0.024
Fisher's Exact Test	1.000	0.002	0.004	1.000	0.002	0.004
WExT	0.690	0.019	0.037	0.690	0.019	0.037

(b) Metrics for BRCA data. (1026 samples | 737 CGC-CGC pairs)

Method	Precision	Sensitivity	F1 Score	$\text{Precision}_{\text{strict}}$	$\text{Sensitivity}_{\text{strict}}$	F1 $_{\text{strict}}$ Score
DISCOVER	0.719	0.024	0.047	0.719	0.024	0.046
DISCOVER Strat	0.789	0.031	0.060	0.789	0.031	0.060
Fisher's Exact Test	0.667	0.002	0.004	0.667	0.002	0.004
WExT	0.705	0.058	0.107	0.726	0.056	0.104

(c) Metrics for COADREAD data. (498 samples | 1625 CGC-CGC pairs)

Method	Precision	Sensitivity	F1 Score	$\text{Precision}_{\text{strict}}$	$\text{Sensitivity}_{\text{strict}}$	F1 $_{\text{strict}}$ Score
DISCOVER	0.643	0.052	0.097	0.653	0.046	0.086
DISCOVER Strat	0.600	0.012	0.024	0.600	0.012	0.024
Fisher's Exact Test	0.591	0.007	0.015	0.591	0.007	0.014
WExT	0.652	0.120	0.202	0.677	0.102	0.177

(d) Metrics for LUAD data. (568 samples | 1197 CGC-CGC pairs)

Method	Precision	Sensitivity	F1 Score	$\text{Precision}_{\text{strict}}$	$\text{Sensitivity}_{\text{strict}}$	F1 $_{\text{strict}}$ Score
DISCOVER	0.800	0.015	0.029	0.792	0.014	0.028
Fisher's Exact Test	0.000	0.000	NaN	0.000	0.000	NaN
WExT	0.784	0.030	0.058	0.792	0.029	0.056

(e) Metrics for LUSC data. (485 samples | 906 CGC-CGC pairs)

Method	Precision	Sensitivity	F1 Score	$\text{Precision}_{\text{strict}}$	$\text{Sensitivity}_{\text{strict}}$	F1 $_{\text{strict}}$ Score
DISCOVER	1.000	0.002	0.004	1.000	0.002	0.004
Fisher's Exact Test	1.000	0.002	0.004	1.000	0.002	0.004
WExT	0.857	0.006	0.011	0.857	0.006	0.012

(f) Metrics for SKCM data. (468 samples | 2142 CGC-CGC pairs)

Method	Precision	Sensitivity	F1 Score	$\text{Precision}_{\text{strict}}$	$\text{Sensitivity}_{\text{strict}}$	F1 $_{\text{strict}}$ Score
DISCOVER	0.821	0.014	0.028	0.821	0.014	0.028
Fisher's Exact Test	1.000	0.001	0.002	1.000	0.001	0.002
WExT	0.723	0.043	0.080	0.730	0.042	0.079

(g) Metrics for STAD data. (438 samples | 1308 CGC-CGC pairs)

Method	Precision	Sensitivity	F1 Score	$\text{Precision}_{\text{strict}}$	$\text{Sensitivity}_{\text{strict}}$	F1 $_{\text{strict}}$ Score
DISCOVER	0.667	0.028	0.053	0.673	0.026	0.050
Fisher's Exact Test	0.667	0.003	0.005	0.667	0.003	0.006
WExT	0.692	0.069	0.125	0.711	0.060	0.111

(h) Metrics for UCEC data. (531 samples | 2668 CGC-CGC pairs)

Method	Precision	Sensitivity	F1 Score	$\text{Precision}_{\text{strict}}$	$\text{Sensitivity}_{\text{strict}}$	F1 $_{\text{strict}}$ Score
DISCOVER	0.661	0.128	0.215	0.704	0.110	0.190
Fisher's Exact Test	0.769	0.004	0.007	0.769	0.004	0.008
WExT	0.628	0.213	0.318	0.675	0.180	0.284

Table 40: Results of network-centric ME evaluation framework with $\mathcal{G}$ = Intact (w conf. threshold 0.35), $\mathcal{S}$ = CGC, c= X_2, p_t = 0.01, t=5, robustness_iterations = 100

(a) Metrics for BLCA data. (411 samples | 895 CGC-CGC pairs)

Method	Precision	Sensitivity	F1 Score	$\text{Precision}_{\text{strict}}$	$\text{Sensitivity}_{\text{strict}}$	F1 $_{\text{strict}}$ Score
DISCOVER	1.0	0.002	0.004	1.0	0.002	0.004
Fisher's Exact Test	NaN	0.000	NaN	NaN	0.000	NaN
WExT	0.8	0.004	0.009	0.8	0.004	0.008

(b) Metrics for BRCA data. (1026 samples | 737 CGC-CGC pairs)

Method	Precision	Sensitivity	F1 Score	$\text{Precision}_{\text{strict}}$	$\text{Sensitivity}_{\text{strict}}$	F1 $_{\text{strict}}$ Score
DISCOVER	1.000	0.008	0.016	1.000	0.008	0.016
DISCOVER Strat	0.857	0.008	0.016	0.857	0.008	0.016
Fisher's Exact Test	NaN	0.000	NaN	NaN	0.000	NaN
WExT	0.692	0.012	0.024	0.692	0.012	0.024

(c) Metrics for COADREAD data. (498 samples | 1625 CGC-CGC pairs)

Method	Precision	Sensitivity	F1 Score	$\text{Precision}_{\text{strict}}$	$\text{Sensitivity}_{\text{strict}}$	F1 $_{\text{strict}}$ Score
DISCOVER	0.756	0.021	0.041	0.767	0.020	0.039
DISCOVER Strat	0.000	0.000	NaN	0.000	0.000	NaN
Fisher's Exact Test	0.286	0.001	0.002	0.286	0.001	0.002
WExT	0.716	0.068	0.125	0.775	0.057	0.106

(d) Metrics for LUAD data. (568 samples | 1197 CGC-CGC pairs)

Method	Precision	Sensitivity	F1 Score	$\text{Precision}_{\text{strict}}$	$\text{Sensitivity}_{\text{strict}}$	F1 $_{\text{strict}}$ Score
DISCOVER	0.800	0.003	0.007	0.800	0.003	0.006
Fisher's Exact Test	NaN	0.000	NaN	NaN	0.000	NaN
WExT	0.889	0.013	0.027	0.889	0.013	0.026

(e) Metrics for LUSC data. (485 samples | 906 CGC-CGC pairs)

Method	Precision	Sensitivity	F1 Score	$\text{Precision}_{\text{strict}}$	$\text{Sensitivity}_{\text{strict}}$	F1 $_{\text{strict}}$ Score
DISCOVER	1.000	0.002	0.004	1.000	0.002	0.004
Fisher's Exact Test	NaN	0.000	NaN	NaN	0.000	NaN
WExT	0.667	0.002	0.004	0.667	0.002	0.004

(f) Metrics for SKCM data. (468 samples | 2142 CGC-CGC pairs)

Method	Precision	Sensitivity	F1 Score	$\text{Precision}_{\text{strict}}$	$\text{Sensitivity}_{\text{strict}}$	F1 $_{\text{strict}}$ Score
DISCOVER	1.00	0.003	0.006	1.00	0.003	0.006
Fisher's Exact Test	1.00	0.001	0.002	1.00	0.001	0.002
WExT	0.87	0.009	0.019	0.87	0.009	0.018

(g) Metrics for STAD data. (438 samples | 1308 CGC-CGC pairs)

Method	Precision	Sensitivity	F1 Score	$\text{Precision}_{\text{strict}}$	$\text{Sensitivity}_{\text{strict}}$	F1 $_{\text{strict}}$ Score
DISCOVER	0.667	0.008	0.015	0.667	0.008	0.016
Fisher's Exact Test	1.000	0.002	0.003	1.000	0.002	0.004
WExT	0.679	0.028	0.053	0.744	0.025	0.048

(h) Metrics for UCEC data. (531 samples | 2668 CGC-CGC pairs)

Method	Precision	Sensitivity	F1 Score	$\text{Precision}_{\text{strict}}$	$\text{Sensitivity}_{\text{strict}}$	F1 $_{\text{strict}}$ Score
DISCOVER	0.740	0.074	0.134	0.813	0.069	0.127
Fisher's Exact Test	1.000	0.002	0.004	1.000	0.002	0.004
WExT	0.686	0.137	0.229	0.776	0.122	0.211

Table 41: Results of network-centric ME evaluation framework with $\mathcal{G}$ = Intact (w conf. threshold 0.35), $\mathcal{S}$ = CGC, c= X_2, p_t = 0.1, t=5, robustness_iterations = 100

(a) Metrics for BLCA data. (411 samples | 895 CGC-CGC pairs)

Method	Precision	Sensitivity	F1 Score	$\text{Precision}_{\text{strict}}$	$\text{Sensitivity}_{\text{strict}}$	F1 $_{\text{strict}}$ Score
DISCOVER	0.909	0.022	0.044	0.909	0.022	0.043
Fisher's Exact Test	1.000	0.002	0.004	1.000	0.002	0.004
WExT	0.746	0.053	0.098	0.738	0.050	0.094

(b) Metrics for BRCA data. (1026 samples | 737 CGC-CGC pairs)

Method	Precision	Sensitivity	F1 Score	$\text{Precision}_{\text{strict}}$	$\text{Sensitivity}_{\text{strict}}$	F1 $_{\text{strict}}$ Score
DISCOVER	0.740	0.050	0.094	0.755	0.050	0.094
DISCOVER Strat	0.741	0.058	0.108	0.750	0.057	0.106
Fisher's Exact Test	1.000	0.003	0.005	1.000	0.003	0.006
WExT	0.700	0.104	0.181	0.720	0.093	0.165

(c) Metrics for COADREAD data. (498 samples | 1625 CGC-CGC pairs)

Method	Precision	Sensitivity	F1 Score	$\text{Precision}_{\text{strict}}$	$\text{Sensitivity}_{\text{strict}}$	F1 $_{\text{strict}}$ Score
DISCOVER	0.689	0.089	0.158	0.718	0.081	0.146
DISCOVER Strat	0.663	0.041	0.077	0.660	0.039	0.074
Fisher's Exact Test	0.698	0.018	0.036	0.692	0.017	0.033
WExT	0.659	0.183	0.286	0.703	0.163	0.265

(d) Metrics for LUAD data. (568 samples | 1197 CGC-CGC pairs)

Method	Precision	Sensitivity	F1 Score	$\text{Precision}_{\text{strict}}$	$\text{Sensitivity}_{\text{strict}}$	F1 $_{\text{strict}}$ Score
DISCOVER	0.813	0.031	0.060	0.847	0.030	0.058
Fisher's Exact Test	1.000	0.004	0.008	1.000	0.004	0.008
WExT	0.795	0.056	0.105	0.838	0.053	0.100

(e) Metrics for LUSC data. (485 samples | 906 CGC-CGC pairs)

Method	Precision	Sensitivity	F1 Score	$\text{Precision}_{\text{strict}}$	$\text{Sensitivity}_{\text{strict}}$	F1 $_{\text{strict}}$ Score
DISCOVER	0.800	0.004	0.009	0.800	0.004	0.008
Fisher's Exact Test	0.500	0.002	0.004	0.500	0.002	0.004
WExT	0.619	0.015	0.028	0.619	0.015	0.029

(f) Metrics for SKCM data. (468 samples | 2142 CGC-CGC pairs)

Method	Precision	Sensitivity	F1 Score	$\text{Precision}_{\text{strict}}$	$\text{Sensitivity}_{\text{strict}}$	F1 $_{\text{strict}}$ Score
DISCOVER	0.777	0.039	0.075	0.784	0.039	0.074
Fisher's Exact Test	1.000	0.002	0.004	1.000	0.002	0.004
WExT	0.716	0.080	0.144	0.726	0.079	0.142

(g) Metrics for STAD data. (438 samples | 1308 CGC-CGC pairs)

Method	Precision	Sensitivity	F1 Score	$\text{Precision}_{\text{strict}}$	$\text{Sensitivity}_{\text{strict}}$	F1 $_{\text{strict}}$ Score
DISCOVER	0.741	0.056	0.104	0.771	0.053	0.099
Fisher's Exact Test	0.727	0.006	0.012	0.727	0.006	0.012
WExT	0.658	0.098	0.171	0.706	0.089	0.158

(h) Metrics for UCEC data. (531 samples | 2668 CGC-CGC pairs)

Method	Precision	Sensitivity	F1 Score	$\text{Precision}_{\text{strict}}$	$\text{Sensitivity}_{\text{strict}}$	F1 $_{\text{strict}}$ Score
DISCOVER	0.658	0.173	0.274	0.725	0.154	0.254
Fisher's Exact Test	0.810	0.006	0.013	0.810	0.006	0.012
WExT	0.620	0.271	0.377	0.681	0.237	0.352

Table 42: Results of network-centric ME evaluation framework with $\mathcal{G}$ = Intact (w conf. threshold 0.35), $\mathcal{S}$ = CGC_{SNV} , c= X_2, p_t = 0.05, t=5, robustness_iterations = 100

(a) Metrics for BLCA data. (411 samples | 584 CGC_{SNV}-CGC_{SNV} pairs)

Method	Precision	Sensitivity	F1 Score	$\text{Precision}_{\text{strict}}$	$\text{Sensitivity}_{\text{strict}}$	F1 $_{\text{strict}}$ Score
DISCOVER	1.000	0.014	0.027	1.000	0.014	0.028
Fisher's Exact Test	1.000	0.003	0.007	1.000	0.003	0.006
WExT	0.824	0.024	0.047	0.824	0.024	0.047

(b) Metrics for BRCA data. (1026 samples | 505 CGC_{SNV}-CGC_{SNV} pairs)

Method	Precision	Sensitivity	F1 Score	$\text{Precision}_{\text{strict}}$	$\text{Sensitivity}_{\text{strict}}$	F1 $_{\text{strict}}$ Score
DISCOVER	0.800	0.032	0.061	0.800	0.032	0.062
DISCOVER Strat	0.846	0.044	0.083	0.846	0.044	0.084
Fisher's Exact Test	1.000	0.004	0.008	1.000	0.004	0.008
WExT	0.757	0.084	0.151	0.781	0.082	0.148

(c) Metrics for COADREAD data. (498 samples | 919 CGC_{SNV}-CGC_{SNV} pairs)

Method	Precision	Sensitivity	F1 Score	$\text{Precision}_{\text{strict}}$	$\text{Sensitivity}_{\text{strict}}$	F1 $_{\text{strict}}$ Score
DISCOVER	0.725	0.068	0.124	0.771	0.064	0.118
DISCOVER Strat	0.731	0.021	0.040	0.731	0.021	0.041
Fisher's Exact Test	0.688	0.012	0.024	0.688	0.012	0.024
WExT	0.697	0.144	0.239	0.752	0.128	0.219

(d) Metrics for LUAD data. (568 samples | 645 CGC_{SNV}-CGC_{SNV} pairs)

Method	Precision	Sensitivity	F1 Score	$\text{Precision}_{\text{strict}}$	$\text{Sensitivity}_{\text{strict}}$	F1 $_{\text{strict}}$ Score
DISCOVER	0.867	0.020	0.039	0.867	0.020	0.039
Fisher's Exact Test	NaN	0.000	NaN	NaN	0.000	NaN
WExT	0.816	0.049	0.092	0.833	0.047	0.089

(e) Metrics for LUSC data. (485 samples | 586 CGC_{SNV}-CGC_{SNV} pairs)

Method	Precision	Sensitivity	F1 Score	$\text{Precision}_{\text{strict}}$	$\text{Sensitivity}_{\text{strict}}$	F1 $_{\text{strict}}$ Score
DISCOVER	1.0	0.003	0.007	1.0	0.003	0.006
Fisher's Exact Test	1.0	0.003	0.007	1.0	0.003	0.006
WExT	0.6	0.010	0.020	0.6	0.010	0.020

(f) Metrics for SKCM data. (468 samples | 1104 CGC_{SNV}-CGC_{SNV} pairs)

Method	Precision	Sensitivity	F1 Score	$\text{Precision}_{\text{strict}}$	$\text{Sensitivity}_{\text{strict}}$	F1 $_{\text{strict}}$ Score
DISCOVER	0.789	0.014	0.027	0.833	0.014	0.028
Fisher's Exact Test	1.000	0.002	0.004	1.000	0.002	0.004
WExT	0.849	0.057	0.107	0.871	0.056	0.105

(g) Metrics for STAD data. (438 samples | 722 CGC_{SNV}-CGC_{SNV} pairs)

Method	Precision	Sensitivity	F1 Score	$\text{Precision}_{\text{strict}}$	$\text{Sensitivity}_{\text{strict}}$	F1 $_{\text{strict}}$ Score
DISCOVER	0.734	0.033	0.062	0.750	0.031	0.060
Fisher's Exact Test	0.667	0.003	0.006	0.667	0.003	0.006
WExT	0.733	0.089	0.158	0.781	0.080	0.145

(h) Metrics for UCEC data. (531 samples | 1255 CGC_{SNV}-CGC_{SNV} pairs)

Method	Precision	Sensitivity	F1 Score	$\text{Precision}_{\text{strict}}$	$\text{Sensitivity}_{\text{strict}}$	F1 $_{\text{strict}}$ Score
DISCOVER	0.721	0.179	0.287	0.812	0.163	0.271
Fisher's Exact Test	0.909	0.008	0.016	0.909	0.008	0.016
WExT	0.680	0.291	0.408	0.773	0.259	0.388

Table 43: Results of network-centric ME evaluation framework with $\mathcal{G}$ = Intact (w conf. threshold 0.35), $\mathcal{S}$ = Intogen , c= X_2, p_t = 0.05, t=5, robustness_iterations = 100

(a) Metrics for BLCA data. (411 samples | 844 Intogen-Intogen pairs)

Method	Precision	Sensitivity	F1 Score	$\text{Precision}_{\text{strict}}$	$\text{Sensitivity}_{\text{strict}}$	F1 $_{\text{strict}}$ Score
DISCOVER	1.00	0.019	0.037	1.00	0.019	0.037
Fisher's Exact Test	1.00	0.002	0.005	1.00	0.002	0.004
WExT	0.83	0.026	0.051	0.83	0.026	0.050

(b) Metrics for BRCA data. (1026 samples | 754 Intogen-Intogen pairs)

Method	Precision	Sensitivity	F1 Score	$\text{Precision}_{\text{strict}}$	$\text{Sensitivity}_{\text{strict}}$	F1 $_{\text{strict}}$ Score
DISCOVER	0.800	0.027	0.051	0.800	0.027	0.052
DISCOVER Strat	0.862	0.033	0.064	0.862	0.033	0.064
Fisher's Exact Test	1.000	0.003	0.005	1.000	0.003	0.006
WExT	0.708	0.068	0.124	0.727	0.064	0.118

(c) Metrics for COADREAD data. (498 samples | 1495 Intogen-Intogen pairs)

Method	Precision	Sensitivity	F1 Score	$\text{Precision}_{\text{strict}}$	$\text{Sensitivity}_{\text{strict}}$	F1 $_{\text{strict}}$ Score
DISCOVER	0.754	0.068	0.124	0.790	0.063	0.117
DISCOVER Strat	0.775	0.021	0.041	0.775	0.021	0.041
Fisher's Exact Test	0.591	0.009	0.017	0.591	0.009	0.018
WExT	0.704	0.141	0.236	0.765	0.126	0.216

(d) Metrics for LUAD data. (568 samples | 1056 Intogen-Intogen pairs)

Method	Precision	Sensitivity	F1 Score	$\text{Precision}_{\text{strict}}$	$\text{Sensitivity}_{\text{strict}}$	F1 $_{\text{strict}}$ Score
DISCOVER	0.842	0.015	0.030	0.842	0.015	0.029
Fisher's Exact Test	NaN	0.000	NaN	NaN	0.000	NaN
WExT	0.812	0.037	0.071	0.826	0.036	0.069

(e) Metrics for LUSC data. (485 samples | 867 Intogen-Intogen pairs)

Method	Precision	Sensitivity	F1 Score	$\text{Precision}_{\text{strict}}$	$\text{Sensitivity}_{\text{strict}}$	F1 $_{\text{strict}}$ Score
DISCOVER	1.000	0.003	0.007	1.000	0.003	0.006
Fisher's Exact Test	0.667	0.002	0.005	0.667	0.002	0.004
WExT	0.692	0.010	0.021	0.692	0.010	0.020

(f) Metrics for SKCM data. (468 samples | 1774 Intogen-Intogen pairs)

Method	Precision	Sensitivity	F1 Score	$\text{Precision}_{\text{strict}}$	$\text{Sensitivity}_{\text{strict}}$	F1 $_{\text{strict}}$ Score
DISCOVER	0.833	0.014	0.028	0.833	0.014	0.028
Fisher's Exact Test	1.000	0.001	0.002	1.000	0.001	0.002
WExT	0.774	0.048	0.090	0.787	0.047	0.089

(g) Metrics for STAD data. (438 samples | 1258 Intogen-Intogen pairs)

Method	Precision	Sensitivity	F1 Score	$\text{Precision}_{\text{strict}}$	$\text{Sensitivity}_{\text{strict}}$	F1 $_{\text{strict}}$ Score
DISCOVER	0.778	0.033	0.064	0.800	0.032	0.062
Fisher's Exact Test	0.833	0.004	0.008	0.833	0.004	0.008
WExT	0.751	0.084	0.152	0.808	0.077	0.141

(h) Metrics for UCEC data. (531 samples | 2029 Intogen-Intogen pairs)

Method	Precision	Sensitivity	F1 Score	$\text{Precision}_{\text{strict}}$	$\text{Sensitivity}_{\text{strict}}$	F1 $_{\text{strict}}$ Score
DISCOVER	0.719	0.171	0.276	0.811	0.155	0.260
Fisher's Exact Test	0.875	0.007	0.014	0.875	0.007	0.014
WExT	0.664	0.257	0.371	0.762	0.228	0.351

Table 44: Results of network-centric ME evaluation framework with $\mathcal{G}$ = Intact (w conf. threshold 0.25), $\mathcal{S}$ = CGC, c= X_2, p_t = 0.05, t=5, robustness_iterations = 100

(a) Metrics for BLCA data. (411 samples | 1439 CGC-CGC pairs)

Method	Precision	Sensitivity	F1 Score	$\text{Precision}_{\text{strict}}$	$\text{Sensitivity}_{\text{strict}}$	F1 $_{\text{strict}}$ Score
DISCOVER	1.000	0.013	0.025	1.000	0.013	0.026
Fisher's Exact Test	1.000	0.004	0.008	1.000	0.004	0.008
WExT	0.833	0.021	0.041	0.833	0.021	0.041

(b) Metrics for BRCA data. (1026 samples | 1238 CGC-CGC pairs)

Method	Precision	Sensitivity	F1 Score	$\text{Precision}_{\text{strict}}$	$\text{Sensitivity}_{\text{strict}}$	F1 $_{\text{strict}}$ Score
DISCOVER	0.827	0.027	0.053	0.827	0.027	0.052
DISCOVER Strat	0.927	0.031	0.059	0.927	0.031	0.060
Fisher's Exact Test	1.000	0.003	0.006	1.000	0.003	0.006
WExT	0.746	0.058	0.108	0.771	0.056	0.104

(c) Metrics for COADREAD data. (498 samples | 2494 CGC-CGC pairs)

Method	Precision	Sensitivity	F1 Score	$\text{Precision}_{\text{strict}}$	$\text{Sensitivity}_{\text{strict}}$	F1 $_{\text{strict}}$ Score
DISCOVER	0.702	0.043	0.081	0.736	0.040	0.076
DISCOVER Strat	0.659	0.012	0.023	0.659	0.012	0.024
Fisher's Exact Test	0.667	0.009	0.017	0.677	0.008	0.016
WExT	0.668	0.104	0.180	0.709	0.092	0.163

(d) Metrics for LUAD data. (568 samples | 1862 CGC-CGC pairs)

Method	Precision	Sensitivity	F1 Score	$\text{Precision}_{\text{strict}}$	$\text{Sensitivity}_{\text{strict}}$	F1 $_{\text{strict}}$ Score
DISCOVER	0.848	0.015	0.030	0.875	0.015	0.029
Fisher's Exact Test	1.000	0.002	0.004	1.000	0.002	0.004
WExT	0.818	0.030	0.058	0.840	0.030	0.058

(e) Metrics for LUSC data. (485 samples | 1417 CGC-CGC pairs)

Method	Precision	Sensitivity	F1 Score	$\text{Precision}_{\text{strict}}$	$\text{Sensitivity}_{\text{strict}}$	F1 $_{\text{strict}}$ Score
DISCOVER	1.000	0.001	0.003	1.000	0.001	0.002
Fisher's Exact Test	1.000	0.001	0.003	1.000	0.001	0.002
WExT	0.696	0.006	0.011	0.696	0.006	0.012

(f) Metrics for SKCM data. (468 samples | 3113 CGC-CGC pairs)

Method	Precision	Sensitivity	F1 Score	$\text{Precision}_{\text{strict}}$	$\text{Sensitivity}_{\text{strict}}$	F1 $_{\text{strict}}$ Score
DISCOVER	0.839	0.013	0.025	0.839	0.013	0.026
Fisher's Exact Test	1.000	0.001	0.001	1.000	0.001	0.002
WExT	0.766	0.036	0.069	0.775	0.036	0.069

(g) Metrics for STAD data. (438 samples | 2194 CGC-CGC pairs)

Method	Precision	Sensitivity	F1 Score	$\text{Precision}_{\text{strict}}$	$\text{Sensitivity}_{\text{strict}}$	F1 $_{\text{strict}}$ Score
DISCOVER	0.768	0.029	0.055	0.803	0.028	0.054
Fisher's Exact Test	0.889	0.004	0.007	0.889	0.004	0.008
WExT	0.729	0.062	0.115	0.794	0.056	0.105

(h) Metrics for UCEC data. (531 samples | 4046 CGC-CGC pairs)

Method	Precision	Sensitivity	F1 Score	$\text{Precision}_{\text{strict}}$	$\text{Sensitivity}_{\text{strict}}$	F1 $_{\text{strict}}$ Score
DISCOVER	0.702	0.116	0.200	0.776	0.105	0.185
Fisher's Exact Test	0.875	0.003	0.007	0.875	0.003	0.006
WExT	0.657	0.201	0.308	0.727	0.179	0.287

Table 45: Results of network-centric ME evaluation framework with $\mathcal{G}$ = Intact (w conf. threshold 0.45), $\mathcal{S}$ = CGC, c= X_2, p_t = 0.05, t=5, robustness_iterations = 100

(a) Metrics for BLCA data. (411 samples | 359 CGC-CGC pairs)

Method	Precision	Sensitivity	F1 Score	$\text{Precision}_{\text{strict}}$	$\text{Sensitivity}_{\text{strict}}$	F1 $_{\text{strict}}$ Score
DISCOVER	1.000	0.011	0.022	1.000	0.011	0.022
Fisher's Exact Test	1.000	0.006	0.011	1.000	0.006	0.012
WExT	0.667	0.017	0.033	0.667	0.017	0.033

(b) Metrics for BRCA data. (1026 samples | 286 CGC-CGC pairs)

Method	Precision	Sensitivity	F1 Score	$\text{Precision}_{\text{strict}}$	$\text{Sensitivity}_{\text{strict}}$	F1 $_{\text{strict}}$ Score
DISCOVER	0.571	0.014	0.027	0.571	0.014	0.027
DISCOVER Strat	0.727	0.028	0.054	0.727	0.028	0.054
Fisher's Exact Test	1.000	0.003	0.007	1.000	0.003	0.006
WExT	0.649	0.065	0.119	0.636	0.062	0.113

(c) Metrics for COADREAD data. (498 samples | 605 CGC-CGC pairs)

Method	Precision	Sensitivity	F1 Score	$\text{Precision}_{\text{strict}}$	$\text{Sensitivity}_{\text{strict}}$	F1 $_{\text{strict}}$ Score
DISCOVER	0.712	0.061	0.113	0.739	0.056	0.104
DISCOVER Strat	0.643	0.015	0.029	0.643	0.015	0.029
Fisher's Exact Test	0.545	0.010	0.020	0.545	0.010	0.020
WExT	0.704	0.135	0.227	0.767	0.115	0.200

(d) Metrics for LUAD data. (568 samples | 448 CGC-CGC pairs)

Method	Precision	Sensitivity	F1 Score	$\text{Precision}_{\text{strict}}$	$\text{Sensitivity}_{\text{strict}}$	F1 $_{\text{strict}}$ Score
DISCOVER	0.857	0.013	0.026	0.857	0.013	0.026
Fisher's Exact Test	NaN	0.000	NaN	NaN	0.000	NaN
WExT	0.778	0.032	0.061	0.778	0.032	0.061

(e) Metrics for LUSC data. (485 samples | 359 CGC-CGC pairs)

Method	Precision	Sensitivity	F1 Score	$\text{Precision}_{\text{strict}}$	$\text{Sensitivity}_{\text{strict}}$	F1 $_{\text{strict}}$ Score
DISCOVER	1.000	0.006	0.011	1.000	0.006	0.012
Fisher's Exact Test	0.667	0.006	0.011	0.667	0.006	0.012
WExT	0.714	0.014	0.027	0.714	0.014	0.027

(f) Metrics for SKCM data. (468 samples | 860 CGC-CGC pairs)

Method	Precision	Sensitivity	F1 Score	$\text{Precision}_{\text{strict}}$	$\text{Sensitivity}_{\text{strict}}$	F1 $_{\text{strict}}$ Score
DISCOVER	0.857	0.014	0.028	0.857	0.014	0.028
Fisher's Exact Test	NaN	0.000	NaN	NaN	0.000	NaN
WExT	0.844	0.045	0.085	0.844	0.045	0.085

(g) Metrics for STAD data. (438 samples | 545 CGC-CGC pairs)

Method	Precision	Sensitivity	F1 Score	$\text{Precision}_{\text{strict}}$	$\text{Sensitivity}_{\text{strict}}$	F1 $_{\text{strict}}$ Score
DISCOVER	0.690	0.037	0.070	0.731	0.035	0.067
Fisher's Exact Test	0.667	0.004	0.007	0.667	0.004	0.008
WExT	0.652	0.083	0.147	0.696	0.072	0.130

(h) Metrics for UCEC data. (531 samples | 1096 CGC-CGC pairs)

Method	Precision	Sensitivity	F1 Score	$\text{Precision}_{\text{strict}}$	$\text{Sensitivity}_{\text{strict}}$	F1 $_{\text{strict}}$ Score
DISCOVER	0.683	0.152	0.249	0.762	0.135	0.229
Fisher's Exact Test	0.800	0.007	0.014	0.800	0.007	0.014
WExT	0.632	0.226	0.333	0.708	0.192	0.302

Table 46: Results of network-centric ME evaluation framework with $\mathcal{G}$ = HINT, $\mathcal{S}$ = CGC, c= X_2, p_t = 0.05, t=5, robustness_iterations = 100

(a) Metrics for BLCA data. (411 samples | 577 CGC-CGC pairs)

Method	Precision	Sensitivity	F1 Score	$\text{Precision}_{\text{strict}}$	$\text{Sensitivity}_{\text{strict}}$	F1 $_{\text{strict}}$ Score
DISCOVER	0.867	0.023	0.044	0.867	0.023	0.045
Fisher's Exact Test	1.000	0.005	0.010	1.000	0.005	0.010
WExT	0.710	0.038	0.073	0.710	0.038	0.072

(b) Metrics for BRCA data. (1026 samples | 427 CGC-CGC pairs)

Method	Precision	Sensitivity	F1 Score	$\text{Precision}_{\text{strict}}$	$\text{Sensitivity}_{\text{strict}}$	F1 $_{\text{strict}}$ Score
DISCOVER	0.800	0.028	0.054	0.800	0.028	0.054
DISCOVER Strat	0.800	0.037	0.072	0.842	0.037	0.071
Fisher's Exact Test	1.000	0.005	0.009	1.000	0.005	0.010
WExT	0.813	0.087	0.157	0.833	0.082	0.149

(c) Metrics for COADREAD data. (498 samples | 1059 CGC-CGC pairs)

Method	Precision	Sensitivity	F1 Score	$\text{Precision}_{\text{strict}}$	$\text{Sensitivity}_{\text{strict}}$	F1 $_{\text{strict}}$ Score
DISCOVER	0.736	0.074	0.135	0.777	0.069	0.127
DISCOVER Strat	0.719	0.030	0.058	0.736	0.030	0.058
Fisher's Exact Test	0.733	0.021	0.040	0.750	0.020	0.039
WExT	0.644	0.143	0.234	0.721	0.126	0.215

(d) Metrics for LUAD data. (568 samples | 748 CGC-CGC pairs)

Method	Precision	Sensitivity	F1 Score	$\text{Precision}_{\text{strict}}$	$\text{Sensitivity}_{\text{strict}}$	F1 $_{\text{strict}}$ Score
DISCOVER	0.583	0.009	0.018	0.583	0.009	0.018
Fisher's Exact Test	1.000	0.001	0.003	1.000	0.001	0.002
WExT	0.733	0.030	0.057	0.724	0.028	0.054

(e) Metrics for LUSC data. (485 samples | 524 CGC-CGC pairs)

Method	Precision	Sensitivity	F1 Score	$\text{Precision}_{\text{strict}}$	$\text{Sensitivity}_{\text{strict}}$	F1 $_{\text{strict}}$ Score
DISCOVER	1.000	0.004	0.008	1.000	0.004	0.008
Fisher's Exact Test	0.667	0.004	0.008	0.667	0.004	0.008
WExT	0.500	0.008	0.015	0.500	0.008	0.016

(f) Metrics for SKCM data. (468 samples | 1324 CGC-CGC pairs)

Method	Precision	Sensitivity	F1 Score	$\text{Precision}_{\text{strict}}$	$\text{Sensitivity}_{\text{strict}}$	F1 $_{\text{strict}}$ Score
DISCOVER	0.875	0.011	0.021	0.875	0.011	0.022
Fisher's Exact Test	NaN	0.000	NaN	NaN	0.000	NaN
WExT	0.816	0.039	0.074	0.816	0.039	0.074

(g) Metrics for STAD data. (438 samples | 854 CGC-CGC pairs)

Method	Precision	Sensitivity	F1 Score	$\text{Precision}_{\text{strict}}$	$\text{Sensitivity}_{\text{strict}}$	F1 $_{\text{strict}}$ Score
DISCOVER	0.619	0.030	0.058	0.658	0.029	0.056
Fisher's Exact Test	0.667	0.002	0.005	0.667	0.002	0.004
WExT	0.692	0.090	0.160	0.735	0.079	0.143

(h) Metrics for UCEC data. (531 samples | 1707 CGC-CGC pairs)

Method	Precision	Sensitivity	F1 Score	$\text{Precision}_{\text{strict}}$	$\text{Sensitivity}_{\text{strict}}$	F1 $_{\text{strict}}$ Score
DISCOVER	0.701	0.160	0.261	0.800	0.145	0.246
Fisher's Exact Test	0.846	0.006	0.013	0.846	0.006	0.012
WExT	0.660	0.254	0.367	0.755	0.225	0.347

Table 47: Results of network-centric ME evaluation framework with $\mathcal{G}$ = STRING, $\mathcal{S}$ = CGC, c= X_2, p_t = 0.05, t=5, robustness_iterations = 100

(a) Metrics for BLCA data. (411 samples | 1210 CGC-CGC pairs)

Method	Precision	Sensitivity	F1 Score	$\text{Precision}_{\text{strict}}$	$\text{Sensitivity}_{\text{strict}}$	F1 $_{\text{strict}}$ Score
DISCOVER	1.000	0.011	0.021	1.000	0.011	0.022
Fisher's Exact Test	1.000	0.003	0.007	1.000	0.003	0.006
WExT	0.724	0.017	0.034	0.724	0.017	0.033

(b) Metrics for BRCA data. (1026 samples | 818 CGC-CGC pairs)

Method	Precision	Sensitivity	F1 Score	$\text{Precision}_{\text{strict}}$	$\text{Sensitivity}_{\text{strict}}$	F1 $_{\text{strict}}$ Score
DISCOVER	0.903	0.034	0.066	0.933	0.034	0.066
DISCOVER Strat	0.968	0.037	0.071	0.968	0.037	0.071
Fisher's Exact Test	NaN	0.000	NaN	NaN	0.000	NaN
WExT	0.794	0.068	0.126	0.857	0.066	0.123

(c) Metrics for COADREAD data. (498 samples | 1906 CGC-CGC pairs)

Method	Precision	Sensitivity	F1 Score	$\text{Precision}_{\text{strict}}$	$\text{Sensitivity}_{\text{strict}}$	F1 $_{\text{strict}}$ Score
DISCOVER	0.742	0.048	0.091	0.796	0.045	0.085
DISCOVER Strat	0.687	0.018	0.035	0.716	0.018	0.035
Fisher's Exact Test	0.857	0.009	0.019	0.857	0.009	0.018
WExT	0.707	0.096	0.168	0.768	0.087	0.156

(d) Metrics for LUAD data. (568 samples | 1330 CGC-CGC pairs)

Method	Precision	Sensitivity	F1 Score	$\text{Precision}_{\text{strict}}$	$\text{Sensitivity}_{\text{strict}}$	F1 $_{\text{strict}}$ Score
DISCOVER	0.792	0.014	0.028	0.826	0.014	0.028
Fisher's Exact Test	1.000	0.008	0.015	1.000	0.008	0.016
WExT	0.721	0.023	0.045	0.763	0.022	0.043

(e) Metrics for LUSC data. (485 samples | 1057 CGC-CGC pairs)

Method	Precision	Sensitivity	F1 Score	$\text{Precision}_{\text{strict}}$	$\text{Sensitivity}_{\text{strict}}$	F1 $_{\text{strict}}$ Score
DISCOVER	1.000	0.003	0.006	1.000	0.003	0.006
Fisher's Exact Test	1.000	0.001	0.002	1.000	0.001	0.002
WExT	0.667	0.004	0.008	0.667	0.004	0.008

(f) Metrics for SKCM data. (468 samples | 2309 CGC-CGC pairs)

Method	Precision	Sensitivity	F1 Score	$\text{Precision}_{\text{strict}}$	$\text{Sensitivity}_{\text{strict}}$	F1 $_{\text{strict}}$ Score
DISCOVER	0.740	0.016	0.032	0.750	0.016	0.031
Fisher's Exact Test	1.000	0.002	0.003	1.000	0.002	0.004
WExT	0.722	0.036	0.069	0.743	0.035	0.067

(g) Metrics for STAD data. (438 samples | 1517 CGC-CGC pairs)

Method	Precision	Sensitivity	F1 Score	$\text{Precision}_{\text{strict}}$	$\text{Sensitivity}_{\text{strict}}$	F1 $_{\text{strict}}$ Score
DISCOVER	0.818	0.018	0.035	0.839	0.017	0.033
Fisher's Exact Test	1.000	0.001	0.003	1.000	0.001	0.002
WExT	0.742	0.039	0.074	0.806	0.037	0.071

(h) Metrics for UCEC data. (531 samples | 2890 CGC-CGC pairs)

Method	Precision	Sensitivity	F1 Score	$\text{Precision}_{\text{strict}}$	$\text{Sensitivity}_{\text{strict}}$	F1 $_{\text{strict}}$ Score
DISCOVER	0.728	0.114	0.197	0.803	0.104	0.184
Fisher's Exact Test	0.923	0.004	0.008	0.923	0.004	0.008
WExT	0.663	0.197	0.304	0.737	0.174	0.282

Table 48: Degree-normalized network-centric evaluations X_2 and $t = 5$

(a) Metrics for BLCA data.

Method	Precision	Sensitivity	F1 Score	$\text{Precision}_{\text{strict}}$	$\text{Sensitivity}_{\text{strict}}$	F1 $_{\text{strict}}$ Score
DISCOVER	1.0	1.0	0.366	1.0	0.267	0.366
Fisher's Exact Test	1.0	1.0	0.133	1.0	0.072	0.133
WExT	0.882	0.976	0.391	0.882	0.294	0.391

(b) Metrics for BRCA data.

Method	Precision	Sensitivity	F1 Score	$\text{Precision}_{\text{strict}}$	$\text{Sensitivity}_{\text{strict}}$	F1 $_{\text{strict}}$ Score
DISCOVER	0.81	0.902	0.478	0.81	0.417	0.478
DISCOVER Strat	0.936	0.989	0.478	0.936	0.409	0.478
Fisher's Exact Test	1.0	1.0	0.373	1.0	0.271	0.373
WExT	0.846	0.911	0.425	0.85	0.343	0.424

(c) Metrics for COADREAD data.

Method	Precision	Sensitivity	F1 Score	$\text{Precision}_{\text{strict}}$	$\text{Sensitivity}_{\text{strict}}$	F1 $_{\text{strict}}$ Score
DISCOVER	0.879	0.953	0.388	0.88	0.277	0.369
DISCOVER Strat	0.774	0.931	0.195	0.774	0.106	0.176
Fisher's Exact Test	0.898	0.968	0.291	0.898	0.195	0.291
WExT	0.815	0.913	0.44	0.819	0.302	0.394

(d) Metrics for LUAD data.

Method	Precision	Sensitivity	F1 Score	$\text{Precision}_{\text{strict}}$	$\text{Sensitivity}_{\text{strict}}$	F1 $_{\text{strict}}$ Score
DISCOVER	0.977	0.993	0.482	0.977	0.377	0.482
Fisher's Exact Test	NaN	NaN	NaN	NaN	NaN	NaN
WExT	0.943	0.981	0.398	0.944	0.299	0.397

(e) Metrics for LUSC data.

Method	Precision	Sensitivity	F1 Score	$\text{Precision}_{\text{strict}}$	$\text{Sensitivity}_{\text{strict}}$	F1 $_{\text{strict}}$ Score
DISCOVER	1.0	1.0	0.667	1.0	0.6	0.667
Fisher's Exact Test	0.667	0.986	0.667	0.667	0.4	0.444
WExT	0.792	0.944	0.418	0.792	0.326	0.418

(f) Metrics for SKCM data.

Method	Precision	Sensitivity	F1 Score	$\text{Precision}_{\text{strict}}$	$\text{Sensitivity}_{\text{strict}}$	F1 $_{\text{strict}}$ Score
DISCOVER	0.812	0.977	0.272	0.812	0.156	0.237
Fisher's Exact Test	1.0	1.0	0.243	1.0	0.139	0.243
WExT	0.82	0.958	0.302	0.821	0.182	0.268

(g) Metrics for STAD data.

Method	Precision	Sensitivity	F1 Score	$\text{Precision}_{\text{strict}}$	$\text{Sensitivity}_{\text{strict}}$	F1 $_{\text{strict}}$ Score
DISCOVER	0.897	0.972	0.317	0.897	0.203	0.302
Fisher's Exact Test	0.889	0.989	0.331	0.889	0.227	0.331
WExT	0.906	0.959	0.42	0.903	0.298	0.399

(h) Metrics for UCEC data.

Method	Precision	Sensitivity	F1 Score	$\text{Precision}_{\text{strict}}$	$\text{Sensitivity}_{\text{strict}}$	F1 $_{\text{strict}}$ Score
DISCOVER	0.908	0.931	0.377	0.915	0.284	0.382
Fisher's Exact Test	0.952	0.992	0.152	0.952	0.086	0.152
WExT	0.813	0.878	0.422	0.827	0.347	0.44

Scatterplots of percentage significance of mutual exclusivity runs vs mutation load association (MLA) when $t = 20$

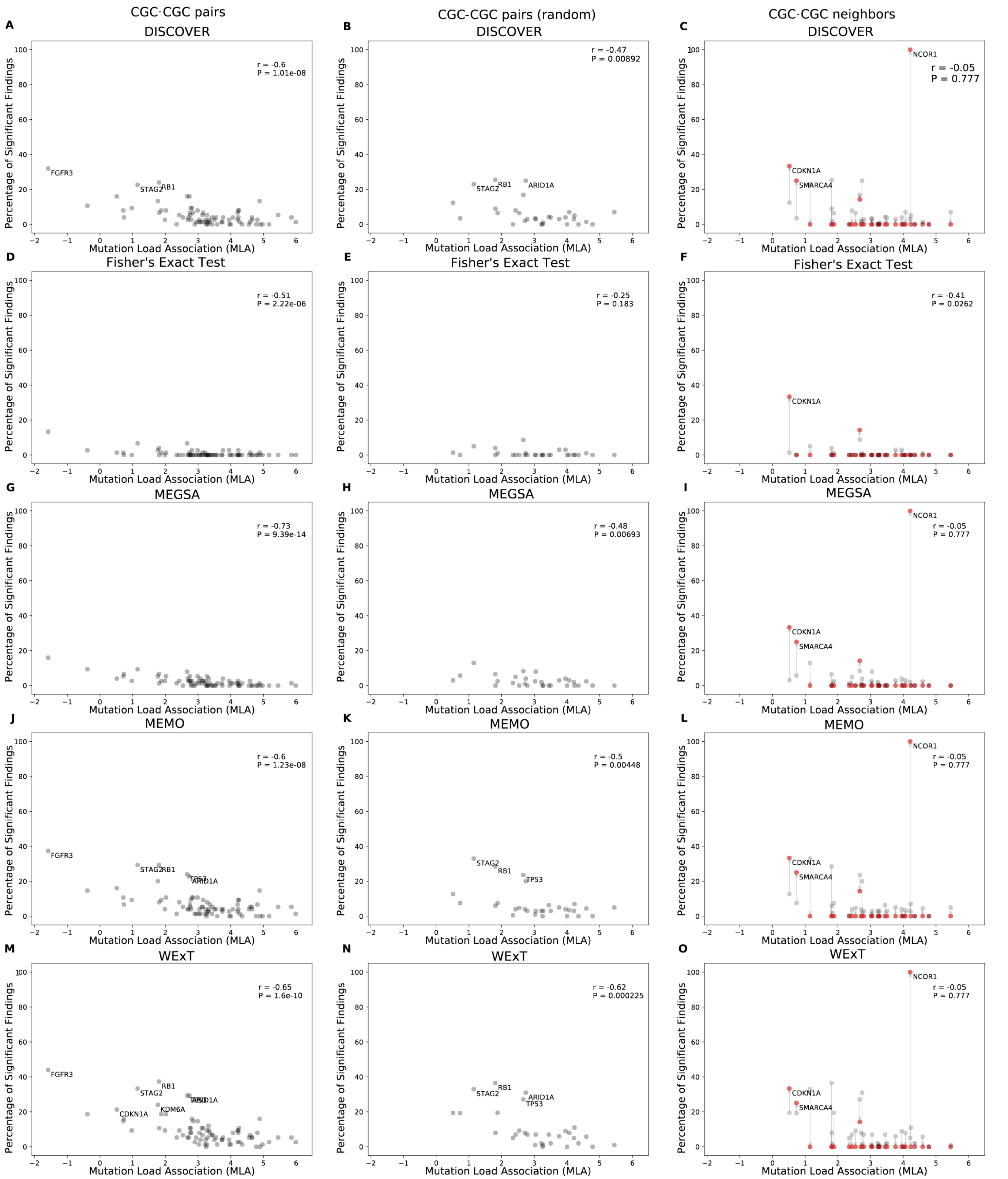

Fig 1: Comparison of ME results of all considered methods on TCGA BLCA cohort with $t = 20$ (411 samples). The scatterplots show the percentage significance of ME runs (p-value<0.05) versus MLA values for CGC genes. (A) Results of DISCOVER where tests are performed between a CGC gene and all other CGC genes. B) Results of DISCOVER where tests are performed between a CGC gene and a random subset of all other CGC genes so that ME of a CGC gene of interest is checked with same sized group of genes in both B and C. (C) Results of DISCOVER where tests are performed between a CGC gene and its PPI neighbors that are in CGC (red) compared with (B) in gray. Analogous results are shown for Fisher's Exact Test (D, E, F), MEGSA (G, H, I), MEMO (J, K, L) and WExT (M, N, O) where coloring is the same as previously described for (C).

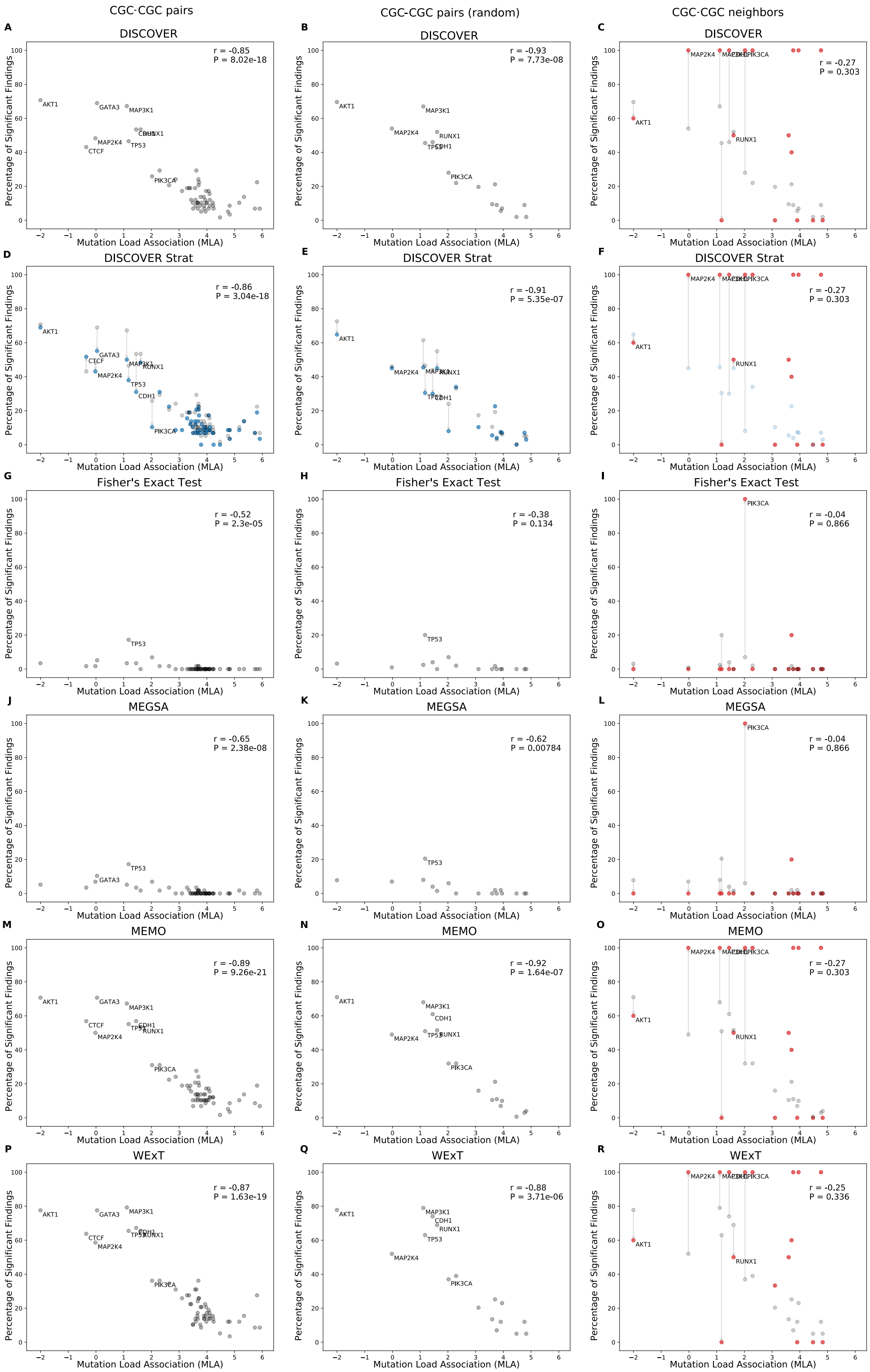

Fig 2: Comparison of ME results of all considered methods on TCGA BRCA cohort with t = 20 (1026 samples). The scatterplots show the percentage significance of ME runs (p-value<0.05) versus MLA values for CGC genes. (A) Results of DISCOVER where tests are performed between a CGC gene and all other CGC genes. B) Results of DISCOVER where tests are performed between a CGC gene and a random subset of all other CGC genes so that ME of a CGC gene of interest is checked with same sized group of genes in both B and C. (C) Results of DISCOVER where tests are performed between a CGC gene and its PPI neighbors that are in CGC (red) compared with (B) in gray. (D) Results of DISCOVER Strat where tests are performed between a CGC gene and all other CGC genes (blue) compared with (A) in gray. (E) Results of DISCOVER Strat where tests are performed between a CGC gene and a random subset of all other CGC genes so that ME of a CGC gene of interest is checked with same sized group of genes in both E and F. Values are shown in blue and compared with (B) in gray. (F) Results of DISCOVER where tests are performed between a CGC gene and its PPI neighbors that are in CGC (red) compared with (E) in blue. Analogous results are shown for Fisher's Exact Test (G, H, I), MEGSA (J, K, L), MEMO (M, N, O) and WExT (P, Q, R) where coloring is the same as previously described for (C).

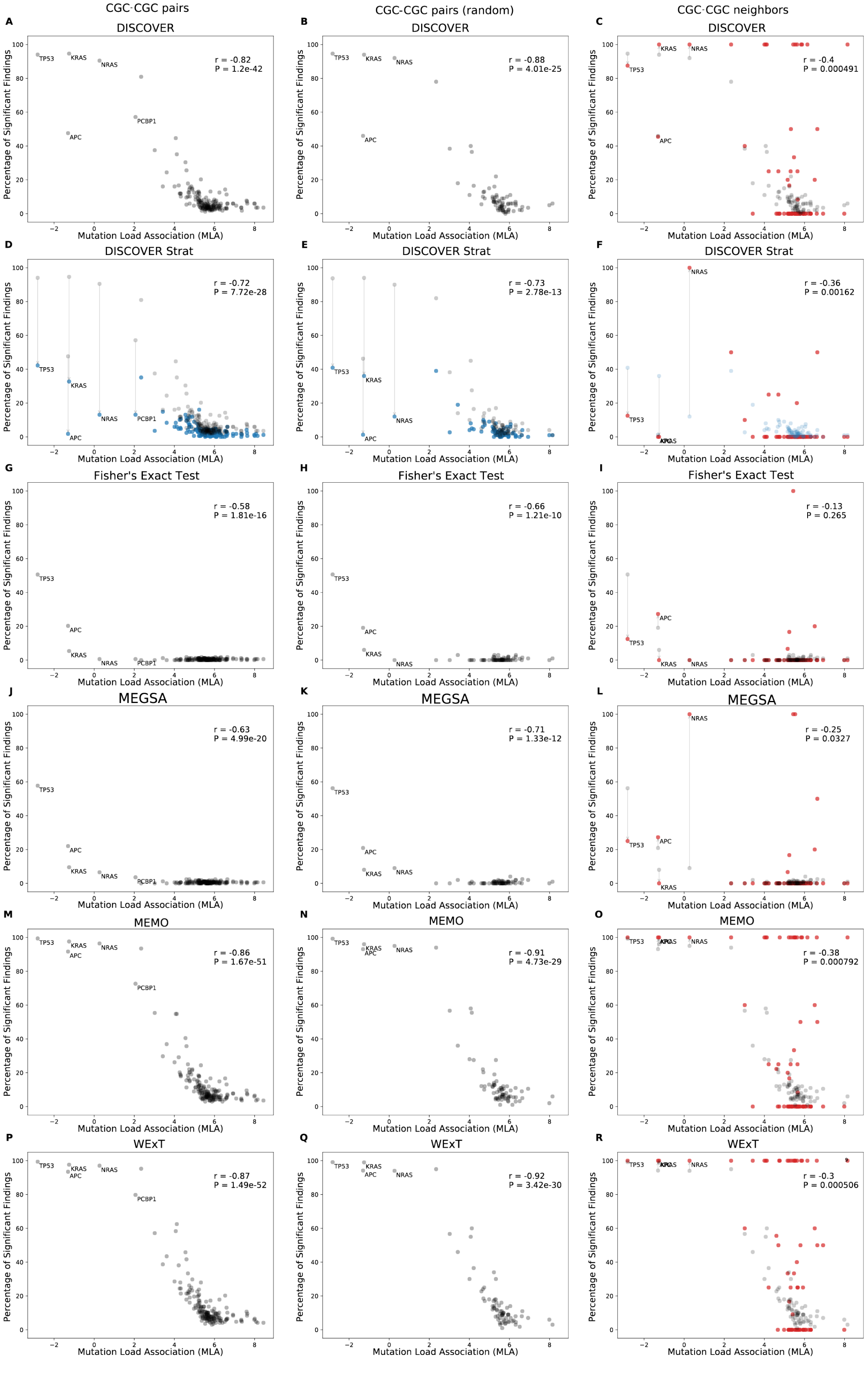

Fig 3: Comparison of ME results of all considered methods on TCGA COADREAD cohort with $t = 20$ (498 samples). The scatterplots show the percentage significance of ME runs (p-value<0.05) versus MLA values for CGC genes. (A) Results of DISCOVER where tests are performed between a CGC gene and all other CGC genes. B) Results of DISCOVER where tests are performed between a CGC gene and a random subset of all other CGC genes so that ME of a CGC gene of interest is checked with same sized group of genes in both B and C. (C) Results of DISCOVER where tests are performed between a CGC gene and its PPI neighbors that are in CGC (red) compared with (B) in gray. (D) Results of DISCOVER Strat where tests are performed between a CGC gene and all other CGC genes (blue) compared with (A) in gray. (E) Results of DISCOVER Strat where tests are performed between a CGC gene and a random subset of all other CGC genes so that ME of a CGC gene of interest is checked with same sized group of genes in both E and F. Values are shown in blue and compared with (B) in gray. (F) Results of DISCOVER where tests are performed between a CGC gene and its PPI neighbors that are in CGC (red) compared with (E) in blue. Analogous results are shown for Fisher's Exact Test (G, H, I), MEGSA (J, K, L), MEMO (M, N, O) and WExT (P, Q, R) where coloring is the same as previously described for (C).

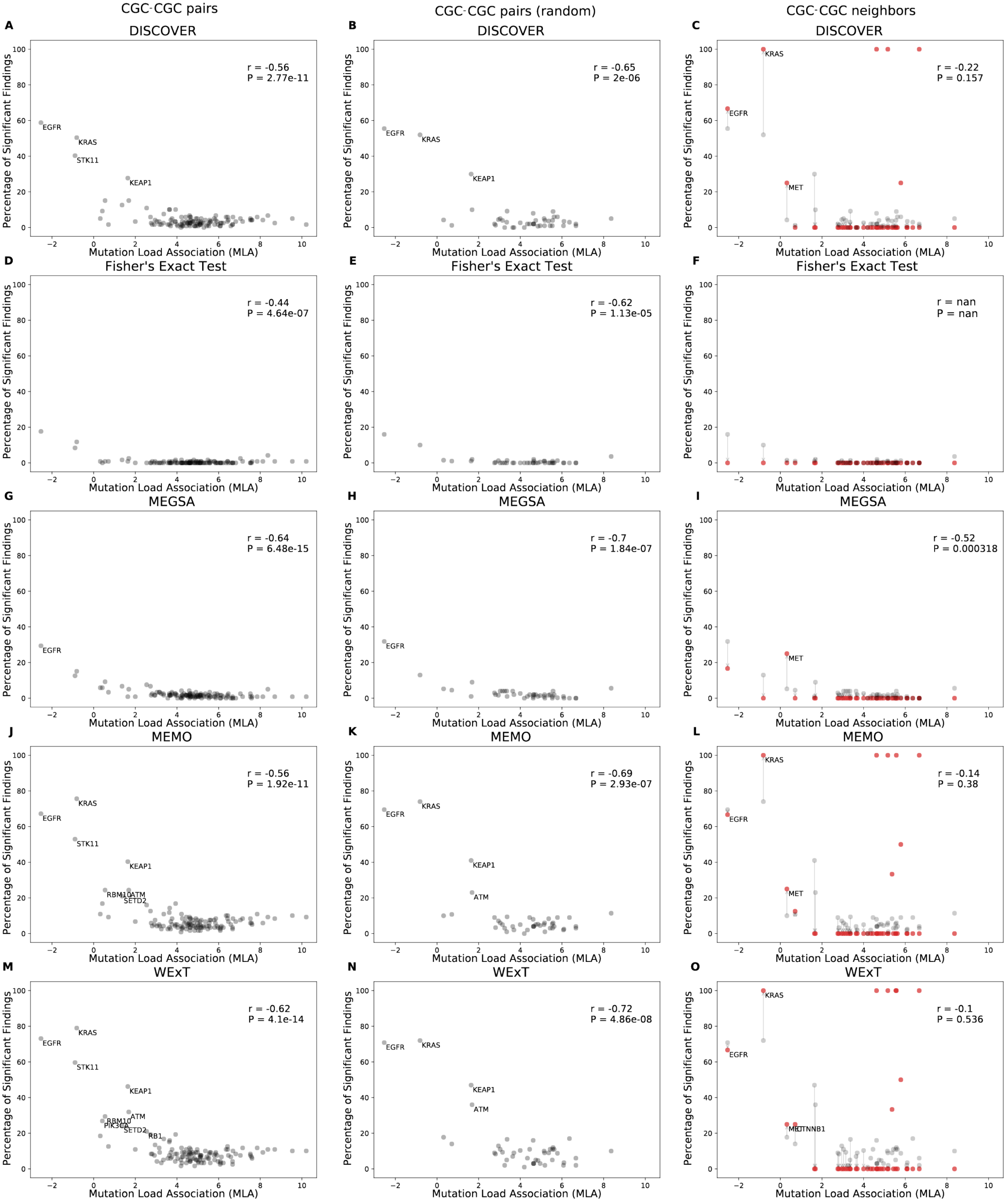

Fig 4: Comparison of ME results of all considered methods on TCGA LUAD cohort with $t = 20$ (568 samples). The scatterplots show the percentage significance of ME runs (p-value<0.05) versus MLA values for CGC genes. (A) Results of DISCOVER where tests are performed between a CGC gene and all other CGC genes. B) Results of DISCOVER where tests are performed between a CGC gene and a random subset of all other CGC genes so that ME of a CGC gene of interest is checked with same sized group of genes in both B and C. (C) Results of DISCOVER where tests are performed between a CGC gene and its PPI neighbors that are in CGC (red) compared with (B) in gray. Analogous results are shown for Fisher's Exact Test (D, E, F), MEGSA (G, H, I), MEMO (J, K, L) and WExT (M, N, O) where coloring is the same as previously described for (C).

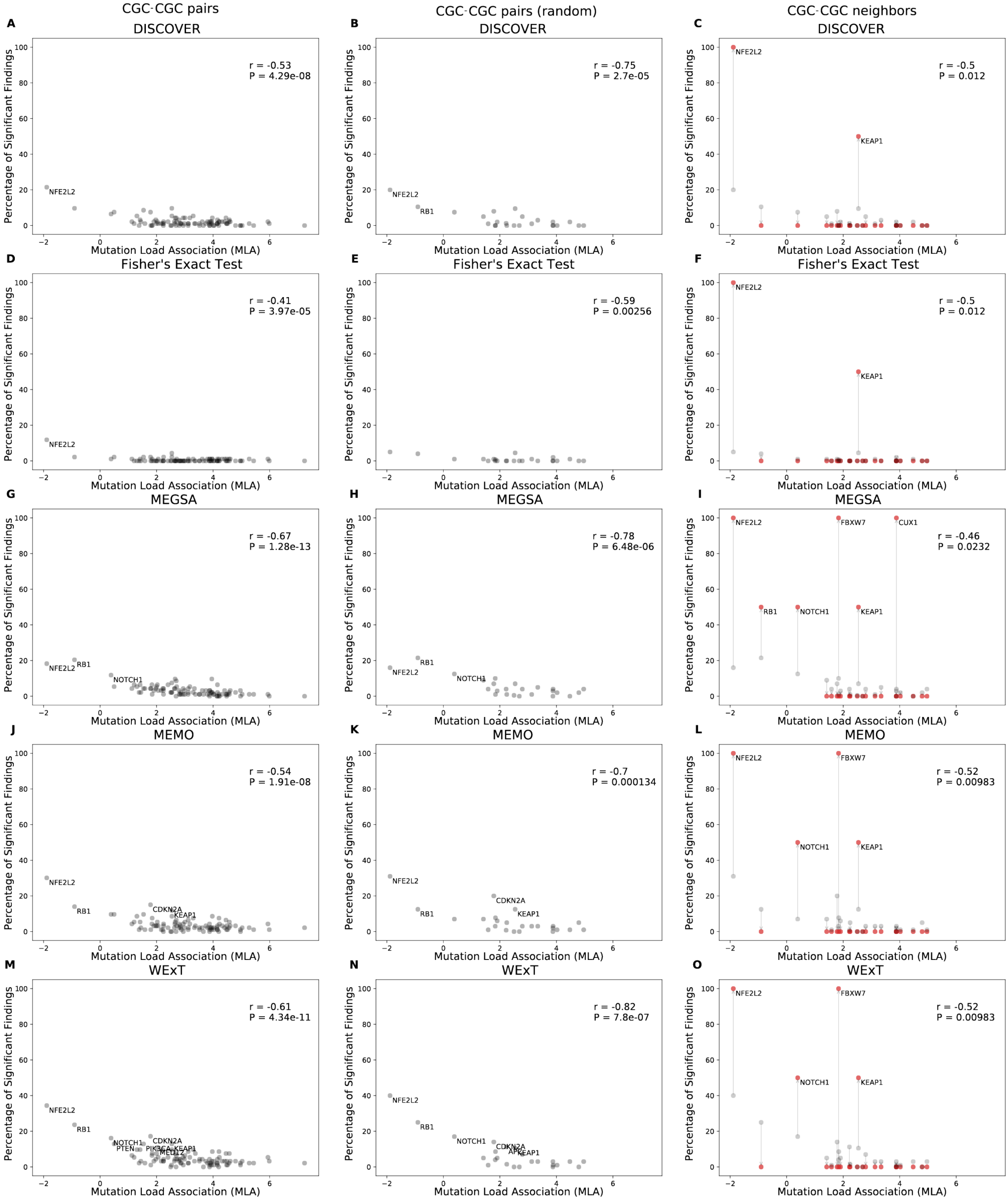

Fig 5: Comparison of ME results of all considered methods on TCGA LUSC cohort with $t = 20$ (485 samples). The scatterplots show the percentage significance of ME runs (p-value<0.05) versus MLA values for CGC genes. (A) Results of DISCOVER where tests are performed between a CGC gene and all other CGC genes. B) Results of DISCOVER where tests are performed between a CGC gene and a random subset of all other CGC genes so that ME of a CGC gene of interest is checked with same sized group of genes in both B and C. (C) Results of DISCOVER where tests are performed between a CGC gene and its PPI neighbors that are in CGC (red) compared with (B) in gray. Analogous results are shown for Fisher's Exact Test (D, E, F), MEGSA (G, H, I), MEMO (J, K, L) and WExT (M, N, O) where coloring is the same as previously described for (C).

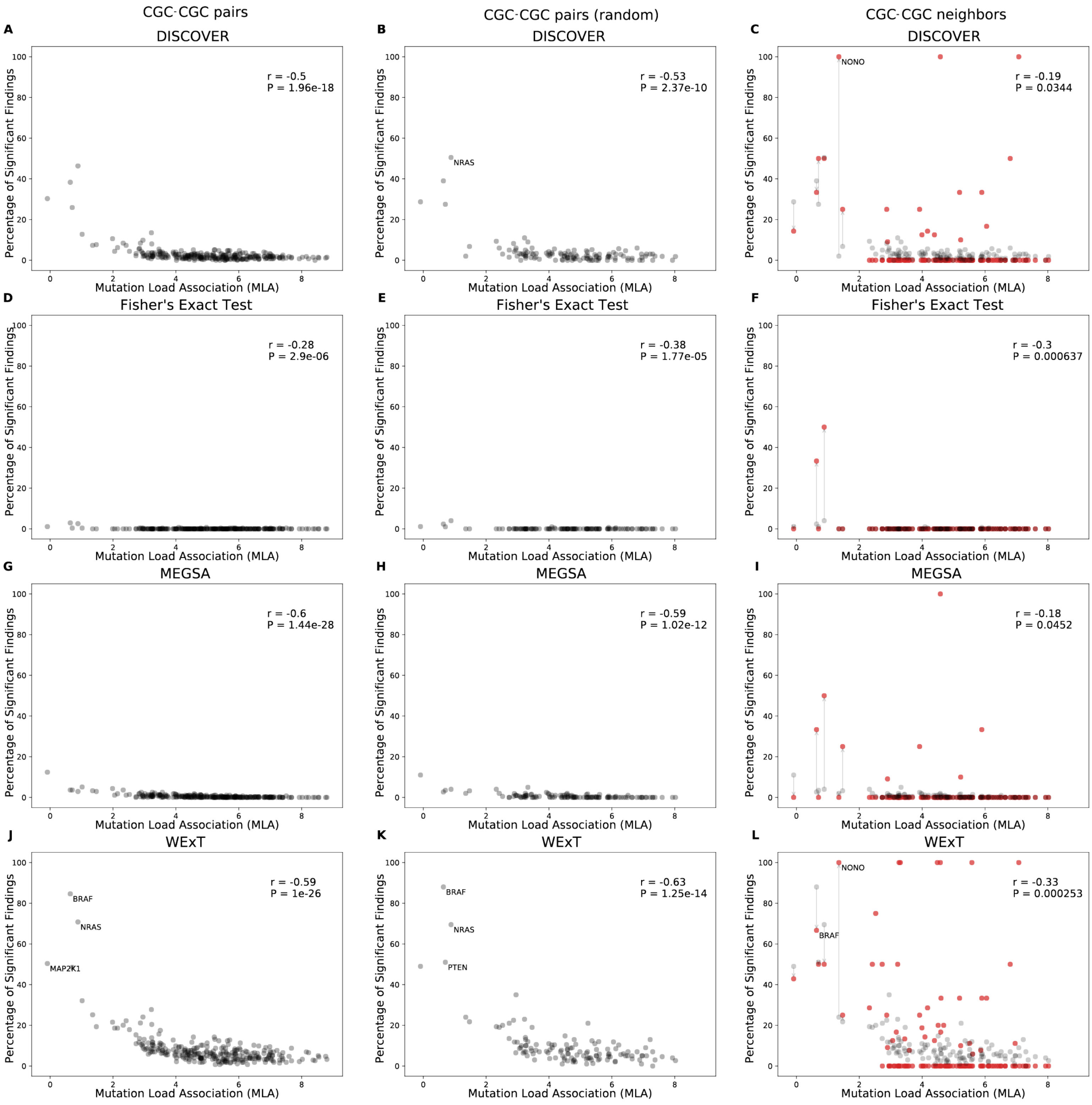

Fig 6: Comparison of ME results of all considered methods on TCGA SKCM cohort with $t = 20$ (468 samples). The scatterplots show the percentage significance of ME runs (p-value<0.05) versus MLA values for CGC genes. (A) Results of DISCOVER where tests are performed between a CGC gene and all other CGC genes. B) Results of DISCOVER where tests are performed between a CGC gene and a random subset of all other CGC genes so that ME of a CGC gene of interest is checked with same sized group of genes in both B and C. (C) Results of DISCOVER where tests are performed between a CGC gene and its PPI neighbors that are in CGC (red) compared with (B) in gray. Analogous results are shown for Fisher's Exact Test (D, E, F), MEGSA (G, H, I), and WExT (J, K, L) where coloring is the same as previously described for (C).

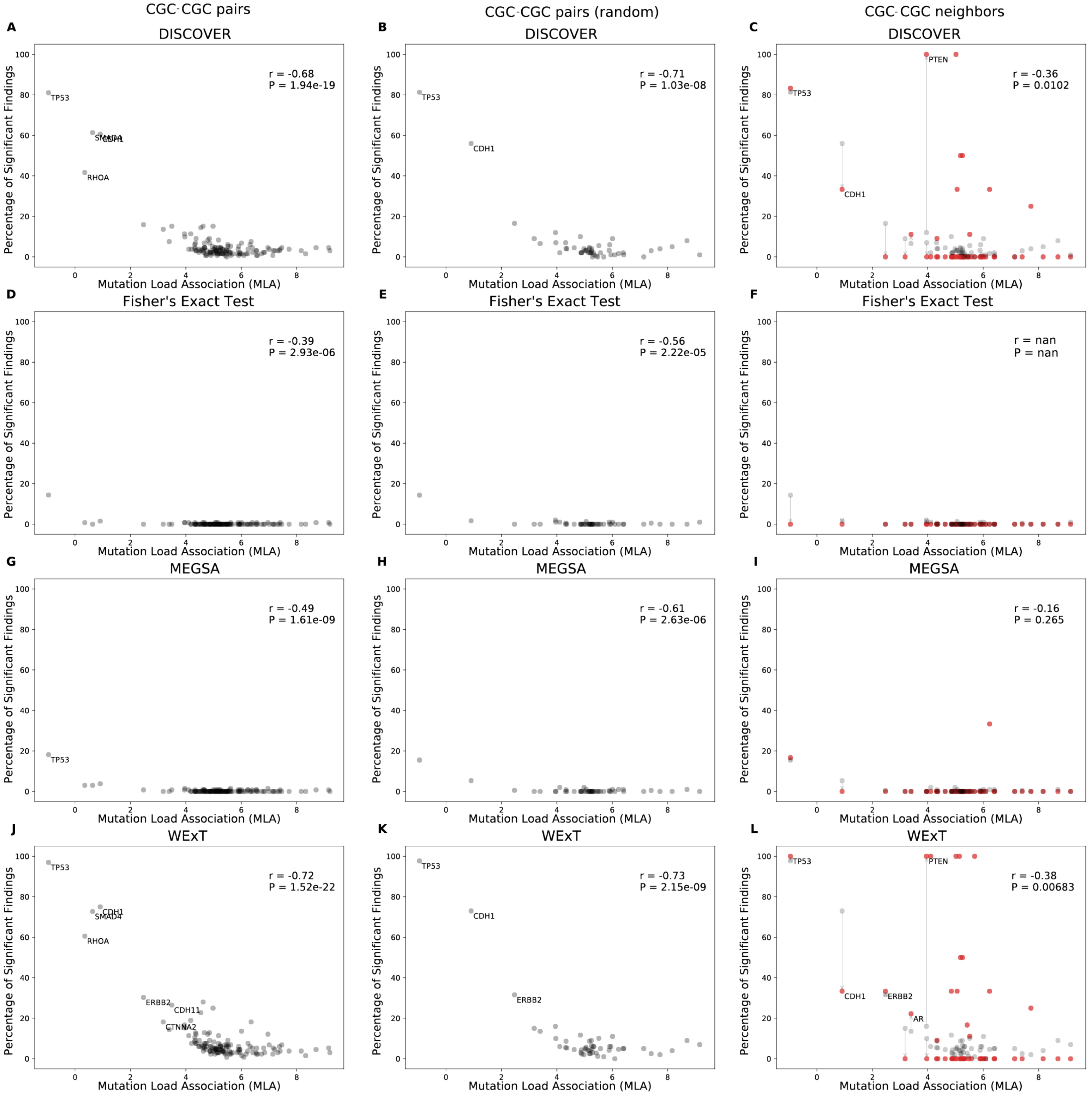

Fig 7: Comparison of ME results of all considered methods on TCGA STAD cohort with $t = 20$ (438 samples). The scatterplots show the percentage significance of ME runs (p-value<0.05) versus MLA values for CGC genes. (A) Results of DISCOVER where tests are performed between a CGC gene and all other CGC genes. B) Results of DISCOVER where tests are performed between a CGC gene and a random subset of all other CGC genes so that ME of a CGC gene of interest is checked with same sized group of genes in both B and C. (C) Results of DISCOVER where tests are performed between a CGC gene and its PPI neighbors that are in CGC (red) compared with (B) in gray. Analogous results are shown for Fisher's Exact Test (D, E, F), MEGSA (G, H, I), and WExT (J, K, L) where coloring is the same as previously described for (C).

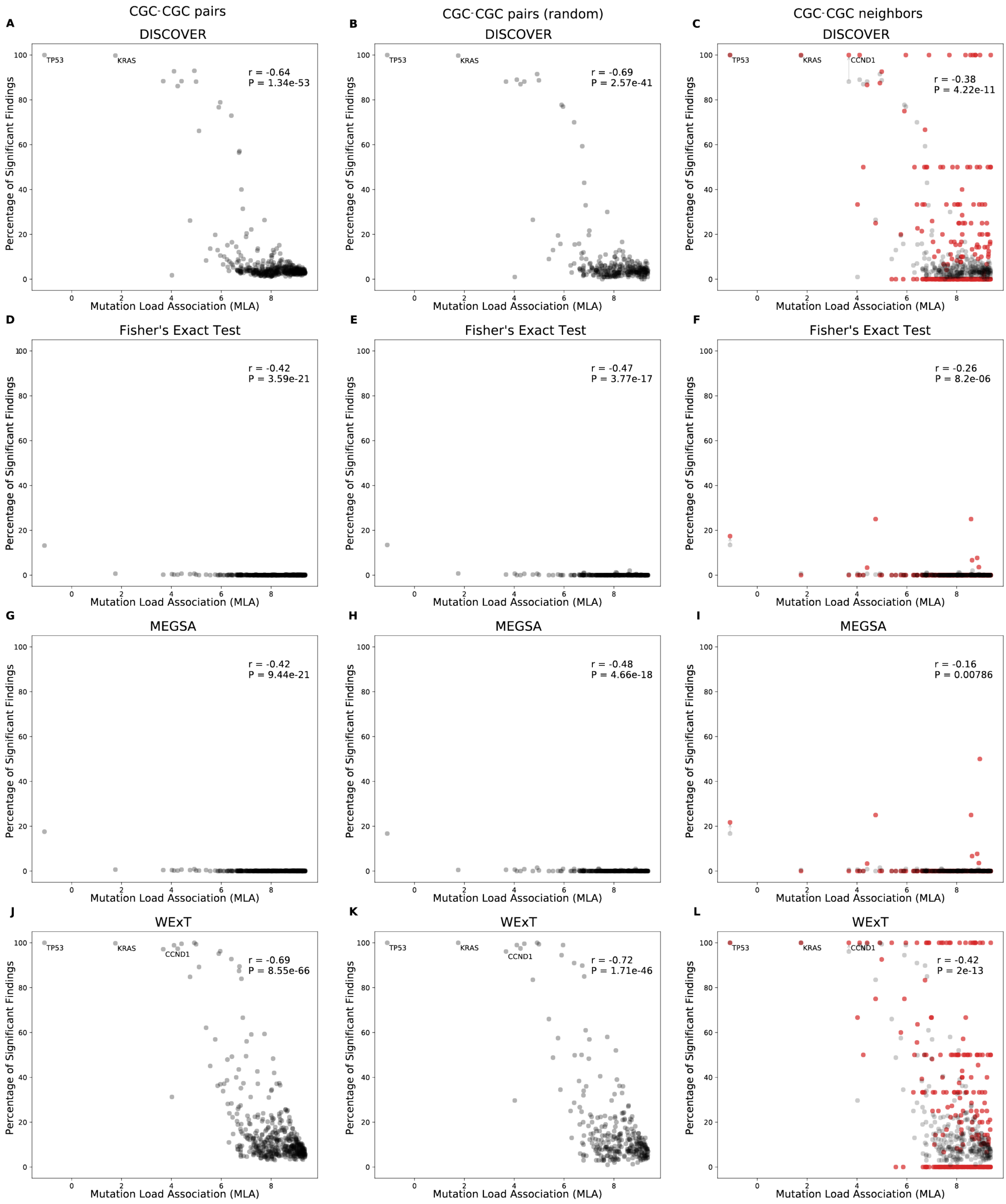

Fig 8: Comparison of ME results of all considered methods on TCGA UCEC cohort with $t = 20$ (531 samples). The scatterplots show the percentage significance of ME runs (p-value<0.05) versus MLA values for CGC genes. (A) Results of DISCOVER where tests are performed between a CGC gene and all other CGC genes. B) Results of DISCOVER where tests are performed between a CGC gene and a random subset of all other CGC genes so that ME of a CGC gene of interest is checked with same sized group of genes in both B and C. (C) Results of DISCOVER where tests are performed between a CGC gene and its PPI neighbors that are in CGC (red) compared with (B) in gray. Analogous results are shown for Fisher's Exact Test (D, E, F), MEGSA (G, H, I), and WExT (J, K, L) where coloring is the same as previously described for (C).

Scatterplots of percentage significance of mutual exclusivity runs vs mutation load association (MLA) when $t = 5$

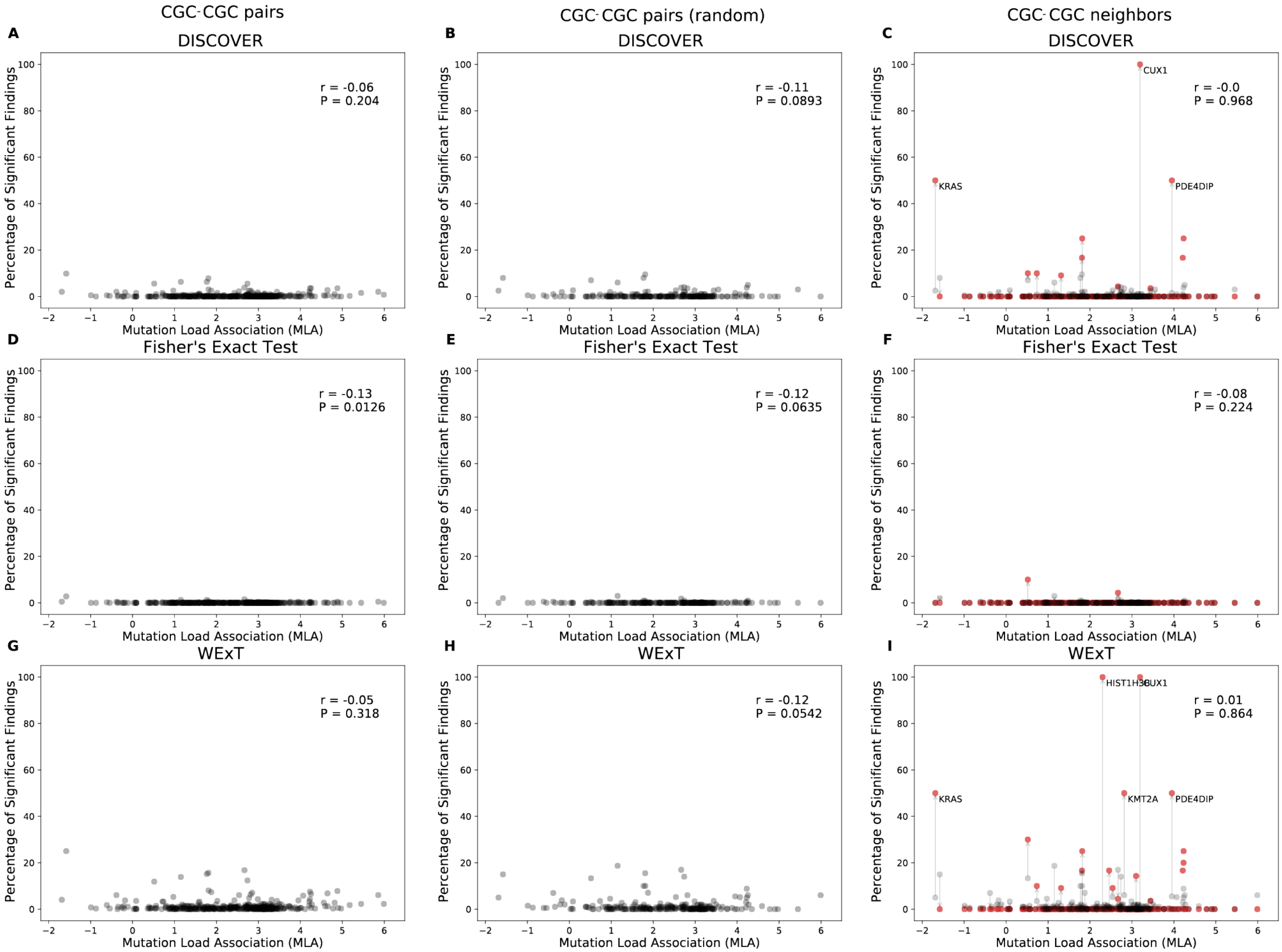

Fig 9: Comparison of ME results of all considered methods on TCGA BLCA cohort with $t = 5$ (411 samples). The scatterplots show the percentage significance of ME runs (p-value<0.05) versus MLA values for CGC genes. (A) Results of DISCOVER where tests are performed between a CGC gene and all other CGC genes. B) Results of DISCOVER where tests are performed between a CGC gene and a random subset of all other CGC genes so that ME of a CGC gene of interest is checked with same sized group of genes in both B and C. (C) Results of DISCOVER where tests are performed between a CGC gene and its PPI neighbors that are in CGC (red) compared with (B) in gray. Analogous results are shown for Fisher's Exact Test (D, E, F), and WExT (G, H, I) where coloring is the same as previously described for (C).

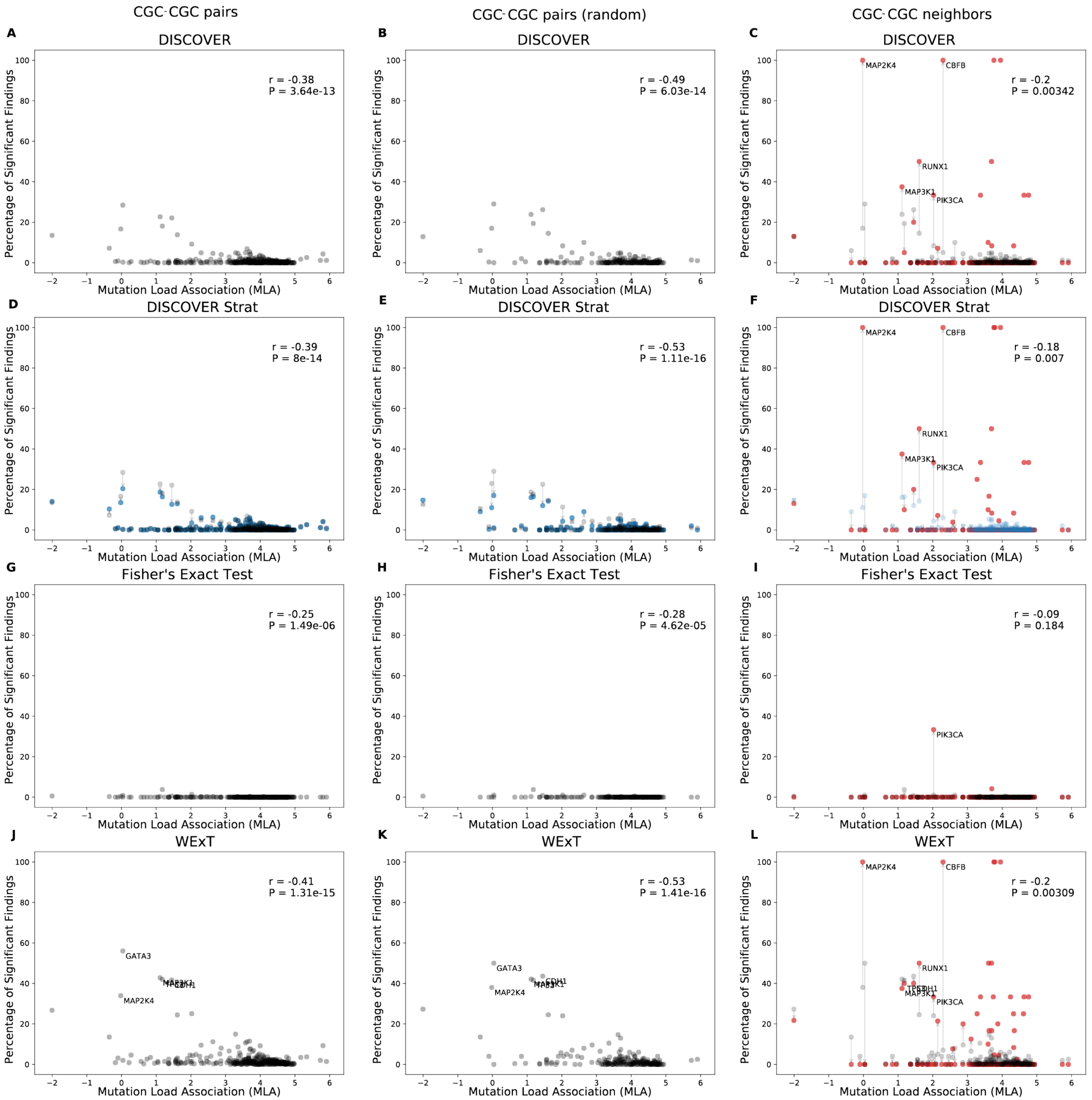

Fig 10: Comparison of ME results of all considered methods on TCGA BRCA cohort with $t = 5$ (1026 samples). The scatterplots show the percentage significance of ME runs (p-value<0.05) versus MLA values for CGC genes. (A) Results of DISCOVER where tests are performed between a CGC gene and all other CGC genes. B) Results of DISCOVER where tests are performed between a CGC gene and a random subset of all other CGC genes so that ME of a CGC gene of interest is checked with same sized group of genes in both B and C. (C) Results of DISCOVER where tests are performed between a CGC gene and its PPI neighbors that are in CGC (red) compared with (B) in gray. (D) Results of DISCOVER Strat where tests are performed between a CGC gene and all other CGC genes (blue) compared with (A) in gray. (E) Results of DISCOVER Strat where tests are performed between a CGC gene and a random subset of all other CGC genes so that ME of a CGC gene of interest is checked with same sized group of genes in both E and F. Values are shown in blue and compared with (B) in gray. (F) Results of DISCOVER where tests are performed between a CGC gene and its PPI neighbors that are in CGC (red) compared with (E) in blue. Analogous results are shown for Fisher's Exact Test (G, H, I), and WExT (J, K, L) where coloring is the same as previously described for (C).

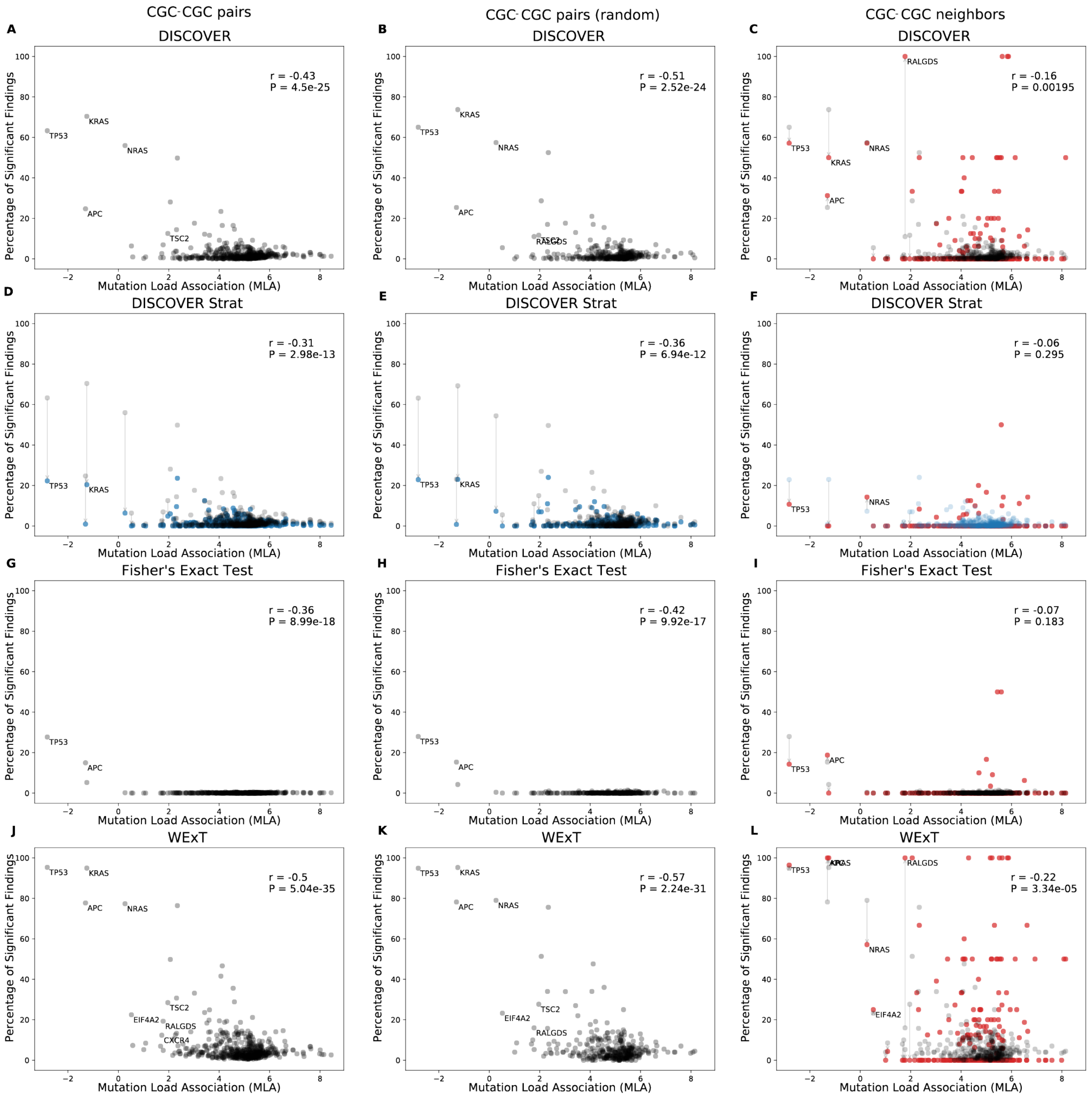

Fig 11: Comparison of ME results of all considered methods on TCGA COADREAD cohort with $t = 5$ (498 samples). The scatterplots show the percentage significance of ME runs (p-value<0.05) versus MLA values for CGC genes. (A) Results of DISCOVER where tests are performed between a CGC gene and all other CGC genes. B) Results of DISCOVER where tests are performed between a CGC gene and a random subset of all other CGC genes so that ME of a CGC gene of interest is checked with same sized group of genes in both B and C. (C) Results of DISCOVER where tests are performed between a CGC gene and its PPI neighbors that are in CGC (red) compared with (B) in gray. (D) Results of DISCOVER Strat where tests are performed between a CGC gene and all other CGC genes (blue) compared with (A) in gray. (E) Results of DISCOVER Strat where tests are performed between a CGC gene and a random subset of all other CGC genes so that ME of a CGC gene of interest is checked with same sized group of genes in both E and F. Values are shown in blue and compared with (B) in gray. (F) Results of DISCOVER where tests are performed between a CGC gene and its PPI neighbors that are in CGC (red) compared with (E) in blue. Analogous results are shown for Fisher's Exact Test (G, H, I), and WExT (J, K, L) where coloring is the same as previously described for (C).

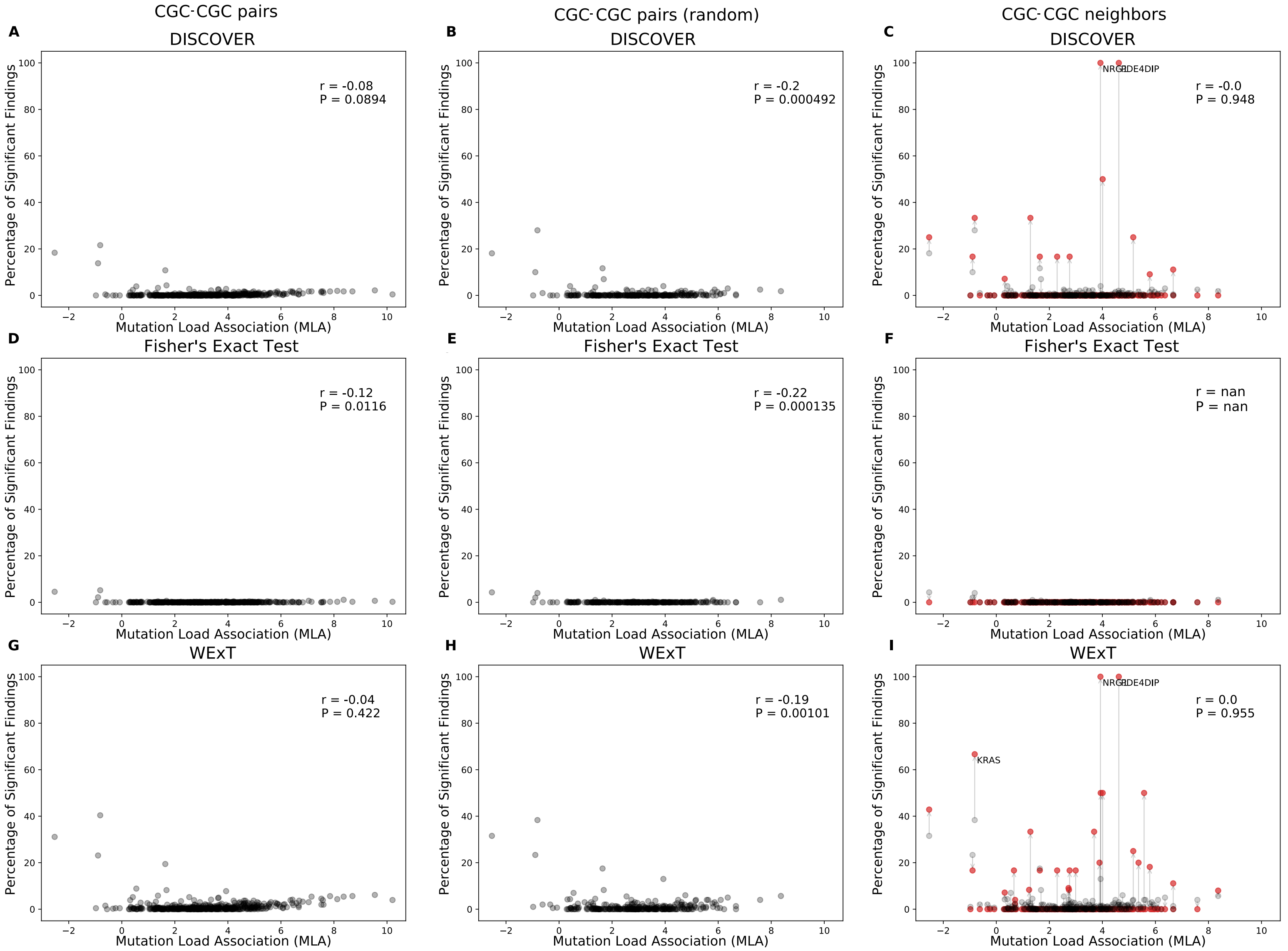

Fig 12: Comparison of ME results of all considered methods on TCGA LUAD cohort with $t = 5$ (568 samples). The scatterplots show the percentage significance of ME runs (p-value<0.05) versus MLA values for CGC genes. (A) Results of DISCOVER where tests are performed between a CGC gene and all other CGC genes. B) Results of DISCOVER where tests are performed between a CGC gene and a random subset of all other CGC genes so that ME of a CGC gene of interest is checked with same sized group of genes in both B and C. (C) Results of DISCOVER where tests are performed between a CGC gene and its PPI neighbors that are in CGC (red) compared with (B) in gray. Analogous results are shown for Fisher's Exact Test (D, E, F), and WExT (G, H, I) where coloring is the same as previously described for (C).

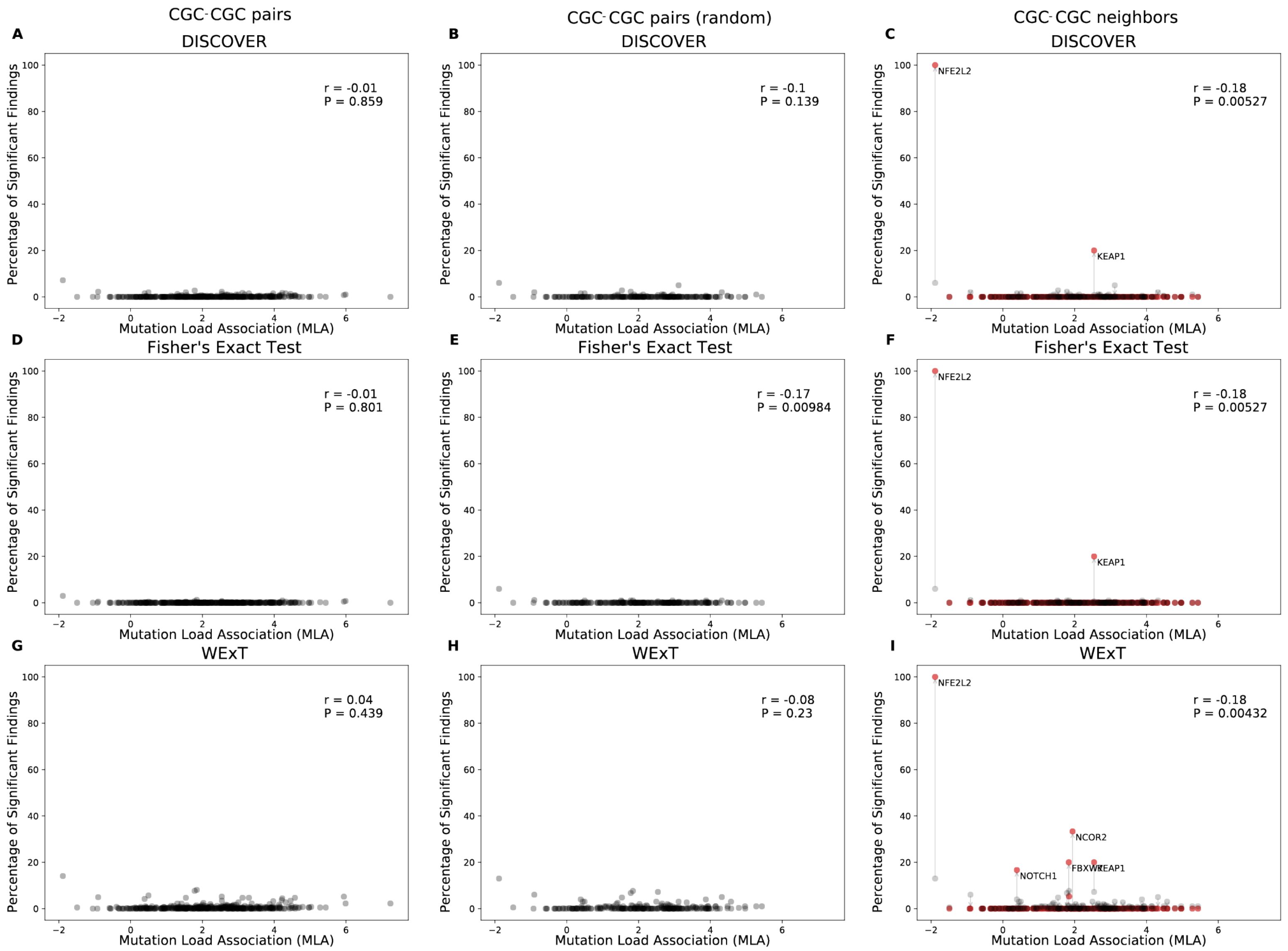

Fig 13: Comparison of ME results of all considered methods on TCGA LUSC cohort with $t = 5$ (485 samples). The scatterplots show the percentage significance of ME runs (p-value<0.05) versus MLA values for CGC genes. (A) Results of DISCOVER where tests are performed between a CGC gene and all other CGC genes. B) Results of DISCOVER where tests are performed between a CGC gene and a random subset of all other CGC genes so that ME of a CGC gene of interest is checked with same sized group of genes in both B and C. (C) Results of DISCOVER where tests are performed between a CGC gene and its PPI neighbors that are in CGC (red) compared with (B) in gray. Analogous results are shown for Fisher's Exact Test (D, E, F), and WExT (G, H, I) where coloring is the same as previously described for (C).

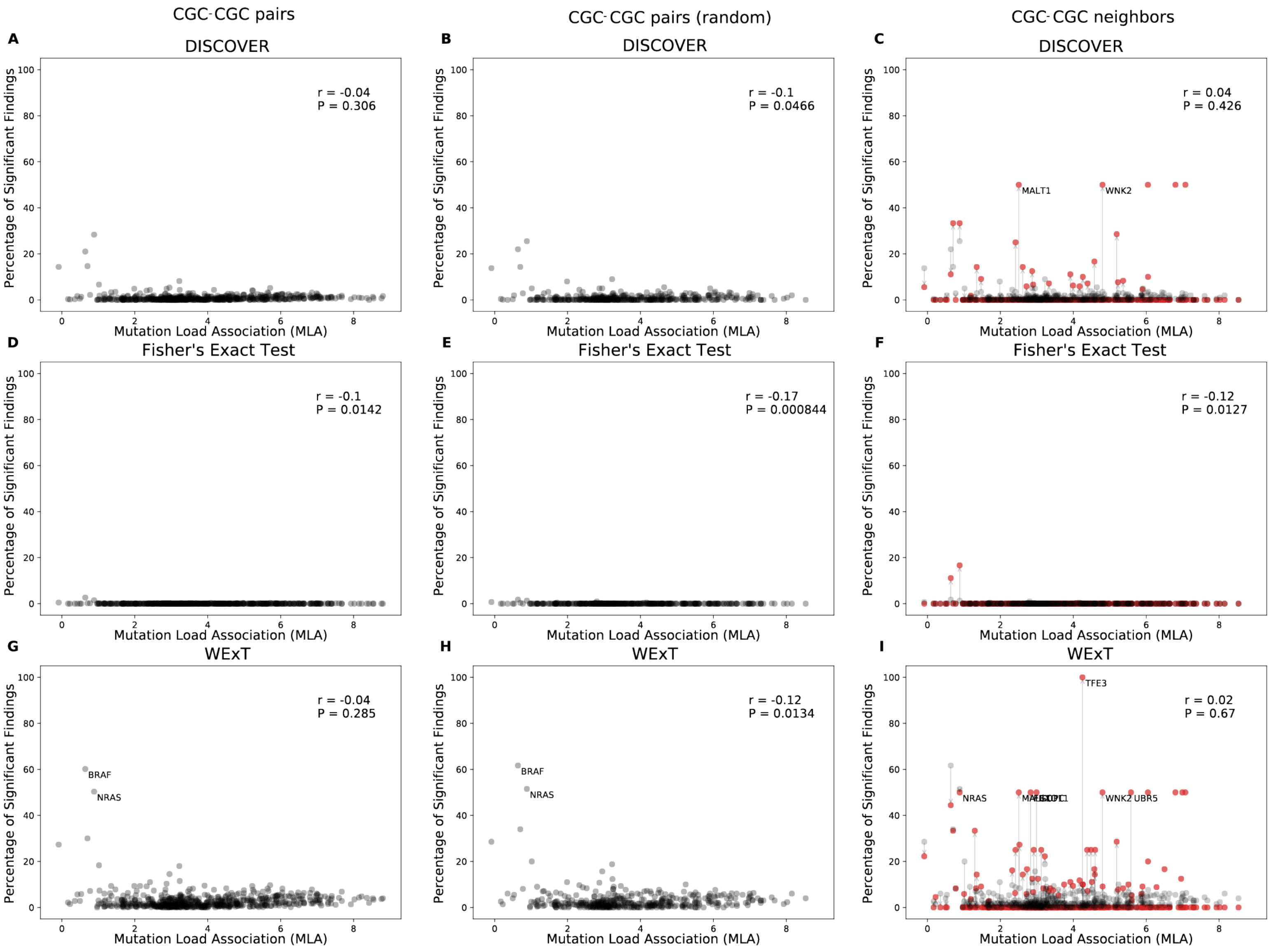

Fig 14: Comparison of ME results of all considered methods on TCGA SKCM cohort with $t = 5$ (468 samples). The scatterplots show the percentage significance of ME runs (p-value<0.05) versus MLA values for CGC genes. (A) Results of DISCOVER where tests are performed between a CGC gene and all other CGC genes. B) Results of DISCOVER where tests are performed between a CGC gene and a random subset of all other CGC genes so that ME of a CGC gene of interest is checked with same sized group of genes in both B and C. (C) Results of DISCOVER where tests are performed between a CGC gene and its PPI neighbors that are in CGC (red) compared with (B) in gray. Analogous results are shown for Fisher's Exact Test (D, E, F), and WExT (G, H, I) where coloring is the same as previously described for (C).

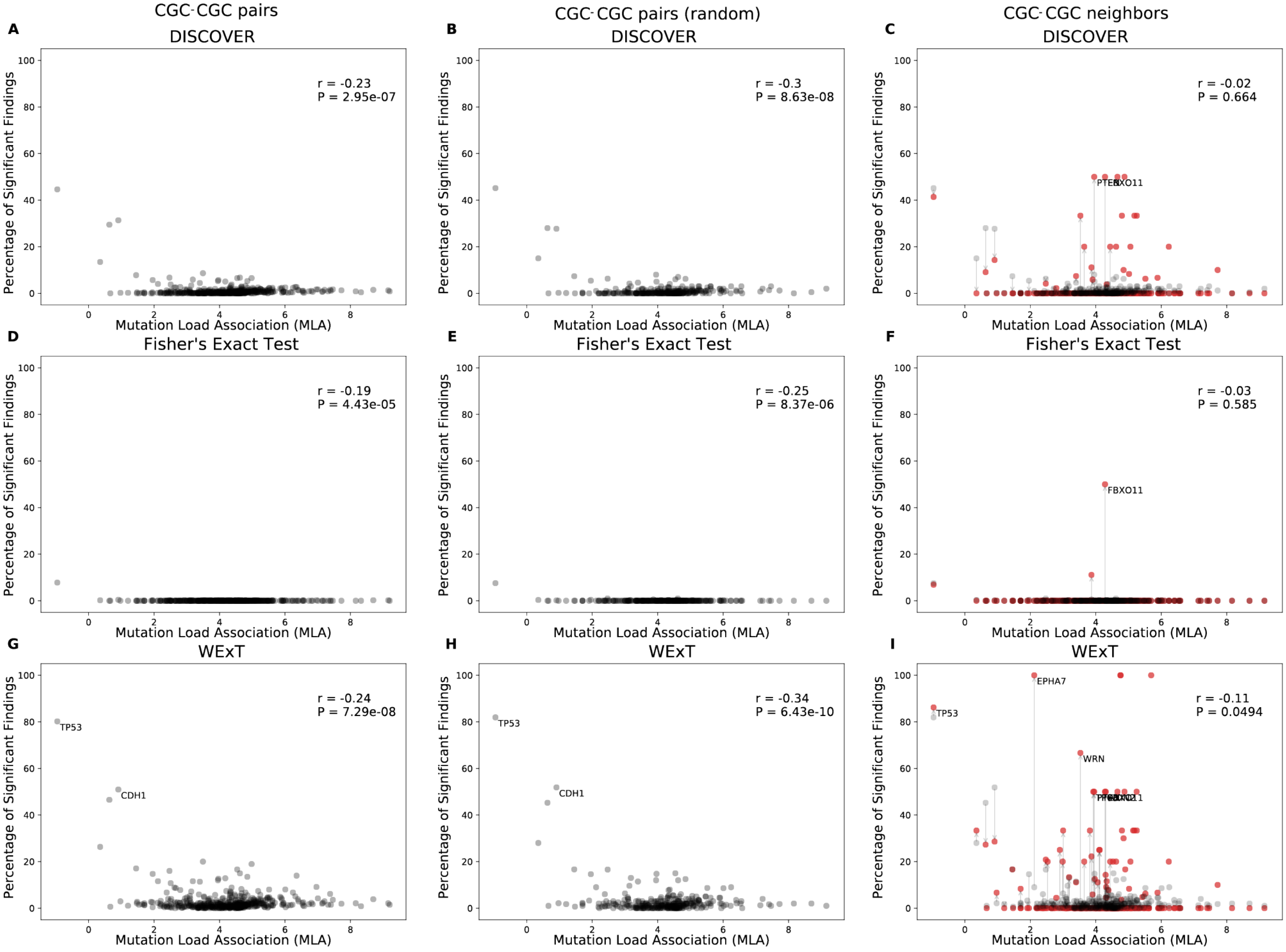

Fig 15: Comparison of ME results of all considered methods on TCGA STAD cohort with $t = 5$ (438 samples). The scatterplots show the percentage significance of ME runs (p-value<0.05) versus MLA values for CGC genes. (A) Results of DISCOVER where tests are performed between a CGC gene and all other CGC genes. B) Results of DISCOVER where tests are performed between a CGC gene and a random subset of all other CGC genes so that ME of a CGC gene of interest is checked with same sized group of genes in both B and C. (C) Results of DISCOVER where tests are performed between a CGC gene and its PPI neighbors that are in CGC (red) compared with (B) in gray. Analogous results are shown for Fisher's Exact Test (D, E, F), and WExT (G, H, I) where coloring is the same as previously described for (C).

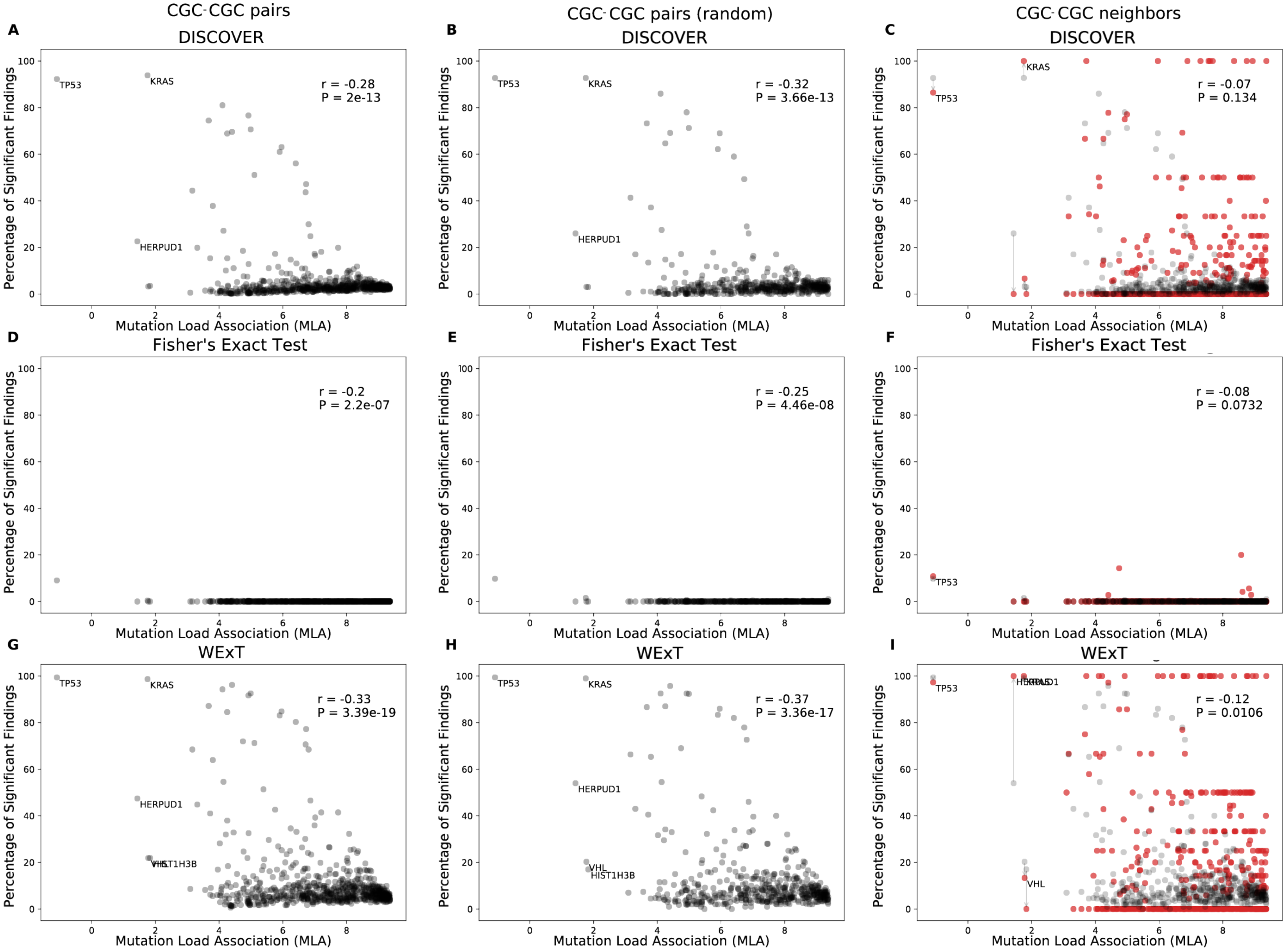

Fig 16: Comparison of ME results of all considered methods on TCGA UCEC cohort with $t = 5$ (531 samples). The scatterplots show the percentage significance of ME runs (p-value<0.05) versus MLA values for CGC genes. (A) Results of DISCOVER where tests are performed between a CGC gene and all other CGC genes. B) Results of DISCOVER where tests are performed between a CGC gene and a random subset of all other CGC genes so that ME of a CGC gene of interest is checked with same sized group of genes in both B and C. (C) Results of DISCOVER where tests are performed between a CGC gene and its PPI neighbors that are in CGC (red) compared with (B) in gray. Analogous results are shown for Fisher's Exact Test (D, E, F), and WExT (G, H, I) where coloring is the same as previously described for (C).

Scatterplots of percentage significance of mutual exclusivity runs vs mutation load association (MLA) when only CGC genes that have > 1 neighbors are included ($t = 20$)

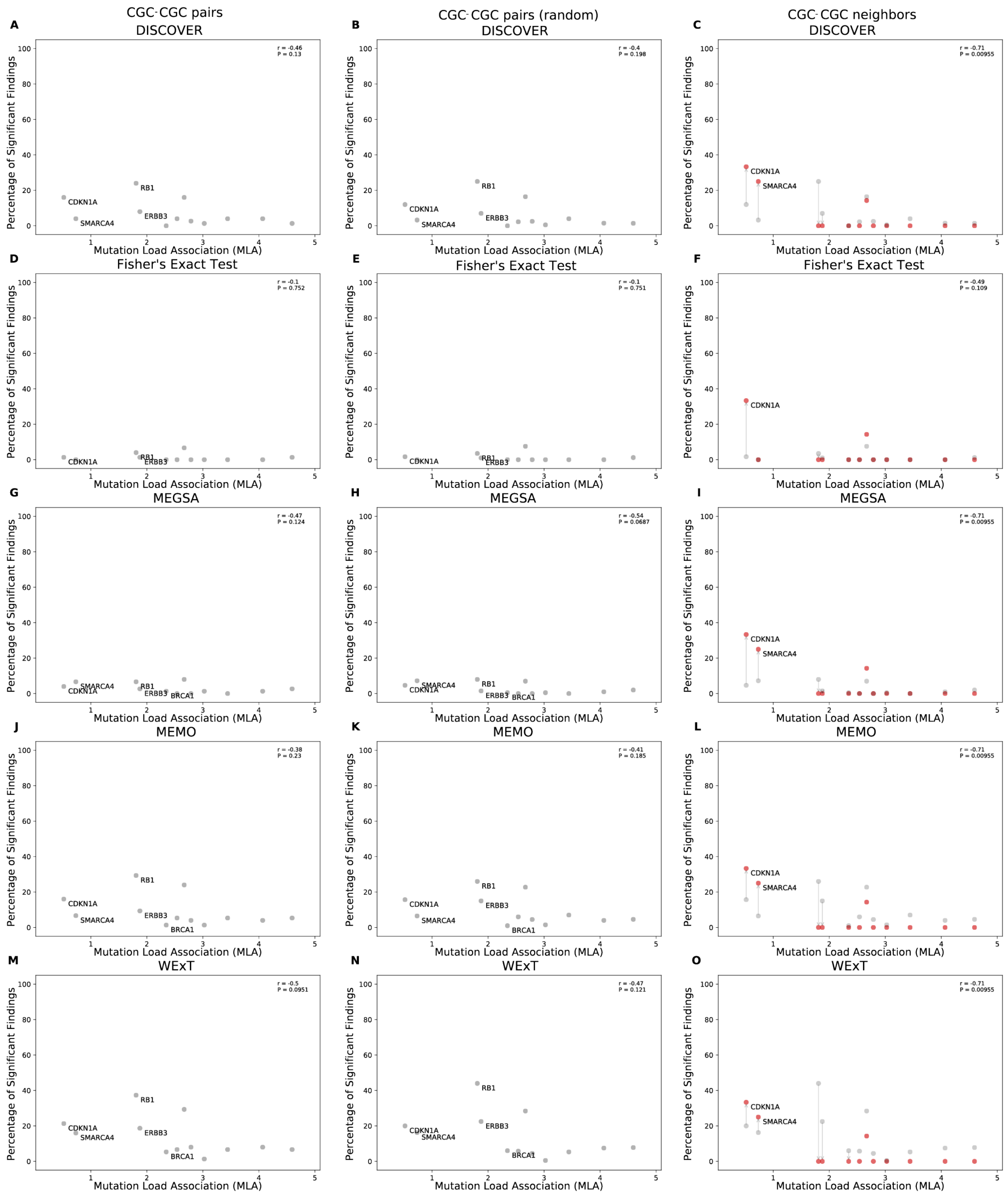

Fig 17: Comparison of ME results of all considered methods on TCGA BLCA cohort with $t = 20$ (411 samples) for CGC genes that have > 1 neighbors. The scatterplots show the percentage significance of ME runs (p-value<0.05) versus MLA values for CGC genes. (A) Results of DISCOVER where tests are performed between a CGC gene and all other CGC genes. B) Results of DISCOVER where tests are performed between a CGC gene and a random subset of all other CGC genes so that ME of a CGC gene of interest is checked with same sized group of genes in both B and C. (C) Results of DISCOVER where tests are performed between a CGC gene and its PPI neighbors that are in CGC (red) compared with (B) in gray. Analogous results are shown for Fisher's Exact Test (D, E, F), MEGSA(G, H, I), MEMO(J, K, L) and WExT (M, N, O) where coloring is the same as previously described for (C).

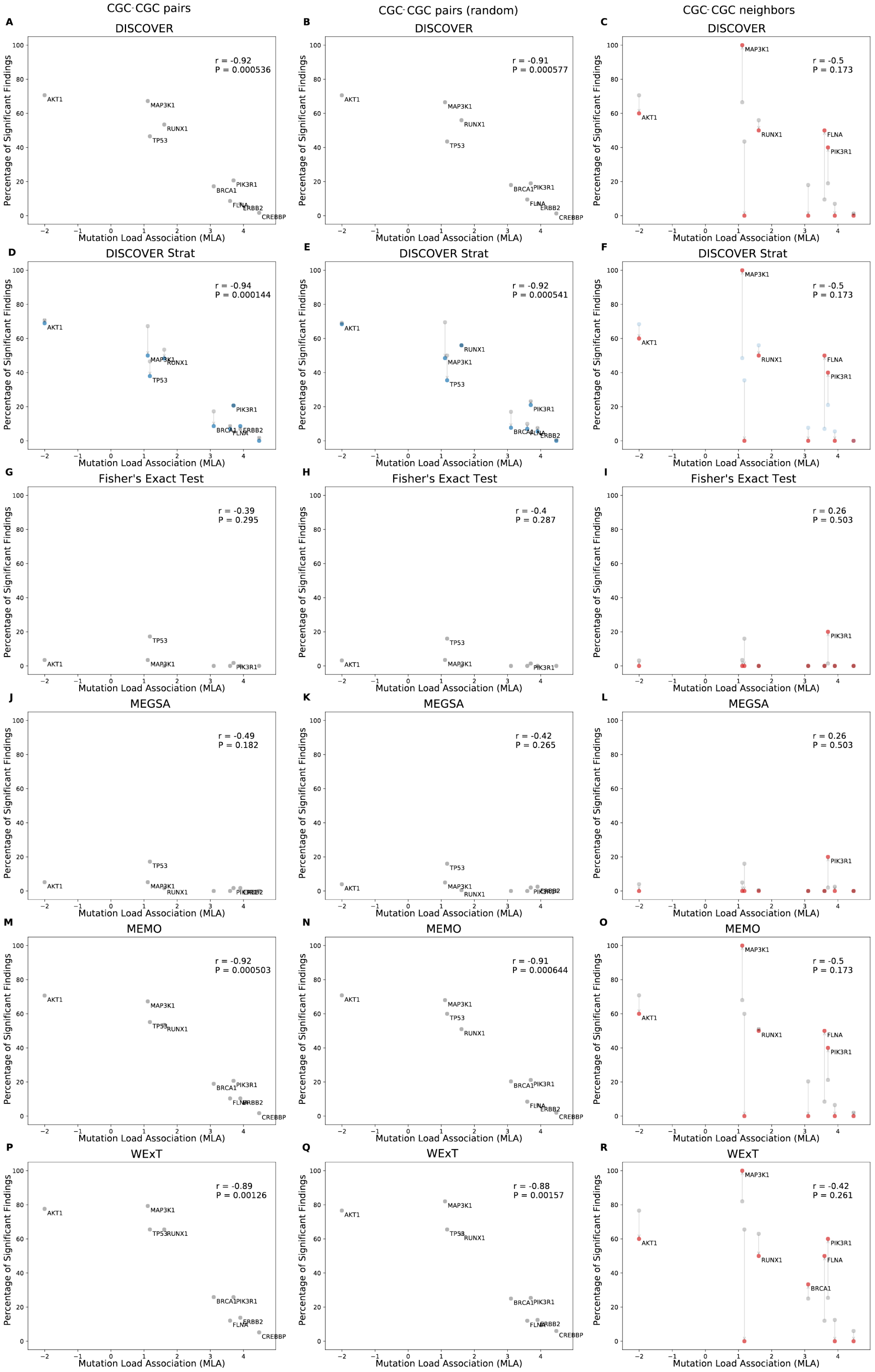

Fig 18: Comparison of ME results of all considered methods on TCGA BRCA cohort with $t = 20$ (1026 samples) for CGC genes that have > 1 neighbors. The scatterplots show the percentage significance of ME runs (p-value<0.05) versus MLA values for CGC genes. (A) Results of DISCOVER where tests are performed between a CGC gene and all other CGC genes. B) Results of DISCOVER where tests are performed between a CGC gene and a random subset of all other CGC genes so that ME of a CGC gene of interest is checked with same sized group of genes in both B and C. (C) Results of DISCOVER where tests are performed between a CGC gene and its PPI neighbors that are in CGC (red) compared with (B) in gray. (D) Results of DISCOVER Strat where tests are performed between a CGC gene and all other CGC genes (blue) compared with (A) in gray. (E) Results of DISCOVER Strat where tests are performed between a CGC gene and a random subset of all other CGC genes so that ME of a CGC gene of interest is checked with same sized group of genes in both E and F. Values are shown in blue and compared with (B) in gray. (F) Results of DISCOVER where tests are performed between a CGC gene and its PPI neighbors that are in CGC (red) compared with (E) in blue. Analogous results are shown for Fisher's Exact Test (G, H, I), MEGSA (J, K, L), MEMO (M, N, O) and WExT (P, Q, R) where coloring is the same as previously described for (C).

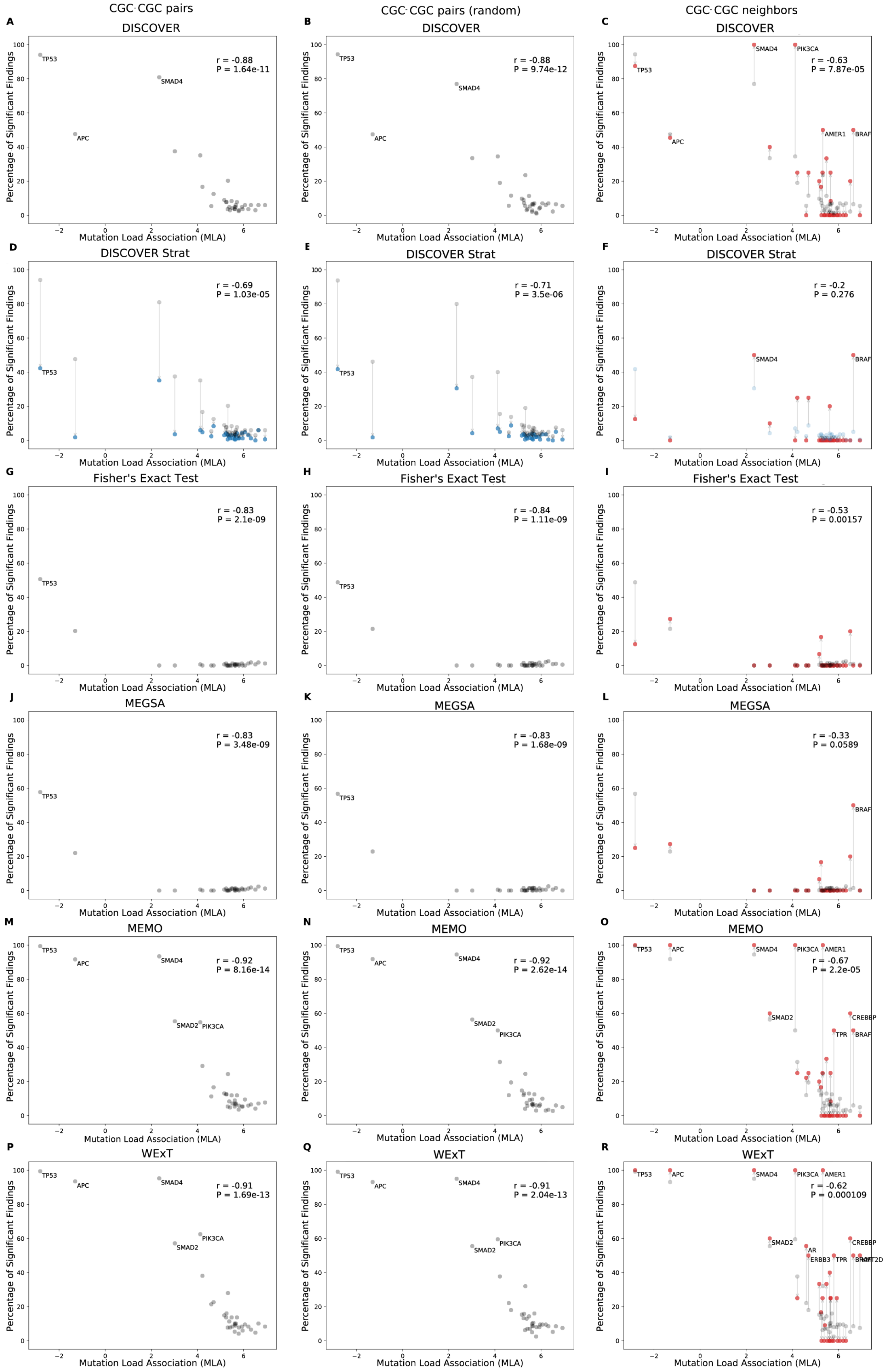

Fig 19: Comparison of ME results of all considered methods on TCGA COADREAD cohort with $t = 20$ (498 samples) for CGC genes that have > 1 neighbors. The scatterplots show the percentage significance of ME runs (p-value<0.05) versus MLA values for CGC genes. (A) Results of DISCOVER where tests are performed between a CGC gene and all other CGC genes. B) Results of DISCOVER where tests are performed between a CGC gene and a random subset of all other CGC genes so that ME of a CGC gene of interest is checked with same sized group of genes in both B and C. (C) Results of DISCOVER where tests are performed between a CGC gene and its PPI neighbors that are in CGC (red) compared with (B) in gray. (D) Results of DISCOVER Strat where tests are performed between a CGC gene and all other CGC genes (blue) compared with (A) in gray. (E) Results of DISCOVER Strat where tests are performed between a CGC gene and a random subset of all other CGC genes so that ME of a CGC gene of interest is checked with same sized group of genes in both E and F. Values are shown in blue and compared with (B) in gray. (F) Results of DISCOVER where tests are performed between a CGC gene and its PPI neighbors that are in CGC (red) compared with (E) in blue. Analogous results are shown for Fisher's Exact Test (G, H, I), MEGSA(J, K, L), MEMO (M, N, O) and WExT (P, Q, R) where coloring is the same as previously described for (C).

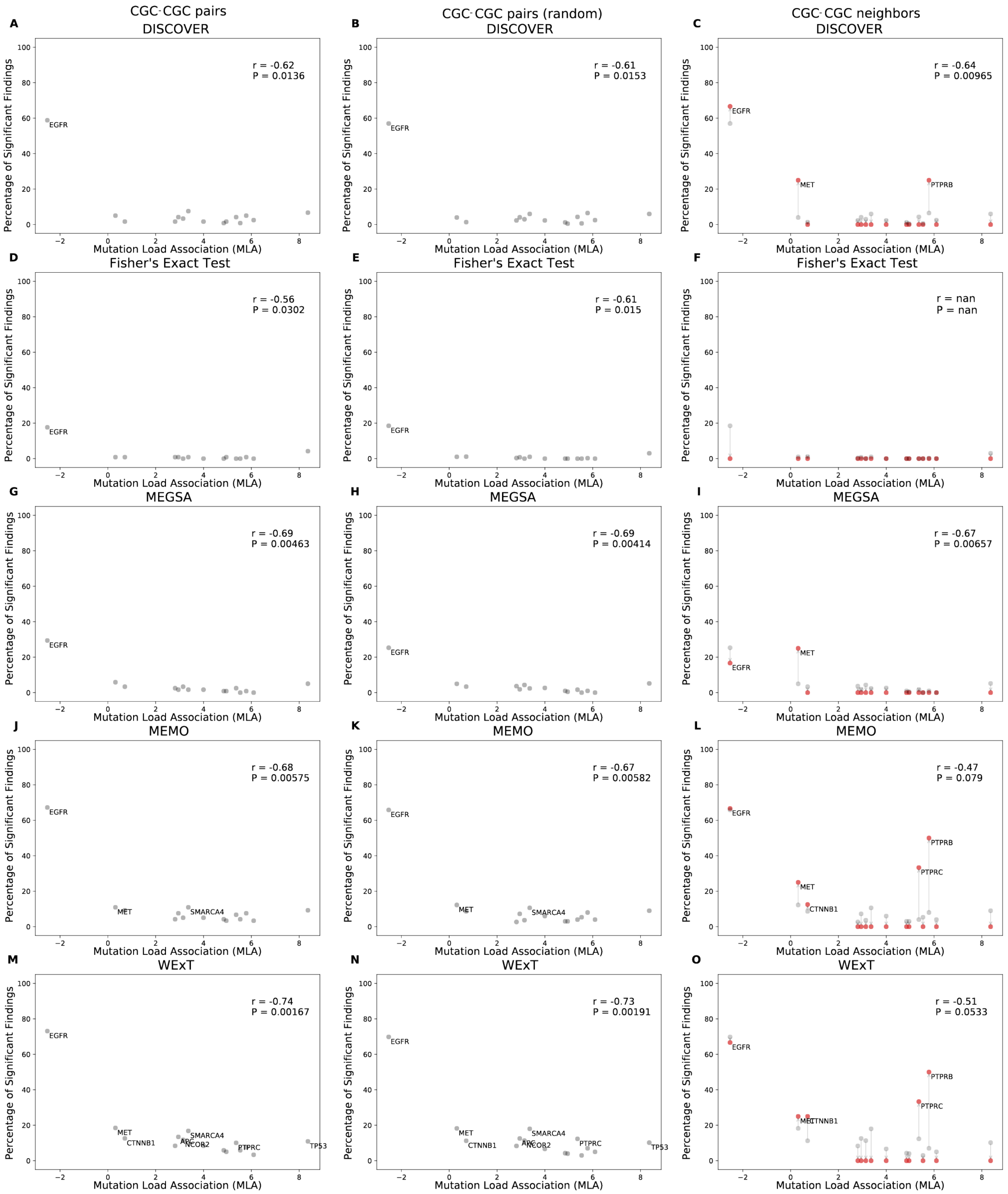

Fig 20: Comparison of ME results of all considered methods on TCGA LUAD cohort with $t = 20$ (568 samples) for CGC genes that have > 1 neighbors. The scatterplots show the percentage significance of ME runs (p-value<0.05) versus MLA values for CGC genes. (A) Results of DISCOVER where tests are performed between a CGC gene and all other CGC genes. B) Results of DISCOVER where tests are performed between a CGC gene and a random subset of all other CGC genes so that ME of a CGC gene of interest is checked with same sized group of genes in both B and C. (C) Results of DISCOVER where tests are performed between a CGC gene and its PPI neighbors that are in CGC (red) compared with (B) in gray. Analogous results are shown for Fisher's Exact Test (D, E, F), MEGSA (G, H, I), MEMO (J, K, L) and WExT (M, N, O) where coloring is the same as previously described for (C).

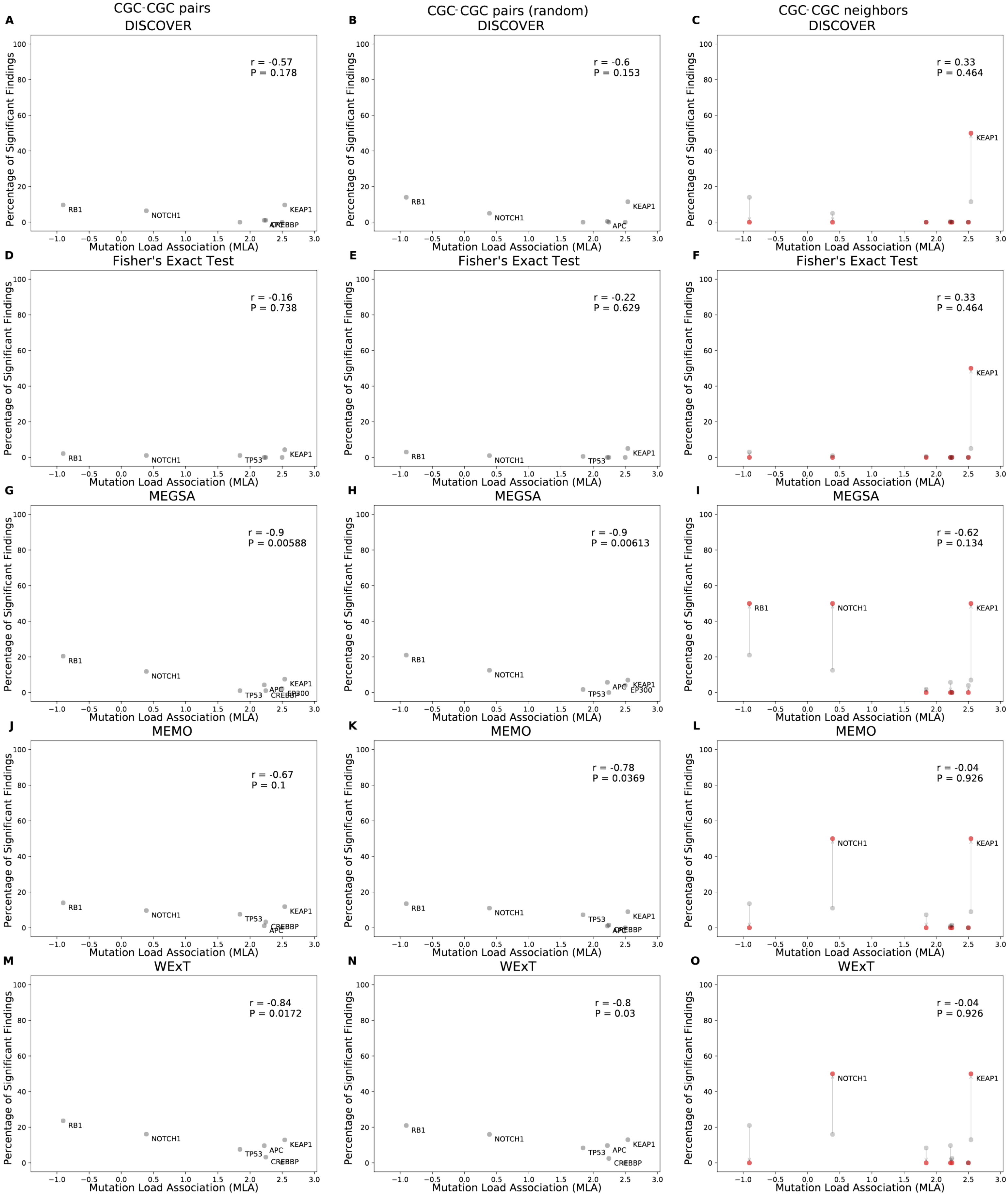

Fig 21: Comparison of ME results of all considered methods on TCGA LUSC cohort with $t = 20$ (485 samples) for CGC genes that have > 1 neighbors. The scatterplots show the percentage significance of ME runs (p-value<0.05) versus MLA values for CGC genes. (A) Results of DISCOVER where tests are performed between a CGC gene and all other CGC genes. B) Results of DISCOVER where tests are performed between a CGC gene and a random subset of all other CGC genes so that ME of a CGC gene of interest is checked with same sized group of genes in both B and C. (C) Results of DISCOVER where tests are performed between a CGC gene and its PPI neighbors that are in CGC (red) compared with (B) in gray. Analogous results are shown for Fisher's Exact Test (D, E, F), MEGSA (G, H, I), MEMO (J, K, L) and WExT (M, N, O) where coloring is the same as previously described for (C).

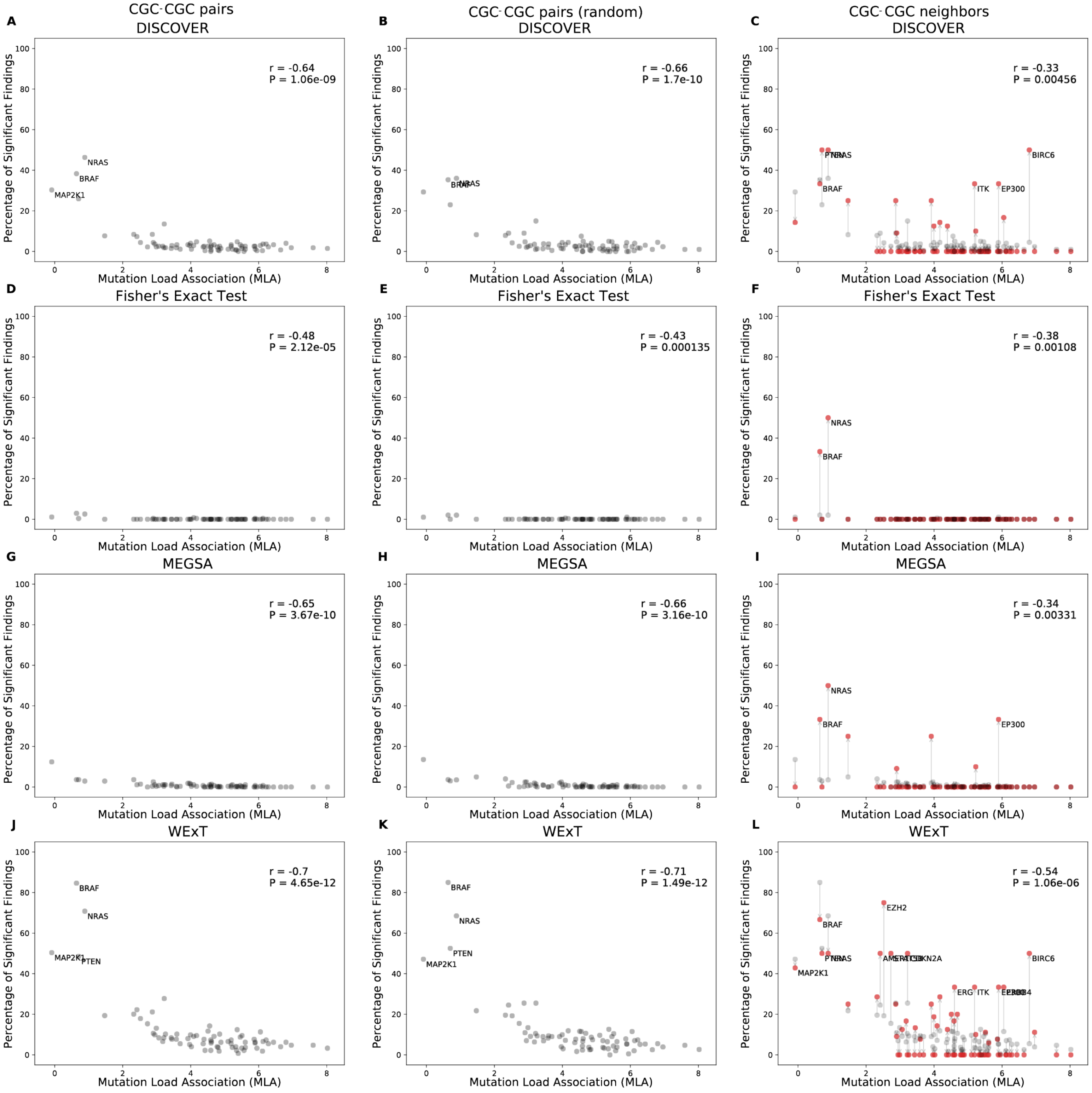

Fig 22: Comparison of ME results of all considered methods on TCGA SKCM cohort with $t = 20$ (468 samples) for CGC genes that have > 1 neighbors. The scatterplots show the percentage significance of ME runs (p-value<0.05) versus MLA values for CGC genes. (A) Results of DISCOVER where tests are performed between a CGC gene and all other CGC genes. B) Results of DISCOVER where tests are performed between a CGC gene and a random subset of all other CGC genes so that ME of a CGC gene of interest is checked with same sized group of genes in both B and C. (C) Results of DISCOVER where tests are performed between a CGC gene and its PPI neighbors that are in CGC (red) compared with (B) in gray. Analogous results are shown for Fisher's Exact Test (D, E, F), MEGSA (G, H, I), and WExT (J, K, L) where coloring is the same as previously described for (C).

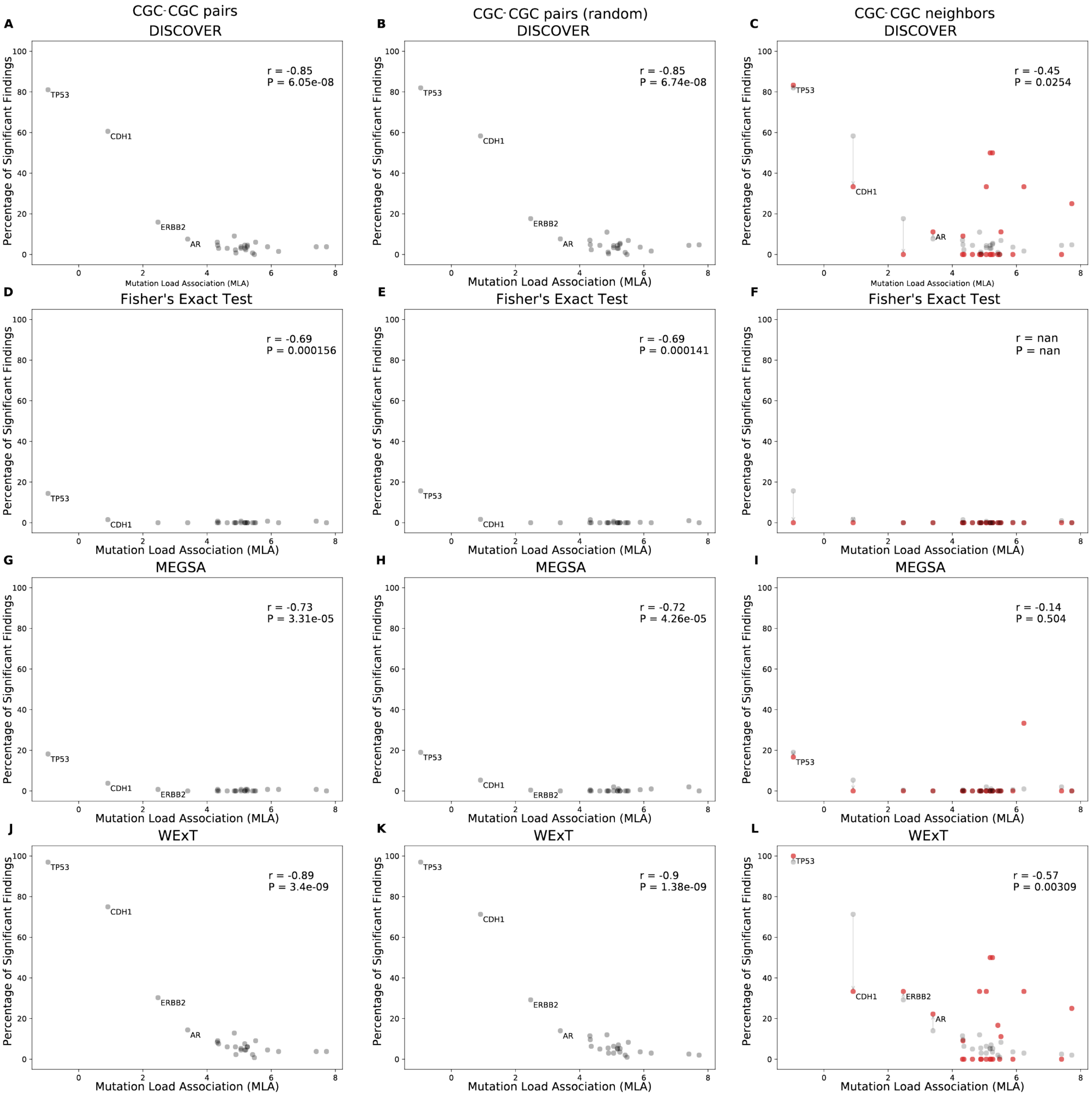

Fig 23: Comparison of ME results of all considered methods on TCGA STAD cohort with $t = 20$ (438 samples) for CGC genes that have > 1 neighbors. The scatterplots show the percentage significance of ME runs (p-value<0.05) versus MLA values for CGC genes. (A) Results of DISCOVER where tests are performed between a CGC gene and all other CGC genes. B) Results of DISCOVER where tests are performed between a CGC gene and a random subset of all other CGC genes so that ME of a CGC gene of interest is checked with same sized group of genes in both B and C. (C) Results of DISCOVER where tests are performed between a CGC gene and its PPI neighbors that are in CGC (red) compared with (B) in gray. Analogous results are shown for Fisher's Exact Test (D, E, F), MEGSA (G, H, I), and WExT (J, K, L) where coloring is the same as previously described for (C).

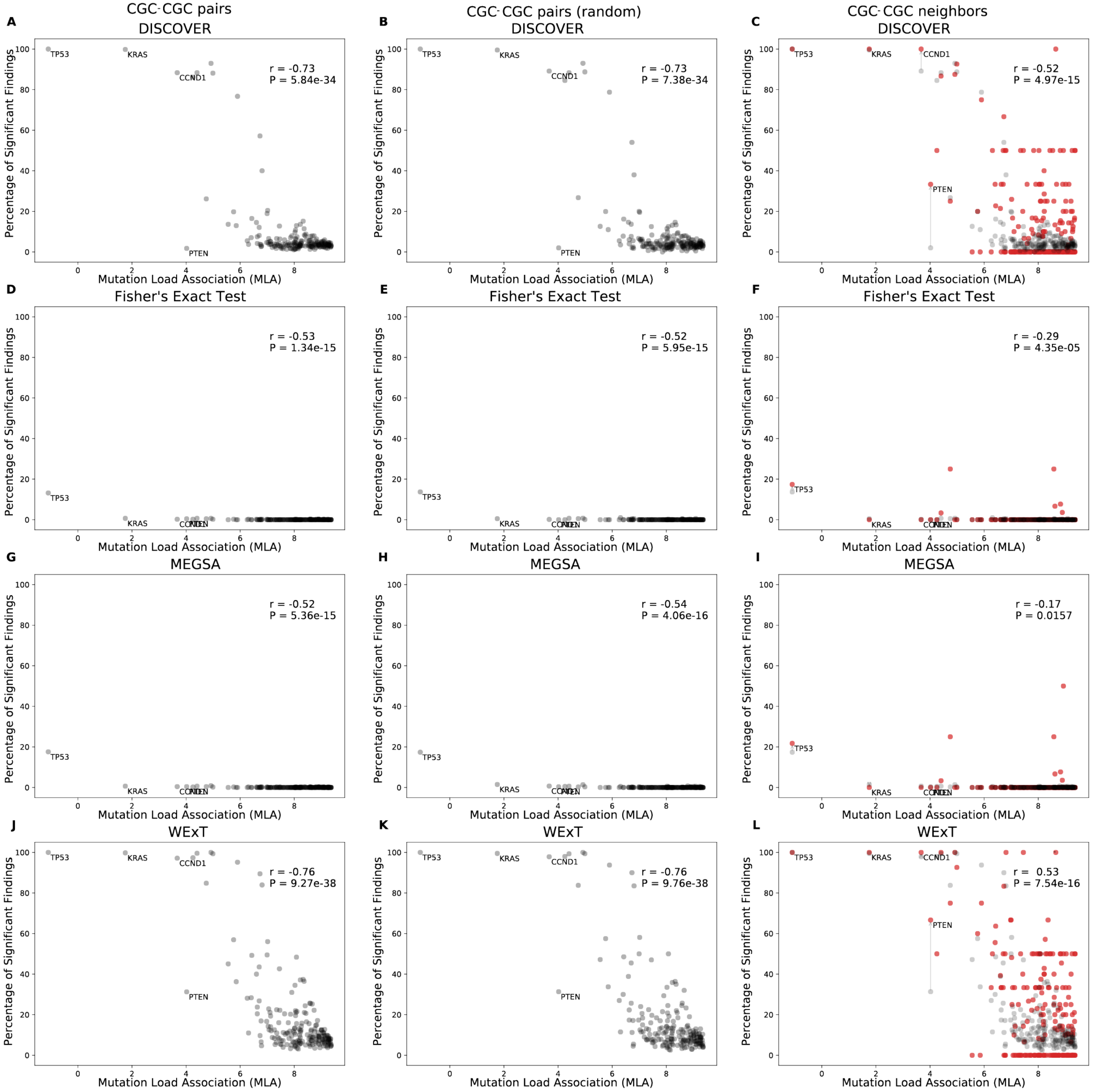

Fig 24: Comparison of ME results of all considered methods on TCGA UCEC cohort with $t = 20$ (531 samples) for CGC genes that have > 1 neighbors. The scatterplots show the percentage significance of ME runs (p-value<0.05) versus MLA values for CGC genes. (A) Results of DISCOVER where tests are performed between a CGC gene and all other CGC genes. B) Results of DISCOVER where tests are performed between a CGC gene and a random subset of all other CGC genes so that ME of a CGC gene of interest is checked with same sized group of genes in both B and C. (C) Results of DISCOVER where tests are performed between a CGC gene and its PPI neighbors that are in CGC (red) compared with (B) in gray. Analogous results are shown for Fisher's Exact Test (D, E, F), MEGSA (G, H, I), and WExT (J, K, L) where coloring is the same as previously described for (C).

Scatterplots of percentage significance of mutual exclusivity runs vs mutation load association (MLA) when only CGC genes that have > 1 neighbors are included ($t = 5$)

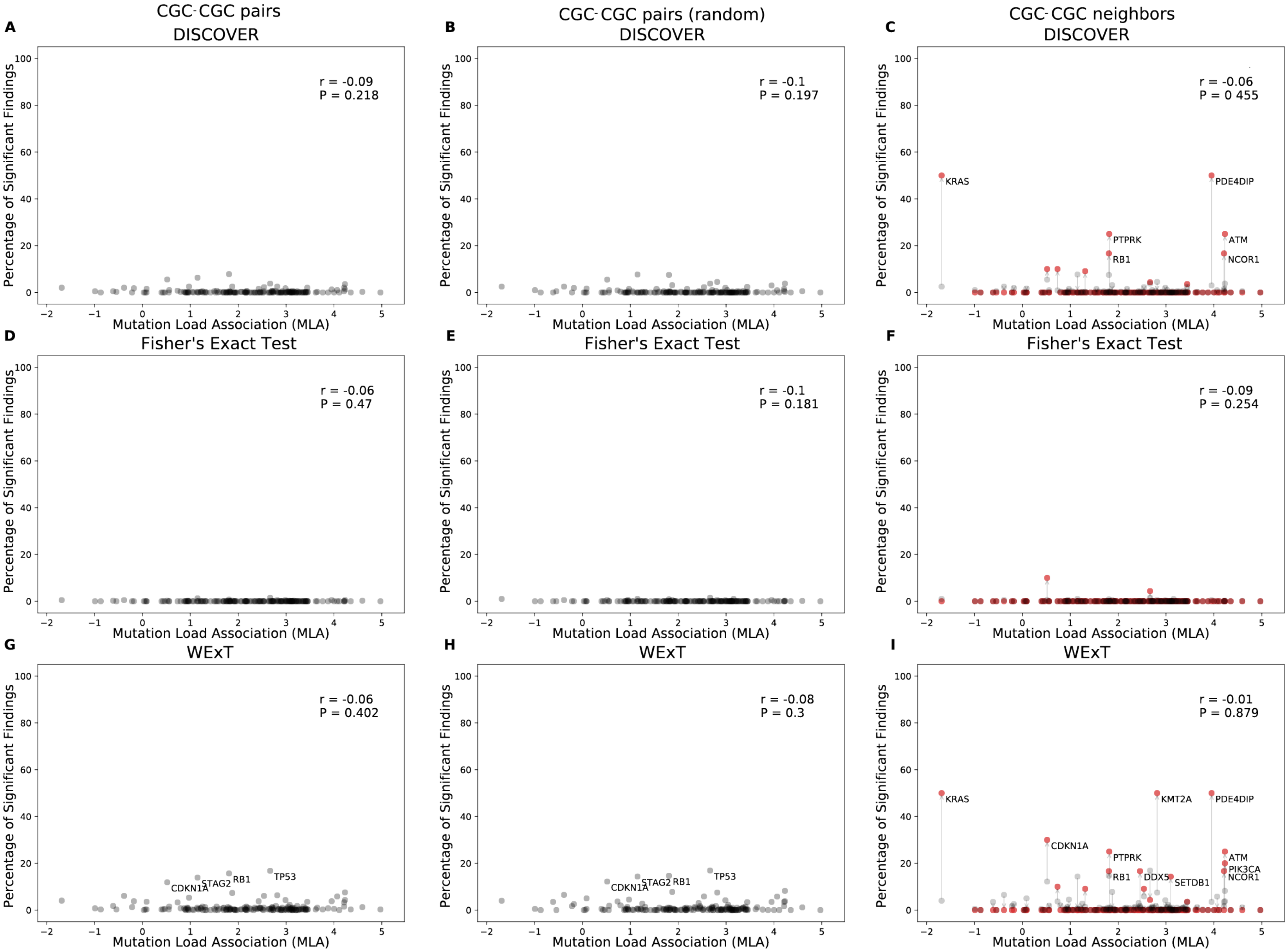

Fig 25: Comparison of ME results of all considered methods on TCGA BLCA cohort with $t = 5$ (411 samples) for CGC genes that have > 1 neighbors. The scatterplots show the percentage significance of ME runs (p-value<0.05) versus MLA values for CGC genes. (A) Results of DISCOVER where tests are performed between a CGC gene and all other CGC genes. B) Results of DISCOVER where tests are performed between a CGC gene and a random subset of all other CGC genes so that ME of a CGC gene of interest is checked with same sized group of genes in both B and C. (C) Results of DISCOVER where tests are performed between a CGC gene and its PPI neighbors that are in CGC (red) compared with (B) in gray. Analogous results are shown for Fisher's Exact Test (D, E, F), and WExT (G, H, I) where coloring is the same as previously described for (C).

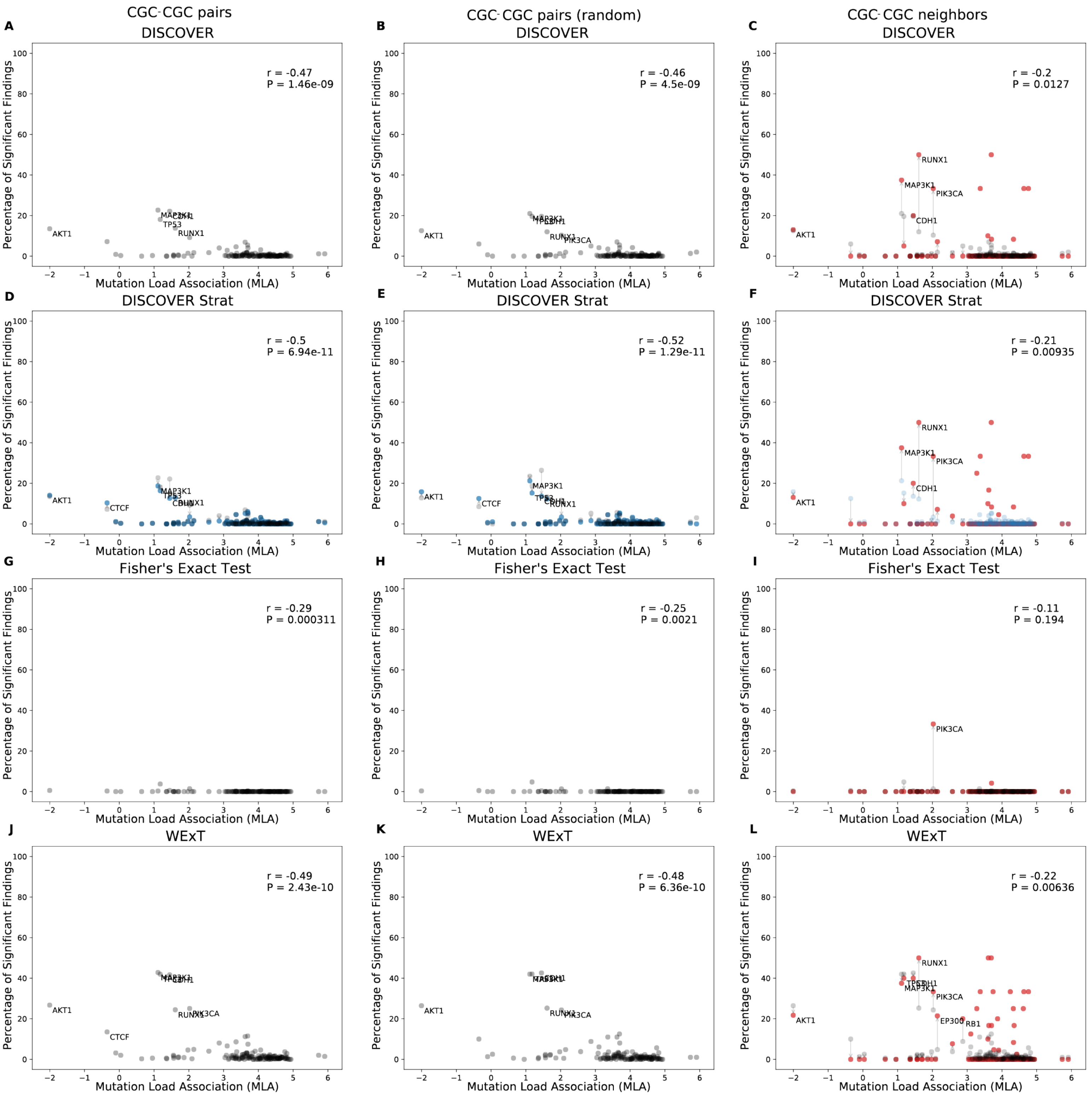

Fig 26: Comparison of ME results of all considered methods on TCGA BRCA cohort with $t = 5$ (1026 samples) for CGC genes that have > 1 neighbors. The scatterplots show the percentage significance of ME runs (p-value<0.05) versus MLA values for CGC genes. (A) Results of DISCOVER where tests are performed between a CGC gene and all other CGC genes. B) Results of DISCOVER where tests are performed between a CGC gene and a random subset of all other CGC genes so that ME of a CGC gene of interest is checked with same sized group of genes in both B and C. (C) Results of DISCOVER where tests are performed between a CGC gene and its PPI neighbors that are in CGC (red) compared with (B) in gray. (D) Results of DISCOVER Strat where where tests are performed between a CGC gene and all other CGC genes (blue) compared with (A) in gray. (E) Results of DISCOVER Strat where where tests are performed between a CGC gene and a random subset of all other CGC genes so that ME of a CGC gene of interest is checked with same sized group of genes in both E and F. Values are shown in blue and compared with (B) in gray. (F) Results of DISCOVER where tests are performed between a CGC gene and its PPI neighbors that are in CGC (red) compared with (E) in blue. Analogous results are shown for Fisher's Exact Test (G, H, I), and WExT (J, K, L) where coloring is the same as previously described for (C).

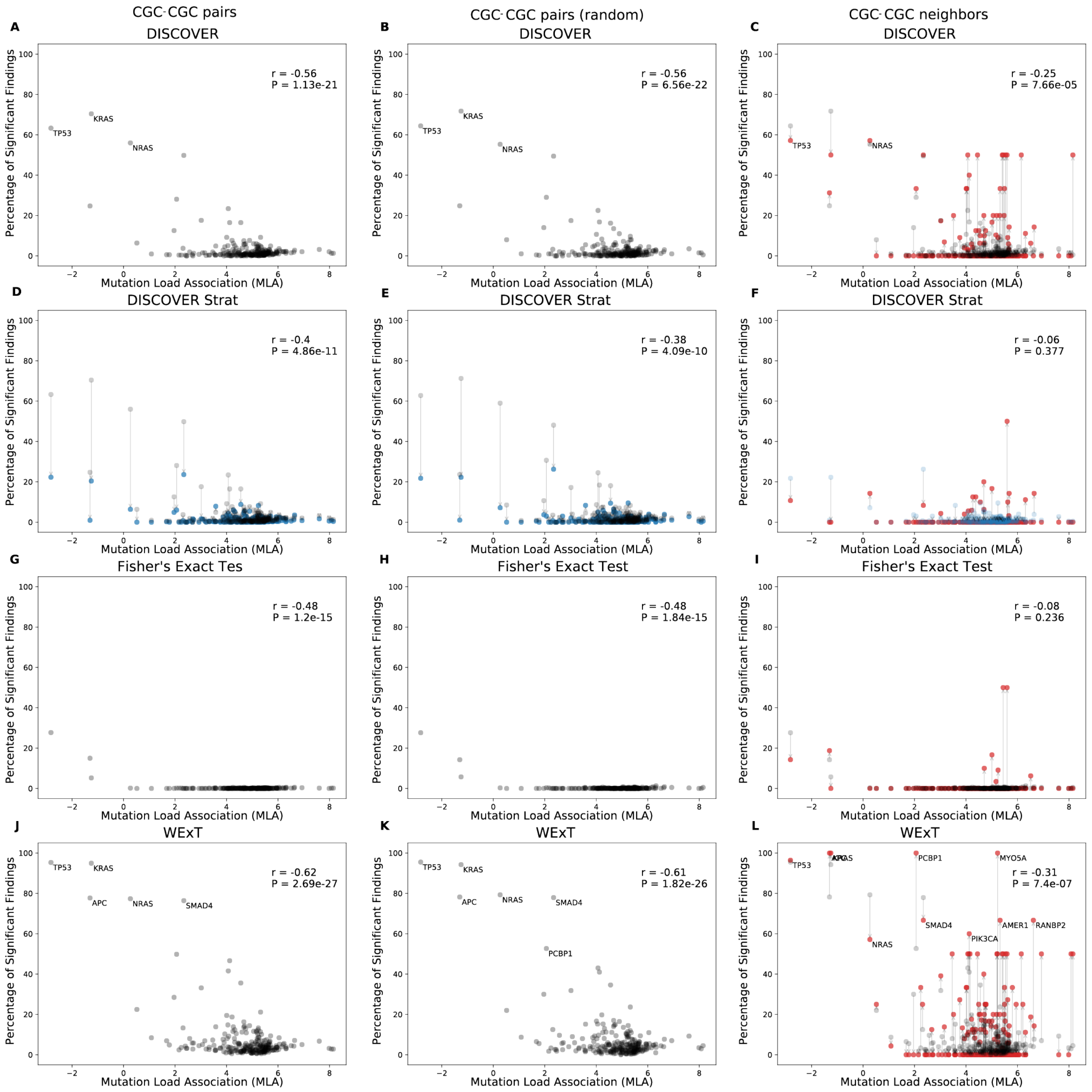

Fig 27: Comparison of ME results of all considered methods on TCGA COADREAD cohort with $t = 5$ (498 samples) for CGC genes that have > 1 neighbors. The scatterplots show the percentage significance of ME runs (p-value<0.05) versus MLA values for CGC genes. (A) Results of DISCOVER where tests are performed between a CGC gene and all other CGC genes. B) Results of DISCOVER where tests are performed between a CGC gene and a random subset of all other CGC genes so that ME of a CGC gene of interest is checked with same sized group of genes in both B and C. (C) Results of DISCOVER where tests are performed between a CGC gene and its PPI neighbors that are in CGC (red) compared with (B) in gray. (D) Results of DISCOVER Strat where where tests are performed between a CGC gene and all other CGC genes (blue) compared with (A) in gray. (E) Results of DISCOVER Strat where where tests are performed between a CGC gene and a random subset of all other CGC genes so that ME of a CGC gene of interest is checked with same sized group of genes in both E and F. Values are shown in blue and compared with (B) in gray. (F) Results of DISCOVER where tests are performed between a CGC gene and its PPI neighbors that are in CGC (red) compared with (E) in blue. Analogous results are shown for Fisher's Exact Test (G, H, I), and WExT (J, K, L) where coloring is the same as previously described for (C).

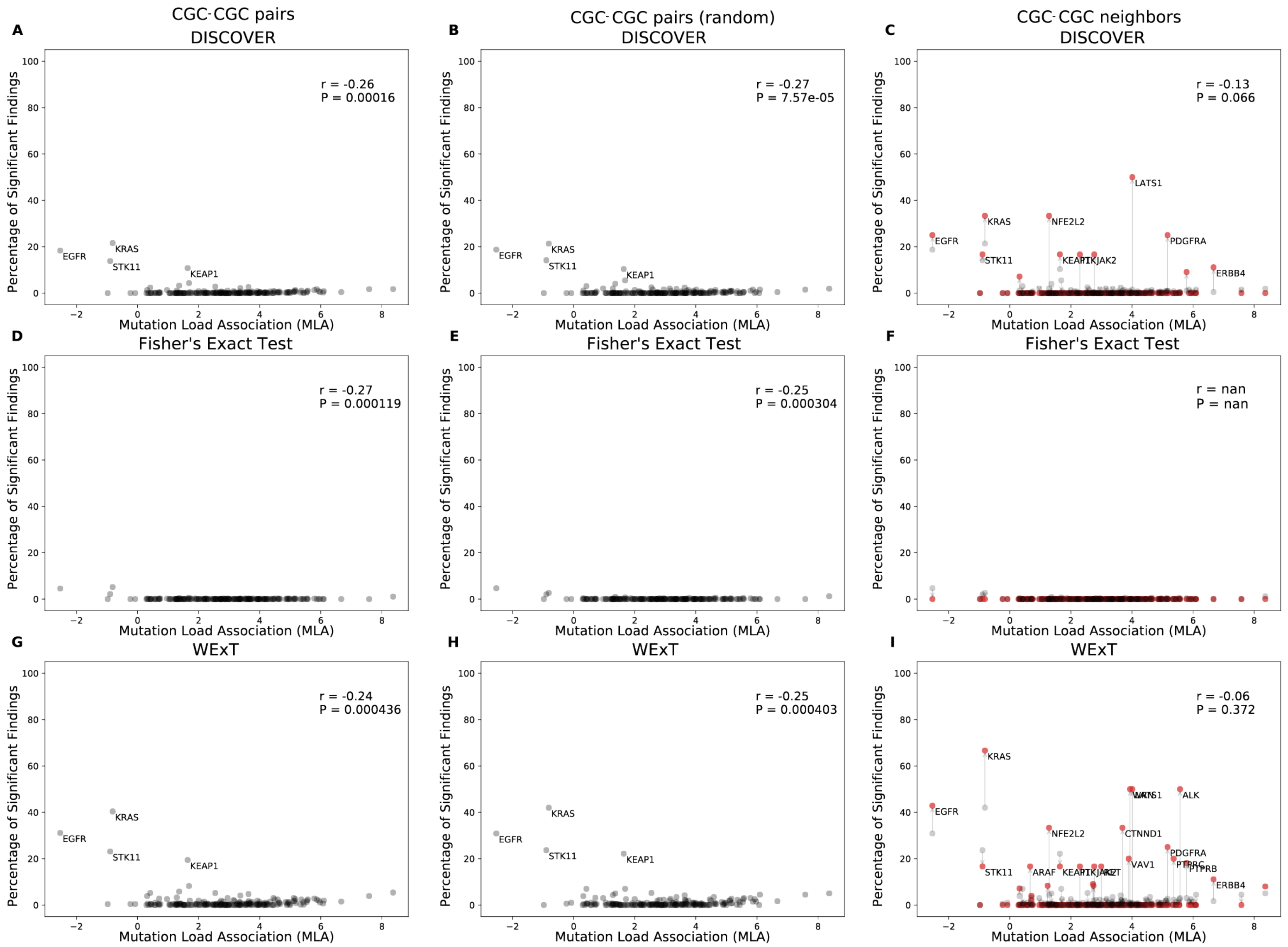

Fig 28: Comparison of ME results of all considered methods on TCGA LUAD cohort with $t = 5$ (568 samples) for CGC genes that have > 1 neighbors. The scatterplots show the percentage significance of ME runs (p-value<0.05) versus MLA values for CGC genes. (A) Results of DISCOVER where tests are performed between a CGC gene and all other CGC genes. B) Results of DISCOVER where tests are performed between a CGC gene and a random subset of all other CGC genes so that ME of a CGC gene of interest is checked with same sized group of genes in both B and C. (C) Results of DISCOVER where tests are performed between a CGC gene and its PPI neighbors that are in CGC (red) compared with (B) in gray. Analogous results are shown for Fisher's Exact Test (D, E, F), and WExT (G, H, I) where coloring is the same as previously described for (C).

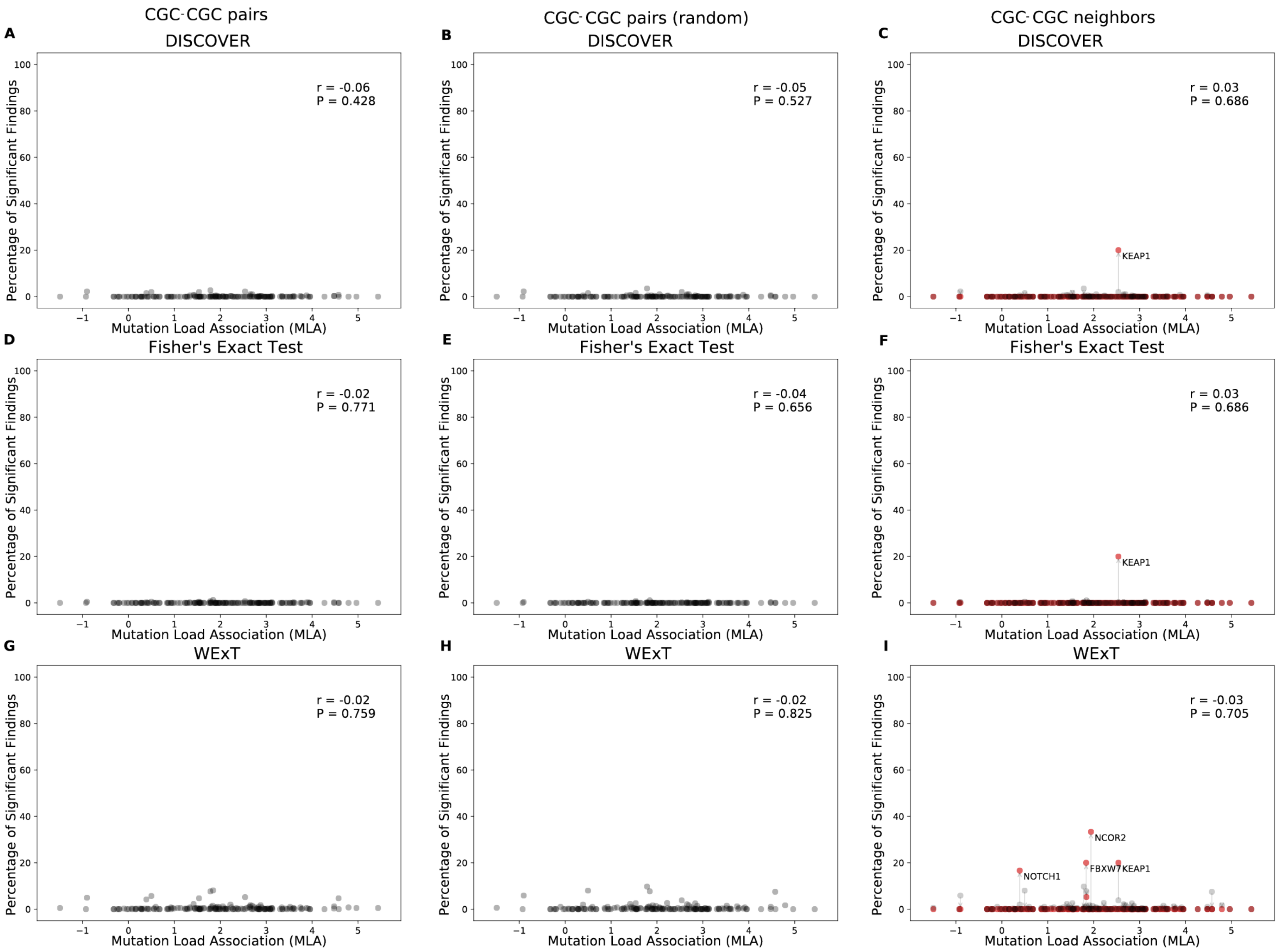

Fig 29: Comparison of ME results of all considered methods on TCGA LUSC cohort with $t = 5$ (485 samples) for CGC genes that have > 1 neighbors. The scatterplots show the percentage significance of ME runs (p-value<0.05) versus MLA values for CGC genes. (A) Results of DISCOVER where tests are performed between a CGC gene and all other CGC genes. B) Results of DISCOVER where tests are performed between a CGC gene and a random subset of all other CGC genes so that ME of a CGC gene of interest is checked with same sized group of genes in both B and C. (C) Results of DISCOVER where tests are performed between a CGC gene and its PPI neighbors that are in CGC (red) compared with (B) in gray. Analogous results are shown for Fisher's Exact Test (D, E, F), and WExT (G, H, I) where coloring is the same as previously described for (C).

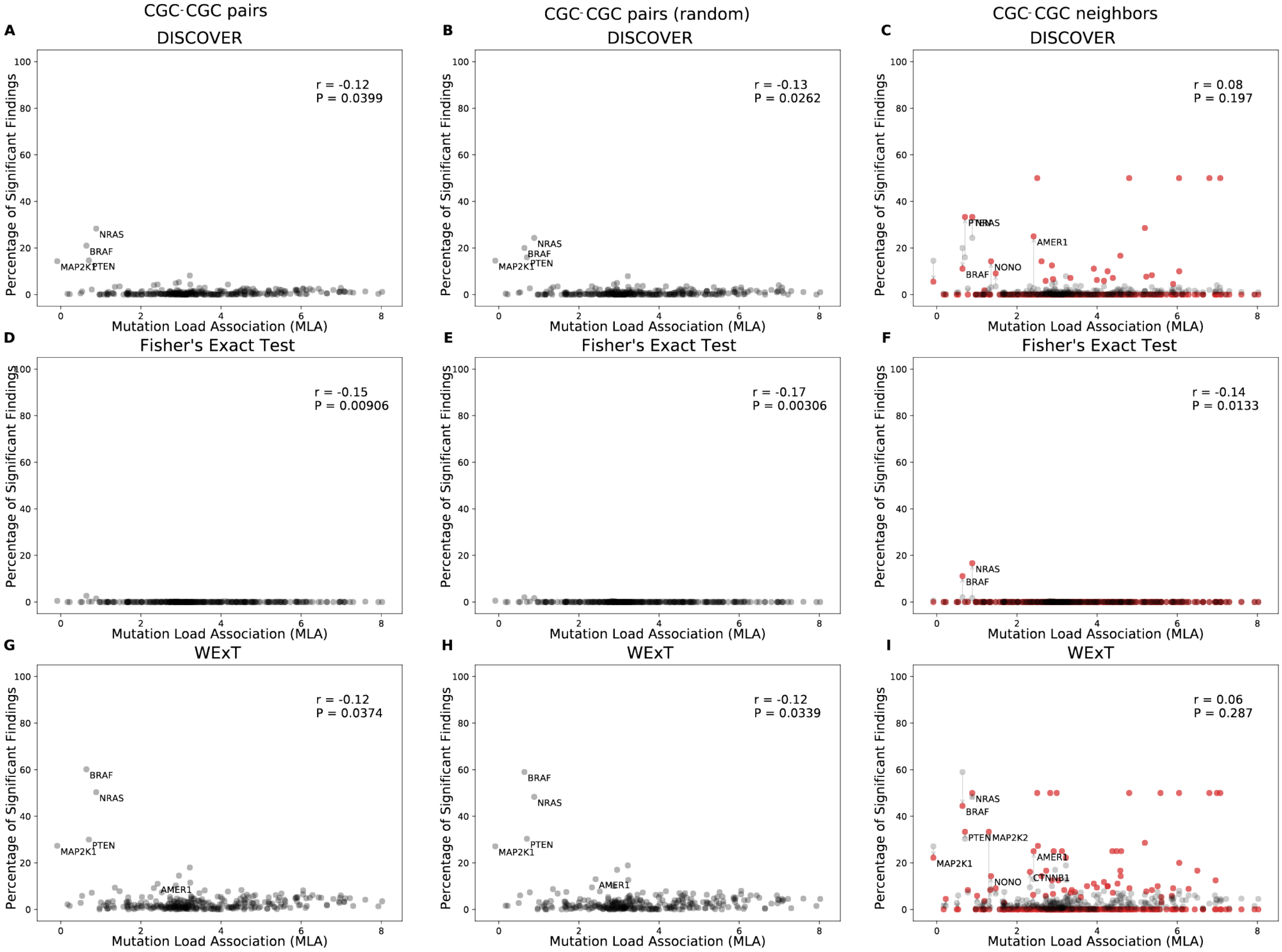

Fig 30: Comparison of ME results of all considered methods on TCGA SKCM cohort with $t = 5$ (468 samples) for CGC genes that have > 1 neighbors. The scatterplots show the percentage significance of ME runs (p-value<0.05) versus MLA values for CGC genes. (A) Results of DISCOVER where tests are performed between a CGC gene and all other CGC genes. B) Results of DISCOVER where tests are performed between a CGC gene and a random subset of all other CGC genes so that ME of a CGC gene of interest is checked with same sized group of genes in both B and C. (C) Results of DISCOVER where tests are performed between a CGC gene and its PPI neighbors that are in CGC (red) compared with (B) in gray. Analogous results are shown for Fisher's Exact Test (D, E, F), and WExT (G, H, I) where coloring is the same as previously described for (C).

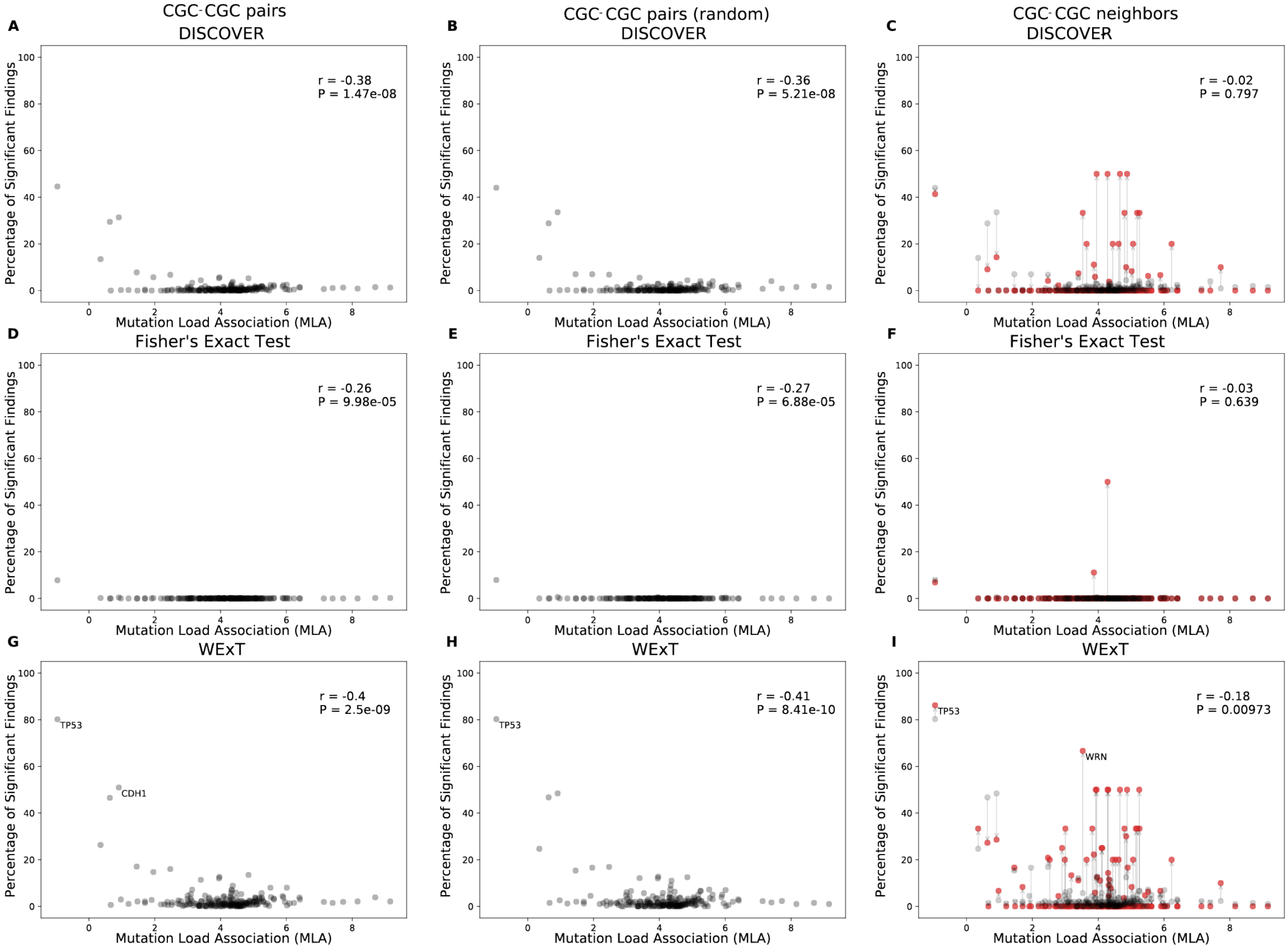

Fig 31: Comparison of ME results of all considered methods on TCGA STAD cohort with $t = 5$ (438 samples) for CGC genes that have > 1 neighbors. The scatterplots show the percentage significance of ME runs (p-value<0.05) versus MLA values for CGC genes. (A) Results of DISCOVER where tests are performed between a CGC gene and all other CGC genes. B) Results of DISCOVER where tests are performed between a CGC gene and a random subset of all other CGC genes so that ME of a CGC gene of interest is checked with same sized group of genes in both B and C. (C) Results of DISCOVER where tests are performed between a CGC gene and its PPI neighbors that are in CGC (red) compared with (B) in gray. Analogous results are shown for Fisher's Exact Test (D, E, F), and WExT (G, H, I) where coloring is the same as previously described for (C).

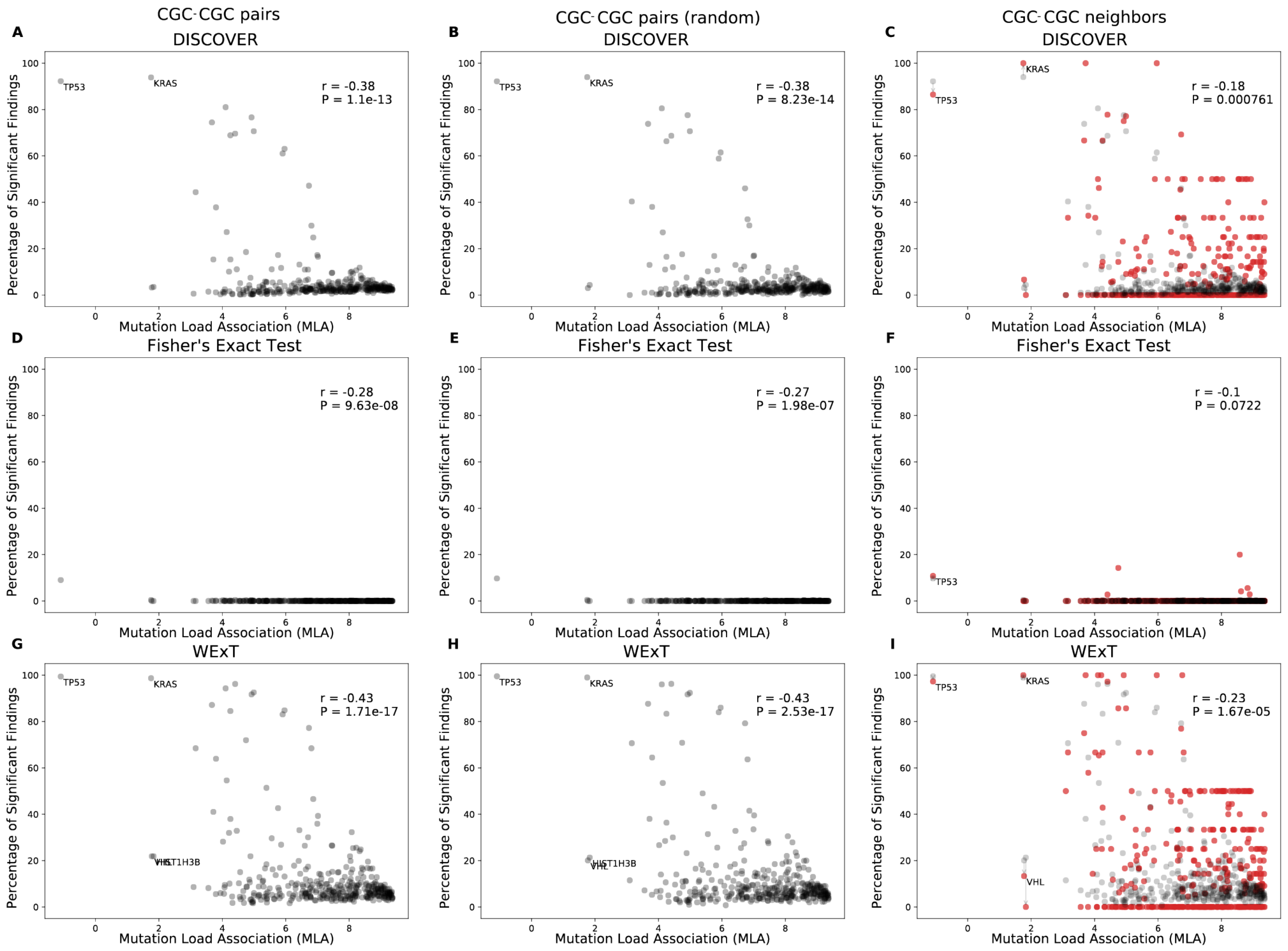

Fig 32: Comparison of ME results of all considered methods on TCGA UCEC cohort with $t = 5$ (531 samples) for CGC genes that have > 1 neighbors. The scatterplots show the percentage significance of ME runs (p-value<0.05) versus MLA values for CGC genes. (A) Results of DISCOVER where tests are performed between a CGC gene and all other CGC genes. B) Results of DISCOVER where tests are performed between a CGC gene and a random subset of all other CGC genes so that ME of a CGC gene of interest is checked with same sized group of genes in both B and C. (C) Results of DISCOVER where tests are performed between a CGC gene and its PPI neighbors that are in CGC (red) compared with (B) in gray. Analogous results are shown for Fisher's Exact Test (D, E, F), and WExT (G, H, I) where coloring is the same as previously described for (C).

Table 49: Number of available samples for each tissue within the RNA-seq datasets downloaded from GTEx

Cancer Type	Tissue	Number of Samples
BLCA	Bladder	21
BRCA	Breast Mammary Tissue	459
COADREAD	Colon Sigmoid	779
	Colon Transverse	
LUAD	Lung	578
LUSC	Lung	578
SKCM	Skin Not Sun Exposed (Suprapubic)	1809
	Skin Sun Exposed (Lower leg)	
	Cells Cultured fibroblasts	
STAD	Stomach	359
UCEC	Uterus	142

Results of network-centric ME evaluation framework run on the tissue-specific network (TSN) with control group X_1 and $t = 20$

Table 50: Metrics for BLCA data on the TSN network constructed with co-expression ratio threshold 0.0, X_1 as the control group and $t = 20$ (Bladder tissue | 21 samples | 56 CGC-CGC pairs)

Method	Precision	Sensitivity	F1 Score	$\text{Precision}_{\text{strict}}$	$\text{Sensitivity}_{\text{strict}}$	F1 $_{\text{strict}}$ Score
DISCOVER	0.800	0.077	0.140	0.800	0.077	0.140
Fisher's Exact Test	1.000	0.037	0.071	1.000	0.037	0.071
MEGSA	1.000	0.072	0.134	1.000	0.072	0.134
MEMO	0.800	0.076	0.139	0.800	0.076	0.139
WExT	0.571	0.078	0.137	0.571	0.078	0.137

Table 51: Metrics for BLCA data on the TSN network constructed with co-expression ratio threshold 0.5, X_1 as the control group and $t = 20$ (Bladder tissue | 21 samples | 52 CGC-CGC pairs)

Method	Precision	Sensitivity	F1 Score	$\text{Precision}_{\text{strict}}$	$\text{Sensitivity}_{\text{strict}}$	F1 $_{\text{strict}}$ Score
DISCOVER	0.800	0.082	0.148	0.800	0.082	0.149
Fisher's Exact Test	1.000	0.040	0.077	1.000	0.040	0.077
MEGSA	1.000	0.077	0.143	1.000	0.077	0.143
MEMO	0.667	0.085	0.151	0.667	0.085	0.151
WExT	0.667	0.086	0.152	0.667	0.086	0.152

Table 52: Metrics for BRCA data on the TSN network constructed with co-expression ratio threshold 0.0, X_1 as the control group and $t = 20$ (Breast tissue | 459 samples | 28 CGC-CGC pairs)

Method	Precision	Sensitivity	F1 Score	$\text{Precision}_{\text{strict}}$	$\text{Sensitivity}_{\text{strict}}$	F1 $_{\text{strict}}$ Score
DISCOVER	0.632	0.444	0.522	0.625	0.370	0.465
DISCOVER Strat	0.833	0.417	0.556	0.833	0.417	0.556
Fisher's Exact Test	1.000	0.074	0.138	1.000	0.074	0.138
MEGSA	1.000	0.071	0.133	1.000	0.071	0.133
MEMO	0.636	0.467	0.538	0.630	0.378	0.472
WExT	0.550	0.440	0.489	0.562	0.360	0.439

Table 53: Metrics for BRCA data on the TSN network constructed with co-expression ratio threshold 0.5, X_1 as the control group and $t = 20$ (Breast tissue | 459 samples | 28 CGC-CGC pairs)

Method	Precision	Sensitivity	F1 Score	$\text{Precision}_{\text{strict}}$	$\text{Sensitivity}_{\text{strict}}$	F1 $_{\text{strict}}$ Score
DISCOVER	0.632	0.444	0.522	0.625	0.370	0.465
DISCOVER Strat	0.750	0.409	0.529	0.750	0.409	0.529
Fisher's Exact Test	1.000	0.071	0.133	1.000	0.071	0.133
MEGSA	1.000	0.071	0.133	1.000	0.071	0.133
MEMO	0.677	0.447	0.538	0.704	0.404	0.513
WExT	0.556	0.417	0.476	0.600	0.375	0.462

Table 54: Metrics for COADREAD data on the TSN network constructed with co-expression ratio threshold 0.0, X_1 as the control group and $t = 20$ (Colon tissue | 779 samples | 194 CGC-CGC pairs)

Method	Precision	Sensitivity	F1 Score	$\text{Precision}_{\text{strict}}$	$\text{Sensitivity}_{\text{strict}}$	F1 $_{\text{strict}}$ Score
DISCOVER	0.672	0.226	0.339	0.720	0.189	0.299
DISCOVER Strat	0.667	0.042	0.079	0.636	0.037	0.070
Fisher's Exact Test	0.583	0.037	0.069	0.583	0.037	0.070
MEGSA	0.632	0.062	0.113	0.611	0.057	0.104
MEMO	0.653	0.342	0.449	0.639	0.246	0.355
WExT	0.681	0.414	0.515	0.736	0.340	0.465

Table 55: Metrics for COADREAD data on the TSN network constructed with co-expression ratio threshold 0.5, X_1 as the control group and $t = 20$ (Colon tissue | 779 samples | 194 CGC-CGC pairs)

Method	Precision	Sensitivity	F1 Score	$\text{Precision}_{\text{strict}}$	$\text{Sensitivity}_{\text{strict}}$	F1 $_{\text{strict}}$ Score
DISCOVER	0.662	0.234	0.346	0.699	0.191	0.300
DISCOVER Strat	0.727	0.042	0.079	0.727	0.042	0.079
Fisher's Exact Test	0.565	0.034	0.064	0.565	0.034	0.064
MEGSA	0.611	0.057	0.104	0.588	0.052	0.096
MEMO	0.663	0.340	0.449	0.667	0.248	0.362
WExT	0.692	0.410	0.515	0.743	0.336	0.463

Table 56: Metrics for LUAD data on the TSN network constructed with co-expression ratio threshold 0.0, X_1 as the control group and $t = 20$ (Lung tissue | 578 samples | 88 CGC-CGC pairs)

Method	Precision	Sensitivity	F1 Score	$\text{Precision}_{\text{strict}}$	$\text{Sensitivity}_{\text{strict}}$	F1 $_{\text{strict}}$ Score
DISCOVER	0.750	0.106	0.186	0.700	0.082	0.147
Fisher's Exact Test	0.000	0.000	NaN	0.000	0.000	NaN
MEGSA	0.667	0.023	0.044	0.667	0.023	0.044
MEMO	0.722	0.160	0.263	0.733	0.136	0.229
WExT	0.667	0.187	0.292	0.700	0.164	0.266

Table 57: Metrics for LUAD data on the TSN network constructed with co-expression ratio threshold 0.5, X_1 as the control group and $t = 20$ (Lung tissue | 578 samples | 82 CGC-CGC pairs)

Method	Precision	Sensitivity	F1 Score	$\text{Precision}_{\text{strict}}$	$\text{Sensitivity}_{\text{strict}}$	F1 $_{\text{strict}}$ Score
DISCOVER	0.778	0.093	0.167	0.750	0.080	0.145
Fisher's Exact Test	0.000	0.000	NaN	0.000	0.000	NaN
MEGSA	0.667	0.025	0.048	0.667	0.025	0.048
MEMO	0.692	0.118	0.201	0.727	0.105	0.183
WExT	0.632	0.152	0.245	0.688	0.139	0.231

Table 58: Metrics for LUSC data on the TSN network constructed with co-expression ratio threshold 0.0, X_1 as the control group and $t = 20$ (Lung tissue | 578 samples | 38 CGC-CGC pairs)

Method	Precision	Sensitivity	F1 Score	$\text{Precision}_{\text{strict}}$	$\text{Sensitivity}_{\text{strict}}$	F1 $_{\text{strict}}$ Score
DISCOVER	1.0	0.053	0.100	1.0	0.053	0.101
Fisher's Exact Test	1.0	0.053	0.100	1.0	0.053	0.101
MEGSA	1.0	0.158	0.273	1.0	0.158	0.273
MEMO	1.0	0.113	0.203	1.0	0.113	0.203
WExT	1.0	0.110	0.198	1.0	0.110	0.198

Table 59: Metrics for LUSC data on the TSN network constructed with co-expression ratio threshold 0.5, X_1 as the control group and $t = 20$ (Lung tissue | 578 samples | 36 CGC-CGC pairs)

Method	Precision	Sensitivity	F1 Score	$\text{Precision}_{\text{strict}}$	$\text{Sensitivity}_{\text{strict}}$	F1 $_{\text{strict}}$ Score
DISCOVER	1.0	0.056	0.107	1.0	0.056	0.106
Fisher's Exact Test	1.0	0.056	0.105	1.0	0.056	0.106
MEGSA	1.0	0.167	0.286	1.0	0.167	0.286
MEMO	1.0	0.119	0.213	1.0	0.119	0.213
WExT	1.0	0.119	0.213	1.0	0.119	0.213

Table 60: Metrics for SKCM data on the TSN network constructed with co-expression ratio threshold 0.0, X_1 as the control group and $t = 20$ (Skin tissue | 1809 samples | 456 CGC-CGC pairs)

Method	Precision	Sensitivity	F1 Score	$\text{Precision}_{\text{strict}}$	$\text{Sensitivity}_{\text{strict}}$	F1 $_{\text{strict}}$ Score
DISCOVER	0.833	0.045	0.086	0.833	0.045	0.085
Fisher's Exact Test	1.000	0.004	0.009	1.000	0.004	0.008
MEGSA	0.889	0.018	0.034	0.889	0.018	0.035
WExT	0.687	0.118	0.202	0.699	0.115	0.198

Table 61: Metrics for SKCM data on the TSN network constructed with co-expression ratio threshold 0.5, X_1 as the control group and $t = 20$ (Skin tissue | 1809 samples | 390 CGC-CGC pairs)

Method	Precision	Sensitivity	F1 Score	$\text{Precision}_{\text{strict}}$	$\text{Sensitivity}_{\text{strict}}$	F1 $_{\text{strict}}$ Score
DISCOVER	0.842	0.042	0.08	0.842	0.042	0.080
Fisher's Exact Test	1.000	0.005	0.01	1.000	0.005	0.010
MEGSA	0.889	0.021	0.04	0.889	0.021	0.041
WExT	0.667	0.117	0.20	0.677	0.112	0.192

Table 62: Metrics for STAD data on the TSN network constructed with co-expression ratio threshold 0.0, X_1 as the control group and $t = 20$ (Stomach tissue | 359 samples | 140 CGC-CGC pairs)

Method	Precision	Sensitivity	F1 Score	$\text{Precision}_{\text{strict}}$	$\text{Sensitivity}_{\text{strict}}$	F1 $_{\text{strict}}$ Score
DISCOVER	0.653	0.119	0.201	0.667	0.096	0.168
Fisher's Exact Test	0.000	0.000	NaN	0.000	0.000	NaN
MEGSA	0.667	0.014	0.028	0.667	0.014	0.027
WExT	0.634	0.193	0.295	0.636	0.156	0.251

Table 63: Metrics for STAD data on the TSN network constructed with co-expression ratio threshold 0.5, X_1 as the control group and $t = 20$ (Stomach tissue | 359 samples | 126 CGC-CGC pairs)

Method	Precision	Sensitivity	F1 Score	$\text{Precision}_{\text{strict}}$	$\text{Sensitivity}_{\text{strict}}$	F1 $_{\text{strict}}$ Score
DISCOVER	0.667	0.130	0.217	0.684	0.105	0.182
Fisher's Exact Test	0.000	0.000	NaN	0.000	0.000	NaN
MEGSA	0.667	0.016	0.031	0.667	0.016	0.031
WExT	0.649	0.197	0.302	0.667	0.164	0.263

Table 64: Metrics for UCEC data on the TSN network constructed with co-expression ratio threshold 0.0, X_1 as the control group and $t = 20$ (Uterus tissue | 142 samples | 1322 CGC-CGC pairs)

Method	Precision	Sensitivity	F1 Score	$\text{Precision}_{\text{strict}}$	$\text{Sensitivity}_{\text{strict}}$	F1 $_{\text{strict}}$ Score
DISCOVER	0.653	0.180	0.282	0.719	0.146	0.243
Fisher's Exact Test	0.769	0.008	0.015	0.769	0.008	0.016
MEGSA	0.786	0.008	0.016	0.786	0.008	0.016
WExT	0.619	0.282	0.388	0.667	0.230	0.342

Table 65: Metrics for UCEC data on the TSN network constructed with co-expression ratio threshold 0.5, X_1 as the control group and $t = 20$ (Uterus tissue | 142 samples | 1224 CGC-CGC pairs)

Method	Precision	Sensitivity	F1 Score	$\text{Precision}_{\text{strict}}$	$\text{Sensitivity}_{\text{strict}}$	F1 $_{\text{strict}}$ Score
DISCOVER	0.658	0.186	0.290	0.717	0.150	0.248
Fisher's Exact Test	0.833	0.008	0.016	0.833	0.008	0.016
MEGSA	0.786	0.009	0.018	0.786	0.009	0.018
WExT	0.614	0.286	0.390	0.665	0.233	0.345

Results of network-centric ME evaluation framework run on the tissue-specific network (TSN) with control group X_2 and $t = 20$

Table 66: Metrics for BLCA data on the TSN network constructed with co-expression ratio threshold 0.0, X_2 as the control group and $t = 20$ (Bladder tissue | 21 samples | 24 CGC-CGC pairs)

Method	Precision	Sensitivity	F1 Score	$\text{Precision}_{\text{strict}}$	$\text{Sensitivity}_{\text{strict}}$	F1 $_{\text{strict}}$ Score
DISCOVER	0.537	0.276	0.365	0.579	0.210	0.308
DISCOVER Strat	0.455	0.048	0.086	0.400	0.038	0.069
Fisher's Exact Test	0.444	0.038	0.069	0.375	0.028	0.052
MEGSA	0.571	0.075	0.133	0.538	0.066	0.118
MEMO	0.566	0.388	0.460	0.495	0.215	0.300
WExT	0.575	0.438	0.497	0.596	0.295	0.395

Table 67: Metrics for BLCA data on the TSN network constructed with co-expression ratio threshold 0.5, X_2 as the control group and $t = 20$ (Bladder tissue | 21 samples | 21 CGC-CGC pairs)

Method	Precision	Sensitivity	F1 Score	$\text{Precision}_{\text{strict}}$	$\text{Sensitivity}_{\text{strict}}$	F1 $_{\text{strict}}$ Score
DISCOVER	0.537	0.276	0.365	0.579	0.210	0.308
DISCOVER Strat	0.455	0.048	0.086	0.400	0.038	0.069
Fisher's Exact Test	0.444	0.038	0.069	0.375	0.028	0.052
MEGSA	0.571	0.075	0.133	0.538	0.066	0.118
MEMO	0.566	0.388	0.460	0.495	0.215	0.300
WExT	0.575	0.438	0.497	0.596	0.295	0.395

Table 68: Metrics for BRCA data on the TSN network constructed with co-expression ratio threshold 0.0, X_2 as the control group and $t = 20$ (Breast tissue | 459 samples | 9 CGC-CGC pairs)

Method	Precision	Sensitivity	F1 Score	$\text{Precision}_{\text{strict}}$	$\text{Sensitivity}_{\text{strict}}$	F1 $_{\text{strict}}$ Score
DISCOVER	0.537	0.276	0.365	0.579	0.210	0.308
DISCOVER Strat	0.455	0.048	0.086	0.400	0.038	0.069
Fisher's Exact Test	0.444	0.038	0.069	0.375	0.028	0.052
MEGSA	0.571	0.075	0.133	0.538	0.066	0.118
MEMO	0.566	0.388	0.460	0.495	0.215	0.300
WExT	0.575	0.438	0.497	0.596	0.295	0.395

Table 69: Metrics for BRCA data on the TSN network constructed with co-expression ratio threshold 0.5, X_2 as the control group and $t = 20$ (Breast tissue | 459 samples | 5 CGC-CGC pairs)

Method	Precision	Sensitivity	F1 Score	$\text{Precision}_{\text{strict}}$	$\text{Sensitivity}_{\text{strict}}$	F1 $_{\text{strict}}$ Score
DISCOVER	0.537	0.276	0.365	0.579	0.210	0.308
DISCOVER Strat	0.455	0.048	0.086	0.400	0.038	0.069
Fisher's Exact Test	0.444	0.038	0.069	0.375	0.028	0.052
MEGSA	0.571	0.075	0.133	0.538	0.066	0.118
MEMO	0.566	0.388	0.460	0.495	0.215	0.300
WExT	0.575	0.438	0.497	0.596	0.295	0.395

Table 70: Metrics for COADREAD data on the TSN network constructed with co-expression ratio threshold 0.0, X_2 as the control group and $t = 20$ (Colon tissue | 779 samples | 105 CGC-CGC pairs)

Method	Precision	Sensitivity	F1 Score	$\text{Precision}_{\text{strict}}$	$\text{Sensitivity}_{\text{strict}}$	F1 $_{\text{strict}}$ Score
DISCOVER	0.537	0.276	0.365	0.579	0.210	0.308
DISCOVER Strat	0.455	0.048	0.086	0.400	0.038	0.069
Fisher's Exact Test	0.444	0.038	0.069	0.375	0.028	0.052
MEGSA	0.571	0.075	0.133	0.538	0.066	0.118
MEMO	0.566	0.388	0.460	0.495	0.215	0.300
WExT	0.575	0.438	0.497	0.596	0.295	0.395

Table 71: Metrics for COADREAD data on the TSN network constructed with co-expression ratio threshold 0.5, X_2 as the control group and $t = 20$ (Colon tissue | 779 samples | 105 CGC-CGC pairs)

Method	Precision	Sensitivity	F1 Score	$\text{Precision}_{\text{strict}}$	$\text{Sensitivity}_{\text{strict}}$	F1 $_{\text{strict}}$ Score
DISCOVER	0.537	0.276	0.365	0.579	0.210	0.308
DISCOVER Strat	0.455	0.048	0.086	0.400	0.038	0.069
Fisher's Exact Test	0.444	0.038	0.069	0.375	0.028	0.052
MEGSA	0.571	0.075	0.133	0.538	0.066	0.118
MEMO	0.566	0.388	0.460	0.495	0.215	0.300
WExT	0.575	0.438	0.497	0.596	0.295	0.395

Table 72: Metrics for LUAD data on the TSN network constructed with co-expression ratio threshold 0.0, X_2 as the control group and $t = 20$ (Lung tissue | 578 samples | 53 CGC-CGC pairs)

Method	Precision	Sensitivity	F1 Score	$\text{Precision}_{\text{strict}}$	$\text{Sensitivity}_{\text{strict}}$	F1 $_{\text{strict}}$ Score
DISCOVER	0.537	0.276	0.365	0.579	0.210	0.308
DISCOVER Strat	0.455	0.048	0.086	0.400	0.038	0.069
Fisher's Exact Test	0.444	0.038	0.069	0.375	0.028	0.052
MEGSA	0.571	0.075	0.133	0.538	0.066	0.118
MEMO	0.566	0.388	0.460	0.495	0.215	0.300
WExT	0.575	0.438	0.497	0.596	0.295	0.395

Table 73: Metrics for LUAD data on the TSN network constructed with co-expression ratio threshold 0.5, X_2 as the control group and $t = 20$ (Lung tissue | 578 samples | 34 CGC-CGC pairs)

Method	Precision	Sensitivity	F1 Score	$\text{Precision}_{\text{strict}}$	$\text{Sensitivity}_{\text{strict}}$	F1 $_{\text{strict}}$ Score
DISCOVER	0.537	0.276	0.365	0.579	0.210	0.308
DISCOVER Strat	0.455	0.048	0.086	0.400	0.038	0.069
Fisher's Exact Test	0.444	0.038	0.069	0.375	0.028	0.052
MEGSA	0.571	0.075	0.133	0.538	0.066	0.118
MEMO	0.566	0.388	0.460	0.495	0.215	0.300
WExT	0.575	0.438	0.497	0.596	0.295	0.395

Table 74: **Metrics for LUSC data on the TSN network constructed with co-expression ratio threshold 0.0, X_2 as the control group and $t = 20$ (Lung tissue | 578 samples | 21 CGC-CGC pairs)**

Method	Precision	Sensitivity	F1 Score	$\text{Precision}_{\text{strict}}$	$\text{Sensitivity}_{\text{strict}}$	F1 $_{\text{strict}}$ Score
DISCOVER	0.537	0.276	0.365	0.579	0.210	0.308
DISCOVER Strat	0.455	0.048	0.086	0.400	0.038	0.069
Fisher's Exact Test	0.444	0.038	0.069	0.375	0.028	0.052
MEGSA	0.571	0.075	0.133	0.538	0.066	0.118
MEMO	0.566	0.388	0.460	0.495	0.215	0.300
WExT	0.575	0.438	0.497	0.596	0.295	0.395

Table 75: **Metrics for LUSC data on the TSN network constructed with co-expression ratio threshold 0.5, X_2 as the control group and $t = 20$ (Lung tissue | 578 samples | 20 CGC-CGC pairs)**

Method	Precision	Sensitivity	F1 Score	$\text{Precision}_{\text{strict}}$	$\text{Sensitivity}_{\text{strict}}$	F1 $_{\text{strict}}$ Score
DISCOVER	0.537	0.276	0.365	0.579	0.210	0.308
DISCOVER Strat	0.455	0.048	0.086	0.400	0.038	0.069
Fisher's Exact Test	0.444	0.038	0.069	0.375	0.028	0.052
MEGSA	0.571	0.075	0.133	0.538	0.066	0.118
MEMO	0.566	0.388	0.460	0.495	0.215	0.300
WExT	0.575	0.438	0.497	0.596	0.295	0.395

Table 76: **Metrics for SKCM data on the TSN network constructed with co-expression ratio threshold 0.0, X_2 as the control group and $t = 20$ (Skin tissue | 1809 samples | 312 CGC-CGC pairs)**

Method	Precision	Sensitivity	F1 Score	$\text{Precision}_{\text{strict}}$	$\text{Sensitivity}_{\text{strict}}$	F1 $_{\text{strict}}$ Score
DISCOVER	0.537	0.276	0.365	0.579	0.210	0.308
DISCOVER Strat	0.455	0.048	0.086	0.400	0.038	0.069
Fisher's Exact Test	0.444	0.038	0.069	0.375	0.028	0.052
MEGSA	0.571	0.075	0.133	0.538	0.066	0.118
MEMO	0.566	0.388	0.460	0.495	0.215	0.300
WExT	0.575	0.438	0.497	0.596	0.295	0.395

Table 77: **Metrics for SKCM data on the TSN network constructed with co-expression ratio threshold 0.5, X_2 as the control group and $t = 20$ (Skin tissue | 1809 samples | 263 CGC-CGC pairs)**

Method	Precision	Sensitivity	F1 Score	$\text{Precision}_{\text{strict}}$	$\text{Sensitivity}_{\text{strict}}$	F1 $_{\text{strict}}$ Score
DISCOVER	0.537	0.276	0.365	0.579	0.210	0.308
DISCOVER Strat	0.455	0.048	0.086	0.400	0.038	0.069
Fisher's Exact Test	0.444	0.038	0.069	0.375	0.028	0.052
MEGSA	0.571	0.075	0.133	0.538	0.066	0.118
MEMO	0.566	0.388	0.460	0.495	0.215	0.300
WExT	0.575	0.438	0.497	0.596	0.295	0.395

Table 78: **Metrics for STAD data on the TSN network constructed with co-expression ratio threshold 0.0, X_2 as the control group and $t = 20$ (Stomach tissue | 359 samples | 70 CGC-CGC pairs)**

Method	Precision	Sensitivity	F1 Score	$\text{Precision}_{\text{strict}}$	$\text{Sensitivity}_{\text{strict}}$	F1 $_{\text{strict}}$ Score
DISCOVER	0.537	0.276	0.365	0.579	0.210	0.308
DISCOVER Strat	0.455	0.048	0.086	0.400	0.038	0.069
Fisher's Exact Test	0.444	0.038	0.069	0.375	0.028	0.052
MEGSA	0.571	0.075	0.133	0.538	0.066	0.118
MEMO	0.566	0.388	0.460	0.495	0.215	0.300
WExT	0.575	0.438	0.497	0.596	0.295	0.395

Table 79: Metrics for STAD data on the TSN network constructed with co-expression ratio threshold 0.5, X_2 as the control group and $t = 20$ (Stomach tissue | 359 samples | 53 CGC-CGC pairs)

Method	Precision	Sensitivity	F1 Score	$\text{Precision}_{\text{strict}}$	$\text{Sensitivity}_{\text{strict}}$	F1 $_{\text{strict}}$ Score
DISCOVER	0.537	0.276	0.365	0.579	0.210	0.308
DISCOVER Strat	0.455	0.048	0.086	0.400	0.038	0.069
Fisher's Exact Test	0.444	0.038	0.069	0.375	0.028	0.052
MEGSA	0.571	0.075	0.133	0.538	0.066	0.118
MEMO	0.566	0.388	0.460	0.495	0.215	0.300
WExT	0.575	0.438	0.497	0.596	0.295	0.395

Table 80: Metrics for UCEC data on the TSN network constructed with co-expression ratio threshold 0.0, X_2 as the control group and $t = 20$ (Uterus tissue | 142 samples | 1159 CGC-CGC pairs)

Method	Precision	Sensitivity	F1 Score	$\text{Precision}_{\text{strict}}$	$\text{Sensitivity}_{\text{strict}}$	F1 $_{\text{strict}}$ Score
DISCOVER	0.537	0.276	0.365	0.579	0.210	0.308
DISCOVER Strat	0.455	0.048	0.086	0.400	0.038	0.069
Fisher's Exact Test	0.444	0.038	0.069	0.375	0.028	0.052
MEGSA	0.571	0.075	0.133	0.538	0.066	0.118
MEMO	0.566	0.388	0.460	0.495	0.215	0.300
WExT	0.575	0.438	0.497	0.596	0.295	0.395

Table 81: Metrics for UCEC data on the TSN network constructed with co-expression ratio threshold 0.5, X_2 as the control group and $t = 20$ (Uterus tissue | 142 samples | 1078 CGC-CGC pairs)

Method	Precision	Sensitivity	F1 Score	$\text{Precision}_{\text{strict}}$	$\text{Sensitivity}_{\text{strict}}$	F1 $_{\text{strict}}$ Score
DISCOVER	0.537	0.276	0.365	0.579	0.210	0.308
DISCOVER Strat	0.455	0.048	0.086	0.400	0.038	0.069
Fisher's Exact Test	0.444	0.038	0.069	0.375	0.028	0.052
MEGSA	0.571	0.075	0.133	0.538	0.066	0.118
MEMO	0.566	0.388	0.460	0.495	0.215	0.300
WExT	0.575	0.438	0.497	0.596	0.295	0.395

Table 82: Standard deviation values of F1 and F1 $_{strict}$ Scores computed across 20 runs for various robustness_iterations values on COADREAD dataset.

(a) Standard deviation values of F1 scores C = X_1 and $t = 20$

Method	5	50	100	300	500
DISCOVER	0.0062	0.0077	0.0039	0.0042	0.00338
DISCOVER Strat	0.0045	0.00319	0.0017	0.0005	0.00082
Fisher's Exact Test	0.0053	0.00491	0.0053	0.0058	0.0043
MEGSA	0.0050	0.0039	0.0042	0.00317	0.00269
MEMO	0.0069	0.0066	0.0032	0.00292	0.00215
WExT	0.011	0.0048	0.0055	0.0035	0.0031

(b) Standard deviation values of F1 scores C = X_2 and $t = 20$

Method	5	50	100	300	500
DISCOVER	0.0077	0.00387	0.0050	0.0046	0.00459
DISCOVER Strat	0.0059	0.00696	0.0076	0.0072	0.0078
Fisher's Exact Test	0.0078	0.0076	0.0078	0.0018	0.0051
MEGSA	0.0057	0.0051	0.0062	0.00617	0.0035
MEMO	0.0038	0.0044	0.0059	0.0042	0.0046
WExT	0.0058	0.0048	0.0045	0.0039	0.0038

(c) Standard deviation values of F1 $_{strict}$ Score C = X_1 and $t = 20$

Method	5	50	100	300	500
DISCOVER	0.0092	0.00724	0.0049	0.0049	0.00314
DISCOVER Strat	0.0068	0.00387	0.0032	0.00047	0.00048
Fisher's Exact Test	0.0061	0.00643	0.0061	0.0056	0.0037
MEGSA	0.0074	0.00417	0.0043	0.00710	0.00290
MEMO	0.0124	0.00812	0.0045	0.0032	0.0020
WExT	0.017	0.0089	0.0068	0.0044	0.0046

(d) Standard deviation values of F1 $_{strict}$ Score C = X_2 and $t = 20$

Method	5	50	100	300	500
DISCOVER	0.0158	0.0071	0.0091	0.0091	0.00872
DISCOVER Strat	0.0128	0.0099	0.013	0.0085	0.00936
Fisher's Exact Test	0.0119	0.01175	0.0119	0.0057	0.0074
MEGSA	0.0124	0.00561	0.0068	0.0067	0.00377
MEMO	0.0134	0.0116	0.0151	0.0113	0.0097
WExT	0.018	0.0087	0.009	0.0078	0.0094

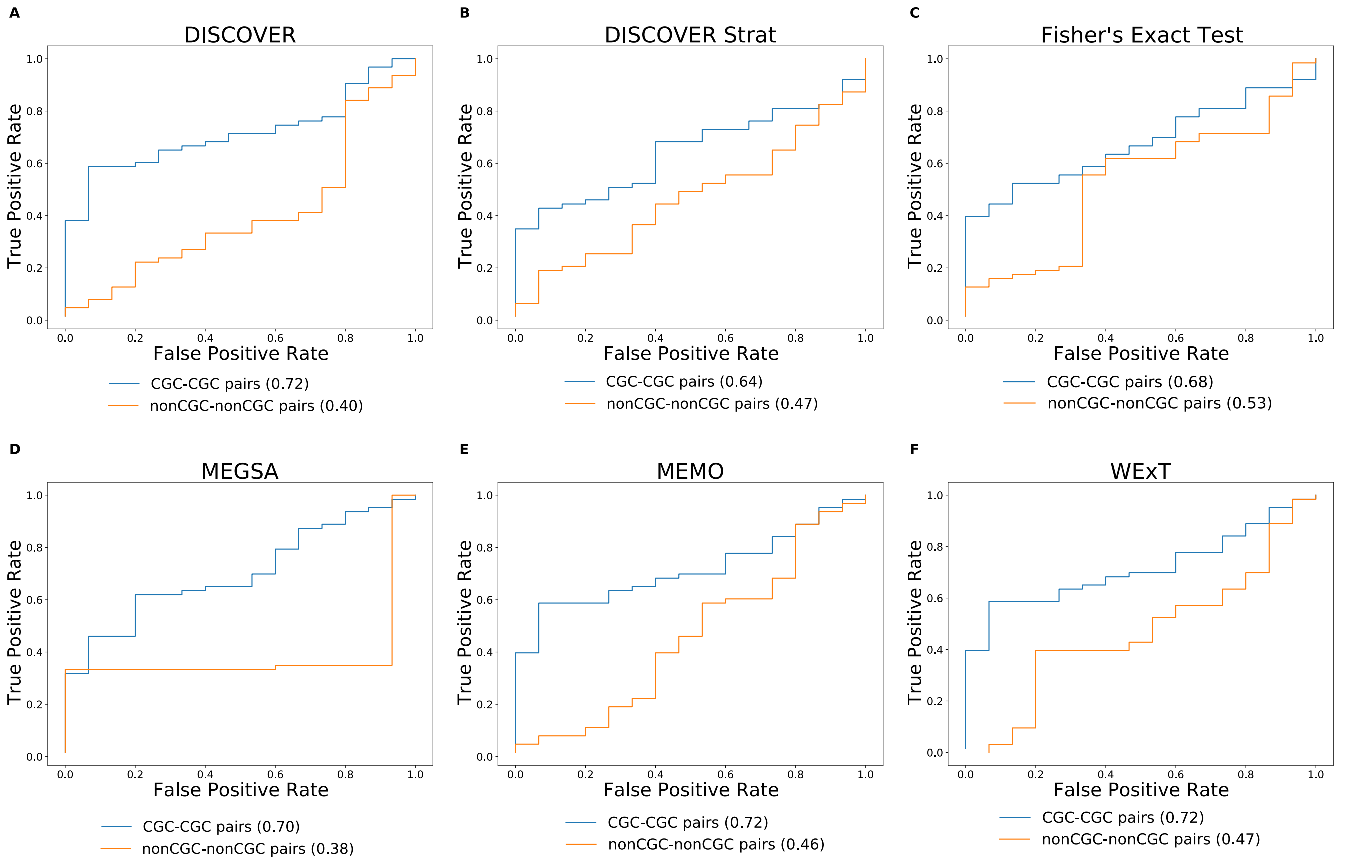

Fig 33: ROC curves for comparing the mutual exclusivities of tissue-specific and non-tissue-specific CGC-CGC gene pairs and non-CGC-non-CGC gene pairs on COADREAD data with $t = 20$ setting. (positive set: 63 edges, negative set: 15 edges)

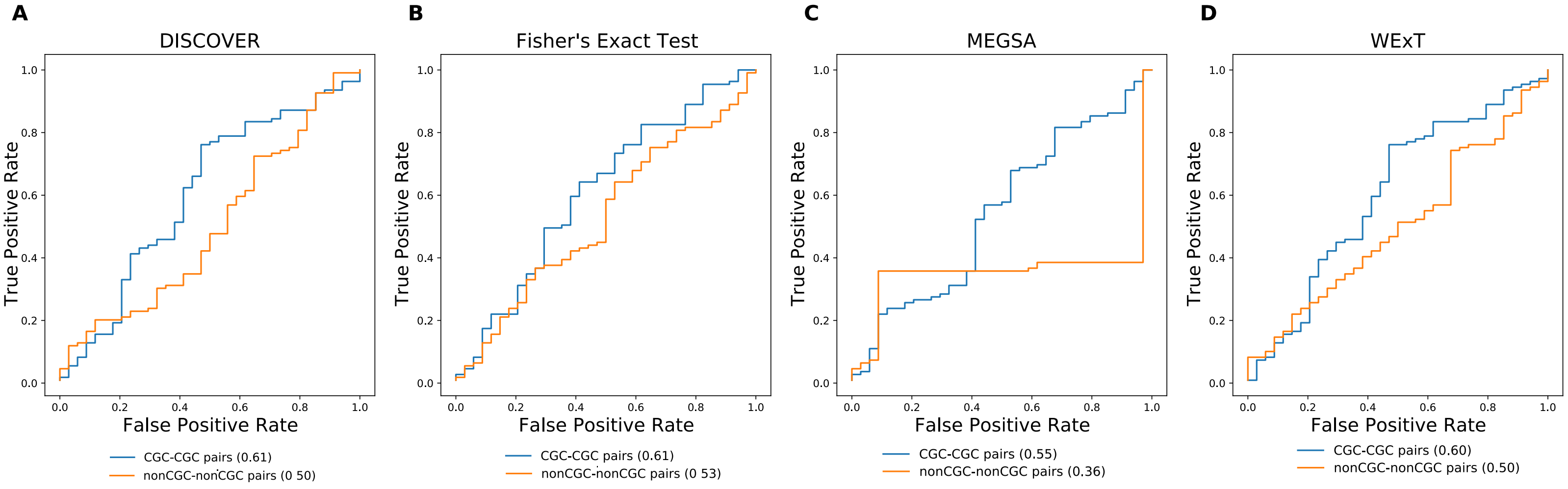

Fig 34: ROC curves for comparing the mutual exclusivities of tissue-specific and non-tissue-specific CGC-CGC gene pairs and non-CGC-non-CGC gene pairs on SKCM data with $t = 20$ setting. (positive set: 109 edges, negative set: 34 edges)

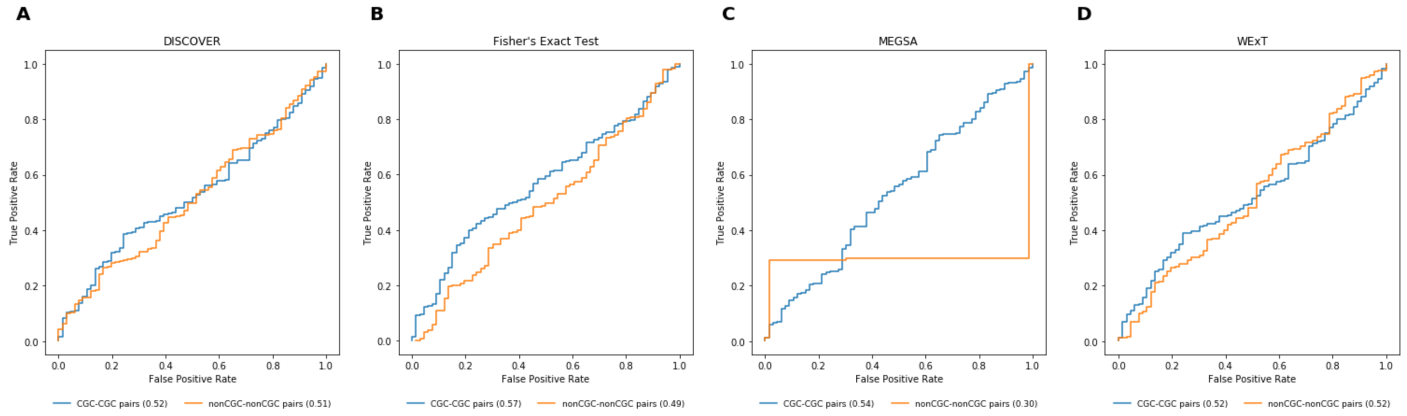

Fig 35: ROC curves for comparing the mutual exclusivities of tissue-specific and non-tissue-specific CGC-CGC gene pairs and non-CGC-non-CGC gene pairs on UCEC data with $t = 20$ setting. (positive set: 519 edges, negative set: 66 edges)

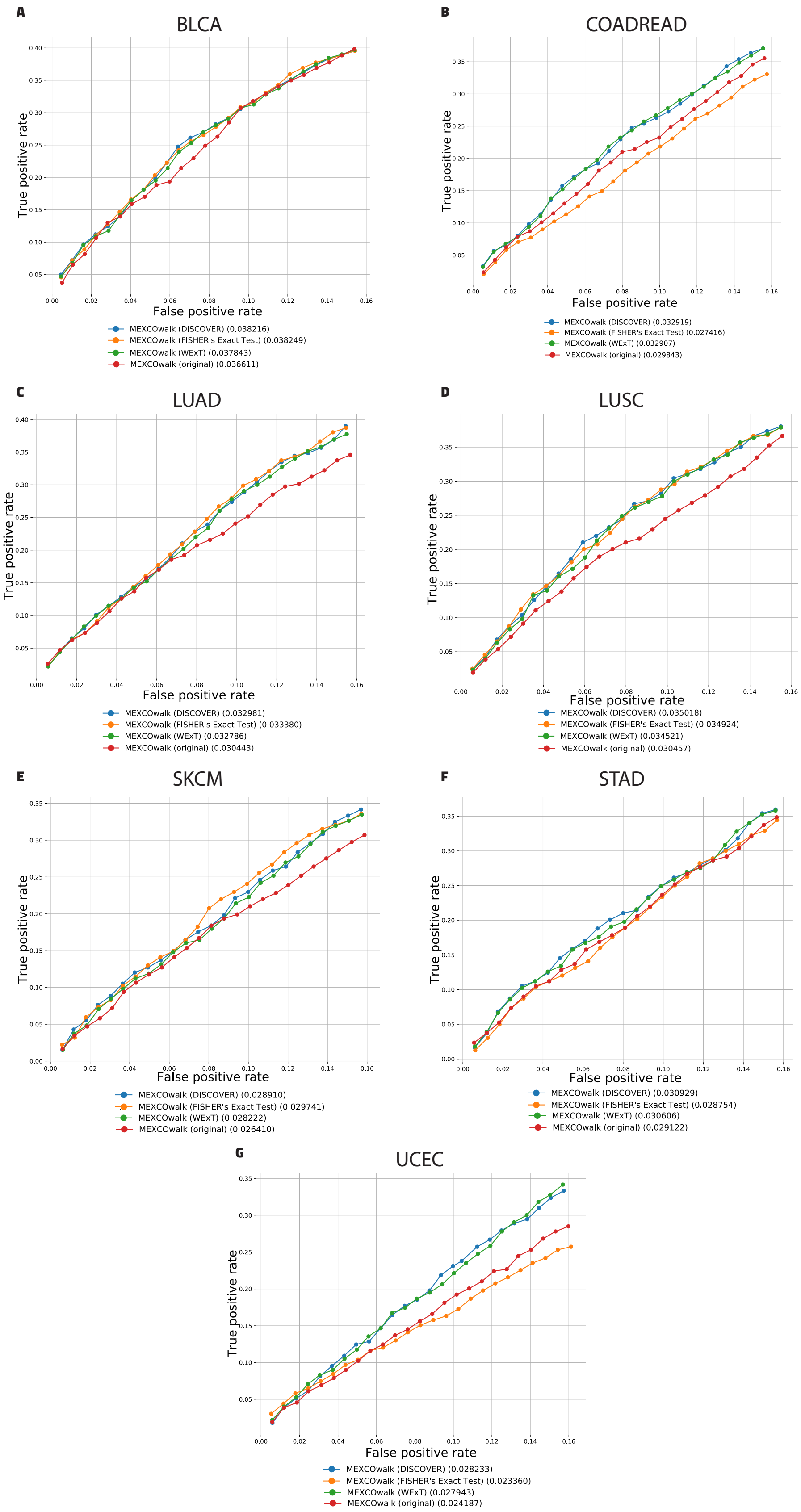

Fig 36: Effect of estimating ME with DISCOVER, Fisher's Exact Test and WExT within the MEXCOwalk model

Table 83: Results of network-centric ME evaluation framework for PCAWG COADREAD data with c=X_1.

(a) Metrics with $\mathcal{G}$ = Intact (w conf. threshold 0.35), $\mathcal{S}$ = CGC, c= X_1, t=20, p_t = 0.05, robustness_iterations = 100. (402 samples | 190 CGC-CGC pairs)

Method	Precision	Sensitivity	F1 Score	$\text{Precision}_{\text{strict}}$	$\text{Sensitivity}_{\text{strict}}$	F1 $_{\text{strict}}$ Score
DISCOVER	0.717	0.182	0.291	0.778	0.155	0.258
Fisher's Exact Test	0.8	0.064	0.119	0.846	0.059	0.11
WExT	0.671	0.299	0.414	0.73	0.25	0.372
MEGSA	0.737	0.074	0.135	0.75	0.063	0.116
MEMO	0.675	0.229	0.342	0.649	0.17	0.269

(b) Metrics with $\mathcal{G}$ = Intact (w conf. threshold 0.35), $\mathcal{S}$ = CGC, c= X_1, t=20, p_t = 0.05, robustness_iterations = 300. (402 samples | 190 CGC-CGC pairs)

Method	Precision	Sensitivity	F1 Score	$\text{Precision}_{\text{strict}}$	$\text{Sensitivity}_{\text{strict}}$	F1 $_{\text{strict}}$ Score
DISCOVER	0.717	0.182	0.29	0.778	0.154	0.257
Fisher's Exact Test	0.8	0.064	0.118	0.846	0.059	0.11
WExT	0.671	0.302	0.417	0.73	0.253	0.376
MEGSA	0.765	0.069	0.127	0.8	0.064	0.119
MEMO	0.667	0.231	0.343	0.646	0.17	0.269

(c) Metrics with $\mathcal{G}$ = Intact (w conf. threshold 0.35), $\mathcal{S}$ = CGC, c= X_1, t=20, p_t = 0.05, robustness_iterations = 500. (402 samples | 190 CGC-CGC pairs)

Method	Precision	Sensitivity	F1 Score	$\text{Precision}_{\text{strict}}$	$\text{Sensitivity}_{\text{strict}}$	F1 $_{\text{strict}}$ Score
DISCOVER	0.708	0.187	0.296	0.757	0.154	0.256
Fisher's Exact Test	0.8	0.065	0.119	0.846	0.059	0.11
WExT	0.667	0.29	0.404	0.714	0.242	0.361
MEGSA	0.765	0.069	0.127	0.8	0.064	0.119
MEMO	0.672	0.238	0.352	0.667	0.177	0.28

(d) Metrics with $\mathcal{G}$ = Intact (w conf. threshold 0.35), $\mathcal{S}$ = CGC, c= X_1, t=20, p_t = 0.01, robustness_iterations = 100. (402 samples | 190 CGC-CGC pairs)

Method	Precision	Sensitivity	F1 Score	$\text{Precision}_{\text{strict}}$	$\text{Sensitivity}_{\text{strict}}$	F1 $_{\text{strict}}$ Score
DISCOVER	0.704	0.102	0.178	0.762	0.086	0.155
Fisher's Exact Test	0.5	0.011	0.021	0.5	0.011	0.022
WExT	0.692	0.196	0.306	0.789	0.163	0.27
MEGSA	0.75	0.032	0.061	0.714	0.026	0.05
MEMO	0.7	0.191	0.3	0.649	0.131	0.218

(e) Metrics with $\mathcal{G}$ = Intact (w conf. threshold 0.35), $\mathcal{S}$ = CGC, c= X_1, t=20, p_t = 0.1, robustness_iterations = 100. (402 samples | 190 CGC-CGC pairs)

Method	Precision	Sensitivity	F1 Score	$\text{Precision}_{\text{strict}}$	$\text{Sensitivity}_{\text{strict}}$	F1 $_{\text{strict}}$ Score
DISCOVER	0.701	0.27	0.389	0.752	0.23	0.352
Fisher's Exact Test	0.714	0.079	0.143	0.765	0.069	0.127
WExT	0.668	0.394	0.496	0.719	0.331	0.453
MEGSA	0.7	0.074	0.134	0.765	0.069	0.127
MEMO	0.695	0.383	0.494	0.701	0.302	0.422

(f) Metrics with $\mathcal{G}$ = Intact (w conf. threshold 0.35), $\mathcal{S}$ = CGC_{SNV}, c= X_1, t=20 p_t = 0.05, robustness_iterations = 100. (402 samples | 126 $CGC_{SNV} - CGC_{SNV}$ pairs)

Method	Precision	Sensitivity	F1 Score	$\text{Precision}_{\text{strict}}$	$\text{Sensitivity}_{\text{strict}}$	F1 $_{\text{strict}}$ Score
DISCOVER	0.743	0.216	0.334	0.815	0.183	0.299
Fisher's Exact Test	0.769	0.079	0.144	0.818	0.071	0.131
WExT	0.679	0.328	0.442	0.727	0.276	0.4
MEGSA	0.8	0.096	0.171	0.846	0.088	0.159
MEMO	0.64	0.234	0.342	0.618	0.172	0.269

(g) Metrics with $\mathcal{G}$ = Intact (w conf. threshold 0.35), $\mathcal{S}$ = Intogen, c= X_1, t=20 p_t = 0.05, robustness_iterations = 100. (402 samples | 204 Intogen-Intogen pairs)

Method	Precision	Sensitivity	F1 Score	$\text{Precision}_{\text{strict}}$	$\text{Sensitivity}_{\text{strict}}$	F1 $_{\text{strict}}$ Score
DISCOVER	0.661	0.182	0.285	0.706	0.152	0.25
Fisher's Exact Test	0.737	0.069	0.126	0.75	0.059	0.109
WExT	0.671	0.293	0.408	0.723	0.242	0.363
MEGSA	0.727	0.079	0.142	0.778	0.069	0.127
MEMO	0.64	0.226	0.335	0.632	0.17	0.268

(h) Metrics with $\mathcal{G}$ = Intact (w conf. threshold 0.25), $\mathcal{S}$ = CGC, c= X_1, t=20, p_t = 0.05, robustness_iterations = 100. (402 samples | 254 CGC-CGC pairs)

Method	Precision	Sensitivity	F1 Score	$\text{Precision}_{\text{strict}}$	$\text{Sensitivity}_{\text{strict}}$	F1 $_{\text{strict}}$ Score
DISCOVER	0.692	0.182	0.288	0.776	0.154	0.257
Fisher's Exact Test	0.769	0.08	0.145	0.818	0.072	0.132
WExT	0.695	0.288	0.408	0.763	0.243	0.369
MEGSA	0.733	0.087	0.155	0.8	0.079	0.144
MEMO	0.647	0.223	0.331	0.587	0.15	0.239

(i) Metrics with $\mathcal{G}$ = Intact (w conf. threshold 0.45), $\mathcal{S}$ = CGC, c= X_1, t=20, p_t = 0.05, robustness_iterations = 100. (402 samples | 88 CGC-CGC pairs)

Method	Precision	Sensitivity	F1 Score	$\text{Precision}_{\text{strict}}$	$\text{Sensitivity}_{\text{strict}}$	F1 $_{\text{strict}}$ Score
DISCOVER	0.762	0.196	0.312	0.875	0.172	0.287
Fisher's Exact Test	0.833	0.115	0.202	0.9	0.103	0.185
WExT	0.657	0.284	0.397	0.704	0.235	0.352
MEGSA	0.769	0.114	0.198	0.818	0.102	0.181
MEMO	0.72	0.226	0.344	0.684	0.164	0.265

(j) Metrics with $\mathcal{G}$ = HINT, $\mathcal{S}$ = CGC, c= X_1, t=20, p_t = 0.05, robustness_iterations = 100. (402 samples | 172 CGC-CGC pairs)

Method	Precision	Sensitivity	F1 Score	$\text{Precision}_{\text{strict}}$	$\text{Sensitivity}_{\text{strict}}$	F1 $_{\text{strict}}$ Score
DISCOVER	0.636	0.166	0.263	0.719	0.136	0.229
Fisher's Exact Test	0.812	0.076	0.14	0.857	0.071	0.131
WExT	0.684	0.321	0.437	0.763	0.278	0.408
MEGSA	0.762	0.093	0.166	0.833	0.087	0.158
MEMO	0.656	0.257	0.369	0.633	0.19	0.292

(k) Metrics with $\mathcal{G}$ = STRING, $\mathcal{S}$ = CGC, c= X_1, t=20 p_t = 0.05, robustness_iterations = 100. (402 samples | 250 CGC-CGC pairs)

Method	Precision	Sensitivity	F1 Score	$\text{Precision}_{\text{strict}}$	$\text{Sensitivity}_{\text{strict}}$	F1 $_{\text{strict}}$ Score
DISCOVER	0.629	0.091	0.159	0.655	0.079	0.141
Fisher's Exact Test	0.857	0.024	0.047	0.857	0.024	0.047
WExT	0.614	0.181	0.28	0.638	0.156	0.251
MEGSA	0.714	0.02	0.039	0.714	0.02	0.039
MEMO	0.624	0.152	0.245	0.608	0.123	0.205

Table 84: Results of network-centric ME evaluation framework for PCAWG COADREAD data with c=X_2.

(a) Metrics with $\mathcal{G}$ = Intact (w conf. threshold 0.35), $\mathcal{S}$ = CGC, c= X_2, t=20, p_t = 0.05, robustness_iterations = 100. (402 samples | 113 CGC-CGC pairs)

Method	Precision	Sensitivity	F1 Score	$\text{Precision}_{\text{strict}}$	$\text{Sensitivity}_{\text{strict}}$	F1 $_{\text{strict}}$ Score
DISCOVER	0.579	0.2	0.297	0.593	0.145	0.233
Fisher's Exact Test	0.625	0.088	0.154	0.615	0.07	0.126
WExT	0.614	0.321	0.422	0.6	0.22	0.322
MEGSA	0.686	0.106	0.183	0.69	0.088	0.156
MEMO	0.577	0.269	0.367	0.444	0.143	0.216

(b) Metrics with $\mathcal{G}$ = Intact (w conf. threshold 0.35), $\mathcal{S}$ = CGC, c= X_2, t=20, p_t = 0.05, robustness_iterations = 300. (402 samples | 113 CGC-CGC pairs)

Method	Precision	Sensitivity	F1 Score	$\text{Precision}_{\text{strict}}$	$\text{Sensitivity}_{\text{strict}}$	F1 $_{\text{strict}}$ Score
DISCOVER	0.579	0.195	0.291	0.593	0.142	0.229
Fisher's Exact Test	0.625	0.088	0.154	0.615	0.07	0.126
WExT	0.614	0.321	0.422	0.625	0.229	0.335
MEGSA	0.667	0.106	0.183	0.667	0.088	0.155
MEMO	0.594	0.267	0.368	0.464	0.142	0.217

(c) Metrics with $\mathcal{G}$ = Intact (w conf. threshold 0.35), $\mathcal{S}$ = CGC, c= X_2, t=20, p_t = 0.05, robustness_iterations = 500. (402 samples | 113 CGC-CGC pairs)

Method	Precision	Sensitivity	F1 Score	$\text{Precision}_{\text{strict}}$	$\text{Sensitivity}_{\text{strict}}$	F1 $_{\text{strict}}$ Score
DISCOVER	0.595	0.2	0.299	0.615	0.145	0.235
Fisher's Exact Test	0.625	0.088	0.155	0.615	0.071	0.127
WExT	0.614	0.315	0.417	0.625	0.225	0.331
MEGSA	0.667	0.105	0.182	0.667	0.088	0.155
MEMO	0.583	0.269	0.368	0.451	0.143	0.217

(d) Metrics with $\mathcal{G}$ = Intact (w conf. threshold 0.35), $\mathcal{S}$ = CGC, c= X_2, t=20, p_t = 0.01, robustness_iterations = 100. (402 samples | 113 CGC-CGC pairs)

Method	Precision	Sensitivity	F1 Score	$\text{Precision}_{\text{strict}}$	$\text{Sensitivity}_{\text{strict}}$	F1 $_{\text{strict}}$ Score
DISCOVER	0.667	0.142	0.234	0.667	0.106	0.183
Fisher's Exact Test	0.333	0.009	0.017	0.333	0.009	0.018
WExT	0.578	0.23	0.329	0.6	0.159	0.251
MEGSA	0.625	0.044	0.083	0.571	0.035	0.066
MEMO	0.605	0.23	0.333	0.448	0.115	0.183

(e) Metrics with $\mathcal{G}$ = Intact (w conf. threshold 0.35), $\mathcal{S}$ = CGC, c= X_2, t=20, p_t = 0.1, robustness_iterations = 100. (402 samples | 113 CGC-CGC pairs)

Method	Precision	Sensitivity	F1 Score	$\text{Precision}_{\text{strict}}$	$\text{Sensitivity}_{\text{strict}}$	F1 $_{\text{strict}}$ Score
DISCOVER	0.582	0.281	0.379	0.585	0.211	0.31
Fisher's Exact Test	0.667	0.106	0.183	0.667	0.088	0.155
WExT	0.627	0.429	0.509	0.639	0.315	0.422
MEGSA	0.647	0.1	0.173	0.643	0.082	0.145
MEMO	0.636	0.393	0.486	0.542	0.243	0.336

(f) Metrics with $\mathcal{G}$ = Intact (w conf. threshold 0.35), $\mathcal{S}$ = CGC_{SNV}, c= X_2, t=20 p_t = 0.05, robustness_iterations = 100. (402 samples | 93 $CGC_{SNV} - CGC_{SNV}$ pairs)

Method	Precision	Sensitivity	F1 Score	$\text{Precision}_{\text{strict}}$	$\text{Sensitivity}_{\text{strict}}$	F1 $_{\text{strict}}$ Score
DISCOVER	0.661	0.204	0.312	0.692	0.149	0.245
Fisher's Exact Test	0.68	0.092	0.162	0.684	0.07	0.127
WExT	0.622	0.308	0.412	0.667	0.242	0.355
MEGSA	0.714	0.108	0.187	0.727	0.086	0.154
MEMO	0.568	0.226	0.323	0.423	0.118	0.185

(g) Metrics with $\mathcal{G}$ = Intact (w conf. threshold 0.35), $\mathcal{S}$ = Intogen, c= X_2, t=20 p_t = 0.05, robustness_iterations = 100. (402 samples | 114 Intogen-Intogen pairs)

Method	Precision	Sensitivity	F1 Score	$\text{Precision}_{\text{strict}}$	$\text{Sensitivity}_{\text{strict}}$	F1 $_{\text{strict}}$ Score
DISCOVER	0.585	0.214	0.314	0.586	0.152	0.241
Fisher's Exact Test	0.658	0.109	0.187	0.645	0.087	0.153
WExT	0.576	0.341	0.428	0.574	0.242	0.34
MEGSA	0.737	0.124	0.212	0.75	0.106	0.186
MEMO	0.582	0.283	0.381	0.447	0.15	0.225

(h) Metrics with $\mathcal{G}$ = Intact (w conf. threshold 0.25), $\mathcal{S}$ = CGC, c= X_2, t=20, p_t = 0.05, robustness_iterations = 100. (402 samples | 175 CGC-CGC pairs)

Method	Precision	Sensitivity	F1 Score	$\text{Precision}_{\text{strict}}$	$\text{Sensitivity}_{\text{strict}}$	F1 $_{\text{strict}}$ Score
DISCOVER	0.614	0.202	0.304	0.675	0.156	0.253
Fisher's Exact Test	0.654	0.096	0.167	0.667	0.079	0.141
WExT	0.626	0.295	0.401	0.667	0.22	0.331
MEGSA	0.712	0.106	0.185	0.744	0.092	0.164
MEMO	0.597	0.244	0.347	0.46	0.131	0.204

(i) Metrics with $\mathcal{G}$ = Intact (w conf. threshold 0.45), $\mathcal{S}$ = CGC, c= X_2, t=20, p_t = 0.05, robustness_iterations = 100. (402 samples | 26 CGC-CGC pairs)

Method	Precision	Sensitivity	F1 Score	$\text{Precision}_{\text{strict}}$	$\text{Sensitivity}_{\text{strict}}$	F1 $_{\text{strict}}$ Score	
DISCOVER	0.5	0.308	0.692	0.381	0.6	0.231	0.334
Fisher's Exact Test	0.625	0.2	0.88	0.303	0.667	0.16	0.258
WExT	0.529	0.346	0.692	0.419	0.5	0.192	0.277
MEGSA	0.647	0.216	0.882	0.324	0.692	0.176	0.281
MEMO	0.5	0.308	0.692	0.381	0.2	0.077	0.111

(j) Metrics with $\mathcal{G}$ = HINT, $\mathcal{S}$ = CGC, c= X_2, t=20, p_t = 0.05, robustness_iterations = 100. (402 samples | 58 CGC-CGC pairs)

Method	Precision	Sensitivity	F1 Score	$\text{Precision}_{\text{strict}}$	$\text{Sensitivity}_{\text{strict}}$	F1 $_{\text{strict}}$ Score
DISCOVER	0.486	0.277	0.353	0.52	0.2	0.289
Fisher's Exact Test	0.6	0.134	0.22	0.636	0.104	0.179
WExT	0.537	0.439	0.483	0.568	0.318	0.408
MEGSA	0.625	0.149	0.241	0.667	0.119	0.202
MEMO	0.52	0.391	0.446	0.353	0.18	0.238

(k) Metrics with $\mathcal{G}$ = STRING, $\mathcal{S}$ = CGC, c= X_2, t=20 p_t = 0.05, robustness_iterations = 100. (402 samples | 106 CGC-CGC pairs)

Method	Precision	Sensitivity	F1 Score	$\text{Precision}_{\text{strict}}$	$\text{Sensitivity}_{\text{strict}}$	F1 $_{\text{strict}}$ Score
DISCOVER	0.488	0.096	0.161	0.516	0.077	0.134
Fisher's Exact Test	0.833	0.048	0.09	0.833	0.048	0.091
WExT	0.517	0.142	0.222	0.55	0.104	0.175
MEGSA	0.833	0.047	0.089	0.833	0.047	0.089
MEMO	0.478	0.106	0.174	0.375	0.058	0.1

Table 85: Degree-normalized network-centric evaluations with t = 20 for PCAWG COADREAD data.

(a) Metrics with c= X_1

Method	Precision	Sensitivity	F1 Score	$\text{Precision}_{\text{strict}}$	$\text{Sensitivity}_{\text{strict}}$	F1 $_{\text{strict}}$ Score
DISCOVER	0.88	0.243	0.67	0.912	0.226	0.674
Fisher's Exact Test	0.792	0.023	0.435	0.792	0.023	0.435
WExT	0.857	0.414	0.743	0.86	0.373	0.739
MEMO	0.832	0.353	0.738	0.824	0.307	0.743
MEGSA	0.865	0.059	0.55	0.852	0.057	0.537

(b) Metrics with c= X_2

Method	Precision	Sensitivity	F1 Score	$\text{Precision}_{\text{strict}}$	$\text{Sensitivity}_{\text{strict}}$	F1 $_{\text{strict}}$ Score
DISCOVER	0.664	0.821	0.673	0.712	0.228	0.683
Fisher's Exact Test	0.458	0.988	0.283	0.458	0.01	0.283
WExT	0.729	0.775	0.721	0.724	0.327	0.726
MEMO	0.682	0.78	0.722	0.726	0.306	0.714
MEGSA	0.79	0.982	0.653	0.767	0.068	0.629

Results of network-centric ME evaluation framework run on the tissue-specific network (TSN) with $t = 20$

Table 86: Metrics for PCAWG COADREAD data on the TSN networks

(a) Metrics for PCAWG COADREAD data on the TSN network constructed with co-expression ratio threshold 0.0, control group X_1 and $t = 20$ (Colon tissue | 779 samples | 186 CGC-CGC pairs)

Method	Precision	Sensitivity	F1 Score	$\text{Precision}_{\text{strict}}$	$\text{Sensitivity}_{\text{strict}}$	F1 $_{\text{strict}}$ Score
DISCOVER	0.723	0.193	0.304	0.806	0.164	0.273
Fisher's Exact Test	0.8	0.066	0.121	0.769	0.055	0.103
WExT	0.675	0.305	0.42	0.726	0.254	0.376
MEGSA	0.765	0.071	0.129	0.8	0.065	0.12
MEMO	0.667	0.246	0.36	0.66	0.185	0.289

(b) Metrics for PCAWG COADREAD data on the TSN network constructed with co-expression ratio threshold 0.5, control group X_1 and $t = 20$ (Colon tissue | 779 samples | 178 CGC-CGC pairs)

Method	Precision	Sensitivity	F1 Score	$\text{Precision}_{\text{strict}}$	$\text{Sensitivity}_{\text{strict}}$	F1 $_{\text{strict}}$ Score
DISCOVER	0.708	0.201	0.313	0.757	0.165	0.271
Fisher's Exact Test	0.8	0.069	0.126	0.846	0.063	0.117
WExT	0.679	0.314	0.43	0.73	0.263	0.387
MEGSA	0.765	0.074	0.135	0.8	0.068	0.125
MEMO	0.677	0.257	0.372	0.673	0.192	0.299

(c) Metrics for PCAWG COADREAD data on the TSN network constructed with co-expression ratio threshold 0.0, control group X_2 and $t = 20$ (Colon tissue | 779 samples | 111 CGC-CGC pairs)

Method	Precision	Sensitivity	F1 Score	$\text{Precision}_{\text{strict}}$	$\text{Sensitivity}_{\text{strict}}$	F1 $_{\text{strict}}$ Score
DISCOVER	0.575	0.207	0.305	0.586	0.153	0.243
Fisher's Exact Test	0.625	0.09	0.157	0.615	0.072	0.129
WExT	0.6	0.321	0.419	0.595	0.223	0.324
MEGSA	0.667	0.108	0.186	0.667	0.09	0.159
MEMO	0.588	0.274	0.374	0.457	0.146	0.221

(d) Metrics for PCAWG COADREAD data on the TSN network constructed with co-expression ratio threshold 0.5, control group X_1 and $t = 20$ (Colon tissue | 779 samples | 105 CGC-CGC pairs)

Method	Precision	Sensitivity	F1 Score	$\text{Precision}_{\text{strict}}$	$\text{Sensitivity}_{\text{strict}}$	F1 $_{\text{strict}}$ Score
DISCOVER	0.56	0.202	0.297	0.566	0.144	0.23
Fisher's Exact Test	0.667	0.095	0.167	0.667	0.076	0.136
WExT	0.586	0.33	0.422	0.585	0.233	0.333
MEGSA	0.706	0.114	0.197	0.714	0.095	0.168
MEMO	0.58	0.278	0.375	0.441	0.144	0.217

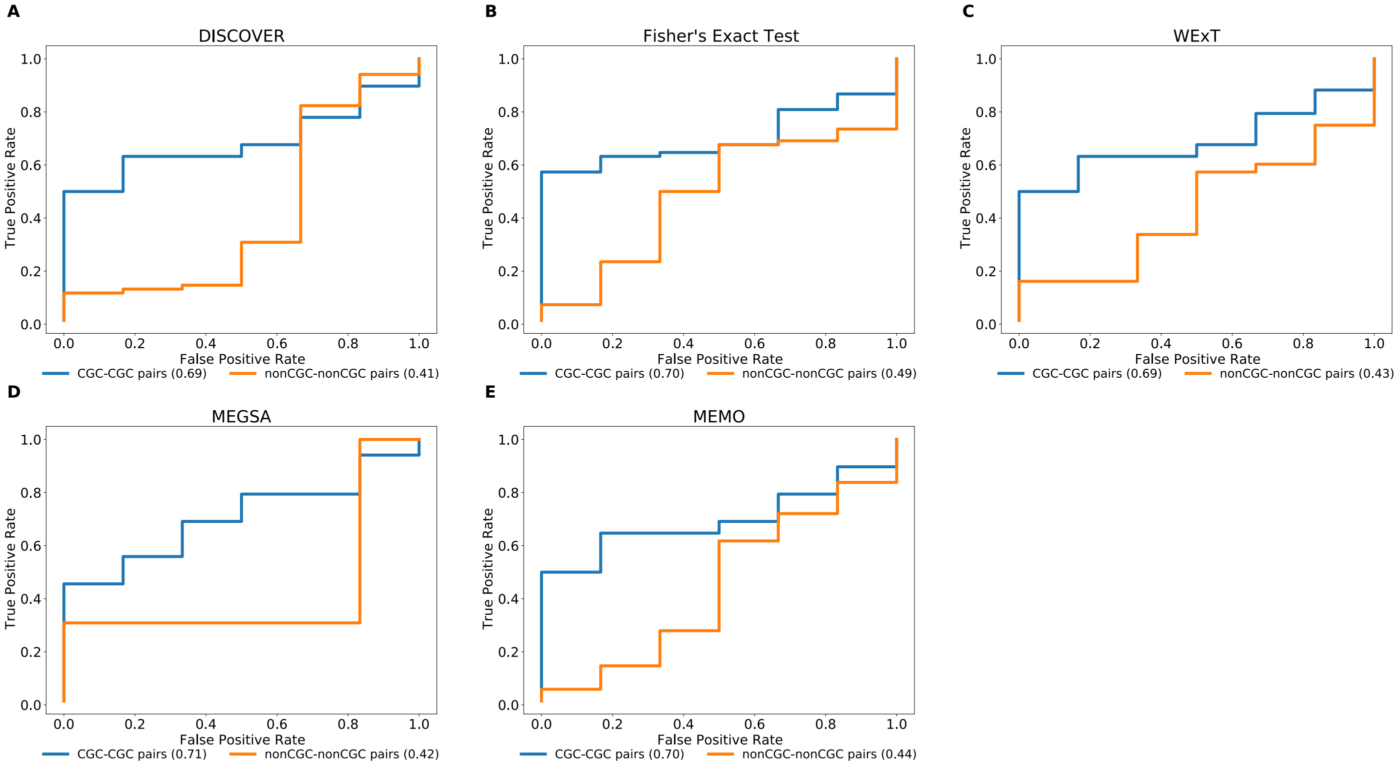

Fig 37: ROC curves for comparing the mutual exclusivities of tissue-specific and non-tissue-specific CGC-CGC gene pairs and non-CGC-non-CGC gene pairs on PCAWG COADREAD data with $t = 20$ setting. (positive set: 68 edges, negative set: 6 edges)

Scatterplots of percentage significance of mutual exclusivity runs vs mutation load association (MLA) when $t = 20$ for PCAWG COADREAD data

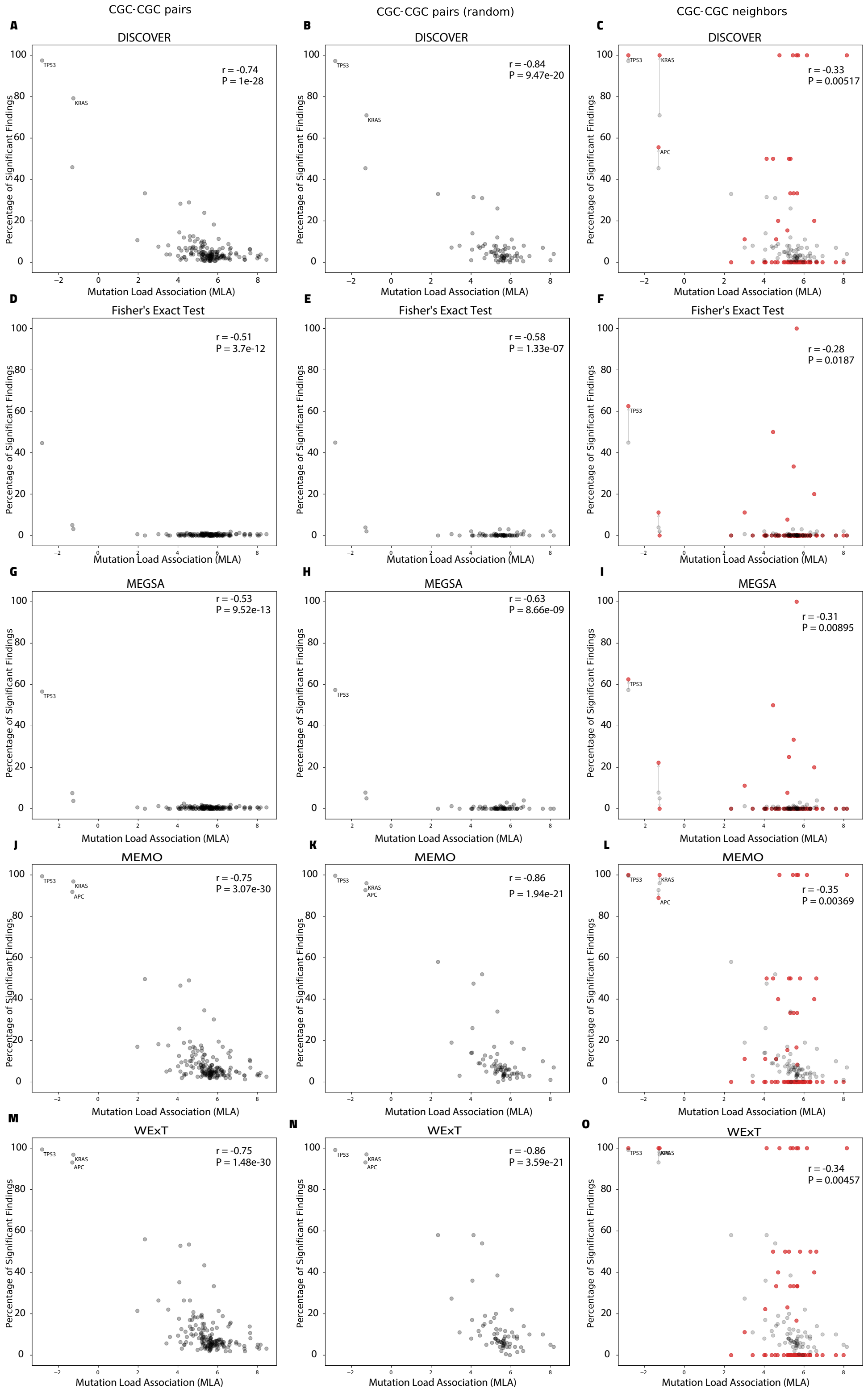

Fig 38: Comparison of ME results of all considered methods on PCAWG COADREAD cohort with $t = 20$ (402 samples). The scatterplots show the percentage significance of ME runs (p-value<0.05) versus MLA values for CGC genes. (A) Results of DISCOVER where tests are performed between a CGC gene and all other CGC genes. B) Results of DISCOVER where tests are performed between a CGC gene and a random subset of all other CGC genes so that ME of a CGC gene of interest is checked with same sized group of genes in both B and C. (C) Results of DISCOVER where tests are performed between a CGC gene and its PPI neighbors that are in CGC (red) compared with (B) in gray. Analogous results are shown for Fisher's Exact Test (D, E, F), MEGSA (G, H, I), MEMO (J, K, L) and WExT (M, N, O) where coloring is the same as previously described for (C).

Table 87: Results of network-centric ME evaluation framework for COADREAD data with filtration based on [Muinos et al., 2021] Results with c=X_1.

(a) Metrics with $\mathcal{G}$ = Intact (w conf. threshold 0.35), $\mathcal{S}$ = CGC, c= X_1, t=20, p_t = 0.05, robustness_iterations = 100. (498 samples | 196 CGC-CGC pairs)

Method	Precision	Sensitivity	F1 Score	Precision$_{\text{strict}}$	Sensitivity$_{\text{strict}}$	F1 $_{\text{strict}}$ Score
DISCOVER	0.614	0.274	0.379	0.697	0.222	0.337
DISCOVER Strat	0.66	0.165	0.264	0.692	0.144	0.238
Fisher's Exact Test	0.632	0.125	0.208	0.679	0.099	0.173
WExT	0.635	0.376	0.472	0.665	0.285	0.399
MEMO	0.632	0.34	0.442	0.56	0.211	0.307
MEGSA	0.634	0.133	0.22	0.667	0.103	0.178

(b) Metrics with $\mathcal{G}$ = Intact (w conf. threshold 0.35), $\mathcal{S}$ = CGC, c= X_1, t=20, p_t = 0.05, robustness_iterations = 300. (498 samples | 196 CGC-CGC pairs)

Method	Precision	Sensitivity	F1 Score	Precision$_{\text{strict}}$	Sensitivity$_{\text{strict}}$	F1 $_{\text{strict}}$ Score
DISCOVER	0.605	0.275	0.378	0.661	0.217	0.327
DISCOVER Strat	0.674	0.165	0.266	0.718	0.149	0.247
Fisher's Exact Test	0.61	0.128	0.211	0.633	0.097	0.168
WExT	0.647	0.377	0.476	0.707	0.301	0.422
MEMO	0.62	0.336	0.436	0.545	0.209	0.302
MEGSA	0.634	0.134	0.221	0.667	0.103	0.178

(c) Metrics with $\mathcal{G}$ = Intact (w conf. threshold 0.35), $\mathcal{S}$ = CGC, c= X_1, t=20, p_t = 0.05, robustness_iterations = 500. (498 samples | 196 CGC-CGC pairs)

Method	Precision	Sensitivity	F1 Score	Precision$_{\text{strict}}$	Sensitivity$_{\text{strict}}$	F1 $_{\text{strict}}$ Score
DISCOVER	0.601	0.274	0.377	0.654	0.213	0.321
DISCOVER Strat	0.682	0.161	0.261	0.73	0.145	0.242
Fisher's Exact Test	0.615	0.122	0.203	0.655	0.096	0.167
WExT	0.645	0.384	0.481	0.7	0.303	0.423
MEMO	0.624	0.333	0.434	0.549	0.206	0.3
MEGSA	0.634	0.134	0.221	0.667	0.103	0.178

(d) Metrics with $\mathcal{G}$ = Intact (w conf. threshold 0.35), $\mathcal{S}$ = CGC, c= X_1, t=20, p_t = 0.01, robustness_iterations = 100. (498 samples | 196 CGC-CGC pairs)

Method	Precision	Sensitivity	F1 Score	Precision$_{\text{strict}}$	Sensitivity$_{\text{strict}}$	F1 $_{\text{strict}}$ Score
DISCOVER	0.671	0.259	0.373	0.717	0.201	0.314
DISCOVER Strat	0.8	0.063	0.116	0.8	0.063	0.117
Fisher's Exact Test	0.645	0.102	0.177	0.696	0.082	0.147
WExT	0.635	0.286	0.395	0.712	0.223	0.34
MEMO	0.633	0.263	0.372	0.554	0.163	0.252
MEGSA	0.606	0.102	0.175	0.625	0.077	0.137

(e) Metrics with $\mathcal{G}$ = Intact (w conf. threshold 0.35), $\mathcal{S}$ = CGC, c= X_1, t=20, p_t = 0.1, robustness_iterations = 100. (498 samples | 196 CGC-CGC pairs)

Method	Precision	Sensitivity	F1 Score	Precision$_{\text{strict}}$	Sensitivity$_{\text{strict}}$	F1 $_{\text{strict}}$ Score
DISCOVER	0.641	0.362	0.462	0.701	0.296	0.416
DISCOVER Strat	0.647	0.249	0.359	0.685	0.21	0.321
Fisher's Exact Test	0.64	0.144	0.235	0.667	0.11	0.189
WExT	0.631	0.43	0.512	0.708	0.35	0.468
MEMO	0.647	0.418	0.508	0.591	0.283	0.383
MEGSA	0.692	0.185	0.291	0.737	0.144	0.241

(f) Metrics with $\mathcal{G}$ = Intact (w conf. threshold 0.35), $\mathcal{S}$ = CGC_{SNV}, c= X_1, t=20 p_t = 0.05, robustness_iterations = 100. (498 samples | 146 $CGC_{SNV} - CGC_{SNV}$ pairs)

Method	Precision	Sensitivity	F1 Score	$\text{Precision}_{\text{strict}}$	$\text{Sensitivity}_{\text{strict}}$	F1 $_{\text{strict}}$ Score
DISCOVER	0.594	0.268	0.369	0.644	0.204	0.31
DISCOVER Strat	0.581	0.129	0.212	0.607	0.122	0.203
Fisher's Exact Test	0.524	0.076	0.132	0.5	0.055	0.099
WExT	0.605	0.366	0.456	0.638	0.276	0.385
MEMO	0.586	0.326	0.419	0.523	0.206	0.296
MEGSA	0.583	0.097	0.166	0.556	0.069	0.123

(g) Metrics with $\mathcal{G}$ = Intact (w conf. threshold 0.35), $\mathcal{S}$ = Intogen, c= X_1, t=20 p_t = 0.05, robustness_iterations = 100. (498 samples | 206 Intogen-Intogen pairs)

Method	Precision	Sensitivity	F1 Score	$\text{Precision}_{\text{strict}}$	$\text{Sensitivity}_{\text{strict}}$	F1 $_{\text{strict}}$ Score
DISCOVER	0.621	0.287	0.392	0.679	0.226	0.339
DISCOVER Strat	0.653	0.155	0.251	0.699	0.145	0.24
Fisher's Exact Test	0.611	0.108	0.184	0.63	0.084	0.148
WExT	0.642	0.388	0.484	0.69	0.307	0.425
MEMO	0.624	0.35	0.448	0.545	0.216	0.309
MEGSA	0.618	0.114	0.193	0.632	0.088	0.154

(h) Metrics with $\mathcal{G}$ = Intact (w conf. threshold 0.25), $\mathcal{S}$ = CGC, c= X_1, t=20, p_t = 0.05, robustness_iterations = 100. (498 samples | 262 CGC-CGC pairs)

Method	Precision	Sensitivity	F1 Score	$\text{Precision}_{\text{strict}}$	$\text{Sensitivity}_{\text{strict}}$	F1 $_{\text{strict}}$ Score
DISCOVER	0.607	0.253	0.357	0.667	0.198	0.305
DISCOVER Strat	0.629	0.145	0.236	0.646	0.127	0.212
Fisher's Exact Test	0.612	0.115	0.194	0.649	0.092	0.161
WExT	0.637	0.347	0.449	0.691	0.27	0.388
MEMO	0.621	0.319	0.421	0.547	0.196	0.289
MEGSA	0.62	0.119	0.2	0.658	0.096	0.168

(i) Metrics with $\mathcal{G}$ = Intact (w conf. threshold 0.45), $\mathcal{S}$ = CGC, c= X_1, t=20, p_t = 0.05, robustness_iterations = 100. (498 samples | 100 CGC-CGC pairs)

Method	Precision	Sensitivity	F1 Score	$\text{Precision}_{\text{strict}}$	$\text{Sensitivity}_{\text{strict}}$	F1 $_{\text{strict}}$ Score
DISCOVER	0.634	0.268	0.377	0.75	0.216	0.335
DISCOVER Strat	0.622	0.123	0.205	0.697	0.123	0.209
Fisher's Exact Test	0.5	0.036	0.066	0.462	0.03	0.056
WExT	0.624	0.36	0.456	0.709	0.296	0.418
MEMO	0.611	0.305	0.407	0.585	0.2	0.298
MEGSA	0.556	0.051	0.093	0.5	0.04	0.074

(j) Metrics with $\mathcal{G}$ = HINT, $\mathcal{S}$ = CGC, c= X_1, t=20, p_t = 0.05, robustness_iterations = 100. (498 samples | 198 CGC-CGC pairs)

Method	Precision	Sensitivity	F1 Score	$\text{Precision}_{\text{strict}}$	$\text{Sensitivity}_{\text{strict}}$	F1 $_{\text{strict}}$ Score
DISCOVER	0.612	0.291	0.395	0.685	0.227	0.341
DISCOVER Strat	0.674	0.165	0.265	0.718	0.149	0.247
Fisher's Exact Test	0.676	0.129	0.216	0.741	0.103	0.181
WExT	0.596	0.341	0.434	0.646	0.268	0.379
MEMO	0.607	0.325	0.424	0.532	0.197	0.288
MEGSA	0.658	0.128	0.214	0.714	0.102	0.178

(k) Metrics with $\mathcal{G}$ = STRING, $\mathcal{S}$ = CGC, c= X_1, t=20 p_t = 0.05, robustness_iterations = 100. (498 samples | 292 CGC-CGC pairs)

Method	Precision	Sensitivity	F1 Score	$\text{Precision}_{\text{strict}}$	$\text{Sensitivity}_{\text{strict}}$	F1 $_{\text{strict}}$ Score
DISCOVER	0.617	0.289	0.393	0.716	0.239	0.358
DISCOVER Strat	0.721	0.201	0.315	0.78	0.18	0.292
Fisher's Exact Test	0.727	0.055	0.103	0.737	0.048	0.09
WExT	0.585	0.314	0.409	0.675	0.257	0.372
MEMO	0.589	0.294	0.392	0.58	0.206	0.304
MEGSA	0.72	0.062	0.114	0.762	0.055	0.103

Table 88: Results of network-centric ME evaluation framework for COADREAD data with filtration based on (Muiños et al., 2021). Results with c=X_2.

(a) Metrics with $\mathcal{G}$ = Intact (w conf. threshold 0.35), $\mathcal{S}$ = CGC, c= X_2, t=20, p_t = 0.05, robustness_iterations = 100. (498 samples | 126 CGC-CGC pairs)

Method	Precision	Sensitivity	F1 Score	$\text{Precision}_{\text{strict}}$	$\text{Sensitivity}_{\text{strict}}$	F1 $_{\text{strict}}$ Score
DISCOVER	0.552	0.346	0.425	0.545	0.224	0.318
DISCOVER Strat	0.513	0.189	0.276	0.448	0.123	0.193
Fisher's Exact Test	0.517	0.142	0.222	0.421	0.075	0.127
WExT	0.543	0.419	0.473	0.569	0.276	0.372
MEMO	0.553	0.393	0.459	0.438	0.196	0.271
MEGSA	0.529	0.17	0.257	0.455	0.094	0.156

(b) Metrics with $\mathcal{G}$ = Intact (w conf. threshold 0.35), $\mathcal{S}$ = CGC, c= X_2, t=20, p_t = 0.05, robustness_iterations = 300. (498 samples | 126 CGC-CGC pairs)

Method	Precision	Sensitivity	F1 Score	$\text{Precision}_{\text{strict}}$	$\text{Sensitivity}_{\text{strict}}$	F1 $_{\text{strict}}$ Score
DISCOVER	0.561	0.346	0.428	0.568	0.234	0.331
DISCOVER Strat	0.514	0.181	0.268	0.483	0.133	0.209
Fisher's Exact Test	0.531	0.157	0.243	0.476	0.093	0.156
WExT	0.549	0.425	0.479	0.577	0.283	0.38
MEMO	0.553	0.393	0.459	0.417	0.187	0.258
MEGSA	0.529	0.17	0.257	0.455	0.094	0.156

(c) Metrics with $\mathcal{G}$ = Intact (w conf. threshold 0.35), $\mathcal{S}$ = CGC, c= X_2, t=20, p_t = 0.05, robustness_iterations = 500. (498 samples | 126 CGC-CGC pairs)

Method	Precision	Sensitivity	F1 Score	$\text{Precision}_{\text{strict}}$	$\text{Sensitivity}_{\text{strict}}$	F1 $_{\text{strict}}$ Score
DISCOVER	0.561	0.349	0.43	0.568	0.236	0.333
DISCOVER Strat	0.514	0.181	0.268	0.464	0.124	0.196
Fisher's Exact Test	0.516	0.15	0.232	0.45	0.084	0.142
WExT	0.549	0.425	0.479	0.577	0.283	0.38
MEMO	0.554	0.387	0.456	0.415	0.184	0.255
MEGSA	0.529	0.17	0.257	0.455	0.094	0.156

(d) Metrics with $\mathcal{G}$ = Intact (w conf. threshold 0.35), $\mathcal{S}$ = CGC, c= X_2, t=20, p_t = 0.01, robustness_iterations = 100. (498 samples | 126 CGC-CGC pairs)

Method	Precision	Sensitivity	F1 Score	$\text{Precision}_{\text{strict}}$	$\text{Sensitivity}_{\text{strict}}$	F1 $_{\text{strict}}$ Score
DISCOVER	0.607	0.318	0.417	0.595	0.206	0.306
DISCOVER Strat	0.4	0.056	0.098	0.385	0.046	0.082
Fisher's Exact Test	0.538	0.13	0.209	0.471	0.074	0.128
WExT	0.6	0.336	0.431	0.59	0.215	0.315
MEMO	0.593	0.33	0.424	0.385	0.142	0.207
MEGSA	0.538	0.131	0.211	0.471	0.075	0.129

(e) Metrics with $\mathcal{G}$ = Intact (w conf. threshold 0.35), $\mathcal{S}$ = CGC, c= X_2, t=20, p_t = 0.1, robustness_iterations = 100. (498 samples | 126 CGC-CGC pairs)

Method	Precision	Sensitivity	F1 Score	$\text{Precision}_{\text{strict}}$	$\text{Sensitivity}_{\text{strict}}$	F1 $_{\text{strict}}$ Score
DISCOVER	0.538	0.406	0.462	0.538	0.264	0.354
DISCOVER Strat	0.588	0.286	0.385	0.553	0.2	0.294
Fisher's Exact Test	0.565	0.185	0.279	0.511	0.109	0.18
WExT	0.558	0.457	0.503	0.582	0.305	0.4
MEMO	0.56	0.439	0.492	0.454	0.229	0.304
MEGSA	0.628	0.252	0.36	0.586	0.159	0.25

(f) Metrics with $\mathcal{G}$ = Intact (w conf. threshold 0.35), $\mathcal{S}$ = CGC_{SNV}, c= X_2, t=20 p_t = 0.05, robustness_iterations = 100. (498 samples | 101 $CGC_{SNV} - CGC_{SNV}$ pairs)

Method	Precision	Sensitivity	F1 Score	$\text{Precision}_{\text{strict}}$	$\text{Sensitivity}_{\text{strict}}$	F1 $_{\text{strict}}$ Score
DISCOVER	0.583	0.28	0.378	0.578	0.185	0.28
DISCOVER Strat	0.491	0.14	0.218	0.432	0.095	0.156
Fisher's Exact Test	0.444	0.079	0.134	0.25	0.03	0.054
WExT	0.574	0.39	0.464	0.578	0.26	0.359
MEMO	0.588	0.337	0.428	0.507	0.191	0.277
MEGSA	0.5	0.1	0.166	0.357	0.05	0.088

(g) Metrics with $\mathcal{G}$ = Intact (w conf. threshold 0.35), $\mathcal{S}$ = Intogen, c= X_2, t=20 p_t = 0.05, robustness_iterations = 100. (498 samples | 105 Intogen-Intogen pairs)

Method	Precision	Sensitivity	F1 Score	$\text{Precision}_{\text{strict}}$	$\text{Sensitivity}_{\text{strict}}$	F1 $_{\text{strict}}$ Score
DISCOVER	0.559	0.358	0.437	0.545	0.226	0.32
DISCOVER Strat	0.487	0.181	0.264	0.433	0.124	0.193
Fisher's Exact Test	0.483	0.133	0.209	0.421	0.076	0.129
WExT	0.571	0.457	0.508	0.585	0.295	0.392
MEMO	0.579	0.427	0.492	0.458	0.214	0.292
MEGSA	0.5	0.153	0.234	0.429	0.086	0.143

(h) Metrics with $\mathcal{G}$ = Intact (w conf. threshold 0.25), $\mathcal{S}$ = CGC, c= X_2, t=20, p_t = 0.05, robustness_iterations = 100. (498 samples | 175 CGC-CGC pairs)

Method	Precision	Sensitivity	F1 Score	$\text{Precision}_{\text{strict}}$	$\text{Sensitivity}_{\text{strict}}$	F1 $_{\text{strict}}$ Score
DISCOVER	0.588	0.287	0.386	0.605	0.198	0.298
DISCOVER Strat	0.536	0.149	0.234	0.539	0.118	0.194
Fisher's Exact Test	0.561	0.133	0.215	0.552	0.092	0.158
WExT	0.574	0.358	0.441	0.597	0.249	0.351
MEMO	0.592	0.335	0.428	0.469	0.173	0.253
MEGSA	0.553	0.149	0.234	0.545	0.103	0.173

(i) Metrics with $\mathcal{G}$ = Intact (w conf. threshold 0.45), $\mathcal{S}$ = CGC, c= X_2, t=20, p_t = 0.05, robustness_iterations = 100. (498 samples | 15 CGC-CGC pairs)

Method	Precision	Sensitivity	F1 Score	$\text{Precision}_{\text{strict}}$	$\text{Sensitivity}_{\text{strict}}$	F1 $_{\text{strict}}$ Score
DISCOVER	0.467	0.467	0.467	0.444	0.267	0.333
DISCOVER Strat	0.2	0.071	0.105	0.2	0.071	0.105
Fisher's Exact Test	0.333	0.071	0.118	0.333	0.071	0.117
WExT	0.529	0.6	0.562	0.5	0.333	0.4
MEMO	0.5	0.533	0.516	0.3	0.2	0.24
MEGSA	0.4	0.133	0.2	0.25	0.067	0.106

(j) Metrics with $\mathcal{G}$ = HINT, $\mathcal{S}$ = CGC, c= X_2, t=20, p_t = 0.05, robustness_iterations = 100. (498 samples | 58 CGC-CGC pairs)

Method	Precision	Sensitivity	F1 Score	$\text{Precision}_{\text{strict}}$	$\text{Sensitivity}_{\text{strict}}$	F1 $_{\text{strict}}$ Score
DISCOVER	0.511	0.404	0.451	0.464	0.228	0.306
DISCOVER Strat	0.467	0.237	0.315	0.455	0.169	0.246
Fisher's Exact Test	0.517	0.254	0.341	0.444	0.136	0.208
WExT	0.5	0.483	0.491	0.486	0.293	0.366
MEMO	0.538	0.491	0.514	0.303	0.175	0.222
MEGSA	0.5	0.241	0.326	0.471	0.138	0.213

(k) Metrics with $\mathcal{G}$ = STRING, $\mathcal{S}$ = CGC, c= X_2, t=20 p_t = 0.05, robustness_iterations = 100. (498 samples | 116 CGC-CGC pairs)

Method	Precision	Sensitivity	F1 Score	$\text{Precision}_{\text{strict}}$	$\text{Sensitivity}_{\text{strict}}$	F1 $_{\text{strict}}$ Score
DISCOVER	0.632	0.29	0.398	0.74	0.234	0.356
DISCOVER Strat	0.667	0.218	0.329	0.721	0.192	0.303
Fisher's Exact Test	0.706	0.104	0.182	0.786	0.096	0.171
WExT	0.561	0.319	0.407	0.674	0.25	0.365
MEMO	0.58	0.299	0.394	0.494	0.169	0.252
MEGSA	0.684	0.113	0.194	0.75	0.104	0.183

Table 89: Degree-normalized network-centric evaluations with t = 20 for COADREAD data with filtration based on (Muiños et al., 2021).

(a) Metrics with c= X_1

Method	Precision	Sensitivity	F1 Score	$\text{Precision}_{\text{strict}}$	$\text{Sensitivity}_{\text{strict}}$	F1 $_{\text{strict}}$ Score
DISCOVER	0.813	0.3	0.696	0.844	0.271	0.692
DISCOVER Strat	0.883	0.16	0.604	0.89	0.154	0.601
Fisher's Exact Test	0.91	0.119	0.691	0.902	0.112	0.671
MEMO	0.803	0.35	0.723	0.776	0.285	0.723
WExT	0.801	0.401	0.711	0.822	0.36	0.7
MEGSA	0.92	0.133	0.721	0.908	0.124	0.696

(b) Metrics with c= X_2

Method	Precision	Sensitivity	F1 Score	$\text{Precision}_{\text{strict}}$	$\text{Sensitivity}_{\text{strict}}$	F1 $_{\text{strict}}$ Score
DISCOVER	0.691	0.79	0.704	0.686	0.256	0.721
DISCOVER Strat	0.611	0.85	0.609	0.58	0.132	0.606
Fisher's Exact Test	0.801	0.964	0.762	0.75	0.093	0.687
WExT	0.699	0.748	0.696	0.74	0.344	0.714
MEMO	0.704	0.782	0.728	0.685	0.276	0.77
MEGSA	0.836	0.959	0.813	0.79	0.119	0.741

Table 90: Results of network-centric ME evaluation framework run on the tissue-specific network (TSN) for COADREAD data with filtration based on (Muiños et al., 2021).

(a) Metrics for COADREAD data on the TSN network constructed with co-expression ratio threshold 0.0, control group X_1 and $t = 20$ (Colon tissue | 779 samples | 194 CGC-CGC pairs)

Method	Precision	Sensitivity	F1 Score	$\text{Precision}_{\text{strict}}$	$\text{Sensitivity}_{\text{strict}}$	F1 $_{\text{strict}}$ Score
ISCOVER	0.612	0.277	0.381	0.667	0.213	0.323
DISCOVER Strat	0.667	0.165	0.264	0.711	0.148	0.245
Fisher's Exact Test	0.615	0.125	0.207	0.655	0.099	0.172
WExT	0.643	0.388	0.484	0.704	0.307	0.428
MEMO	0.614	0.329	0.428	0.542	0.207	0.3
MEGSA	0.634	0.135	0.223	0.667	0.104	0.18

(b) Metrics for COADREAD data on the TSN network constructed with co-expression ratio threshold 0.5, control group X_1 and $t = 20$ (Colon tissue | 779 samples | 166 CGC-CGC pairs)

Method	Precision	Sensitivity	F1 Score	$\text{Precision}_{\text{strict}}$	$\text{Sensitivity}_{\text{strict}}$	F1 $_{\text{strict}}$ Score
DISCOVER	0.618	0.322	0.423	0.689	0.259	0.376
DISCOVER Strat	0.681	0.196	0.305	0.718	0.177	0.284
Fisher's Exact Test	0.615	0.146	0.236	0.655	0.116	0.197
WExT	0.653	0.453	0.535	0.729	0.368	0.489
MEMO	0.641	0.399	0.492	0.591	0.255	0.356
MEGSA	0.634	0.159	0.254	0.667	0.122	0.206

(c) Metrics for COADREAD data on the TSN network constructed with co-expression ratio threshold 0.0, control group X_2 and $t = 20$ (Colon tissue | 779 samples | 105 CGC-CGC pairs)

Method	Precision	Sensitivity	F1 Score	$\text{Precision}_{\text{strict}}$	$\text{Sensitivity}_{\text{strict}}$	F1 $_{\text{strict}}$ Score
DISCOVER	0.548	0.356	0.431	0.562	0.24	0.336
DISCOVER Strat	0.526	0.192	0.282	0.483	0.135	0.211
Fisher's Exact Test	0.517	0.148	0.23	0.436	0.081	0.137
WExT	0.549	0.429	0.481	0.558	0.276	0.369
MEMO	0.561	0.399	0.466	0.436	0.197	0.271
MEGSA	0.53	0.167	0.255	0.465	0.096	0.159

(d) Metrics for COADREAD data on the TSN network constructed with co-expression ratio threshold 0.5, control group X_1 and $t = 20$ (Colon tissue | 779 samples | 86 CGC-CGC pairs)

Method	Precision	Sensitivity	F1 Score	$\text{Precision}_{\text{strict}}$	$\text{Sensitivity}_{\text{strict}}$	F1 $_{\text{strict}}$ Score
DISCOVER	0.556	0.417	0.476	0.585	0.286	0.384
DISCOVER Strat	0.526	0.235	0.325	0.483	0.165	0.246
Fisher's Exact Test	0.533	0.186	0.276	0.45	0.105	0.17
WExT	0.55	0.524	0.537	0.58	0.345	0.433
MEMO	0.569	0.488	0.526	0.444	0.238	0.31
MEGSA	0.529	0.209	0.3	0.455	0.116	0.185

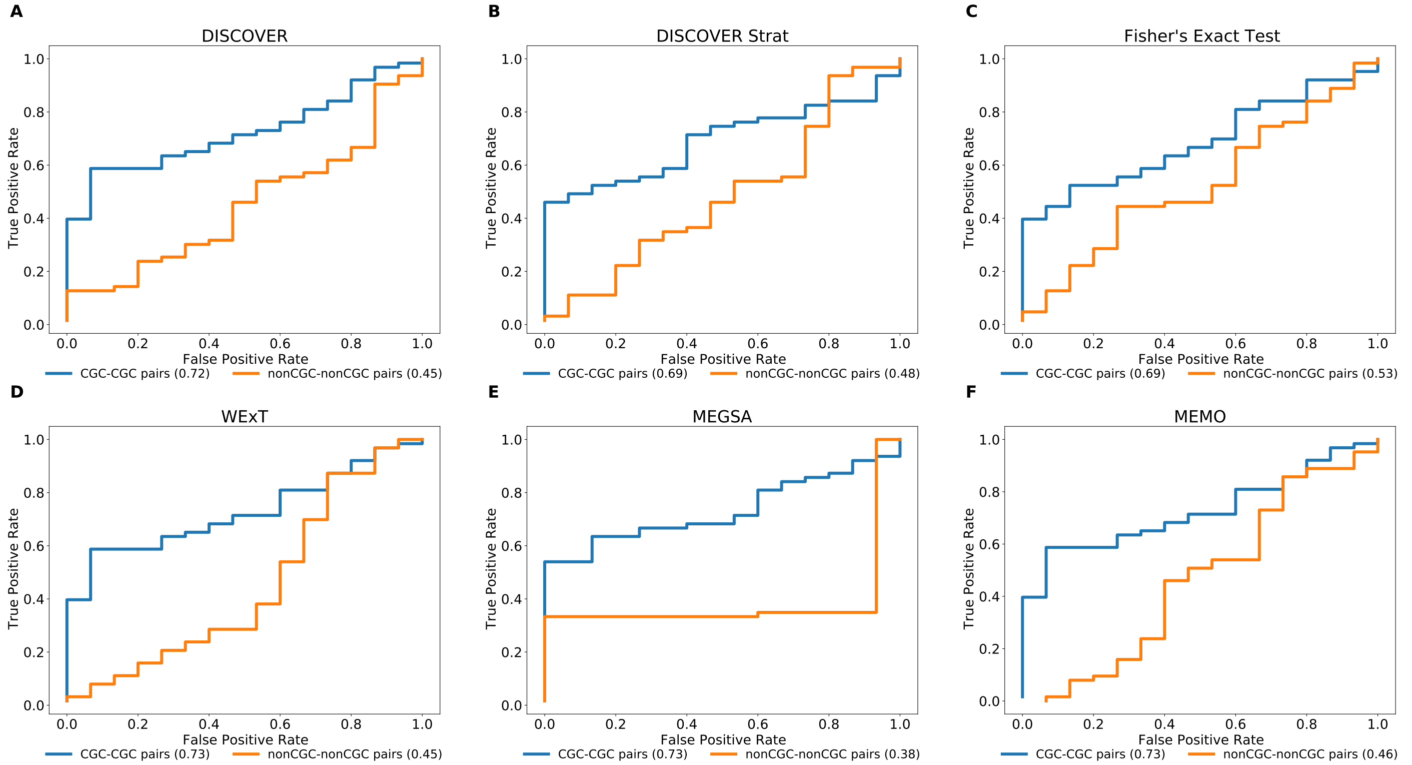

Fig 39: ROC curves for comparing the mutual exclusivities of tissue-specific and non-tissue-specific CGC-CGC gene pairs and non-CGC-non-CGC gene pairs on COADREAD data with filtration based on (Muiños et al., 2021). Results with $t = 20$ setting. (positive set: 63 edges, negative set: 15 edges)

Scatterplots of percentage significance of mutual exclusivity runs vs mutation load association (MLA) when $t = 20$ for COADREAD data with filtration based on (Muiños et al., 2021).

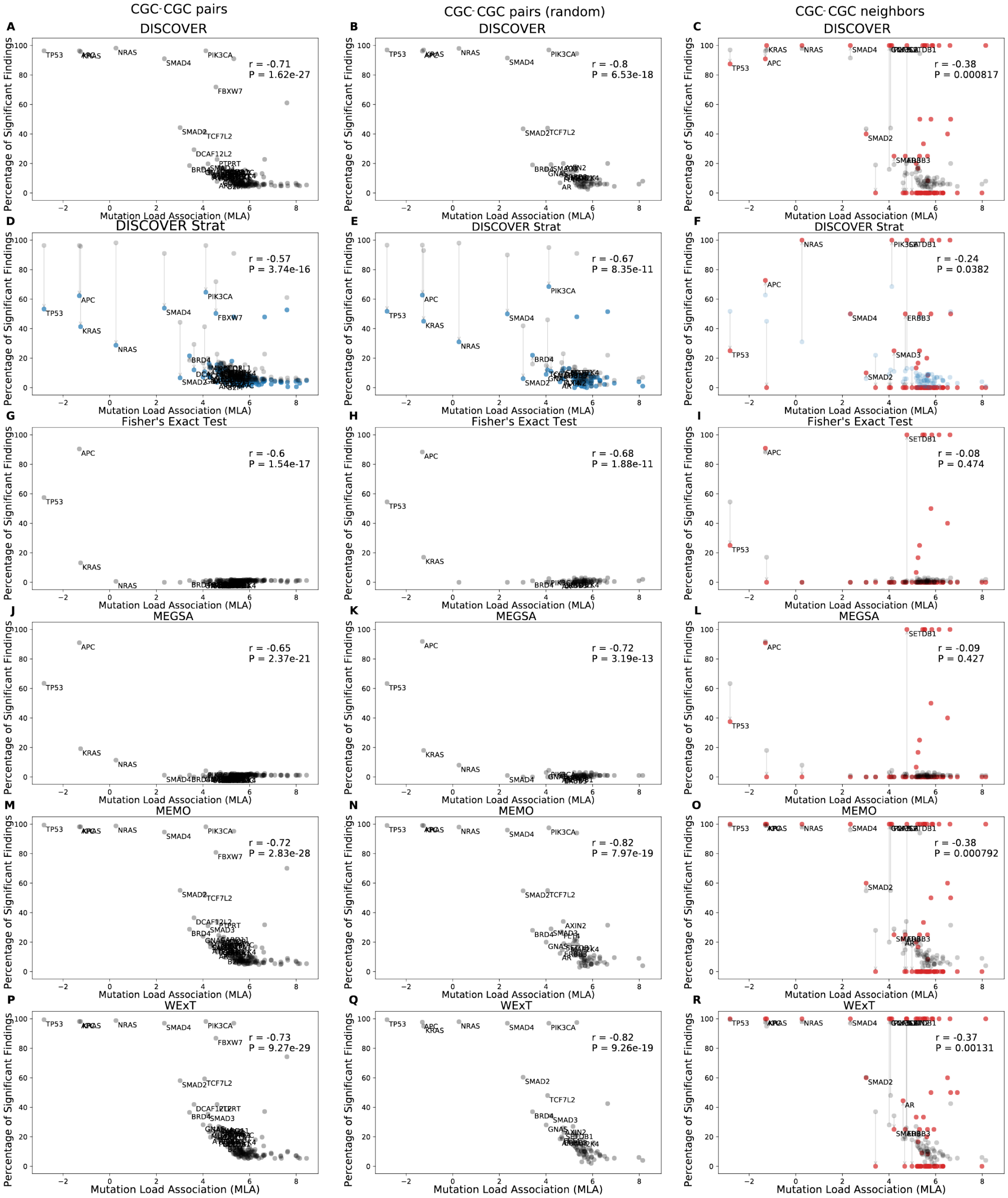

Fig 40: Comparison of ME results of all considered methods on TCGA COADREAD cohort (498 samples). The scatterplots show the percentage significance of ME runs (p-value<0.05) versus MLA values for CGC genes. (A) Results of DISCOVER where tests are performed between a CGC gene and all other CGC genes. B) Results of DISCOVER where tests are performed between a CGC gene and a random subset of all other CGC genes so that ME of a CGC gene of interest is checked with same sized group of genes in both B and C. (C) Results of DISCOVER where tests are performed between a CGC gene and its PPI neighbors that are in CGC (red) compared with (B) in gray. (D) Results of DISCOVER Strat where where tests are performed between a CGC gene and all other CGC genes (blue) compared with (A) in gray. (E) Results of DISCOVER Strat where where tests are performed between a CGC gene and a random subset of all other CGC genes so that ME of a CGC gene of interest is checked with same sized group of genes in both E and F. Values are shown in blue and compared with (B) in gray. (F) Results of DISCOVER where tests are performed between a CGC gene and its PPI neighbors that are in CGC (red) compared with (E) in blue. Analogous results are shown for Fisher's Exact Test (G, H, I), MEGSA (J, K, L), MEMO (M, N, O) and WExT (P, Q, R) where coloring is the same as previously described for (C).

Table 91: Results of network-centric ME evaluation framework for a subset of genes of COADREAD data (genes with mutation frequencies <5% and >30% are removed)

(a) Metrics with $\mathcal{G}$ = Intact (w conf. threshold 0.35), $\mathcal{S}$ = CGC, c= X_1, t=20, p_t = 0.05, robustness_iterations = 100. (498 samples | 60 CGC-CGC pairs)

Method	Precision	Sensitivity	F1 Score	$\text{Precision}_{\text{strict}}$	$\text{Sensitivity}_{\text{strict}}$	F1 $_{\text{strict}}$ Score
DISCOVER	0.81	0.149	0.252	0.895	0.149	0.255
DISCOVER Strat	1	0.035	0.068	1	0.035	0.068
Fisher's Exact Test	NaN	0	NaN	NaN	0	NaN
WExT	0.812	0.232	0.361	0.929	0.232	0.371
MEGSA	1	0.033	0.065	1	0.033	0.064
MEMO	0.75	0.164	0.269	0.9	0.164	0.277

(b) Metrics with $\mathcal{G}$ = Intact (w conf. threshold 0.35), $\mathcal{S}$ = CGC, c= X_2, t=5, p_t = 0.05, robustness_iterations = 100. (498 samples | 21 CGC-CGC pairs)

Method	Precision	Sensitivity	F1 Score	$\text{Precision}_{\text{strict}}$	$\text{Sensitivity}_{\text{strict}}$	F1 $_{\text{strict}}$ Score
DISCOVER	0.5	0.19	0.276	0.667	0.19	0.296
DISCOVER Strat	0.667	0.095	0.167	0.667	0.095	0.166
Fisher's Exact Test	NaN	0	NaN	NaN	0	NaN
WExT	0.556	0.238	0.333	0.714	0.238	0.357
MEGSA	1	0.095	0.174	1	0.095	0.174
MEMO	0.5	0.19	0.276	0.667	0.19	0.296

Table 92: Degree-normalized network-centric evaluations t = 20 for Results of network-centric ME evaluation framework for a subset of genes of COADREAD data (genes with mutation frequencies <5% and >30% are removed)

(a) Metrics with c= X_1

Method	Precision	Sensitivity	F1 Score	$\text{Precision}_{\text{strict}}$	$\text{Sensitivity}_{\text{strict}}$	F1 $_{\text{strict}}$ Score
DISCOVER	0.881	0.15	0.672	0.952	0.15	0.719
DISCOVER Strat	1	0.061	1	1	0.061	1
Fisher's Exact Test	NaN	0	NaN	NaN	0	NaN
WExT	0.795	0.27	0.694	0.932	0.27	0.784
MEGSA	1	0.061	1	1	0.061	1
MEMO	0.833	0.157	0.65	0.952	0.157	0.726

(b) Metrics with c= X_2

Method	Precision	Sensitivity	F1 Score	$\text{Precision}_{\text{strict}}$	$\text{Sensitivity}_{\text{strict}}$	F1 $_{\text{strict}}$ Score
DISCOVER	0.5	0.667	0.639	0.667	0.26	0.806
DISCOVER Strat	0.667	0.917	1	0.667	0.167	1
Fisher's Exact Test	NaN	1	NaN	NaN	0	NaN
WExT	0.571	0.667	0.611	0.714	0.288	0.744
MEGSA	1	1	1	1	0.167	1
MEMO	0.5	0.667	0.639	0.667	0.26	0.806

Results of network-centric ME evaluation framework run on the tissue-specific network (TSN) with $t = 20$

Table 93: Metrics for COADREAD data (genes with mutation frequencies <5% and >30% are removed) on the TSN networks.

(a) Metrics for COADREAD data on the TSN network constructed with co-expression ratio threshold 0.0, control group X_1 and $t = 20$ (Colon tissue | 779 samples | 60 CGC-CGC pairs)

Method	Precision	Sensitivity	F1 Score	$\text{Precision}_{\text{strict}}$	$\text{Sensitivity}_{\text{strict}}$	F1 $_{\text{strict}}$ Score
DISCOVER	0.8	0.143	0.242	0.889	0.143	0.246
DISCOVER Strat	1	0.033	0.065	1	0.033	0.064
Fisher's Exact Test	NaN	0	NaN	NaN	0	NaN
WExT	0.812	0.236	0.366	0.929	0.236	0.376
MEGSA	1	0.033	0.065	1	0.033	0.064
MEMO	0.731	0.167	0.271	0.857	0.158	0.267

(b) Metrics for COADREAD data on the TSN network constructed with co-expression ratio threshold 0.5, control group X_1 and $t = 20$ (Colon tissue | 779 samples | 58 CGC-CGC pairs)

Method	Precision	Sensitivity	F1 Score	$\text{Precision}_{\text{strict}}$	$\text{Sensitivity}_{\text{strict}}$	F1 $_{\text{strict}}$ Score
DISCOVER	0.889	0.152	0.26	1	0.152	0.264
DISCOVER Strat	1	0.034	0.067	1	0.034	0.066
Fisher's Exact Test	NaN	0	NaN	NaN	0	NaN
WExT	0.765	0.243	0.369	0.867	0.243	0.38
MEGSA	1	0.034	0.067	1	0.034	0.066
MEMO	0.75	0.171	0.279	0.9	0.171	0.287

(c) Metrics for COADREAD data on the TSN network constructed with co-expression ratio threshold 0.0, control group X_2 and $t = 20$ (Colon tissue | 779 samples | 21 CGC-CGC pairs)

Method	Precision	Sensitivity	F1 Score	$\text{Precision}_{\text{strict}}$	$\text{Sensitivity}_{\text{strict}}$	F1 $_{\text{strict}}$ Score
DISCOVER	0.5	0.19	0.276	0.667	0.19	0.296
DISCOVER Strat	0.667	0.095	0.167	0.667	0.095	0.166
Fisher's Exact Test	NaN	0	NaN	NaN	0	NaN
WExT	0.556	0.238	0.333	0.714	0.238	0.357
MEGSA	1	0.095	0.174	1	0.095	0.174
MEMO	0.5	0.19	0.276	0.667	0.19	0.296

(d) Metrics for PCAWG COADREAD data on the TSN network constructed with co-expression ratio threshold 0.5, control group X_1 and $t = 20$ (Colon tissue | 779 samples | 20 CGC-CGC pairs)

Method	Precision	Sensitivity	F1 Score	$\text{Precision}_{\text{strict}}$	$\text{Sensitivity}_{\text{strict}}$	F1 $_{\text{strict}}$ Score
DISCOVER	0.571	0.2	0.296	0.8	0.2	0.32
DISCOVER Strat	0.667	0.1	0.174	0.667	0.1	0.174
Fisher's Exact Test	NaN	0	NaN	NaN	0	NaN
WExT	0.625	0.25	0.357	0.833	0.25	0.385
MEGSA	1	0.1	0.182	1	0.1	0.182
MEMO	0.571	0.2	0.296	0.8	0.2	0.32

Scatterplots of percentage significance of mutual exclusivity runs vs mutation load association (MLA) when $t = 20$ for COADREAD data

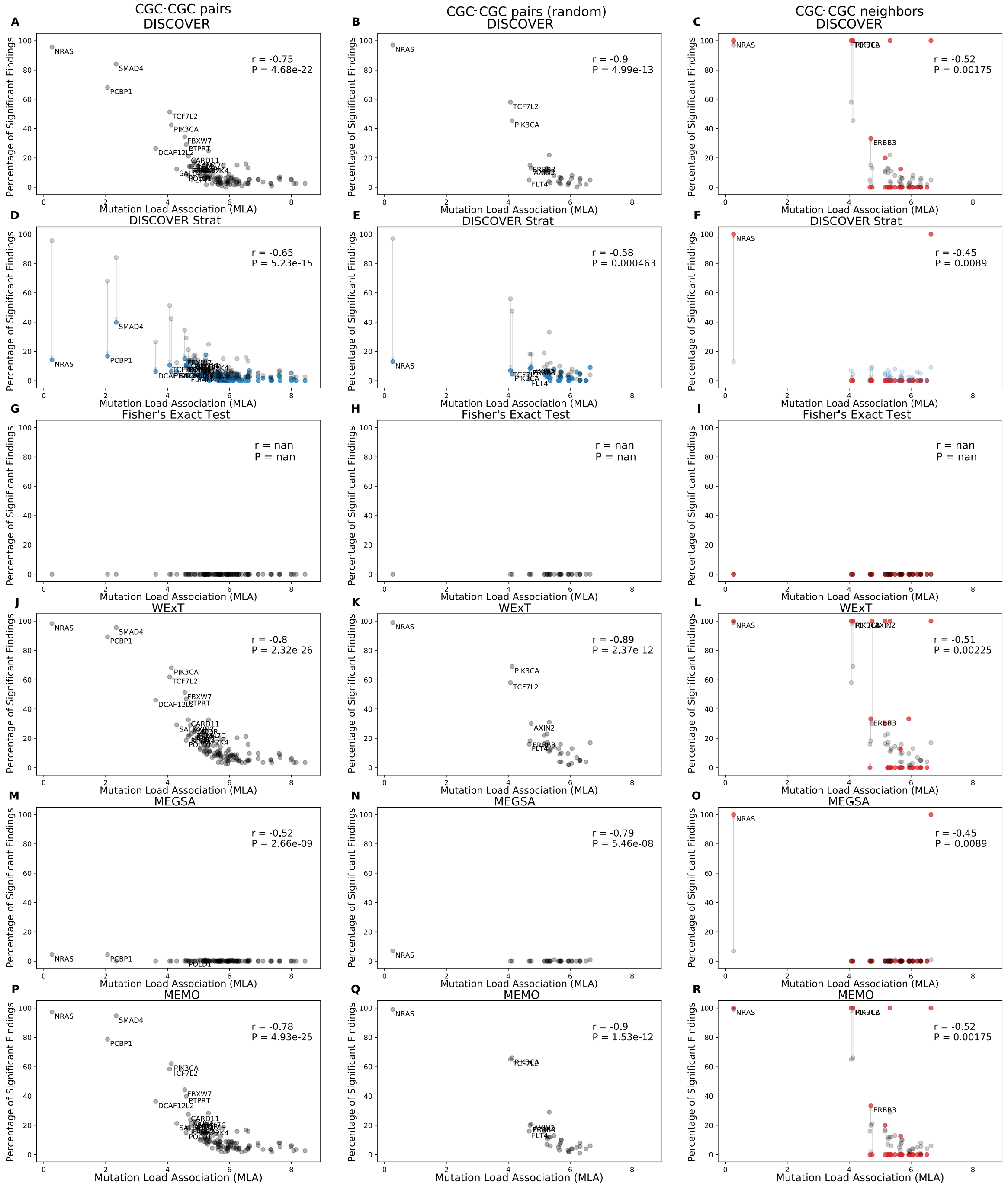

Fig 41: Comparison of ME results of all considered methods on TCGA COADREAD cohort (genes with mutation frequencies $<5\%$ and $>30\%$ are removed) with $t = 20$ (498 samples). The scatterplots show the percentage significance of ME runs (p-value<0.05) versus MLA values for CGC genes. (A) Results of DISCOVER where tests are performed between a CGC gene and all other CGC genes. B) Results of DISCOVER where tests are performed between a CGC gene and a random subset of all other CGC genes so that ME of a CGC gene of interest is checked with same sized group of genes in both B and C. (C) Results of DISCOVER where tests are performed between a CGC gene and its PPI neighbors that are in CGC (red) compared with (B) in gray. (D) Results of DISCOVER Strat where where tests are performed between a CGC gene and all other CGC genes (blue) compared with (A) in gray. (E) Results of DISCOVER Strat where where tests are performed between a CGC gene and a random subset of all other CGC genes so that ME of a CGC gene of interest is checked with same sized group of genes in both E and F. Values are shown in blue and compared with (B) in gray. (F) Results of DISCOVER where tests are performed between a CGC gene and its PPI neighbors that are in CGC (red) compared with (E) in blue. Analogous results are shown for Fisher's Exact Test (G, H, I), WExT (J, K, L), MEGSA (M, N, O) and MEMO (P, Q, R) where coloring is the same as previously described for (C).

Table 94: Results of network-centric ME evaluation framework for COADREAD data with results with c=X_1.

(a) Metrics with $\mathcal{G}$ = Intact (w conf. threshold 0.35), $\mathcal{S}$ = CGC, c= X_1, t=20, p_t = 0.05, $SELECT_t$ = 0.97323 (MAX), robustness_iterations = 100. (498 samples | 126 CGC-CGC pairs for which SELECT results are available)

Method	Precision	Sensitivity	F1 Score	$\text{Precision}_{\text{strict}}$	$\text{Sensitivity}_{\text{strict}}$	F1 $_{\text{strict}}$ Score
DISCOVER	0.641	0.792	0.47	0.673	0.299	0.414
DISCOVER Strat	0.571	0.948	0.124	0.538	0.061	0.11
Fisher's Exact Test	0.571	0.952	0.116	0.538	0.056	0.101
WExT	0.651	0.636	0.664	0.679	0.518	0.588
MEGSA	0.647	0.952	0.154	0.625	0.079	0.14
SELECT	0.467	0.856	0.199	0.467	0.126	0.198
MEMO	0.633	0.687	0.582	0.634	0.391	0.484

(b) Metrics with $\mathcal{G}$ = Intact (w conf. threshold 0.35), $\mathcal{S}$ = CGC, c= X_1, t=20, p_t = 0.05, $SELECT_t$ = 0.97323 (MAX), $SELECT(Subtypes)_t$ = 0.95539 (MAX), robustness_iterations = 100. (498 samples | 108 CGC-CGC pairs for which $SELECT_{subtypes}$ results are available)

Method	Precision	Sensitivity	F1 Score	$\text{Precision}_{\text{strict}}$	$\text{Sensitivity}_{\text{strict}}$	F1 $_{\text{strict}}$ Score
DISCOVER	0.631	0.381	0.475	0.69	0.297	0.415
DISCOVER Strat	0.64	0.077	0.138	0.64	0.077	0.137
Fisher's Exact Test	0.583	0.065	0.118	0.545	0.056	0.102
WExT	0.621	0.656	0.638	0.669	0.495	0.569
MEGSA	0.657	0.108	0.185	0.613	0.089	0.155
SELECT	0.553	0.149	0.234	0.553	0.149	0.235
SELECT (subtypes)	0.513	0.201	0.289	0.514	0.191	0.279
MEMO	0.603	0.482	0.536	0.589	0.323	0.417

(c) Metrics with $\mathcal{G}$ = Intact (w conf. threshold 0.35), $\mathcal{S}$ = CGC, c= X_1, t=20, p_t = 0.05, $SELECT_t$ = 0.97098 (AVG), robustness_iterations = 100. (498 samples | 126 CGC-CGC pairs for which SELECT results are available)

Method	Precision	Sensitivity	F1 Score	$\text{Precision}_{\text{strict}}$	$\text{Sensitivity}_{\text{strict}}$	F1 $_{\text{strict}}$ Score
DISCOVER	0.641	0.355	0.457	0.68	0.294	0.411
DISCOVER Strat	0.636	0.061	0.112	0.636	0.061	0.111
Fisher's Exact Test	0.538	0.056	0.101	0.538	0.056	0.101
WExT	0.661	0.681	0.671	0.698	0.524	0.599
MEGSA	0.605	0.091	0.158	0.571	0.079	0.139
SELECT	0.615	0.07	0.125	0.615	0.07	0.126
MEMO	0.633	0.546	0.586	0.629	0.388	0.48

(d) Metrics with $\mathcal{G}$ = Intact (w conf. threshold 0.35), $\mathcal{S}$ = CGC, c= X_1, t=20, p_t = 0.05, $SELECT_t$ = 0.97098 (AVG), $SELECT(Subtypes)_t$ = 0.95282 (AVG), robustness_iterations = 100. (498 samples | 108 CGC-CGC pairs for which $SELECT_{subtypes}$ results are available)

Method	Precision	Sensitivity	F1 Score	$\text{Precision}_{\text{strict}}$	$\text{Sensitivity}_{\text{strict}}$	F1 $_{\text{strict}}$ Score
DISCOVER	0.632	0.411	0.498	0.67	0.307	0.421
DISCOVER Strat	0.615	0.08	0.141	0.615	0.08	0.142
Fisher's Exact Test	0.571	0.074	0.132	0.5	0.056	0.101
WExT	0.62	0.646	0.633	0.657	0.479	0.554
MEGSA	0.667	0.112	0.192	0.625	0.093	0.162
SELECT	0.64	0.08	0.143	0.64	0.08	0.142
SELECT (subtypes)	0.615	0.079	0.14	0.615	0.079	0.14
MEMO	0.598	0.488	0.537	0.571	0.318	0.408

Table 95: Results of network-centric ME evaluation framework for COADREAD data with results with c=X_2.

(a) Metrics with $\mathcal{G}$ = Intact (w conf. threshold 0.35), $\mathcal{S}$ = CGC, c= X_2, t=20, p_t = 0.05, $SELECT_t$ = 0.97323 (MAX), robustness_iterations = 100. (498 samples | 50 CGC-CGC pairs for which SELECT results are available)

Method	Precision	Sensitivity	F1 Score	$\text{Precision}_{\text{strict}}$	$\text{Sensitivity}_{\text{strict}}$	F1 $_{\text{strict}}$ Score
DISCOVER	0.51	0.52	0.515	0.529	0.36	0.428
DISCOVER Strat	0.545	0.122	0.2	0.6	0.122	0.203
Fisher's Exact Test	0.5	0.083	0.143	0.5	0.083	0.142
WExT	0.535	0.776	0.633	0.535	0.469	0.5
MEGSA	0.5	0.13	0.206	0.458	0.11	0.177
SELECT	0.368	0.151	0.214	0.375	0.129	0.192
MEMO	0.53	0.729	0.614	0.436	0.354	0.391

(b) Metrics with $\mathcal{G}$ = Intact (w conf. threshold 0.35), $\mathcal{S}$ = CGC, c= X_2, t=20, p_t = 0.05, $SELECT_t$ = 0.97323 (MAX), $SELECT(Subtypes)_t$ = 0.95539 (MAX), robustness_iterations = 100. (498 samples | 42 CGC-CGC pairs for which $SELECT_{subtypes}$ results are available)

Method	Precision	Sensitivity	F1 Score	$\text{Precision}_{\text{strict}}$	$\text{Sensitivity}_{\text{strict}}$	F1 $_{\text{strict}}$ Score
DISCOVER	0.535	0.561	0.548	0.593	0.39	0.471
DISCOVER Strat	0.458	0.136	0.21	0.381	0.099	0.157
Fisher's Exact Test	0.444	0.093	0.154	0.375	0.07	0.118
WExT	0.561	0.78	0.653	0.621	0.5	0.554
MEGSA	0.538	0.165	0.252	0.455	0.118	0.187
SELECT	0.421	0.205	0.276	0.444	0.205	0.28
SELECT (subtypes)	0.333	0.205	0.254	0.381	0.205	0.267
MEMO	0.545	0.714	0.619	0.469	0.357	0.405

(c) Metrics with $\mathcal{G}$ = Intact (w conf. threshold 0.35), $\mathcal{S}$ = CGC, c= X_2, t=20, p_t = 0.05, $SELECT_t$ = 0.97098 (AVG), robustness_iterations = 100. (498 samples | 50 CGC-CGC pairs for which SELECT results are available)

Method	Precision	Sensitivity	F1 Score	$\text{Precision}_{\text{strict}}$	$\text{Sensitivity}_{\text{strict}}$	F1 $_{\text{strict}}$ Score
DISCOVER	0.515	0.52	0.518	0.537	0.367	0.436
DISCOVER Strat	0.545	0.121	0.198	0.545	0.121	0.198
Fisher's Exact Test	0.444	0.08	0.136	0.375	0.06	0.103
WExT	0.531	0.778	0.631	0.54	0.475	0.505
MEGSA	0.583	0.143	0.23	0.545	0.122	0.199
SELECT	0.556	0.103	0.174	0.5	0.082	0.141
MEMO	0.515	0.722	0.601	0.45	0.371	0.407

(d) Metrics with $\mathcal{G}$ = Intact (w conf. threshold 0.35), $\mathcal{S}$ = CGC, c= X_2, t=20, p_t = 0.05, $SELECT_t$ = 0.97098 (AVG), $SELECT(Subtypes)_t$ = 0.95282 (AVG), robustness_iterations = 100. (498 samples | 42 CGC-CGC pairs for which $SELECT_{subtypes}$ results are available)

Method	Precision	Sensitivity	F1 Score	$\text{Precision}_{\text{strict}}$	$\text{Sensitivity}_{\text{strict}}$	F1 $_{\text{strict}}$ Score
DISCOVER	0.545	0.571	0.558	0.607	0.405	0.486
DISCOVER Strat	0.455	0.122	0.192	0.455	0.122	0.192
Fisher's Exact Test	0.444	0.095	0.157	0.375	0.071	0.119
WExT	0.561	0.78	0.653	0.667	0.537	0.595
MEGSA	0.538	0.163	0.25	0.455	0.116	0.185
SELECT	0.625	0.135	0.222	0.625	0.135	0.222
SELECT (subtypes)	0.476	0.125	0.198	0.5	0.125	0.2
MEMO	0.537	0.707	0.611	0.452	0.341	0.389

Table 95: Degree-normalized network-centric evaluations t = 20 for COADREAD data.

(e) Metrics with c= X_1, $SELECT_t$ = 0.97323 (MAX).

Method	Precision	Sensitivity	F1 Score	$\text{Precision}_{\text{strict}}$	$\text{Sensitivity}_{\text{strict}}$	F1 $_{\text{strict}}$ Score
DISCOVER	0.860	0.388	0.740	0.894	0.365	0.746
DISCOVER Strat	0.875	0.077	0.671	0.875	0.077	0.671
Fisher's Exact Test (SF)	0.794	0.038	0.436	0.767	0.036	0.495
WExT	0.821	0.725	0.816	0.830	0.642	0.806
MEGSA	0.881	0.095	0.641	0.867	0.093	0.626
SELECT	0.500	0.153	0.588	0.500	0.153	0.588
MeMo	0.782	0.555	0.770	0.779	0.486	0.780

(f) Metrics with c= X_1, $SELECT_t$ = 0.97323 (MAX), $SELECT(Subtypes)_t$ = 0.95539 (MAX).

Method	Precision	Sensitivity	F1 Score	$\text{Precision}_{\text{strict}}$	$\text{Sensitivity}_{\text{strict}}$	F1 $_{\text{strict}}$ Score
DISCOVER	0.767	0.386	0.727	0.798	0.34	0.715
DISCOVER Strat	0.725	0.086	0.64	0.725	0.086	0.64
Fisher's Exact Test	0.767	0.05	0.593	0.75	0.046	0.562
WExT	0.774	0.648	0.785	0.815	0.575	0.801
MEGSA	0.881	0.103	0.663	0.856	0.096	0.629
SELECT	NaN	0	NaN	NaN	0	NaN
SELECT (subtypes)	0.562	0.248	0.616	0.554	0.225	0.625
MEMO	0.71	0.507	0.754	0.703	0.411	0.75

(g) Metrics with c= X_2, $SELECT_t$ = 0.97323 (MAX).

Method	Precision	Sensitivity	F1 Score	$\text{Precision}_{\text{strict}}$	$\text{Sensitivity}_{\text{strict}}$	F1 $_{\text{strict}}$ Score
DISCOVER	0.581	0.502	0.642	0.619	0.387	0.659
DISCOVER Strat	0.531	0.789	0.573	0.531	0.161	0.573
Fisher's Exact Test	0.500	0.970	0.298	0.500	0.027	0.298
WExT	0.628	0.427	0.724	0.685	0.593	0.749
MEGSA	0.759	0.959	0.670	0.729	0.151	0.640
SELECT	0.400	0.729	0.563	0.450	0.231	0.619
MeMo	0.602	0.402	0.706	0.693	0.561	0.773

(h) Metrics with c= X_2, $SELECT_t$ = 0.97323 (MAX), $SELECT(Subtypes)_t$ = 0.95539 (MAX).

Method	Precision	Sensitivity	F1 Score	$\text{Precision}_{\text{strict}}$	$\text{Sensitivity}_{\text{strict}}$	F1 $_{\text{strict}}$ Score
DISCOVER	0.619	0.565	0.675	0.698	0.416	0.689
DISCOVER Strat	0.573	0.818	0.522	0.573	0.155	0.522
Fisher's Exact Test	0.425	0.951	0.391	0.375	0.032	0.313
WExT	0.667	0.471	0.757	0.746	0.596	0.805
MEGSA	0.733	0.947	0.63	0.675	0.116	0.558
SELECT	NaN	0	NaN	NaN	0	NaN
SELECT (subtypes)	0.354	0.583	0.546	0.424	0.252	0.622
MEMO	0.636	0.479	0.74	0.657	0.5	0.731

(a) Metrics with c= X_1, $SELECT_t$ = 0.97098 (AVG).

Method	Precision	Sensitivity	F1 Score	$\text{Precision}_{\text{strict}}$	$\text{Sensitivity}_{\text{strict}}$	F1 $_{\text{strict}}$ Score
DISCOVER	0.862	0.399	0.746	0.893	0.374	0.748
DISCOVER Strat	0.806	0.077	0.598	0.806	0.077	0.598
Fisher's Exact Test	0.792	0.036	0.506	0.792	0.036	0.506
WExT	0.807	0.732	0.81	0.803	0.651	0.793
MEGSA	0.865	0.095	0.636	0.841	0.091	0.61
SELECT	0.7	0.082	0.594	0.7	0.082	0.594
MeMo	0.768	0.569	0.775	0.767	0.499	0.785

(b) Metrics with c= X_1, $SELECT_t$ = 0.97098 (AVG), $SELECT(Subtypes)_t$ = 0.95282 (AVG).

Method	Precision	Sensitivity	F1 Score	$\text{Precision}_{\text{strict}}$	$\text{Sensitivity}_{\text{strict}}$	F1 $_{\text{strict}}$ Score
DISCOVER	0.799	0.408	0.744	0.825	0.359	0.729
DISCOVER Strat	0.725	0.078	0.600	0.725	0.078	0.600
Fisher's Exact Test	0.800	0.052	0.522	0.750	0.045	0.562
WExT	0.784	0.648	0.795	0.830	0.589	0.798
MEGSA	0.881	0.103	0.663	0.856	0.096	0.629
SELECT	NaN	0.000	NaN	NaN	0.000	NaN
SELECT (subtypes)	0.633	0.101	0.790	0.633	0.101	0.790
MEMO	0.764	0.485	0.749	0.757	0.395	0.745

(c) Metrics with c= X_2, $SELECT_t$ = 0.97098 (AVG).

Method	Precision	Sensitivity	F1 Score	$\text{Precision}_{\text{strict}}$	$\text{Sensitivity}_{\text{strict}}$	F1 $_{\text{strict}}$ Score
DISCOVER (SF)	0.594	0.538	0.651	0.654	0.414	0.64
DISCOVER Strat (SF)	0.536	0.789	0.507	0.607	0.161	0.573
Fisher's Exact Test (SF)	0.425	0.965	0.271	0.425	0.025	0.271
WExT (SF)	0.598	0.369	0.705	0.692	0.599	0.757
MEGSA (SF)	0.771	0.962	0.679	0.738	0.152	0.646
SELECT	0.5	0.798	0.609	0.438	0.138	0.594
MeMo (SF)	0.568	0.381	0.685	0.668	0.558	0.703

(d) Metrics with c= X_2, $SELECT_t$ = 0.97098 (AVG), $SELECT(Subtypes)_t$ = 0.95282 (AVG).

Method	Precision	Sensitivity	F1 Score	$\text{Precision}_{\text{strict}}$	$\text{Sensitivity}_{\text{strict}}$	F1 $_{\text{strict}}$ Score
DISCOVER	0.580	0.506	0.644	0.621	0.357	0.658
DISCOVER Strat	0.679	0.887	0.550	0.679	0.165	0.550
Fisher's Exact Test	0.400	0.947	0.356	0.250	0.020	0.400
WExT	0.667	0.471	0.757	0.666	0.525	0.772
MEGSA	0.733	0.947	0.630	0.675	0.116	0.558
SELECT	NaN	1.000	NaN	NaN	0.000	NaN
SELECT (subtypes)	0.560	0.867	0.730	0.607	0.228	0.770
MEMO	0.636	0.479	0.740	0.614	0.462	0.813

Results of network-centric ME evaluation framework run on the tissue-specific network (TSN) with $t = 20$

Table 97: Metrics for COADREAD data on the TSN networks.

(a) Metrics for COADREAD data on the TSN network constructed with co-expression ratio threshold 0.0, control group X_1, $SELECT_t$ = 0.97323 (MAX) and $t = 20$ (Colon tissue | 779 samples | 126 CGC-CGC pairs for which $SELECT$ results are available)

Method	Precision	Sensitivity	F1 Score	$\text{Precision}_{\text{strict}}$	$\text{Sensitivity}_{\text{strict}}$	F1 $_{\text{strict}}$ Score
DISCOVER	0.662	0.379	0.482	0.706	0.317	0.438
DISCOVER Strat	0.667	0.067	0.122	0.667	0.067	0.122
Fisher's Exact Test	0.6	0.049	0.091	0.6	0.049	0.091
WExT	0.658	0.682	0.67	0.685	0.514	0.587
MEGSA	0.611	0.088	0.153	0.588	0.08	0.141
SELECT	0.519	0.126	0.203	0.519	0.126	0.203
MeMo	0.63	0.538	0.581	0.62	0.376	0.468

(b) Metrics for COADREAD data on the TSN network constructed with co-expression ratio threshold 0.0, control group X_1, $SELECT_t$ = 0.97323 (MAX), $SELECT(Subtypes)_t$ = 0.95539 (MAX) and $t = 20$ (Colon tissue | 779 samples | 108 CGC-CGC pairs for which $SELECT_{subtypes}$ results are available)

Method	Precision	Sensitivity	F1 Score	$\text{Precision}_{\text{strict}}$	$\text{Sensitivity}_{\text{strict}}$	F1 $_{\text{strict}}$ Score
DISCOVER	0.625	0.402	0.489	0.689	0.312	0.43
DISCOVER Strat	0.615	0.078	0.139	0.615	0.078	0.138
Fisher's Exact Test	0.565	0.061	0.111	0.524	0.052	0.095
WExT	0.625	0.635	0.63	0.652	0.467	0.544
MEGSA	0.667	0.112	0.192	0.625	0.093	0.162
SELECT	0.54	0.148	0.233	0.531	0.143	0.225
SELECT (subtypes)	0.514	0.182	0.269	0.5	0.172	0.256
MEMO	0.593	0.492	0.538	0.564	0.318	0.407

(c) Metrics for COADREAD data on the TSN network constructed with co-expression ratio threshold 0.0, control group X_1, $SELECT_t$ = 0.97098 (AVG) and $t = 20$ (Colon tissue | 779 samples | 126 CGC-CGC pairs for which $SELECT$ results are available)

Method	Precision	Sensitivity	F1 Score	$\text{Precision}_{\text{strict}}$	$\text{Sensitivity}_{\text{strict}}$	F1 $_{\text{strict}}$ Score
DISCOVER	0.652	0.374	0.475	0.686	0.304	0.421
DISCOVER Strat	0.696	0.067	0.123	0.696	0.067	0.122
Fisher's Exact Test	0.533	0.063	0.113	0.5	0.056	0.101
WExT	0.675	0.691	0.683	0.714	0.538	0.614
MEGSA	0.647	0.089	0.156	0.625	0.081	0.143
SELECT	0.5	0.069	0.121	0.5	0.069	0.121
MeMo	0.636	0.538	0.583	0.639	0.393	0.487

(d) Metrics for COADREAD data on the TSN network constructed with co-expression ratio threshold 0.0, control group X_1, $SELECT_t$ = 0.97098 (AVG), $SELECT(Subtypes)_t$ = 0.95281 (AVG) and $t = 20$ (Colon tissue | 779 samples | 108 CGC-CGC pairs for which $SELECT_{subtypes}$ results are available)

Method	Precision	Sensitivity	F1 Score	$\text{Precision}_{\text{strict}}$	$\text{Sensitivity}_{\text{strict}}$	F1 $_{\text{strict}}$ Score
DISCOVER	0.626	0.41	0.495	0.685	0.315	0.432
DISCOVER Strat	0.652	0.075	0.135	0.652	0.075	0.135
Fisher's Exact Test	0.571	0.074	0.131	0.5	0.056	0.101
WExT	0.636	0.646	0.641	0.681	0.49	0.57
MEGSA	0.667	0.111	0.19	0.625	0.093	0.162
SELECT	0.552	0.082	0.142	0.552	0.082	0.143
SELECT (subtypes)	0.615	0.079	0.14	0.615	0.079	0.14
MEMO	0.592	0.492	0.538	0.574	0.328	0.417

(e) Metrics for COADREAD data on the TSN network constructed with co-expression ratio threshold 0.0, control group X_2, $SELECT_t$ = 0.97323 (MAX) and $t = 20$ (Colon tissue | 779 samples | 50 CGC-CGC pairs for which $SELECT$ results are available)

Method	Precision	Sensitivity	F1 Score	$\text{Precision}_{\text{strict}}$	$\text{Sensitivity}_{\text{strict}}$	F1 $_{\text{strict}}$ Score
DISCOVER	0.52	0.531	0.525	0.588	0.408	0.482
DISCOVER Strat	0.522	0.121	0.197	0.571	0.121	0.2
Fisher's Exact Test	0.5	0.082	0.14	0.5	0.082	0.141
WExT	0.535	0.792	0.639	0.571	0.5	0.533
MEGSA	0.583	0.143	0.23	0.545	0.122	0.199
SELECT	0.412	0.146	0.215	0.438	0.146	0.219
MeMo	0.522	0.732	0.609	0.456	0.371	0.409

(f) Metrics for COADREAD data on the TSN network constructed with co-expression ratio threshold 0.5, control group X_2, $SELECT_t$ = 0.97323 (MAX), $SELECT(Subtypes)_t$ = 0.95539 (MAX) and $t = 20$ (Colon tissue | 779 samples | 35 CGC-CGC pairs for which $SELECT_{subtypes}$ results are available)

Method	Precision	Sensitivity	F1 Score	$\text{Precision}_{\text{strict}}$	$\text{Sensitivity}_{\text{strict}}$	F1 $_{\text{strict}}$ Score
DISCOVER	0.535	0.648	0.586	0.593	0.451	0.512
DISCOVER Strat	0.423	0.159	0.232	0.435	0.145	0.218
Fisher's Exact Test	0.5	0.114	0.186	0.429	0.086	0.143
WExT	0.532	0.853	0.655	0.617	0.544	0.578
MEGSA	0.538	0.194	0.286	0.455	0.139	0.213
SELECT	0.438	0.226	0.298	0.5	0.226	0.311
SELECT (subtypes)	0.298	0.212	0.248	0.378	0.212	0.272
MEMO	0.509	0.824	0.629	0.452	0.412	0.431

(g) Metrics for COADREAD data on the TSN network constructed with co-expression ratio threshold 0.0, control group X_2, $SELECT_t$ = 0.97098 (AVG) and $t = 20$ (Colon tissue | 779 samples | 50 CGC-CGC pairs for which $SELECT$ results are available)

Method	Precision	Sensitivity	F1 Score	$\text{Precision}_{\text{strict}}$	$\text{Sensitivity}_{\text{strict}}$	F1 $_{\text{strict}}$ Score
DISCOVER	0.515	0.515	0.515	0.552	0.374	0.446
DISCOVER Strat	0.522	0.122	0.198	0.55	0.112	0.186
Fisher's Exact Test	0.5	0.082	0.14	0.5	0.082	0.141
WExT	0.529	0.787	0.632	0.585	0.511	0.546
MEGSA	0.56	0.141	0.226	0.522	0.121	0.196
SELECT	0.556	0.102	0.172	0.556	0.102	0.172
MeMo	0.525	0.737	0.613	0.457	0.374	0.411

(h) Metrics for COADREAD data on the TSN network constructed with co-expression ratio threshold 0.5, control group X_2, $SELECT_t$ = 0.97098 (AVG) , $SELECT(Subtypes)_t$ = 0.95281 (AVG) and $t = 20$ (Colon tissue | 779 samples | 35 CGC-CGC pairs for which $SELECT_{subtypes}$ results are available)

Method	Precision	Sensitivity	F1 Score	$\text{Precision}_{\text{strict}}$	$\text{Sensitivity}_{\text{strict}}$	F1 $_{\text{strict}}$ Score
DISCOVER	0.524	0.647	0.579	0.615	0.471	0.533
DISCOVER Strat	0.5	0.147	0.227	0.5	0.147	0.227
Fisher's Exact Test	0.471	0.113	0.182	0.4	0.085	0.14
WExT	0.537	0.853	0.659	0.617	0.544	0.578
MEGSA	0.583	0.197	0.295	0.5	0.141	0.22
SELECT	0.714	0.161	0.263	0.714	0.161	0.263
SELECT (subtypes)	0.545	0.182	0.273	0.6	0.182	0.279
MEMO	0.509	0.824	0.629	0.452	0.412	0.431

References

J. Das and H. Yu. HINT: High-quality protein interactomes and their applications in understanding human disease. *BMC Systems Biology*, 6:92, 2012.

Mark D. M. Leiserson, Fabio Vandin, Hsin-Ta Wu, et al. Pan-cancer network analysis identifies combinations of rare somatic mutations across pathways and protein complexes. *Nat Genet*, 47(2):106–114, 2015. ISSN 1546-1718. doi: 10.1038/ng.3168.

F Martínez-Jiménez, F Muinos, I Senis, et al. A compendium of mutational cancer driver genes. *Nat Rev Cancer*, 20:555–572, 2020.

F Muinos, F Martinez-Jimenes, O Pich, A Gonzalez-Perez, and N. Lopez-Bigas. In silico saturation mutagenesis of cancer genes. *Nature*, 596:428–432, 2021.

P Porras, E Barrera, A Bridge, et al. Towards a unified open access dataset of molecular interactions. *Nat Comm*, 11(6144), 2020. doi: 10.1038/s41467-020-19942-z.

Lyon D Szklarczyk D, Gable AL et al. String v11: protein–protein association networks with increased coverage, supporting functional discovery in genome-wide experimental datasets. *NAR*, 47(D1), 2018. doi: 10.1093/nar/gky1131.

E Sügis, J Dauvillier, A Leontjeva, et al. Hena, heterogeneous network-based data set for alzheimer's disease. *Scientific Data*, 6(151), 2019. doi: 10.1038/s41597-019-0152-0.

H. Yu, L. Tardivo, S. Tam, et al. Next-generation sequencing to generate interactome datasets. *Nature Methods*, 8:478–480, 2011.
